# Host transcriptome and microbiome interaction modulates physiology of full-sibs broilers with divergent feed conversion ratio

**DOI:** 10.1038/s41522-019-0096-3

**Published:** 2019-09-20

**Authors:** Tejas M. Shah, Jignasha G. Patel, Tejas P. Gohil, Damer P. Blake, Chaitanya G. Joshi

**Affiliations:** 1 Department of Animal Biotechnology, College of Veterinary Science & Animal Husbandry, Anand Agricultural University, Anand, Gujarat India; 2 Department of Pathology and Population Sciences, Royal Veterinary College, Hertfordshire, UK; 3 Gujarat Biotechnology Research Centre, Department of Science and Technology, Government of Gujarat, Gandhinagar, Gujarat India

**Keywords:** Microbiome, Metagenomics

## Abstract

Efficient livestock production relies on effective conversion of feed into body weight gain (BWG). High levels of feed conversion are especially important in production of broiler chickens, birds reared for meat, where economic margins are tight. Traits associated with improved broiler growth and feed efficiency have been subjected to intense genetic selection, but measures such as feed conversion ratio (FCR) remain variable, even between full siblings (sibs). Non-genetic factors such as the composition and function of microbial populations within different enteric compartments have been recognized to influence FCR, although the extent of interplay between hosts and their microbiomes is unclear. To examine host–microbiome interactions we investigated variation in the composition and functions of host intestinal-hepatic transcriptomes and the intestinal microbiota of full-sib broilers with divergent FCR. Progeny from 300 broiler families were assessed for divergent FCR set against shared genetic backgrounds and exposure to the same environmental factors. The seven most divergent full-sib pairs were chosen for analysis, exhibiting marked variation in transcription of genes as well as gut microbial diversity. Examination of enteric microbiota in low FCR sibs revealed variation in microbial community structure and function with no difference in feed intake compared to high FCR sibs. Gene transcription in low and high FCR sibs was significantly associated with the abundance of specific microbial taxa. Highly intertwined interactions between host transcriptomes and enteric microbiota are likely to modulate complex traits like FCR and may be amenable to selective modification with relevance to improving intestinal homeostasis and health.

## Introduction

The global human population has been predicted to exceed 9 billion by the year 2050,^1^ prompting the challenge to produce sufficient quantities of safe food for all. The challenge is especially relevant in low and middle income countries (LMICs) where it is being met, in part, by a dramatic expansion in the production of poultry eggs and meat.^2^ More than 1.1 trillion eggs are currently produced every year and the chicken population is predicted to continue expanding from the record ~64 billion currently produced per annum.^3^ To meet demand, improving the scale and efficiency of poultry production will be essential.

The efficiency of food use makes a major contribution to the economic sustainability of broiler production (chickens reared for meat), where feed represents the greatest variable cost.^4^ Feed efficiency can be measured using the feed conversion ratio (FCR), which is a composite trait defined as feed intake per unit of body weight gain (BWG) during a specified period of time.^5^ Genetic selection of livestock based on low FCR can improve efficiency of energy utilization without reducing the capacity for feed intake,^6^ indicating its importance for commercial and ecological aspects of production. As a complex feed efficiency trait, FCR is influenced by livestock genetics and the environment. FCR has shown a moderate heritability in poultry (0.21–0.49), but it has been used as a selection criterion in selective breeding strategies.^7–9^ Several quantitative trait loci (QTL) have been shown by genome wide association and linkage analyses to contribute to FCR,^10–12^ but the genetic evidence has been insufficient to explain the physiology of individuals with extreme differences in trait performance. Transcriptome profiling multiple tissues from divergently selected individuals has revealed important biological and metabolic processes that associate with feed efficiency traits.^13–15^ However, it has also been proposed that the intestinal microbiota should be considered as an important “metabolic organ” with major relevance to feed efficiency.^16^ Thus, monitoring transcriptome and microbiota profile variation between broilers with extreme FCRs offers a new opportunity to define novel contributory mechanisms.

In recent years, transcriptome profiling by RNA sequencing (RNASeq) and microbiota profiling by 16S rDNA amplicon or metagenomics whole genome shotgun (WGS) sequencing have emerged as powerful high-throughput approaches to quantify host gene expression and survey microbiota composition.^17,18^ To gain new insight into the complex traits and underlying functional mechanisms that govern feed efficiency, we have used a combined approach to profile global intestinal and liver tissue transcriptomes and define the associated intestinal luminal microbiota, including differential analysis from full-sib broilers with divergent FCR. This approach has enabled us to identify functional and metabolic processes as well as microbes that might contribute to good feed conversion efficiency in broilers. Outputs include associations between specific gene expression levels and FCR, as well as identification of putatively beneficial bacteria that may be suitable for use as probiotics against specific host genetic backgrounds. Following this approach, we were able to identify the molecular signatures of the host-microbiota “meta-organism” that are likely to contribute to the host system physiology of chickens with low FCR.

## Results

### Growth and feed efficiency parameters from full sibling chickens selected for variation in FCR

In total 300 batches of ten × full-sib Pure-Line Marshall Breed chickens were assessed for variation in food conversion ratio (FCR) and BWG/body weight at 49 days of age (BW49), identifying seven full-sib pairs with the greatest level of variation by 49 days (Supplementary Fig. 1). We observed growth performance and feed efficiency in Full-sib chickens that possessed different FCR values and defined two groups of individuals with low and the high FCR values. At the beginning of the experimental trial, the average BWs of individuals at 35 d of age that were ultimately found to have low and high FCR were 888.71 ± 64.65 and 942.57 ± 80.65 g, respectively. The average BWs at 49 d of age were 1929.14 ± 96.08 g for low and 1615 ± 169.19 g for high FCR individuals (Table 1). During the study period, the food intake (FI) of low and high FCR individuals were 1794.29 ± 228.25 and 2029 ± 269.64 g, respectively. We found that FI and BW35 differences between the two groups were not significant. However, FCR and BWG were significantly different between the lower and higher FCR groups with a difference of 1.4 for FCR and 368 g for BWG. Chickens with low FCR exhibited higher growth rates than their high FCR full-sibs (BWG; *P* < 0.01) with no significant variation in feed intake (*P* > 0.05) (Table 1). This indicated that the energy utilization rate of the low FCR group exceeded the high FCR group.

**Table 1:** Summary of body weight (BW), body weight gain (BWG), food intake (FI), and food conversion ratio (FCR) data collected between 35 and 49 days of age for seven full-sib broiler pairs analyzed in this study

Bird no.	BW35 (g)	BW49 (g)	BWG (g)	FI (g)	FCR	Low/high	Difference	Sire no.	Dam no.
828	860	1976	1110	1910	1.72	Low	1.81	2	1451
1069	870	1415	545	1925	3.53	High			
1001	882	2036	1154	1720	1.49	Low	1.14	7	1502
995	877	1546	669	1790	2.63	High			
999	833	1917	1084	1930	1.78	Low	2.10	8	1512
1003	991	1494	503	1950	3.87	High			
7475	840	1845	1005	2020	2.01	Low	1.73	1	721
7473	940	1500	560	2096	3.74	High			
7549	860	1770	910	1706	1.88	Low	1.27	3	744
7550	1100	1710	610	1916	3.14	High			
7844	1020	2025	1005	1926	1.92	Low	0.81	12	833
7843	920	1875	955	2606	2.73	High			
5154	920	1935	1015	1348	1.33	Low	0.89	20	919
5153	900	1765	865	1920	2.22	High			

Birds are identified by bird cage no., low/high indicates low FCR or high FCR within a pair of birds of the same family. Difference is the difference between high and low FCR of the same family

### Transcriptome sequencing and identification of differentially expressed genes (DEGs)

A total of 71.8 × 10^6^ 150 bp paired-end RNAseq reads were produced from 14 liver samples, including between 2.9 × 10^6^ and 10.8 × 10^6^ reads per sample. For the intestinal tissues 221.0 × 10^6^ reads were produced in total from the duodenal, jejunal, ileal, and cecal samples, including between 2.4 × 10^6^ and 9.0 × 10^6^ per sample. Between 86.5% and 95.0% of sequences from the individual liver samples mapped uniquely to the reference chicken genome Galgal4 (average 90%). For the intestine, 81.7–92.7% (duodenum), 79.8–85.5% (jejunum), 87.3–94.7% (ileum), and 67.4–94.1% (ceca) of reads mapped uniquely (Supplementary Tables 1–5).

By employing the same cut-off (fold-change (FC) > 1.5 and *p*-value ≤ 0.05) for all tissues examined, the global effect of FCR variation on the total number of DEGs per tissue was found to be greatest in the jejunum with a total of 836 genes being differentially expressed (Supplementary Tables 6–10). Approximately 6-fold and 11-fold fewer DEGs were detected in the ceca, duodenum, and liver (142, 77, and 73, respectively), with 15-fold fewer in the ileum (54). Increasing the stringency (FC > 1.5 and corrected *p*-value ≤ 0.05) identified 193 and 14 DEGs in the jejunum and ceca tissues, respectively (Supplementary Tables 6–10). The majority of the jejunum and cecal DEGs (183 and 90, respectively) were up-regulated in the low FCR group. No DEGs were detected in the liver, duodenum, or ileum under the more stringent criteria (Supplementary Tables 6–10). We chose genes for further downstream analysis using a FC > 1.5 and *p*-value ≤ 0.05 as the cut-off.

### Intestinal tissue DEGs associated with FCR variation

**Fig. 1:**
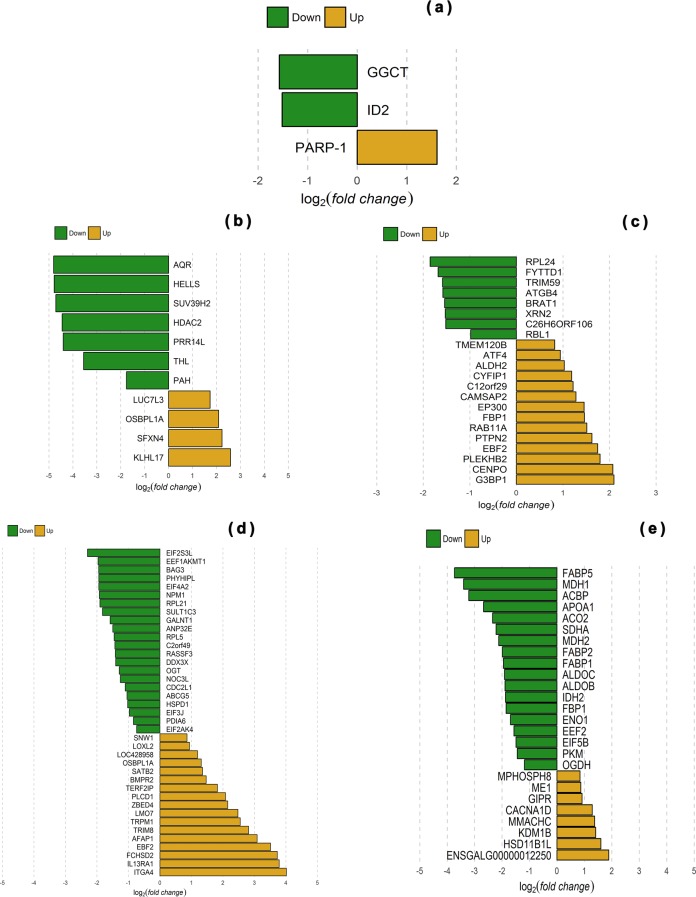
Comparison of gene expression levels and differentially expressed gene distributions between intestinal tissue samples from broilers with extremely low and high feed conversion ratios (FCR). Green represents down-regulated genes with a log_2_ (fold change) > 1.5 and *p*-value < 0.05. Orange represents up-regulated genes with a log_2_ (fold change) < −1.5 and *p*-value < 0.05. Data presented derived from **a** duodenum, **b** jejunum, **c** ileum, **d** ceca, and **e** liver samples

In the duodenum, the genes most up-regulated in low FCR chickens were predominantly associated with physiological processes concerned with circadian feeding behavior and body composition. Notably, inhibitor of DNA binding 2 (ID2), a dominant negative regulator of basic helix-loop-helix (bHLH) transcription factors, was up-regulated in low FCR chickens when compared with their high FCR full-sibs (Fig. 1a). ID2 also acts on circadian clock genes (i.e. CLOCK and BMAL1), participating in several physiological processes through the generation of ~24 h circadian rhythms in gene expression and translating them into rhythms in metabolism and behavior. The gamma-glutamylcyclotransferase (GGCT), which degrades glutathione, the primary intracellular antioxidant, and decreases Notch signaling pathway-mediated processes, acts as an important regulator of body composition and was transcribed at a higher level in low FCR chickens. Conversely, poly(ADP-ribose) polymerase 1 (PARP-1), which has historically been described as a key DNA damage repair enzyme but is also involved in regulation of metabolism and energy expenditure processes and might influence FI behavior, was transcribed at a lower level in low FCR chickens.

Specific to the jejunum, genes up-regulated in low FCR chickens participate in translation processes (including members of the L Ribosomal protein family (44 genes), eukaryotic translation initiation factor 5B (EIF5B), and eukaryotic translation elongation factor 2 (EEF2)), fatty acid transport and metabolism via the PPAR signaling pathway (23 genes including fatty acid binding and transport proteins APOA1, FABP1, FABP2, FABP5, and ACBP), glycolysis and gluconeogenesis (10 genes including FBP1, ALDOB, ALDOC, PKM, and ENO1), and the tricarboxylic acid cycle/citrate cycle (six genes including ACO2, IDH2, MDH1, MDH2, OGDH, and SDHA) (Supplementary Table 11 and Fig. 1b). Genes that were down-regulated in low FCR chickens participate in oxidation–reduction processes, regulation of DNA methylation and the adenylate cyclase-modulating G-protein-coupled receptor signaling pathway (Supplementary Table 11 and Fig. 1b).

Transcription of genes involved in autophagy and protein catabolic processes, which included ubiquitin-specific peptidases (ATG4B, autophagy-related 4B, cysteine peptidase) and ubiquitin-binding proteins (C6orf106, chromosome 6 open-reading frame 106), was up-regulated in the ileum of low FCR chickens. Similarly, genes that were up-regulated in ileal tissue from low FCR chickens also associated with cell proliferation (BRAT1, RBL1, and TRIM59), mRNA export (FYTTD1) and translation (RPL24 and XRN2) processes. A smaller number of genes were down-regulated in ileal tissue from low FCR chickens, including examples involved in mRNA binding (EP300, G3BP1 and CYFIP1), centromere complex formation (CENPO, RAB11A, and CAMSAP2), cell differentiation (C12orf29, EBF2, PLEKHB2, and TMEM120B), and gluconeogenesis (ATF4, ALDH2, FBP1, and PTPN2) (Supplementary Table 12 and Fig. 1c).

The highest number of genes found to be up-regulated in the ceca from low FCR chickens (9 DEGs) were associated with translation processes (Supplementary Table 13 and Fig. 1d). Genes were involved in translation initiation (EIF2S3L, EIF4A2, EIF3J, EIF2AK4, DDX3×), and elongation (EEF1AKMT1), while genes coding for ribosomal subunits (RPL21, RPL5) and involved in their biogenesis (NOC3L) were also highlighted (Supplementary Table 13). Cholesterol homeostasis and fatty acid catabolic processes, which included genes, such as the cholesterol transporter ABCG5 and PHYHIPL, involved in the alpha-oxidation of 3-methyl branched fatty acids, were also up-regulated in low FCR chickens. Several genes from the apoptotic signaling pathway were up-regulated, including those coding for proteins involved in regulation (RASSF3, ANP32E, BAG3, CDC2L1, C2orf49, HSPD1, and NPM1). Finally, genes associated with posttranslational modification, protein folding and sulfur compound metabolic processes were found to be up-regulated. Processes highlighted include O-linked glycosylation (OGT, O-linked N-acetylglucosamine (GlcNAc) transferase and GALNT1, N-acetylgalactosaminyltransferase 1), protein folding (PDIA6, protein disulfide isomerase family A member 6) and sulfur metabolic processes, such as transfer of a sulfonate group (SO_3_^−^) from 3′-phosphoadenosine 5′-phosphosulfate to numerous alcohol, phenol, amine, N-oxide, and N-hydroxy substrates (SULT1C3, sulfotransferase family, cytosolic, 1C, member 3) (Supplementary Table 13). Conversely, several genes that were down-regulated in low FCR chickens were associated with immune responses, including genes which mediate inflammatory responses via the Integrin (ITGA4, FCHSD2, LMO7, and AFAP1), Phopholipase-C (PLCD1, TRPM1, and TRIM8), cytokine–cytokine receptor interactions in Jak-STAT signaling (IL13RA1) and TGF-beta signaling (BMPR2) pathways (Supplementary Table 13). Down-regulated genes were also associated with lipid catabolic process (LOC428958, lysosomal acid lipase/cholesteryl ester hydrolase-like) and lipid transport (OSBPL1A). Transcription regulation processes which includes genes EBF2, LOXL2, ZBED4, SATB2, SNW1, and TERF2IP (Supplementary Table 13), was also identified as down-regulated in low FCR chickens.

### Identification of intestinal epithelial eQTLs associated with FCR

A total of 1656 expression quantitative trait loci (eQTLs) with gene–SNP relationships were identified from the intestinal samples, including 310 eQTLs from the duodenum, 502 from the ileum and 1190 from the ceca (Supplementary Tables 14–16). Jejunum samples were excluded from this analysis since insufficient data coverage to call eQTLs were identified. Association analysis highlighted 66 intestinal eQTLs with 12, 15, and 39 from the duodenum, ileum, and ceca, respectively, showing significant association (*p* < 0.05) to FCR. These eQTLs were defined as the “filtered eQTLs” from the intestinal tissues and were selected for functional analysis and QTL trait enrichment analysis. These filtered eQTLs were associated with eight genes differentially expressed within the duodenum (*EIF2AK2* and *CDKL2*), ileum (*G3BP1* and *ENSGALG00000008738*), and ceca (*PPM1B*, *IGA4*, *ABHD17C*, and *ENSGALG00000001359*) (Supplementary Tables 17–19).

### Liver DEGs associated with FCR variation

In the liver, the majority of the transcripts that were up-regulated in low FCR chickens were associated with transcription regulation (HDAC2, SUV39H2, HELLS, and PRR14L) and mRNA processing (AQR) (Fig. 1e). Two others were involved in the aromatic amino acid metabolic process phenylalanine hydroxylase (PAH), coding for an enzyme involved in production of tyrosine from phenylalanine, and tyrosine hydroxylase-like (THL), which is responsible for the formation of l-dopamine from tyrosine (Supplementary Table 20). Down-regulated transcripts in liver of low FCR chickens were linked to lipid transport, mRNA processing, and protein ubiquitination (Supplementary Table 20). The genes included OSBPL1A (Oxysterol Binding Protein Like 1A) and SFXN4 (Sideroflexin 4), which are involved in lipid and cholesterol homeostasis and their transport, respectively. Down-regulated hepatic genes were also linked to mRNA splice site selection (LUC7L3) and KLHL17, a member of BTB-domain containing proteins family (KLHL17), which has diverse cellular mechanisms, such as control of cytoskeletal organization, ion channel gating, transcription suppression, and protein targeting for ubiquitination through cullin E3 ligases.

### Identification of hepatic eQTLs associated with FCR

A total of 109 eQTLs with gene–SNP relationships were identified from the liver (Supplementary Table 21). Association analysis revealed two eQTLs with significant association (*p* < 0.05) to FCR. These eQTLs were defined as the “filtered eQTLs” from the liver and were selected for functional analysis and QTL trait enrichment analysis. These filtered eQTLs were associated with two genes *SUV39H2* and *EPB41L2* (Supplementary Table 22).

### Genomic location analysis and QTL trait enrichment

Enrichment of the eQTLs for selection effects found 89 QTL traits from Animal Genome Chicken QTLdb to be enriched with genomic overlaps from a total of 57 filtered eQTLs. The top five enriched traits were “Body weight”, “Growth”, “Abdominal fat weight and percentage”, “Carcass weight”, and “Intestine length” (Supplementary Tables 23–26). Grouped by their corresponding QTL categories, the enriched traits contained 73 production traits, 8 health traits, 4 physiology traits, and 2 exterior traits. However, the FCR relevant QTL traits were not enriched. The traits demonstrated significant overlap for 57 filtered eQTLs, associating with the genes *SUV39H2*, *EPB41L2*, *EIF2AK2*, *CDKL2*, *G3BP1*, *ENSGALG00000008738*, *PPM1B*, *ENSSSCG00000024047*, *IGA4*, *ABHD17C*, and *ENSGALG00000001359*.

### Microbiota differed significantly among intestinal locations and between chickens with high or low FCR

A total of 6.44 × 10^6^ sequencing reads (0.032 × 10^6^–0.8 × 10^6^, and 0.14 × 10^6^–4.4 × 10^6^ per sample for 16S rDNA and Metagenomic WGS, respectively) were generated and clustered into tags (Supplementary Table 27). Following OTU selection and chimera checking, all non-unique tags were assigned to 30,048 OTUs, with an average of 884 OTUs per sample (Supplementary Table 28). To evaluate the diversity of bacterial communities in three different intestinal locations, we first performed OTU analysis and compared alpha diversity for the microbiota. The average number of OTUs differed significantly among the three gut locations, with incremental increases in each subsequent gut section: jejunum < ileum (233 vs. 643), ileum < cecum (643 vs. 1462). The Shannon index, which measures species richness and evenness was calculated to evaluate alpha diversity. The jejunum was associated with a significantly lower Shannon index than the ileum and ceca.

Furthermore, we explored the taxonomic distribution of the more abundant bacteria at each intestinal location. Based on the bacterial relative abundance of the top classified phyla, Firmicutes constituted the most prevalent phylotype, comprising 57.0% of the ileum microbial population, followed by Proteobacteria, which represented 3.2%. Proteobacteria accounted for 4.5% and 2.7% of the relative abundances in the jejunum and ceca, respectively. The relative abundance of Bacteroidetes was 0.29%, 1.03%, and 36.12% in the jejunum, ileum, and cecum, respectively. In addition, we also observed Spirochaetes (1.25%) and Tenericutes (1.0%) in the cecum, and Verrucomicrobia (0.01%) in the jejunum (Table 2).

**Table 2:** The relative abundance bacteria at phyla level in jejunum, ileum, and cecum of high and low FCR broilers

Jejunum			Ileum			Cecum		
Phylum	HFCR (mean ± SD)	LFCR (mean ± SD)	Phylum	HFCR (mean ± SD)	LFCR (mean ± SD)	Phylum	HFCR (mean ± SD)	LFCR (mean ± SD)
Firmicutes	50.85 ± 48.03	49.05 ± 42.80	Firmicutes*	77.12 ± 13.46	37.88 ± 23.82*	Firmicutes	49.48 ± 12.91	45.07 ± 12.02
Unclassified bacteria	46.19 ± 45.39	42.25 ± 42.31	Unclassified bacteria*	11.55 ± 12.50	51.72 ± 31.28*	Bacteroidetes	30.61 ± 17.52	41.63 ± 12.11
Proteobacteria	2.75 ± 3.78	6.25 ± 8.90	Proteobacteria	1.55 ± 1.58	4.89 ± 4.06	Unclassified bacteria	13.73 ± 11.88	6.80 ± 3.97
Bacteroidetes	0.05 ± 0.01	0.54 ± 0.56	Actinobacteria	3.10 ± 5.35	0.22 ± 0.25	Proteobacteria	2.99 ± 4.01	2.48 ± 1.17
Actinobacteria	0.02 ± 0.01	0.29 ± 0.51	Bacteroidetes	0.84 ± 1.44	1.22 ± 1.96	Spirochaetes	1.25 ± 2.49	0
Verrucomicrobia	0.01 ± 0.02	0.02 ± 0.03	Tenericutes	0.04 ± 0.05	0.07 ± 0.09	Tenericutes	1.01 ± 0.34	0.72 ± 0.66

^*^Indicates significant difference at *p* < 0.05 (non-parametric Mann–Whitney test)

At the phylum level, the jejunum and ileum microbiota of both groups was dominated by Firmicutes and Unclassified bacteria, with smaller contributions of Proteobacteria, Bacteroidetes, Actinobacteria, and Verrucomicrobia (Table 2). The cecal microbiota of both groups was dominated by Firmicutes and Bacteroidetes, with smaller contribution of Proteobacteria, Spirochaetes, and Tenericutes (Table 1). The relative abundances of microbiota at the phylum level were compared between high and low FCR birds. The jejunum and ileum microbiome of low FCR chickens had a significantly increased relative abundance of the phyla Proteobacteria and Bacteriodetes, but decreased relative abundance of the phyla Firmicutes compared to high FCR. Whereas, the cecal microbiome of low FCR chickens had increased relative abundance of phyla Bacteriodetes, but decreased relative abundance of the phyla Firmicutes compared to high FCR.

**Fig. 2:**
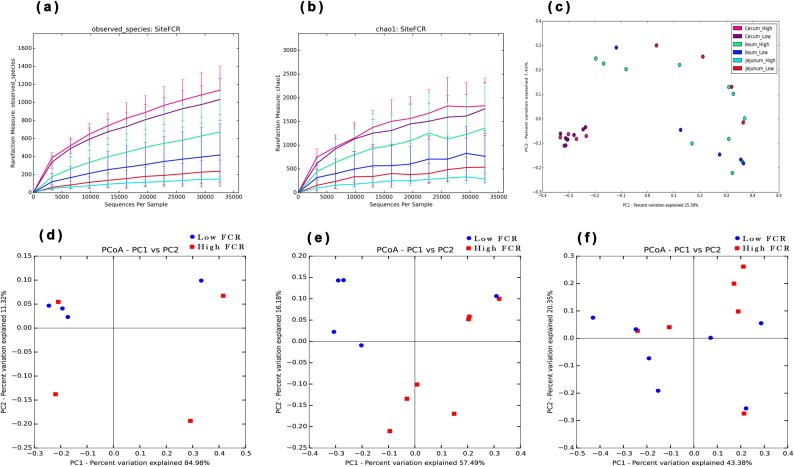
Alpha-diversity and beta-diversity comparisons for the jejunum, ileum, and ceca microbiota from low and high FCR broilers. **a** The number of observed OTUs at the sampling site (mean ± SD). **b** The Chao1 index at the sampling site (mean ± SD). **c** Unweighted UniFrac PCoA of the microbiota across three different intestinal locations. Each color represents a distinct gut location microbiota and low or high FCR. PCoA plots based on unweighted UniFrac distances of the jejunum **d**, ileum **e**, and cecum **f** microbiota of low and high FCR chickens were plotted separately for comparison

We subsequently explored the variations in microbial community composition and the degree of similarity between the samples from the three gut locations at the OTU level. A principal coordinate analysis (PCoA) plot, based on the unweighted Unifrac distance matrices, showed that the gut bacteria composition differed significantly at the different gut locations (Fig. 2c). The microbiota composition in the jejunum samples was significantly different from that of the ileum and ceca (Fig. 2). Gut luminal samples for 16S rRNA gene sequencing were collected from chickens with high and low FCRs. The alpha diversities between the chickens with diverse FCRs in each gut location (jejunum, ileum, and ceca) were then compared. However, the Shannon indices in each gut location were not significantly different between the high and low FCR chickens. A PCoA plot, based on unweighted Unifrac, showed distinct differences in microbial composition in jejunum (Fig. 2d), ileum (Fig. 2e), and cecum (Fig. 2f), when between high and low FCR chickens, however these difference was significant only in ileum of high and low FCR chickens (Adonis, *p* < 0.05; Supplementary Table 29).

**Fig. 3:**
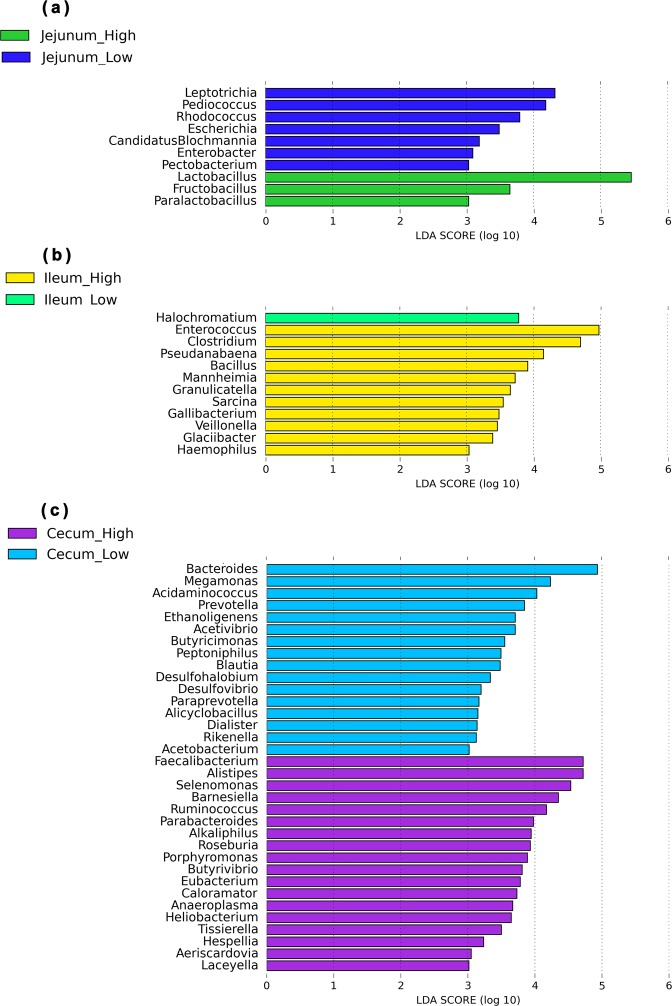
Differential taxonomic profiles of the microbial communities from low and high FCR broilers in three intestinal location. LEfSe identified significantly different bacterial taxa between high and how FCR broilers in the jejunum **a**, ileum **b**, and ceca **c**. The bacterial taxa in this graph were statistically significant based on Kruskal–Wallis test (*p* < 0.05) and had an LDA score ≥ 3

To identify specific bacterial species that were characteristic of the high and low FCR chickens, we performed LEfSe on the taxa with LDA scores greater than three (Fig. 3). The results revealed that 10 bacterial taxa differed significantly in the jejunum, three associated with high FCR, and seven with low FCR (Fig. 3a). In the ileum, 12 bacterial taxa differed significantly, 11 from the high FCR chickens and one from the low FCR (Fig. 3b). In the ceca, 34 bacterial taxa were significantly different, 18 from the high FCR chickens, and 16 from the low FCR (Fig. 3c). In the jejunum, *Lactobacillus*, *Fructobacillus*, and *Paralactobacillus* dominated the LEfSe in the high FCR chickens, while *Leptotrichia*, *Pediococcus*, *Rohdococcus*, and *Escherichia* were dominant in the low FCR chickens (Fig. 3a). In the ileum, *Enterococcus*, *Clostridium*, *Pseudanabaena*, *Bacillus*, *Mannheimia*, and *Granulicatella* were more abundant in the high FCR chickens, while *Halochromatium* was the sole enriched taxa in the low FCR group. In the ceca, *Faecalibacterium*, belonging to the phylum Firmicutes, and *Alistipes*, which belongs to the phylum Bacteriodetes, were more abundant in high FCR chickens, whereas *Bacteroides* and *Megamonas*, belonging to the phyla Bacteriodetes and Firmicutes, respectively, were more abundant in low FCR chickens.

### Microbiota functional capacity differed among three intestinal regions of chickens with different FCR

**Fig. 4:**
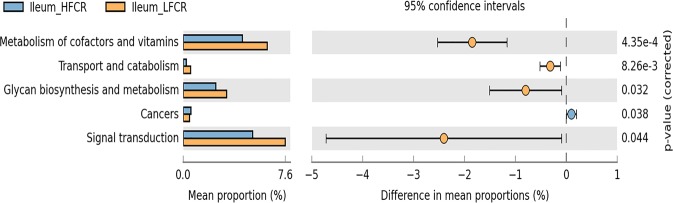
Mean proportion and their differences in functional metagenomics profiles of the ileum microbiome from low and high FCR broilers

**Fig. 5:**
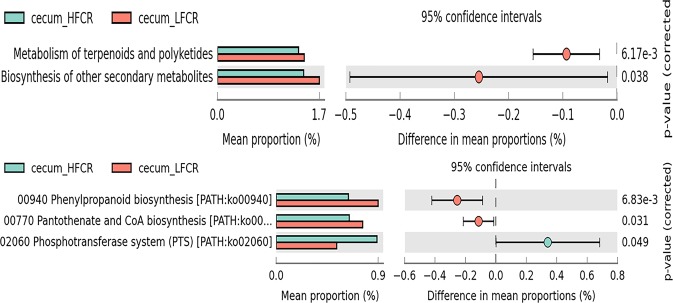
Mean proportion and their differences in functional metagenomics profiles at Level 2 **a** and Level 3 **b** of the ceca microbiome from low and high FCR broilers

Functional annotation was used to further compare the functional capacity of the intestinal microbiome between high and low FCR chickens. In the jejunum, genes related to membrane transport, metabolism of cofactor and vitamins, carbohydrate metabolism, and amino acid metabolism were enriched in the metagenome of high FCR chickens (Supplementary Fig. 2). However, genes associated with lipid metabolism, glycan biosynthesis and metabolism, transcription, nucleotide metabolism, signal transduction and energy metabolism were more abundant in the low FCR chickens. In the ileum, genes related to metabolism of cofactors and vitamins, transport and catabolism, glycan biosynthesis and metabolism, signal transduction (Fig. 4) and glycine, serine and threonine metabolism (Supplementary Fig. 3) had a higher abundance in the low FCR chickens, while genes associated with the cancer category were significantly enriched in the high FCR chickens. The higher abundance of these pathways with a low FCR indicated that the positive activities of some nutrient metabolism pathways may be a contributing factor for high growth. In the ceca, metabolism of terpenoids and polyketides, biosynthesis of other secondary metabolites, phenylpropanoid biosynthesis and, pantothenate and CoA biosynthesis were more highly enriched in the low FCR genes. However, the phosphotransferase system was significantly enriched in the metagenomes of high FCR chickens (Fig. 5).

### Transcriptomic profiling identified transcripts associated with gut microbiota of high and low FCR broilers

**Fig. 6:**
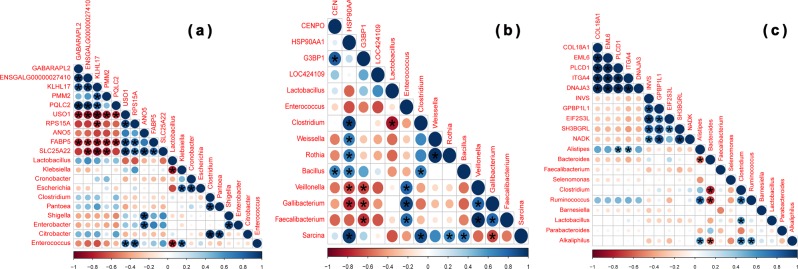
Correlation between bacterial abundance and differentially expressed host genes in specific intestinal compartments. The intensity of the colors and circle size represent the degree of correlation; ‘red’ indicates a negative correlation, ‘blue’ indicates a positive correlation; *p-value ≤ 0.05. **a** Jejunum, **b** ileum, and **c** ceca

**Fig. 7:**
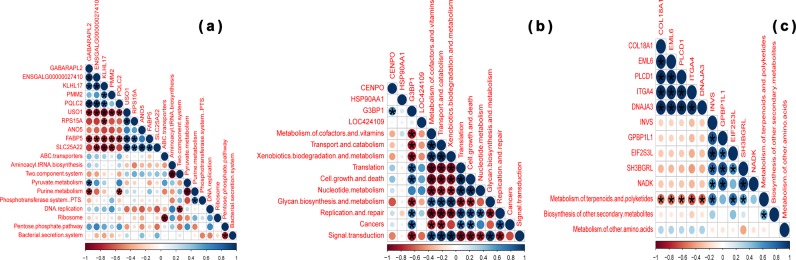
Correlation between functional features of the enteric microbiota and differentially expressed host genes in specific intestinal compartments. The intensity of the colors and circle size represent the degree of correlation; ‘red’ indicates a negative correlation, ‘blue’ indicates a positive correlation; *p-value ≤ 0.05. **a** Jejunum, **b** ileum, and **c** ceca

A Pearson’s correlation analysis was carried out to evaluate the potential link between bacterial abundance and DEGs common to the jejunum, ileum, and ceca of high and low FCR chickens. The abundance of *Shigella*, *Enterobacter*, and *Enterococus* were positively correlated (adjusted *p*-value < 0.0001) with transcription of ANO5, USO1, and RPS15A in the jejunum (Fig. 6a and Supplementary Table 30). *Bacillus* was the only genus positively correlated with transcription of CENPO in the ileum, but did not show significance (adjusted *p*-value = 0.6438) after multiple test corrections (Fig. 6b and Supplementary Table 31). *Clostridium*, *Weissella*, *Rothia*, *Bacillus*, and *Sarcina* were positively correlated but only the abundance of *Clostridium* (adjusted *p*-value = 0.0002) and *Sarcina* (adjusted *p*-value < 0.0001) was found to be significantly correlated, while *Veillonella* (adjusted *p*-value = 0.676) and *Gallibacterium* (adjusted *p*-value = 0.1039) were negatively correlated with transcription of HSP90AA1 in the ileum (Fig. 6b and Supplementary Table 31). Interestingly, *Veillonella* (adjusted *p*-value = 0.5329), *Gallibacterium* (adjusted *p*-value = 0.4211), and *Faecalibacterium* (adjusted *p*-value = 0.013) were also negatively correlated with transcription of G3BP1 (Fig. 6b and Supplementary Table 31). Furthermore, *Alistipes* was positively correlated with PLCD1 (adjusted *p*-value = 0.0117) and ITGA4 (adjusted *p*-value = 0.005) transcription in the ceca (Fig. 6c and Supplementary Table 32). The differences in chicken gene transcription could also be associated with specific microbiota functional features. Purine metabolism was negatively correlated with the transcription of GABARAPL2 (adjusted *p*-value = 0.0002) and PQLC2 (adjusted *p*-value = 0.0003), while pyruvate metabolism was positively correlated with the transcription of GABARAPL2 (adjusted *p*-value < 0.0001) in the jejunum (Fig. 7a and Supplementary Table 33). Only G3BP1 (adjusted *p*-value < 0.0001) transcription in the ileum was positively (“Translation”, “Cell growth and death”, “Replication and repair”, and “Cancers”) and negatively (“Metabolism of cofactors and vitamins”, “Transport and catabolism”, “Xenobiotics biodegradation and metabolism”, “Glycan biosynthesis and metabolism”, and “Signal transduction”) correlated with the various categories of microbiota functional features (Fig. 7b and Supplementary Table 34). The “Metabolism of terpenoids and polyketides” functional feature was positively correlated with the transcription of INVS (adjusted *p*-value < 0.0001), EIF2S3L (adjusted *p*-value < 0.0001), and SH3BGRL (adjusted *p*-value = 0.0037), and negatively correlated with COL18A1 (adjusted *p*-value < 0.0001), EML6, PLCD1 (adjusted *p*-value < 0.0001), ITGA4 (adjusted *p*-value < 0.0001), and DNAJA3 (adjusted *p*-value < 0.0001) in the ceca (Fig. 7c and Supplementary Table 35).

## Discussion

In recent years a major emphasis has been placed on improving feed efficiency in farm animals. FCR and residual feed intake (RFI) are common indices used to access feed efficiency in farm animals. However, most previous studies have focused on single components of these complex traits, defining associations between one or more tissue-specific transcriptomes or gut niche microbiomes on feed efficiency of farm animals.^10,14,19–22^ It is well known that each compartment within the digestive system exhibits a range of overlapping and discrete functions in nutrient digestion and absorption, and that the microbiota composition is similarly distinct.^23^ Studying gene expression dynamics from major digestive organs (e.g. duodenum, jejunum, ileum and ceca, and the liver) in the context of microbiome composition and structure can improve our ability to infer the coevolved functional impact on broiler physiology and, ultimately, feed efficiency.

A global view of variation in gene transcription detected here between high and low FCR performance suggests that FCR can be explained by differences in (1) circadian feeding behavior, (2) immune molecular pathways, (3) lipid metabolism and transport, (4) transcription and translation processes and their regulation, (5) cell division, growth, proliferation, and apoptosis, (6) chromatin methylation and (7) oxidative stress. Thus, genes that are putatively involved in these processes could be considered as candidate genes associated with FCR. Given the complexity of the genes that are involved in these processes, we have only discussed representative genes and their potential role(s) in those functional pathways that could affect host physiology and FCR.

Recently, it has been revealed that metabolism and circadian rhythms are highly intertwined physiological processes and their dysregulation can be associated with various disorders related to metabolism (i.e. diabetes) and appetite.^24^ It has been observed that ID2 knock-out mice present dysregulated circadian rhythms of feeding behavior and locomotor activity, and reduced weight gain regardless of high feed intake.^25^ The down-regulation of ID2 in high FCR chickens might affect the circadian rhythm of feeding behavior. GGCT down-regulates the Notch-signaling pathway involved in development and proper maintenance of adult tissues. Knock-out of this enzyme is lethal in mice but GGCT−/+ mice have reduced body weight, which suggests a role in regulation of body composition.^26^ Furthermore, PARP1 is found to be involved in a pathway which connects feeding behavior to circadian oscillators and might convey these signals through poly(ADP-ribosyl)ation of CLOCK genes.^27^ Therefore, we speculate that the up-regulation of ID2 and GGCT, and down-regulation of PARP-1 in chickens with low FCR may improve feed efficiency with high BWG with similar feed intake and/or reduced energy expenditure compared to chickens with high FCR.

Genes involved in lipid metabolism and cholesterol homeostasis were also found to be associated with FCR.^28,29^ Up-regulation of the fatty acid-binding proteins FABP1, FABP2, and FABP5 in chickens with low FCR may improve the efficient transport of fatty acids from the intestinal lumen, and the subsequent high energy utilization from them, by modulating the PPAR-signaling pathway involved in fatty acid metabolism.

Chickens selected for improved FCR can achieve high BWG with little or no change in feed intake compared to chickens with higher FCR, suggesting that the dynamics of growth (autophagy, cell proliferation, and apoptosis) might be different between high and low FCR individuals. The up-regulated gene, ATG4B, autophagin-1, is a cysteine proteinase and regulates intestinal homeostasis.^30^ BRAT1, which was up-regulated in chickens with a low FCR, has a role in cellular proliferation and metabolism in correlation with mitochondrial functions. Loss of this protein induces mitochondrial malfunctions which suppress cell growth signaling and increase apoptosis.^31^ It is still not clear how low FCR chickens maintain their body weight, but it appears that regulated control of autophagy, cell proliferation, and apoptosis contributes to the maintenance of weight gain.

Activation of the immune system is energetically costly^32^ and long-term stimulation can have a negative host impact. The down-regulation of genes involved in immune response pathways in chickens with low FCR may contribute to greater utilization of absorbed nutrient energy for body growth and composition. However, it is difficult to predict the capability of chickens with low high or high FCR to defend against any immune challenge. In a recent study comparing high and low BWG chickens it was found that growth rate did not influence host responses to a controlled coccidiosis infection when nutrition was not limiting.^33^ These data suggest that optimum or lower expression of genes involved in immune responses might not limit the ability of low FCR chickens to cope with inflammatory events. The eQTL analysis revealed 86 enriched traits which were mainly related to meat and carcass features and production, indicating the likely consequences of selection for low FCR. However, it should be clearly noted that findings from the QTL enrichment are speculative, since no experimental validation has been performed to confirm their associations.

We compared microbiota composition between high and low FCR chickens in three intestinal locations, characterized the functional capacity of the chicken intestinal microbiome and identified potential relationships between the intestinal microbiome and host FCR. The digestion and absorption of starch occurs predominantly in the small intestine, while non-starch polysaccharides are fermented by bacteria to produce short chain fatty acids (SCFA) in the ceca, which serve as important nutrients for the host. In the jejunum of the high FCR chickens most of the dominant OTUs belonged to *Lactobacillus, Fructobacillus*, and *Paralactobacillus*, which have been linked to lactic acid production.^34^ Here, the *Lactobacillus* genus was identified as undesirable for overall performance in terms of FCR. Different strains from the genus *Lactobacillus* have previously been associated with positive or negative influences on farm animal performance.^35–38^ Moreover, different strains of the same *Lactobacillus* species have been associated with weight gain and loss in mice.^39^ Our study suggests that the use of *Lactobacillus* as probiotics must be considered with caution due to a possible indirect effect on FCR.

Focusing on the ileum, *Enterococcus* was found to be significantly enriched in those chickens with a high FCR. Studies in a mouse model have shown that metalloproteases produced by commensal *Enterococcus* strains could contribute to the development of chronic intestinal inflammation by impairing epithelial barrier integrity.^40^ Several *Clostridium* species produce toxins which can cause intestinal damage and inflammation and have been associated with body weight loss in hamsters and mice.^41^ Interestingly, in the functional metagenomics analysis, several subsystems related to cancers were enriched at a low level in the ileum of low FCR chickens. These findings suggest that the potential pathogens and associated inflammation process might contribute to the high FCR noted in these chickens. In this study, the chickens were fed a formula diet that included plant-based fiber-enriched polysaccharide and protein. Therefore, we hypothesize that the cecal microbiome of the low FCR chickens might have had a superior ability to utilize the crude protein or dietary indigestible plant polysaccharides. The most dominant OTUs in the ceca of low FCR chickens belonged to the *Bacteriodes*, *Megamonas*, *Acidaminococcus*, *Prevotella*, and *Paraprevotella*, all of which have a high potential for fermenting various polysaccharides and dietary proteins.^42–46^ In addition to these genera, the ceca from low FCR chickens had a greater abundance of OTUs that represented the genera *Ethanoligenens, Acetivibrio, Butyricimonas, Blautia, Peptoniphilus*, and *Acetobacterium*, all involved in sugar fermentation with the concomitant production of ethanol, acetate, and butyrate as major metabolites.^47–52^ It has been suggested that the fermentation of dietary polysaccharides could produce SCFAs, which can improve the absorptive capacity of the intestine and increase feed efficiency.^53,54^ Thus, the increased abundance of these bacteria could improve cecal and liver health, and as a consequence increase performance.

The capacity of the gut microbiota to change host metabolism and overall energy balance has emerged as a key feature of host–microbe interactions in the intestinal lumen.^55^ In our study, we found a high abundance of *Enterobacter* and *Enterococcus* within the jejunal microbiome of low FCR chickens, which may impact on the efficiency of dietary fat absorption. The abundance of these genera was positively correlated with transcription of USO1, a protein associated with coat protein complex II (COPII) vesicles and, through pre-chylomicron transport vesicles (PCTVs), involved in lipid transport. Similarly, transcription of fatty acid binding and transport proteins increased in the jejunum of low FCR chickens, potentially modulating energy generation and utilization from dietary lipids. In another example, ANO5 transcription was positively correlated with abundance of *Enterobacter* as well as *Shigella*, both *Enterobacteriaceae* associated with human intestinal disease. After membrane injury, ANO5 inclusive vesicles are recruited to the injured cell plasma membrane to repair assaults.^56^ *Shigella* is an intracellular pathogen of humans and chickens^57^ with the ability to invade and reside within cells, indicating a role in membrane disruption.^58,59^ The regulated expression of ANO5 in the presence of these pathogenic microorganisms in chickens may support a healthy intestinal epithelial barrier, encouraging proper growth and efficient energy generation from feed with relevance to physiological performance.

In the ileum we have found that *Clostridium*, *Weissella*, and *Bacillus*, genera identified elsewhere as including strains with probiotic potential, may modulate FCR in chickens by association with regulation of inflammation. It has long been recognized that feed efficiency in animal production is influenced not only by the efficiency of energy utilization but also the inflammatory status of the intestinal mucosa.^37,60–62^ Here, we detected a positive correlation between these genera and heat shock protein HSP90AA1 transcription. Heat shock proteins (HSPs) are involved in many vital cell functions including control of inflammation and oxidation.^63^ The presence of microbial components and metabolites can upregulate HSP expression, indicating that enteric epithelial HSPs may be directly influenced by the composition and metabolic activities of the gut microbiota,^64^ protecting epithelial cells against oxidative stress and inflammation. Many probiotics, especially *Lactobacillus* strains, are able to induce gut epithelial HSPs via different cell receptors and signaling pathways.^65,66^ Further, HSP90AA1 transcription was negatively correlated with abundance of the potentially pathogenic *Gallibacterium* and *Veillonella*. *Gallibacterium* is considered an opportunistic pathogen in chickens. Its effect on poultry production is becoming more pronounced with the emergence of antibiotic-resistant field isolates. Furthermore, the abundance of *Gallibacterium* and *Veillonella*, as well as *Faecalibacterium*, was negatively correlated with transcription of the anti-viral protein, G3BP1. G3BP1 is associated with the Wnt signal transduction pathway.^67^ A study in humans has demonstrated upregulation of Wnt5a via Toll/NF-κB signaling in response to microbial stimulation, illustrating a putative functional involvement in microbial defense and inflammation.^68^ When comparing G3BP1 transcription with microbial pathways, negative correlations were noted for pathways which have been associated with intestinal inflammation in human and/or mouse models.^69–71^ Thus, while these associations are not supported by functional evidence, the involvement of G3BP1 in inflammation and links to the abundance of *Gallibacterium*, *Veillonella*, and *Faecalibacterium* indicate a delicate equilibrium with relevance to host growth and performance.

Microorganisms have the potential to biosynthesize an array of secondary metabolites that can mediate important host–microbe and microbe–microbe interactions,^72^ facilitating actions such as antagonistic competition or quorum sensing. Identifying the biological consequences of these metabolites and the mechanisms by which they mediate interactions may open the door to new bio-therapeutic strategies to treat diseases in animals and humans. Terpenoids and polyketides are examples of secondary metabolites with potential for development as antimicrobial, immunomodulatory, cytotoxic, anti-inflammatory, anti-oxidant, or anti-cancer products.^73^ We found a negative correlation between polyketide and terpenoid metabolism and transcription of ITGA4, PLCD1, DNAJA3, EML6, and COL18A1, and a positive correlation with INVS, EIF2S3L, and SH3BGRL, many of which have been implicated in inflammation via crosstalk between pathways. SH3BGRL has been observed to modulate immune-inflammatory and antioxidant defense in response to bacterial lipopolysaccharides.^74^ DNAJA3 is a regulator of the NF-κB pathway^75,76^ with a central role in innate and adaptive immune responses.^77^ Several studies have highlighted a putative role for Endostatin, acting as an anti-angiogenic factor and influencing inflammation in intestinal pathology.^78–80^ This gradient may point towards a coupling of the host transcriptome and intestinal microbiota, indicating a profound impact on host physiology by maintaining intestinal homeostasis and health. Likewise, the FCR-associated gradient in crosstalk may also indicate functional relevance for the correlated bacterial species. The finding that the occurrence of specific bacterial genera correlated with transcriptome profiles in high FCR chickens supports their potential role in intestinal inflammation.

The work described here identifies genes and components of the enteric microbiota associated with high or low FCR in a broiler chicken line. Differential expression analysis between birds with divergent FCR revealed DE genes in intestinal and liver tissues. Microbiome sequencing identified microbiota that were associated with FCR-relevant traits. While the number of DEGs identified in the intestinal epithelial and liver were low, biological processes related to response to circadian feeding behavior, immune molecular system pathways, lipid metabolism and transport, transcription and translation processes and their regulation, cell proliferation, autophagy and apoptosis were associated with FCR. These results provide new insights into the molecular mechanisms underpinning FCR in chickens, supporting the usefulness of host–microbiome analysis and revealing a convenient use for integrative approaches combining host transcriptome and microbiome data to disentangle the interaction between them in a complex system. Deriving functional proof for these associations will be challenging, but the present findings emphasize that the intestinal microbiota and host intestinal epithelium have to be viewed as a linked entity to permit understanding of FCR trait-specific outcomes. These findings offer value to future breeding strategies, improving feed efficiency traits like FCR.

## Methods

### Ethics statement

All animal experiments in this study were approved by the Institutional Animal Ethics Committee of Anand Agricultural University (Anand, Gujarat, India) with approval number CPCSEA#486, and the experimental procedures were carried out in accordance with the approved guidelines established by this committee.

### Experimental population

A pedigree broiler chicken population was established from Pure-Line Marshall Breed chicks, an indigenous Indian broiler breed developed by Marshall Breeders, Nasik, India, using a genetically restricted flock which has been closed for more than 35 generations. Marshall Breed chickens are farmed across much of India and have been introduced to other countries in Asia and Africa, both regions hosting considerable expansion of poultry production. A total of 30 sires and 300 dams were chosen as the F_0_ generation, 10 dams per sire. Ten chicks were collected from each sire/dam mating to produce 3000 F_1_ progeny. In the absence of identical twins each group of 10 sire/dam-matched chicks were compared as full siblings (sibs). Each independent group of 10 F_1_ chicks was kept in a single enclosure from day of hatch to 35 days of age. A starter diet (2900 kcal ME/kg and 200 g/kg CP) was provided ad libitum during this period. Then, from days 35 to 49, each individual was reared separately and fed a grower diet (3100 kcal ME/kg and 180 g/kg CP). Body weight (BW) was measured at hatching and on days 21, 35, and 49. FCR was calculated as the ratio between feed intake and BWG between days 35 and 49. Full siblings with high and low FCR were selected based upon highly significant (*p* < 0.01) differences in FCR. At 49 days of age chickens were culled and intestinal tissues (duodenum, jejunum, ileum, and ceca), their respective luminal contents and liver were harvested immediately, snap frozen in liquid nitrogen and then stored at −80 °C until further processing.

### RNAseq library preparation and high-throughput sequencing

Total RNA from frozen samples was extracted using TRIzol reagent (Invitrogen) and purified using an RNeasy Mini RNA kit (QIAGEN). Genomic DNA was removed by on-column DNase I treatment according to the manufacturer’s instructions. Total RNA integrity was measured using a RNA Nanochip on a Bioanalyzer 2100 (Agilent Technologies) and samples with a RIN value between 7.5 and 8.0 were retained for use in the study. RNAseq libraries were prepared using a TruSeq Stranded mRNA kit (Illumina, USA) and then subjected to 2 × 150 bp paired-end Illumina MiSeq sequencing as per instructions from the manufacturer.

### RNAseq alignment, differential expression, functional annotation, and SNP calling

Reads were mapped against the reference chicken genome (*galGal4*) and the annotation database Ensembl Genes 84 using the open-source software STAR 2.4.0.2.^81^ The resulting BAM files containing the aligned sequences were subsequently processed with Samtools.^82^ The total number of mapped reads per gene was quantified using HTSeq. 0.9.1^83^ with the GTF file which used in a mapping step for the splice junction-based alignment. Differential expression analysis of contrasting samples from extreme-phenotype individuals was performed using the DESeq2 package.^84^ Genes were considered as DEGs when the rate of change between groups reached |Fold Change (FC)| > 1.5 and differences were significant (*p*-value < 0.05). GO and pathway analysis of DEGs was implemented using DAVID Bioinformatics Resources v6.8 (http://david.abcc.ncifcrf.gov/). *p*-values < 0.05 were deemed to show significant enrichment of DEGs.

The Genome Analysis Toolkit (GATK, version 2.8)^85^ was used to perform Split ‘N’ Trim (ReassignMappingQuality), Indel Realignment and base quality score recalibration to produce a ‘cleaned’ BAM file for each individual. SNP calls were made by the HaplotypeCaller module in GATK using the ‘cleaned’ BAM files in a single batch (14 samples in a batch). The resulting Variant Call Format (VCF, version 4.2) file contained the called variants that overlapped with known SNPs reported in dbSNP v138. The annotated VCF files were then filtered using the GATK variant filter module with a hard filter setting for initial filtering. Variant calls that failed to pass the following filters were eliminated from the call set: QD < 2.0 || FS > 60.0 || MQ < 40.0 || HaplotypeScore > 13.0 || MappingQualityRankSum < −12.5 || ReadPosRankSum < −8.0”. Association analyses were conducted using the simple mixed linear model (MLM) implementation in JMP Genomics 6.1 (SAS, Cary, NC, USA), which can run mixed models to account for fixed and random effects while testing the null hypothesis. False discovery rates were calculated using the Bonferroni correction method.

### 16S rDNA amplicon and metagenomic WGS sequencing

The luminal content collected from each section of the intestinal tract was thawed on ice and ~300 µL used for metagenomic DNA extraction using the QIAamp Fast DNA Stool Mini Kit (QIAGEN, Germany) as per the manufacturer’s instructions. Further, metagenomic DNA was treated with DNase-free RNase (Macherey-Nagel, Germany) to remove any contaminating RNA. The quality and quantity of metagenomic DNA was assessed using agarose gel electrophoresis and a Qubit 3.0 Fluorometer (Invitrogen, Life Technologies, USA), respectively. Metagenomics DNA was stored at −20 °C until further processing. The hypervariable region V3–V4 of the 16S rDNA gene was used to assess microbial diversity between the high and low FCR groups. Amplified amplicons and shotgun metagenomics libraries were made using a Nextera XT DNA library preparation kit (Illumina, USA) according to the manufacturer’s instructions. These libraries were sequenced on an Illumina MiSeq platform using a 2 × 250 sequencing kit.

### Intestinal lumen microbiota analysis

All of the shotgun metagenomic reads generated were submitted to the MG-RAST v 4.0.3 web server for phylogenetic and functional classification of metagenomics WGS data. Low-quality regions were trimmed using SolexaQA with default parameters in MG-RAST.^86^ Artificial duplicate reads were removed using a k-mer-based approach. 16S rDNA amplicon data were analyzed using the QIIME v1.9.0 pipeline.^87^ Operational taxonomic unit (OTU) picking was performed at ≥ 97% sequence similarity and taxonomic identity was assigned by comparing representative sequences against the Greengenes reference database using RDP classifiers. Alpha and beta diversity analyses were performed using several different metrics: observed_OTU and phylogenetic diversity (PD) were used for measurement of alpha diversity within samples, and unweighted and weighted UniFrac PCoA were used for measurement of beta diversity between samples. A non-parametric Kruskal–Wallis (KW) test was used to compare alpha diversity between high and low FCR samples. The LEfSe (linear discriminant analysis coupled with the effect size) algorithm was used to identify those OTUs that differed significantly among the high and low FCR groups for each tissue based on the OTU relative abundance values.^88^ Samples from the jejunum, ileum, and ceca were included. Insufficient duodenal samples passed quality control at the library preparation stage, so the tissue was excluded from this analysis. Briefly, the non-parametric KW sum-rank test was used to detect those taxa which differed significantly in abundance, followed by pairwise Wilcoxon tests to detect biological consistency between the two groups. Finally, an LDA score was used to estimate the effect size of each differentially abundant bacterial taxon. Comparative analysis for taxa and functional features in terms of percentage mean relative frequency was performed using STAMP, where Benjamini–Hochberg FDR was used for multiple test corrections to minimize false discovery rates during multiple group comparative analysis.

### Host transcriptome–microbiome correlation analysis

To obtain a quantitative measure of host transcriptome–microbiome correlation, the Pearson’s correlation was calculated using Hmisc and visualized using the corrplot package for all DEGs and OTUs between the respective gene expression level and OTU abundance. Correlations were considered significant at *p* < 0.05. Multiple test corrections were calculated using Holm–Bonferroni method implemented in Rcmdrmisc package in R.

### Statistical analysis

SNP–Trait association analyses were conducted using the simple MLM implementation in JMP Genomics 6.1 (SAS, Cary, NC, USA), which can run mixed models to account for fixed and random effects while testing the null hypothesis. False discovery rates were calculated using the Bonferroni correction method. Alpha and beta diversity analyses were performed using several different metrics: observed_OTU and PD were used for measurement of alpha diversity within samples, and unweighted and weighted UniFrac PCoA were used for measurement of beta diversity between samples. A non-parametric KW test was used to compare alpha diversity between high and low FCR samples. The LEfSe (linear discriminant analysis coupled with the effect size) algorithm was used to identify those OTUs that differed significantly among the high and low FCR groups for each tissue based on the OTU relative abundance values.^88^ Comparative analysis for taxa and functional features in terms of percentage mean relative frequency was performed using STAMP, where Benjamini–Hochberg FDR was used for multiple test corrections to minimize false discovery rates during multiple group comparative analysis. The Pearson’s correlation was calculated to obtain quantitative measure of host transcriptome–microbiome correlation using Hmisc and visualized using the corrplot package for all DEGs and OTUs between the respective gene expression level and OTU abundance. Correlations were considered significant at *p* < 0.05.

## Supplementary information

Supplementary Information.

ReportingSummary

## Data Availability

All sequencing reads have been deposited in the Sequence Read Archive under BioProject accession number PRJNA503784.

## Supplementary information

**Supplementary information** accompanies the paper on the *npj Biofilms and Microbiomes* website (10.1038/s41522-019-0096-3).

## References

[1] Kc S, Lutz W (2017). The human core of the shared socioeconomic pathways: population scenarios by age, sex and level of education for all countries to 2100. Glob. Environ. Chang..

[2] Grace, D. et al. *Mapping of Poverty and Likely Zoonoses Hotspots*. *Zoonoses Project**4*. Report to the UK Department for International Development (ILRI, Nairobi, Kenya, 2012).

[3] Chengat Prakashbabu B (2017). Eimeria species occurrence varies between geographic regions and poultry production systems and may influence parasite genetic diversity. Vet. Parasitol..

[4] Arthur PF, Archer JA, Herd RM (2004). Feed intake and efficiency in beef cattle: overview of recent Australian research and challenges for the future. Aust. J. Exp. Agric..

[5] Skinner-Noble DO, Teeter RG (2003). Components of feed efficiency in broiler breeding stock: energetics, performance, carcass composition, metabolism, and body temperature. Poult. Sci..

[6] Willems OW, Miller SP, Wood BJ (2013). Aspects of selection for feed efficiency in meat producing poultry. Worlds Poult. Sci. J..

[7] Pakdel A, van Arendonk JA, Vereijken AL, Bovenhuis H (2005). Genetic parameters of ascites-related traits in broilers: correlations with feed efficiency and carcase traits. Br. Poult. Sci..

[8] Aggrey SE, Karnuah AB, Sebastian B, Anthony NB (2010). Genetic properties of feed efficiency parameters in meat-type chickens. Genet. Sel. Evol..

[9] Case LA, Wood BJ, Miller SP (2012). The genetic parameters of feed efficiency and its component traits in the Turkey (*Meleagris gallopavo*). Genet. Sel. Evol..

[10] Xu Z (2016). Combination analysis of genome-wide association and transcriptome sequencing of residual feed intake in quality chickens. BMC Genom..

[11] Pertille F (2017). Genome-wide association study for performance traits in chickens using genotype by sequencing approach. Sci. Rep..

[12] Shah TM (2016). A genome-wide approach to screen for genetic variants in broilers (*Gallus gallus*) with divergent feed conversion ratio. Mol. Genet. Genom..

[13] Gondret F (2017). A transcriptome multi-tissue analysis identifies biological pathways and genes associated with variations in feed efficiency of growing pigs. BMC Genom..

[14] Yi G (2015). In-depth duodenal transcriptome survey in chickens with divergent feed efficiency using RNA-Seq. PLoS ONE.

[15] Chen Y (2011). Global gene expression profiling reveals genes expressed differentially in cattle with high and low residual feed intake. Anim. Genet..

[16] Schokker D (2015). Early life microbial colonization of the gut and intestinal development differ between genetically divergent broiler lines. BMC Genom..

[17] Wang Z, Gerstein M, Snyder M (2009). RNA-Seq: a revolutionary tool for transcriptomics. Nat. Rev. Genet..

[18] Franzosa EA (2015). Sequencing and beyond: integrating molecular ‘omics’ for microbial community profiling. Nat. Rev. Microbiol..

[19] Siegerstetter SC (2017). Intestinal microbiota profiles associated with low and high residual feed intake in chickens across two geographical locations. PLoS ONE.

[20] Mohd Shaufi MA, Sieo CC, Chong CW, Gan HM, Ho YW (2015). Deciphering chicken gut microbial dynamics based on high-throughput 16S rRNA metagenomics analyses. Gut Pathog..

[21] Singh KM (2012). High through put 16S rRNA gene-based pyrosequencing analysis of the fecal microbiota of high FCR and low FCR broiler growers. Mol. Biol. Rep..

[22] Singh KM (2014). Taxonomic and gene-centric metagenomics of the fecal microbiome of low and high feed conversion ratio (FCR) broilers. J. Appl. Genet..

[23] Stanley D, Hughes RJ, Moore RJ (2014). Microbiota of the chicken gastrointestinal tract: influence on health, productivity and disease. Appl. Microbiol. Biotechnol..

[24] Huang W, Ramsey KM, Marcheva B, Bass J (2011). Circadian rhythms, sleep, and metabolism. J. Clin. Invest..

[25] Mathew D (2013). Ablation of the ID2 gene results in altered circadian feeding behavior, and sex-specific enhancement of insulin sensitivity and elevated glucose uptake in skeletal muscle and brown adipose tissue. PLoS ONE.

[26] Crawford RR, Prescott ET, Mungrue IN (2016). Genetic inhibition of Chac1 leads to dysregulation of body composition. FASEB J..

[27] Asher G (2010). Poly(ADP-ribose) polymerase 1 participates in the phase entrainment of circadian clocks to feeding. Cell.

[28] Lee J, Karnuah AB, Rekaya R, Anthony NB, Aggrey SE (2015). Transcriptomic analysis to elucidate the molecular mechanisms that underlie feed efficiency in meat-type chickens. Mol. Genet. Genom..

[29] Karisa B, Moore S, Plastow G (2014). Analysis of biological networks and biological pathways associated with residual feed intake in beef cattle. Anim. Sci. J..

[30] Cabrera S (2013). ATG4B/autophagin-1 regulates intestinal homeostasis and protects mice from experimental colitis. Autophagy.

[31] So EY, Ouchi T (2014). BRAT1 deficiency causes increased glucose metabolism and mitochondrial malfunction. BMC Cancer.

[32] Sheldon BC, Verhulst S (1996). Ecological immunology: costly parasite defences and trade-offs in evolutionary ecology. Trends Ecol. Evol..

[33] Sakkas P (2018). Does selection for growth rate in broilers affect their resistance and tolerance to *Eimeria maxima*?. Vet. Parasitol..

[34] Motato KE (2017). Bacterial diversity of the Colombian fermented milk “Suero Costeno” assessed by culturing and high-throughput sequencing and DGGE analysis of 16S rRNA gene amplicons. Food Microbiol..

[35] Yan W, Sun C, Yuan J, Yang N (2017). Gut metagenomic analysis reveals prominent roles of Lactobacillus and cecal microbiota in chicken feed efficiency. Sci. Rep..

[36] Konsak BM (2013). Identification of differential duodenal gene expression levels and microbiota abundance correlated with differences in energy utilisation in chickens. Anim. Prod. Sci..

[37] Mignon-Grasteau S (2015). Impact of selection for digestive efficiency on microbiota composition in the chicken. PLoS ONE.

[38] Torok VA (2011). Identification and characterization of potential performance-related gut microbiotas in broiler chickens across various feeding trials. Appl. Environ. Microbiol..

[39] Fåk F, Bäckhed F (2012). *Lactobacillus reuteri* prevents diet-induced obesity, but not atherosclerosis, in a strain dependent fashion in Apoe−/− Mice. PLoS ONE.

[40] Steck N (2011). *Enterococcus faecalis* metalloprotease compromises epithelial barrier and contributes to intestinal inflammation. Gastroenterology.

[41] Warn P (2016). Disease progression and resolution in rodent models of *Clostridium difficile* infection and impact of antitoxin antibodies and vancomycin. Antimicrob. Agents Chemother..

[42] Morotomi M, Nagai F, Sakon H, Tanaka R (2009). *Paraprevotella clara* gen. nov., sp. nov. and *Paraprevotella xylaniphila* sp. nov., members of the family ‘Prevotellaceae’ isolated from human faeces. Int. J. Syst. Evol. Microbiol..

[43] Salyers AA, Vercellotti JR, West SE, Wilkins TD (1977). Fermentation of mucin and plant polysaccharides by strains of Bacteroides from the human colon. Appl. Environ. Microbiol..

[44] Zhou W (2018). Simulated digestion and fermentation in vitro by human gut microbiota of polysaccharides from bee collected pollen of Chinese Wolfberry. J. Agric. Food Chem..

[45] Wu GD (2011). Linking long-term dietary patterns with gut microbial enterotypes. Science.

[46] Dai ZL, Zhang J, Wu G, Zhu WY (2010). Utilization of amino acids by bacteria from the pig small intestine. Amino Acids.

[47] Xing D (2006). *Ethanoligenens harbinense* gen. nov., sp. nov., isolated from molasses wastewater. Int. J. Syst. Evol. Microbiol..

[48] Laube VM, Martin SM (1981). Conversion of cellulose to methane and carbon dioxide by triculture of *Acetivibrio cellulolyticus*, *Desulfovibrio* sp., and *Methanosarcina barkeri*. Appl. Environ. Microbiol..

[49] Bernalier A, Willems A, Leclerc M, Rochet V, Collins MD (1996). *Ruminococcus hydrogenotrophicus* sp. nov., a new H2/CO2-utilizing acetogenic bacterium isolated from human feces. Arch. Microbiol..

[50] Amato KR (2013). Habitat degradation impacts black howler monkey (*Alouatta pigra*) gastrointestinal microbiomes. ISME J..

[51] Ezaki T (2001). Proposal of the genera *Anaerococcus* gen. nov., *Peptoniphilus* gen. nov. and *Gallicola* gen. nov. for members of the genus *Peptostreptococcus*. Int. J. Syst. Evol. Microbiol..

[52] Mayer F, Lurz R, Schoberth S (1977). Electron microscopic investigation of the hydrogen-oxidizing acetate-forming anaerobic bacterium *Acetobacterium woodii*. Arch. Microbiol..

[53] Pan D, Yu Z (2014). Intestinal microbiome of poultry and its interaction with host and diet. Gut Microbes.

[54] Pryde SE, Duncan SH, Hold GL, Stewart CS, Flint HJ (2002). The microbiology of butyrate formation in the human colon. FEMS Microbiol. Ecol..

[55] Musso G, Gambino R, Cassader M (2011). Interactions between gut microbiota and host metabolism predisposing to obesity and diabetes. Annu. Rev. Med..

[56] Tian, Y. -M., Wright, J., Cebotaru, L., Wang, H., B Guggino W. Anoctamin5 is related to plasma membrane repair. *JSM Regen. Med. Biomed. Eng.* **3**, 1015 (2015).

[57] Shi R (2014). Pathogenicity of Shigella in chickens. PLoS ONE.

[58] van der Goot FG, Tran van Nhieu G, Allaoui A, Sansonetti P, Lafont F (2004). Rafts can trigger contact-mediated secretion of bacterial effectors via a lipid-based mechanism. J. Biol. Chem..

[59] Mellouk N (2014). Shigella subverts the host recycling compartment to rupture its vacuole. Cell Host Microbe.

[60] Vigors S, O’Doherty JV, Kelly AK, O’Shea CJ, Sweeney T (2016). The effect of divergence in feed efficiency on the intestinal microbiota and the intestinal immune response in both unchallenged and lipopolysaccharide challenged ileal and colonic explants. PLoS ONE.

[61] Kumar S (2018). Effect of antibiotic withdrawal in feed on chicken gut microbial dynamics, immunity, growth performance and prevalence of foodborne pathogens. PLoS ONE.

[62] Korver DR, Klasing KC (1997). Dietary fish oil alters specific and inflammatory immune responses in chicks. J. Nutr..

[63] van Eden W (2015). Diet and the anti-inflammatory effect of heat shock proteins. Endocr. Metab. Immune Disord. Drug Targets.

[64] Arnal ME, Lalles JP (2016). Gut epithelial inducible heat-shock proteins and their modulation by diet and the microbiota. Nutr. Rev..

[65] Fujiya M (2007). The *Bacillus subtilis* quorum-sensing molecule CSF contributes to intestinal homeostasis via OCTN2, a host cell membrane transporter. Cell Host Microbe.

[66] Ueno N (2011). Heat-killed body of lactobacillus brevis SBC8803 ameliorates intestinal injury in a murine model of colitis by enhancing the intestinal barrier function. Inflamm. Bowel Dis..

[67] Bikkavilli RK, Malbon CC (2011). Arginine methylation of G3BP1 in response to Wnt3a regulates beta-catenin mRNA. J. Cell Sci..

[68] Blumenthal A (2006). The Wingless homolog WNT5A and its receptor Frizzled-5 regulate inflammatory responses of human mononuclear cells induced by microbial stimulation. Blood.

[69] Rooks MG (2014). Gut microbiome composition and function in experimental colitis during active disease and treatment-induced remission. ISME J..

[70] Morgan XC (2012). Dysfunction of the intestinal microbiome in inflammatory bowel disease and treatment. Genome Biol..

[71] Tong M (2014). Reprograming of gut microbiome energy metabolism by the FUT2 Crohn’s disease risk polymorphism. ISME J..

[72] Donia MS (2014). A systematic analysis of biosynthetic gene clusters in the human microbiome reveals a common family of antibiotics. Cell.

[73] Salminen A, Lehtonen M, Suuronen T, Kaarniranta K, Huuskonen J (2008). Terpenoids: natural inhibitors of NF-kappaB signaling with anti-inflammatory and anticancer potential. Cell Mol. Life Sci..

[74] Herath TD (2016). Heterogeneous *Porphyromonas gingivalis* LPS modulates immuno-inflammatory response, antioxidant defense and cytoskeletal dynamics in human gingival fibroblasts. Sci. Rep..

[75] Cheng H, Cenciarelli C, Tao M, Parks WP, Cheng-Mayer C (2002). HTLV-1 Tax-associated hTid-1, a human DnaJ protein, is a repressor of Ikappa B kinase beta subunit. J. Biol. Chem..

[76] Momiuchi Y (2015). The role of the phylogenetically conserved cochaperone protein Droj2/DNAJA3 in NF-kappaB signaling. J. Biol. Chem..

[77] Vallabhapurapu S, Karin M (2009). Regulation and function of NF-kappaB transcription factors in the immune system. Annu. Rev. Immunol..

[78] Oikonomou KA (2011). Angiogenin, angiopoietin-1, angiopoietin-2, and endostatin serum levels in inflammatory bowel disease. Inflamm. Bowel Dis..

[79] Deng X (2009). Mesalamine restores angiogenic balance in experimental ulcerative colitis by reducing expression of endostatin and angiostatin: novel molecular mechanism for therapeutic action of mesalamine. J. Pharm. Exp. Ther..

[80] Tolstanova G (2011). Role of anti-angiogenic factor endostatin in the pathogenesis of experimental ulcerative colitis. Life Sci..

[81] Dobin A (2013). STAR: ultrafast universal RNA-seq aligner. Bioinformatics.

[82] Li H (2009). The Sequence Alignment/Map format and SAMtools. Bioinformatics.

[83] Anders S, Pyl PT, Huber W (2015). HTSeq—a Python framework to work with high-throughput sequencing data. Bioinformatics.

[84] Love MI, Huber W, Anders S (2014). Moderated estimation of fold change and dispersion for RNA-seq data with DESeq2. Genome Biol..

[85] McKenna A (2010). The Genome Analysis Toolkit: a MapReduce framework for analyzing next-generation DNA sequencing data. Genome Res..

[86] Cox MP, Peterson DA, Biggs PJ (2010). SolexaQA: at-a-glance quality assessment of Illumina second-generation sequencing data. BMC Bioinforma..

[87] Caporaso JG (2010). QIIME allows analysis of high-throughput community sequencing data. Nat. Methods.

[88] Segata N (2011). Metagenomic biomarker discovery and explanation. Genome Biol..

