# Supplementary material for: Host transcriptome and microbiome interaction modulates physiology of full-sibs broilers with divergent feed conversion ratio
Source: NPJ Biofilms Microbiomes. 2019 Sep 20;5:24. doi: 10.1038/s41522-019-0096-3 (PMC6754422; doi:10.1038/s41522-019-0096-3)
Supplement: Supplementary file 1 — Supplementary Information. [file 41522_2019_96_MOESM1_ESM.pdf]

Supplementary Table 1: Summary of Transcriptome Sequencing data for Duodenum tissue from low and high FCR broilers

Bird ID	Total Number of Reads	Average Input Read Length	Uniquely Mapped Reads	Multi Mapped Reads	Unmapped Reads
828_D	3201945	268	2690948	42269	468728
1069_D	2928453	258	2533338	36870	358245
1001_D	4422985	275	3624859	55838	742288
995_D	5943665	284	5510735	77303	355627
999_D	5837147	284	5382567	76508	378072
1003_D	5095998	286	4670060	67694	358244
7475_D	4761665	285	4380353	62068	317603
7473_D	5723071	283	5218591	78950	423507
7549_D	9098292	294	7639139	119055	1338359
7550_D	7504840	293	6267003	97095	1138484
7844_D	8425592	294	7249917	119736	1055939
7843_D	6109338	293	4995623	81255	1032460
5154_D	3530087	271	3110638	45101	374348
5153_D	3108279	271	2750615	36742	320922

Supplementary Table 2: Summary of Transcriptome Sequencing data for Jejunum tissue from low and high FCR broilers

Bird ID	Total Number of Reads	Average Input Read Length	Uniquely Mapped Reads	Multi Mapped Reads	Unmapped Reads
7475_J	5738659	278	4906128	73264	756929
7473_J	3951243	273	3310312	46669	592687
7549_J	5263589	275	4330546	71453	860597
7550_J	4314083	275	3444856	50076	871519
7844_J	4763576	277	4089996	63053	608785
7843_J	4635737	279	3882546	62312	689334
5154_J	4401613	279	3692159	58168	649678
5153_J	4834646	271	4115392	63472	654127

Supplementary Table 3: Summary of Transcriptome Sequencing data for Ileum tissue from low and high FCR broilers

Bird ID	Total Number of Reads	Average Input Read Length	Uniquely Mapped Reads	Multi Mapped Reads	Unmapped Reads
828_I	5359428	285	5061688	79099	218641
1069_I	4682630	279	4436283	71265	175082
995_I	4332544	286	4075249	62338	194957
999_I	6576858	283	5979823	95065	501970
1003_I	16633	279	11582	1033	4018
7475_I	5056316	280	4488748	67494	498047
7473_I	4527159	282	4048771	60309	416046
7549_I	4088760	283	3745060	59026	284674
7550_I	4291492	277	3821429	54149	414129
7844_I	3917103	286	3583165	53371	278506
7843_I	4966596	283	4542061	64697	357098
5154_I	4964353	279	4333928	66307	562461
5153_I	2416516	289	2188196	34515	193805

Supplementary Table 4: Summary of Transcriptome Sequencing data for Cecum tissue from low and high FCR broilers

Bird ID	Total Number of Reads	Average Input Read Length	Uniquely Mapped Reads	Multi Mapped Reads	Unmapped Reads
828_C	5150892	279	3594014	376360	1180518
1069_C	5602209	283	5111498	81218	409493
1001_C	3808838	286	3557784	57500	193554
995_C	2850755	275	1922159	120277	808319
999_C	4062070	289	3470832	63321	527917
1003_C	5204687	281	4055547	344132	805008
7475_C	4702853	283	4397905	66441	237494
7473_C	8105834	283	7043869	123719	935413
7549_C	3917943	280	3573112	67499	276999
7550_C	3197534	285	2629807	41141	525675
7844_C	5407080	289	4950441	86292	368763
7843_C	3753258	287	3226229	57379	468407
5154_C	4753070	287	4475809	62591	213888
5153_C	3854690	283	3219280	57496	577047

Supplementary Table 5: Summary of Transcriptome Sequencing data for Liver tissue from low and high FCR broilers

Bird ID	Total Number of Reads	Average Input Read Length	Uniquely Mapped Reads	Multi Mapped Reads	Unmapped Reads
828_L	5772822	278	5304271	92737	375814
1069_L	2928453	258	2533338	36870	358245
1001_L	3444122	280	3276007	15128	152987
995_L	5096808	289	4606602	55721	434485
999_L	2161036	279	1578476	59615	522945
1003_L	3182293	271	2896325	17983	267985
7475_L	6264554	290	5454047	75147	735360
7473_L	3428940	270	3155782	34133	239025
7549_L	4493924	284	4250735	45787	197402
7550_L	9582304	290	8336500	104212	1141592
7844_L	10814452	290	9520463	119315	1174674
7843_L	8167985	290	7146193	87958	933834
5154_L	3681949	271	3358853	36672	286424
5153_L	4982931	288	4735206	46237	201310

Supplementary Table 6: Summary of differentially expressed genes (DEGs) in Duodenum of low and high FCR broilers

gene	log2FoldChange	pvalue	padj
ENSGALG00000011530	1.574	0.008	0.997
ENSGALG00000016403	1.513	0.012	0.997
ENSGALG00000024064	1.477	0.014	0.997
ENSGALG00000017098	1.456	0.005	0.997
ENSGALG00000013064	1.404	0.018	0.997
ENSGALG00000003233	1.382	0.013	0.997
ENSGALG00000008457	1.369	0.016	0.997
ENSGALG00000014979	1.367	0.023	0.997
ENSGALG00000018359	1.362	0.024	0.997
ENSGALG00000010156	1.358	0.023	0.997
ENSGALG00000010612	1.352	0.018	0.997
ENSGALG00000016425	1.343	0.019	0.997
ENSGALG00000003016	1.342	0.020	0.997
ENSGALG00000026854	1.327	0.026	0.997
ENSGALG00000027871	1.316	0.015	0.997
ENSGALG00000016467	1.314	0.019	0.997
ENSGALG00000014829	1.283	0.031	0.997
ENSGALG00000028227	1.271	0.027	0.997
ENSGALG00000017079	1.255	0.036	0.997
ENSGALG00000005703	1.215	0.043	0.997
ENSGALG00000010369	1.210	0.043	0.997
ENSGALG00000004021	1.208	0.037	0.997
ENSGALG00000014643	1.206	0.026	0.997
ENSGALG00000013956	1.206	0.040	0.997
ENSGALG00000008045	1.199	0.045	0.997
ENSGALG00000008595	1.196	0.043	0.997
ENSGALG00000011339	1.195	0.024	0.997
ENSGALG00000026913	1.181	0.048	0.997
ENSGALG00000001161	1.174	0.049	0.997
ENSGALG00000011517	1.142	0.024	0.997
ENSGALG00000009970	1.084	0.032	0.997
ENSGALG00000013647	1.067	0.024	0.997
ENSGALG00000006306	1.051	0.036	0.997
ENSGALG00000020986	1.045	0.030	0.997
ENSGALG00000010529	1.037	0.049	0.997
ENSGALG00000003155	1.034	0.027	0.997
ENSGALG00000000086	1.031	0.035	0.997
ENSGALG00000005535	0.932	0.040	0.997

ENSGALG00000018378	0.882	0.025	0.997
ENSGALG00000005817	0.881	0.040	0.997
ENSGALG00000004687	0.874	0.044	0.997
ENSGALG00000008549	0.823	0.015	0.997
ENSGALG00000012126	-0.663	0.033	0.997
ENSGALG00000010963	-0.787	0.045	0.997
ENSGALG00000001577	-0.843	0.015	0.997
ENSGALG00000000277	-0.993	0.042	0.997
ENSGALG00000004215	-1.009	0.031	0.997
ENSGALG00000004306	-1.012	0.048	0.997
ENSGALG00000010119	-1.079	0.001	0.997
ENSGALG00000016754	-1.087	0.051	0.997
ENSGALG00000005598	-1.097	0.037	0.997
ENSGALG00000013727	-1.106	0.037	0.997
ENSGALG00000004955	-1.128	0.011	0.997
ENSGALG00000019527	-1.135	0.047	0.997
ENSGALG00000008731	-1.146	0.029	0.997
ENSGALG00000010560	-1.161	0.002	0.997
ENSGALG00000004493	-1.161	0.047	0.997
ENSGALG00000016364	-1.173	0.022	0.997
ENSGALG00000028083	-1.182	0.045	0.997
ENSGALG00000001782	-1.185	0.044	0.997
ENSGALG00000019325	-1.196	0.038	0.997
ENSGALG00000011992	-1.207	0.036	0.997
ENSGALG00000000463	-1.210	0.036	0.997
ENSGALG00000009556	-1.217	0.039	0.997
ENSGALG00000003371	-1.219	0.040	0.997
ENSGALG00000003881	-1.220	0.016	0.997
ENSGALG00000009870	-1.231	0.038	0.997
ENSGALG00000005442	-1.245	0.020	0.997
ENSGALG00000012199	-1.265	0.034	0.997
ENSGALG00000020003	-1.295	0.028	0.997
ENSGALG00000003925	-1.299	0.031	0.997
ENSGALG00000000804	-1.319	0.015	0.997
ENSGALG00000016953	-1.338	0.026	0.997
ENSGALG00000015836	-1.358	0.023	0.997
ENSGALG00000004981	-1.393	0.019	0.997
ENSGALG00000026003	-1.421	0.016	0.997
ENSGALG00000009228	-1.611	0.007	0.997

Supplementary Table 7: Summary of differentially expressed genes (DEGs) in Jejunum of low and high FCR broilers

gene	log2FoldChange	pvalue	padj
ENSGALG00000010878	4.177	1.21E-04	8.65E-03
ENSGALG00000006771	4.063	3.64E-05	5.95E-03
ENSGALG00000003748	3.815	7.20E-04	2.13E-02
ENSGALG00000015764	3.732	8.82E-04	2.41E-02
ENSGALG00000014295	3.710	9.30E-04	2.42E-02
ENSGALG00000002773	3.643	1.19E-03	2.80E-02
ENSGALG00000008858	3.636	1.24E-03	2.88E-02
ENSGALG00000005789	3.605	1.37E-03	3.01E-02
ENSGALG00000002050	3.590	1.61E-03	3.40E-02
ENSGALG00000016360	3.585	2.53E-06	1.84E-03
ENSGALG00000008482	3.562	1.66E-03	3.42E-02
ENSGALG00000020679	3.497	2.20E-03	3.94E-02
ENSGALG00000014773	3.463	2.34E-03	4.11E-02
ENSGALG00000021655	3.425	2.70E-03	4.44E-02
ENSGALG00000008256	3.403	2.85E-03	4.55E-02
ENSGALG00000001750	3.401	2.87E-03	4.56E-02
ENSGALG00000010433	3.389	5.97E-04	1.92E-02
ENSGALG00000017299	3.386	1.37E-04	8.89E-03
ENSGALG00000004243	3.371	3.29E-03	5.09E-02
ENSGALG00000021040	3.349	3.47E-03	5.26E-02
ENSGALG00000028774	3.342	3.74E-03	5.50E-02
ENSGALG00000004935	3.325	3.71E-03	NA
ENSGALG00000013575	3.323	2.87E-07	4.29E-04
ENSGALG00000006855	3.300	8.78E-04	2.41E-02
ENSGALG00000008821	3.288	4.47E-03	6.37E-02
ENSGALG00000005749	3.273	3.20E-04	1.33E-02
ENSGALG00000004554	3.262	4.62E-03	NA
ENSGALG00000027864	3.252	5.04E-03	6.69E-02
ENSGALG00000026919	3.251	5.02E-03	6.69E-02
ENSGALG00000006169	3.247	8.88E-05	7.92E-03
ENSGALG00000011812	3.243	1.23E-03	2.88E-02
ENSGALG00000001203	3.229	1.31E-03	2.95E-02
ENSGALG00000014950	3.218	4.74E-04	1.69E-02
ENSGALG00000014833	3.217	3.23E-05	5.95E-03
ENSGALG00000009703	3.214	5.49E-03	NA
ENSGALG00000028992	3.208	7.73E-04	2.27E-02
ENSGALG00000016007	3.191	5.14E-04	1.73E-02
ENSGALG00000002813	3.186	3.22E-05	5.95E-03

ENSGALG00000016093	3.177	6.50E-03	7.59E-02
ENSGALG00000009348	3.166	2.60E-04	1.20E-02
ENSGALG00000011089	3.164	6.35E-03	NA
ENSGALG00000011740	3.162	1.75E-03	3.48E-02
ENSGALG00000002412	3.144	4.23E-04	1.56E-02
ENSGALG00000007611	3.121	5.17E-05	6.73E-03
ENSGALG00000011476	3.119	7.18E-03	NA
ENSGALG00000006073	3.117	7.70E-03	NA
ENSGALG00000021106	3.114	3.78E-05	5.95E-03
ENSGALG00000001465	3.105	1.14E-04	8.65E-03
ENSGALG00000021451	3.103	8.13E-03	8.68E-02
ENSGALG00000010343	3.089	6.27E-04	1.97E-02
ENSGALG00000000122	3.072	5.85E-05	6.93E-03
ENSGALG00000012619	3.067	8.36E-03	NA
ENSGALG00000000794	3.045	8.95E-03	NA
ENSGALG00000027035	3.036	3.62E-05	5.95E-03
ENSGALG00000012456	3.020	9.60E-03	NA
ENSGALG00000007549	3.012	1.02E-02	NA
ENSGALG00000002868	3.012	3.61E-06	1.84E-03
ENSGALG00000008620	3.009	6.04E-05	6.93E-03
ENSGALG00000000680	3.005	1.03E-02	NA
ENSGALG00000000533	2.999	1.02E-02	NA
ENSGALG00000010570	2.995	1.04E-02	NA
ENSGALG00000008146	2.990	1.06E-02	NA
ENSGALG00000012172	2.985	7.82E-06	2.41E-03
ENSGALG00000007701	2.978	3.53E-03	5.31E-02
ENSGALG00000001986	2.971	2.34E-03	4.11E-02
ENSGALG00000028810	2.963	1.17E-02	NA
ENSGALG00000008128	2.961	1.14E-02	NA
ENSGALG00000016304	2.958	1.19E-02	NA
ENSGALG00000004039	2.957	1.19E-02	NA
ENSGALG00000009086	2.953	2.41E-04	1.18E-02
ENSGALG00000027151	2.949	2.15E-03	3.92E-02
ENSGALG00000011304	2.941	1.20E-02	NA
ENSGALG00000018386	2.938	4.31E-06	1.84E-03
ENSGALG00000003893	2.937	1.22E-02	NA
ENSGALG00000014432	2.934	2.05E-04	1.10E-02
ENSGALG00000025721	2.931	1.25E-02	NA
ENSGALG00000001708	2.928	1.26E-02	NA
ENSGALG00000001262	2.926	1.27E-02	NA
ENSGALG00000028026	2.915	1.33E-02	NA

ENSGALG00000005176	2.912	5.02E-03	6.69E-02
ENSGALG00000003920	2.908	1.33E-02	NA
ENSGALG00000006779	2.907	1.32E-02	NA
ENSGALG00000008779	2.907	1.27E-03	2.90E-02
ENSGALG00000003505	2.901	1.35E-02	NA
ENSGALG00000027885	2.895	1.37E-02	NA
ENSGALG00000004693	2.886	4.68E-03	6.54E-02
ENSGALG00000008883	2.886	1.40E-02	NA
ENSGALG00000003213	2.871	3.88E-04	1.45E-02
ENSGALG00000004259	2.866	1.49E-02	NA
ENSGALG00000002379	2.865	1.47E-02	NA
ENSGALG00000005465	2.862	2.88E-03	4.56E-02
ENSGALG00000012379	2.862	1.49E-02	NA
ENSGALG00000006077	2.858	1.54E-02	NA
ENSGALG00000016152	2.856	7.32E-03	8.05E-02
ENSGALG00000003601	2.855	1.52E-02	NA
ENSGALG00000008352	2.840	4.98E-05	6.73E-03
ENSGALG00000014644	2.839	2.29E-04	1.16E-02
ENSGALG00000007315	2.838	1.61E-02	NA
ENSGALG00000011689	2.836	1.62E-02	NA
ENSGALG00000027126	2.836	1.62E-02	NA
ENSGALG00000024405	2.830	6.55E-04	2.04E-02
ENSGALG00000015464	2.828	1.64E-02	NA
ENSGALG00000007526	2.828	1.17E-08	3.49E-05
ENSGALG00000002210	2.826	4.49E-05	6.39E-03
ENSGALG00000027142	2.824	6.75E-05	7.17E-03
ENSGALG00000005902	2.818	6.66E-04	2.05E-02
ENSGALG00000008125	2.815	2.65E-04	1.20E-02
ENSGALG00000016359	2.807	1.78E-02	NA
ENSGALG00000016131	2.804	1.03E-04	8.53E-03
ENSGALG00000007493	2.804	1.73E-02	NA
ENSGALG00000015195	2.803	7.06E-04	2.11E-02
ENSGALG00000002777	2.803	7.20E-03	8.03E-02
ENSGALG00000013990	2.795	6.26E-05	6.93E-03
ENSGALG00000028939	2.794	1.81E-02	NA
ENSGALG00000015706	2.793	1.83E-02	NA
ENSGALG00000001949	2.792	1.47E-04	8.89E-03
ENSGALG00000012425	2.789	1.86E-02	NA
ENSGALG00000008016	2.789	6.99E-03	7.92E-02
ENSGALG00000004899	2.785	9.12E-04	2.41E-02
ENSGALG00000014142	2.783	1.87E-02	NA

ENSGALG00000005907	2.782	3.08E-04	1.32E-02
ENSGALG00000019514	2.781	6.83E-03	7.82E-02
ENSGALG00000006899	2.779	1.92E-02	NA
ENSGALG00000006332	2.777	1.87E-02	NA
ENSGALG00000010411	2.777	7.58E-03	8.24E-02
ENSGALG00000025899	2.775	1.92E-02	NA
ENSGALG00000002832	2.773	4.58E-03	6.46E-02
ENSGALG00000007530	2.771	7.32E-03	8.05E-02
ENSGALG00000008844	2.766	1.92E-02	NA
ENSGALG00000011221	2.760	1.99E-02	NA
ENSGALG00000002771	2.759	8.02E-05	7.55E-03
ENSGALG00000003210	2.752	9.10E-03	9.38E-02
ENSGALG00000000366	2.751	1.64E-03	3.42E-02
ENSGALG00000000162	2.745	1.95E-04	1.10E-02
ENSGALG00000007737	2.742	2.10E-02	NA
ENSGALG00000010638	2.740	8.46E-03	8.88E-02
ENSGALG00000011487	2.737	8.32E-03	8.79E-02
ENSGALG00000012529	2.725	1.71E-03	3.47E-02
ENSGALG00000006506	2.724	2.15E-02	NA
ENSGALG00000028204	2.724	2.19E-02	NA
ENSGALG00000001033	2.720	2.18E-02	NA
ENSGALG00000028906	2.715	2.20E-02	NA
ENSGALG00000006409	2.710	9.31E-03	9.50E-02
ENSGALG00000012488	2.708	1.10E-02	1.03E-01
ENSGALG00000009536	2.705	2.27E-02	NA
ENSGALG00000002064	2.699	1.03E-02	1.00E-01
ENSGALG00000011511	2.698	2.13E-04	1.12E-02
ENSGALG00000012350	2.694	2.34E-02	NA
ENSGALG00000008436	2.694	2.35E-02	NA
ENSGALG00000023936	2.693	2.35E-02	NA
ENSGALG00000026182	2.691	2.36E-02	NA
ENSGALG00000001540	2.686	2.40E-02	NA
ENSGALG00000007924	2.686	2.38E-02	NA
ENSGALG00000001039	2.683	1.37E-03	3.01E-02
ENSGALG00000014370	2.681	1.07E-02	1.02E-01
ENSGALG00000008569	2.679	2.43E-02	NA
ENSGALG00000007114	2.679	1.38E-06	1.37E-03
ENSGALG00000012106	2.675	2.43E-02	NA
ENSGALG00000000329	2.674	1.10E-02	1.03E-01
ENSGALG00000010032	2.666	5.40E-03	6.99E-02
ENSGALG00000013233	2.665	5.46E-03	7.04E-02

ENSGALG00000007418	2.664	5.94E-03	7.32E-02
ENSGALG00000003804	2.657	2.51E-02	NA
ENSGALG00000011663	2.655	2.52E-02	NA
ENSGALG00000016813	2.654	2.53E-02	NA
ENSGALG00000006382	2.649	5.37E-03	6.99E-02
ENSGALG00000002205	2.643	2.60E-02	NA
ENSGALG00000010744	2.632	1.16E-02	1.06E-01
ENSGALG00000015339	2.628	5.03E-03	6.69E-02
ENSGALG00000017302	2.628	4.94E-03	6.69E-02
ENSGALG00000006322	2.628	6.95E-05	7.17E-03
ENSGALG00000010582	2.622	2.77E-02	NA
ENSGALG00000012356	2.622	1.33E-02	1.16E-01
ENSGALG00000005422	2.622	2.00E-03	3.84E-02
ENSGALG00000001238	2.621	2.75E-02	NA
ENSGALG00000005575	2.619	2.79E-02	NA
ENSGALG00000008078	2.616	2.81E-02	NA
ENSGALG00000011650	2.614	2.79E-02	NA
ENSGALG00000014309	2.613	1.53E-03	3.30E-02
ENSGALG00000010482	2.610	2.85E-02	NA
ENSGALG00000012416	2.609	2.81E-02	NA
ENSGALG00000006197	2.609	2.88E-02	NA
ENSGALG00000007331	2.604	1.40E-02	1.19E-01
ENSGALG00000014011	2.604	2.87E-02	NA
ENSGALG00000004424	2.604	2.87E-02	NA
ENSGALG00000014371	2.604	2.87E-02	NA
ENSGALG00000010014	2.602	2.90E-02	NA
ENSGALG00000008840	2.600	5.14E-04	1.73E-02
ENSGALG00000001827	2.598	1.44E-02	1.22E-01
ENSGALG00000011056	2.598	2.94E-02	NA
ENSGALG00000005670	2.595	7.14E-03	8.03E-02
ENSGALG00000024248	2.595	6.00E-03	7.36E-02
ENSGALG00000008600	2.590	3.05E-02	NA
ENSGALG00000010614	2.587	2.97E-02	NA
ENSGALG00000010077	2.585	4.01E-05	6.00E-03
ENSGALG00000002971	2.585	3.00E-02	NA
ENSGALG00000001194	2.580	3.11E-02	NA
ENSGALG00000005983	2.576	3.06E-02	NA
ENSGALG00000003781	2.576	3.09E-02	NA
ENSGALG00000000404	2.576	3.07E-02	NA
ENSGALG00000011290	2.570	4.86E-04	1.71E-02
ENSGALG00000008202	2.570	3.43E-03	5.23E-02

ENSGALG00000002837	2.560	1.61E-03	3.40E-02
ENSGALG00000016330	2.559	3.17E-02	NA
ENSGALG00000016887	2.559	3.17E-02	NA
ENSGALG00000016849	2.552	3.22E-02	NA
ENSGALG00000002891	2.550	1.44E-03	3.12E-02
ENSGALG00000005338	2.550	1.97E-02	1.43E-01
ENSGALG00000006384	2.545	9.71E-03	9.71E-02
ENSGALG00000003113	2.542	3.29E-02	NA
ENSGALG00000015629	2.537	1.59E-02	1.29E-01
ENSGALG00000015668	2.536	3.35E-02	NA
ENSGALG00000009070	2.535	1.80E-02	1.39E-01
ENSGALG00000006988	2.534	1.09E-02	1.02E-01
ENSGALG00000000541	2.534	9.27E-03	9.49E-02
ENSGALG00000014948	2.532	1.71E-02	1.35E-01
ENSGALG00000013499	2.531	1.83E-02	1.39E-01
ENSGALG00000000611	2.530	3.42E-02	NA
ENSGALG00000012873	2.529	3.43E-02	NA
ENSGALG00000012043	2.528	1.66E-02	1.33E-01
ENSGALG00000014154	2.528	3.46E-02	NA
ENSGALG00000006080	2.525	3.46E-02	NA
ENSGALG00000004825	2.524	3.43E-02	NA
ENSGALG00000015713	2.521	3.49E-02	NA
ENSGALG00000006157	2.516	3.54E-02	NA
ENSGALG00000000468	2.514	3.54E-02	NA
ENSGALG00000008346	2.514	3.54E-02	NA
ENSGALG00000016479	2.514	9.20E-03	9.45E-02
ENSGALG00000007220	2.512	8.07E-06	2.41E-03
ENSGALG00000006117	2.510	3.58E-02	NA
ENSGALG00000012057	2.507	6.17E-03	7.46E-02
ENSGALG00000007237	2.505	1.90E-02	1.42E-01
ENSGALG00000016073	2.505	3.58E-02	NA
ENSGALG00000004363	2.505	3.59E-02	NA
ENSGALG00000005059	2.504	8.26E-04	2.33E-02
ENSGALG00000009782	2.503	8.68E-03	9.04E-02
ENSGALG00000001386	2.502	3.62E-02	NA
ENSGALG00000015091	2.502	3.62E-02	NA
ENSGALG00000009385	2.497	4.93E-03	6.69E-02
ENSGALG00000022819	2.497	3.71E-02	NA
ENSGALG00000013507	2.495	1.86E-02	1.40E-01
ENSGALG00000012159	2.493	9.88E-03	9.78E-02
ENSGALG00000006096	2.493	1.94E-02	1.42E-01

ENSGALG00000017436	2.492	3.74E-02	NA
ENSGALG00000001475	2.491	9.45E-03	9.60E-02
ENSGALG00000003717	2.491	2.19E-02	1.52E-01
ENSGALG00000011901	2.489	3.70E-02	NA
ENSGALG00000016243	2.488	3.76E-02	NA
ENSGALG00000005610	2.488	3.76E-02	NA
ENSGALG00000014455	2.488	3.76E-02	NA
ENSGALG00000021658	2.485	3.83E-02	NA
ENSGALG00000014375	2.485	3.79E-02	NA
ENSGALG00000017248	2.485	3.79E-02	NA
ENSGALG00000012061	2.484	6.27E-03	7.50E-02
ENSGALG00000010652	2.483	2.75E-03	4.47E-02
ENSGALG00000027963	2.479	3.83E-02	NA
ENSGALG00000008676	2.477	7.90E-04	2.29E-02
ENSGALG00000002222	2.476	2.09E-02	1.50E-01
ENSGALG00000015455	2.474	6.85E-03	7.82E-02
ENSGALG00000000169	2.469	3.88E-02	NA
ENSGALG00000004831	2.469	1.49E-04	8.89E-03
ENSGALG00000023294	2.468	3.89E-03	5.67E-02
ENSGALG00000028520	2.468	1.96E-02	1.42E-01
ENSGALG00000006439	2.467	3.89E-02	NA
ENSGALG00000009369	2.467	3.89E-02	NA
ENSGALG00000019141	2.467	1.35E-02	1.16E-01
ENSGALG00000020688	2.462	7.15E-03	8.03E-02
ENSGALG00000009071	2.455	2.09E-02	1.50E-01
ENSGALG00000005327	2.455	3.88E-03	5.67E-02
ENSGALG00000008667	2.455	7.24E-06	2.41E-03
ENSGALG00000006192	2.451	1.31E-02	1.15E-01
ENSGALG00000027737	2.450	4.11E-02	NA
ENSGALG00000015906	2.449	4.05E-02	NA
ENSGALG00000006311	2.448	4.06E-02	NA
ENSGALG00000014081	2.448	1.54E-02	1.28E-01
ENSGALG00000027269	2.447	1.45E-04	8.89E-03
ENSGALG00000000603	2.446	4.07E-02	NA
ENSGALG00000010027	2.444	4.12E-02	NA
ENSGALG00000001005	2.443	2.63E-03	4.37E-02
ENSGALG00000010276	2.438	2.21E-03	3.94E-02
ENSGALG00000010203	2.436	5.53E-03	7.09E-02
ENSGALG00000011885	2.434	2.20E-02	1.52E-01
ENSGALG00000007519	2.434	2.40E-02	1.58E-01
ENSGALG00000000304	2.433	2.25E-02	1.54E-01

ENSGALG00000010681	2.433	2.32E-02	1.55E-01
ENSGALG00000008051	2.432	4.25E-02	NA
ENSGALG00000013839	2.432	4.25E-02	NA
ENSGALG00000014140	2.431	2.20E-02	1.52E-01
ENSGALG00000017330	2.431	1.05E-03	2.66E-02
ENSGALG00000011902	2.430	1.16E-02	1.06E-01
ENSGALG00000001405	2.430	2.38E-02	1.58E-01
ENSGALG00000002060	2.427	2.28E-02	1.55E-01
ENSGALG00000014171	2.427	2.36E-02	1.57E-01
ENSGALG00000006794	2.426	4.25E-02	NA
ENSGALG00000004396	2.424	4.28E-02	NA
ENSGALG00000012241	2.423	4.33E-02	NA
ENSGALG00000014860	2.422	2.76E-02	1.71E-01
ENSGALG00000003323	2.421	4.35E-02	NA
ENSGALG00000014194	2.421	4.34E-02	NA
ENSGALG00000011990	2.420	4.35E-02	NA
ENSGALG00000016708	2.419	4.36E-02	NA
ENSGALG00000003987	2.419	4.33E-02	NA
ENSGALG00000015356	2.419	4.33E-02	NA
ENSGALG00000007357	2.416	4.38E-02	NA
ENSGALG00000008506	2.416	4.38E-02	NA
ENSGALG00000000542	2.416	4.40E-02	NA
ENSGALG00000023821	2.412	4.42E-02	NA
ENSGALG00000012229	2.412	1.71E-02	1.35E-01
ENSGALG00000009940	2.410	1.86E-02	1.40E-01
ENSGALG00000003932	2.409	4.44E-02	NA
ENSGALG00000011325	2.409	4.50E-02	NA
ENSGALG00000003539	2.400	2.54E-02	NA
ENSGALG00000015496	2.400	2.40E-02	NA
ENSGALG00000012402	2.397	3.00E-02	1.79E-01
ENSGALG00000014067	2.396	4.55E-02	NA
ENSGALG00000004893	2.396	4.58E-02	NA
ENSGALG00000003344	2.396	1.25E-02	1.12E-01
ENSGALG00000009309	2.395	2.88E-02	1.76E-01
ENSGALG00000020788	2.395	4.56E-02	NA
ENSGALG00000006691	2.391	2.77E-02	1.71E-01
ENSGALG00000004555	2.390	4.60E-02	NA
ENSGALG00000005269	2.388	4.62E-02	NA
ENSGALG00000004871	2.388	3.47E-04	1.35E-02
ENSGALG00000012191	2.386	3.06E-02	1.81E-01
ENSGALG00000022557	2.386	4.67E-02	NA

ENSGALG00000010156	2.384	4.70E-02	NA
ENSGALG00000005551	2.383	8.92E-04	2.41E-02
ENSGALG00000006596	2.380	2.88E-02	1.76E-01
ENSGALG00000008523	2.379	2.60E-02	NA
ENSGALG00000014972	2.378	4.75E-02	NA
ENSGALG00000004537	2.376	2.82E-02	NA
ENSGALG00000021271	2.376	4.77E-02	NA
ENSGALG00000013535	2.375	2.96E-02	1.79E-01
ENSGALG00000028872	2.374	8.08E-05	7.55E-03
ENSGALG00000011806	2.374	3.14E-04	1.32E-02
ENSGALG00000004980	2.373	4.76E-02	NA
ENSGALG00000017611	2.367	4.89E-02	NA
ENSGALG00000009906	2.367	4.86E-02	NA
ENSGALG00000001335	2.366	4.83E-02	NA
ENSGALG00000027609	2.364	4.90E-02	NA
ENSGALG00000000177	2.360	4.91E-02	NA
ENSGALG00000001350	2.358	4.91E-02	NA
ENSGALG00000006418	2.358	4.91E-02	NA
ENSGALG00000003971	2.356	1.27E-03	2.90E-02
ENSGALG00000003285	2.352	3.88E-04	1.45E-02
ENSGALG00000008630	2.351	2.89E-02	NA
ENSGALG00000009104	2.351	5.03E-02	NA
ENSGALG00000002488	2.348	5.05E-02	NA
ENSGALG00000004372	2.346	5.04E-02	NA
ENSGALG00000008701	2.345	1.91E-02	1.42E-01
ENSGALG00000011950	2.344	1.11E-03	2.72E-02
ENSGALG00000007866	2.344	5.06E-02	NA
ENSGALG00000028249	2.339	1.54E-02	1.28E-01
ENSGALG00000003770	2.335	1.42E-03	3.11E-02
ENSGALG00000011020	2.333	1.08E-02	1.02E-01
ENSGALG00000004620	2.333	3.19E-02	1.85E-01
ENSGALG00000010238	2.331	1.75E-02	1.36E-01
ENSGALG00000006520	2.331	3.92E-06	1.84E-03
ENSGALG00000028227	2.329	2.95E-02	NA
ENSGALG00000011409	2.324	3.04E-02	NA
ENSGALG00000000489	2.323	2.74E-05	5.95E-03
ENSGALG00000013045	2.318	3.35E-02	NA
ENSGALG00000012684	2.318	1.05E-02	1.01E-01
ENSGALG00000006974	2.317	3.17E-02	NA
ENSGALG00000012108	2.314	2.48E-03	4.26E-02
ENSGALG00000001965	2.310	5.95E-03	7.32E-02

ENSGALG00000006538	2.309	3.24E-02	NA
ENSGALG00000007462	2.308	5.01E-03	6.69E-02
ENSGALG00000002696	2.306	3.47E-04	1.35E-02
ENSGALG00000016554	2.303	9.89E-04	2.55E-02
ENSGALG00000005081	2.302	3.38E-02	NA
ENSGALG00000012052	2.300	3.37E-02	NA
ENSGALG00000017321	2.297	3.30E-02	NA
ENSGALG00000000490	2.295	3.32E-02	NA
ENSGALG00000001452	2.295	5.36E-03	6.99E-02
ENSGALG00000012112	2.290	5.60E-03	7.14E-02
ENSGALG00000008906	2.288	3.31E-02	NA
ENSGALG00000008216	2.287	1.25E-02	1.12E-01
ENSGALG00000003248	2.286	2.02E-02	1.45E-01
ENSGALG00000006767	2.285	3.44E-02	NA
ENSGALG00000011822	2.280	3.82E-02	NA
ENSGALG00000012169	2.280	1.28E-02	1.13E-01
ENSGALG00000016232	2.279	2.50E-03	4.26E-02
ENSGALG00000016567	2.271	3.85E-02	NA
ENSGALG00000008319	2.269	3.92E-02	NA
ENSGALG00000000616	2.267	1.11E-03	2.72E-02
ENSGALG00000011229	2.265	1.98E-04	1.10E-02
ENSGALG00000016704	2.261	2.27E-02	1.54E-01
ENSGALG00000002157	2.258	1.68E-02	1.33E-01
ENSGALG00000011553	2.258	7.93E-03	8.56E-02
ENSGALG00000004390	2.258	3.85E-02	NA
ENSGALG00000002286	2.258	2.57E-03	4.32E-02
ENSGALG00000003766	2.254	4.01E-02	NA
ENSGALG00000005352	2.253	3.62E-03	5.41E-02
ENSGALG00000005108	2.246	1.49E-02	1.25E-01
ENSGALG00000009179	2.244	1.22E-02	1.10E-01
ENSGALG00000028982	2.240	1.89E-02	1.41E-01
ENSGALG00000016325	2.240	2.58E-02	1.65E-01
ENSGALG00000002095	2.236	3.26E-03	5.08E-02
ENSGALG00000028600	2.233	4.48E-04	1.62E-02
ENSGALG00000005002	2.231	1.23E-02	1.10E-01
ENSGALG00000004901	2.224	2.78E-03	4.49E-02
ENSGALG00000014589	2.224	4.43E-02	NA
ENSGALG00000010746	2.224	4.60E-03	6.46E-02
ENSGALG00000007380	2.222	4.09E-02	NA
ENSGALG00000003153	2.219	4.62E-02	NA
ENSGALG00000009733	2.217	2.69E-02	1.69E-01

ENSGALG00000013167	2.217	1.83E-02	1.39E-01
ENSGALG00000019527	2.216	1.64E-02	1.32E-01
ENSGALG00000008073	2.212	1.00E-02	9.79E-02
ENSGALG00000009001	2.210	2.32E-02	1.55E-01
ENSGALG00000011660	2.210	4.22E-02	NA
ENSGALG00000007971	2.209	2.16E-02	1.52E-01
ENSGALG00000011036	2.207	1.16E-03	2.78E-02
ENSGALG00000008348	2.205	1.28E-03	2.90E-02
ENSGALG00000013921	2.203	4.17E-02	NA
ENSGALG00000008291	2.201	2.75E-02	1.70E-01
ENSGALG00000015109	2.200	5.12E-03	6.77E-02
ENSGALG00000014089	2.199	2.42E-02	1.59E-01
ENSGALG00000008480	2.198	1.54E-02	1.28E-01
ENSGALG00000016236	2.188	2.48E-02	1.61E-01
ENSGALG00000005922	2.186	1.27E-04	8.65E-03
ENSGALG00000000812	2.186	1.06E-02	1.01E-01
ENSGALG00000014585	2.184	4.97E-02	NA
ENSGALG00000016791	2.184	4.62E-02	NA
ENSGALG00000015995	2.183	4.42E-02	NA
ENSGALG00000008605	2.182	1.40E-02	1.19E-01
ENSGALG00000006099	2.180	4.71E-02	NA
ENSGALG00000026102	2.178	9.01E-03	9.32E-02
ENSGALG00000018512	2.177	2.96E-02	1.79E-01
ENSGALG00000002159	2.175	5.68E-03	7.14E-02
ENSGALG00000006319	2.174	3.04E-02	1.81E-01
ENSGALG00000008229	2.174	4.67E-02	NA
ENSGALG00000015169	2.171	1.66E-02	1.33E-01
ENSGALG00000028567	2.170	1.78E-04	1.04E-02
ENSGALG00000017380	2.165	1.07E-02	1.02E-01
ENSGALG00000016739	2.161	2.73E-02	1.70E-01
ENSGALG00000003046	2.160	2.11E-02	1.50E-01
ENSGALG00000013879	2.160	4.76E-02	NA
ENSGALG00000011306	2.158	1.58E-03	3.37E-02
ENSGALG00000010235	2.158	1.57E-02	1.28E-01
ENSGALG00000005948	2.157	1.15E-03	2.78E-02
ENSGALG00000005478	2.156	3.01E-02	1.80E-01
ENSGALG00000005398	2.154	5.04E-02	NA
ENSGALG00000002197	2.153	2.01E-04	1.10E-02
ENSGALG00000007037	2.152	3.40E-02	1.94E-01
ENSGALG00000005490	2.151	1.93E-02	1.42E-01
ENSGALG00000000178	2.147	7.80E-05	7.55E-03

ENSGALG00000005624	2.147	4.97E-02	NA
ENSGALG00000012992	2.146	4.97E-02	NA
ENSGALG00000006703	2.142	2.43E-02	1.59E-01
ENSGALG00000016805	2.139	1.56E-02	1.28E-01
ENSGALG00000010645	2.127	1.79E-02	1.38E-01
ENSGALG00000015844	2.122	1.44E-02	1.22E-01
ENSGALG00000011744	2.121	5.10E-02	NA
ENSGALG00000025842	2.119	3.74E-04	1.43E-02
ENSGALG00000010124	2.117	1.16E-04	8.65E-03
ENSGALG00000002932	2.117	2.93E-03	4.61E-02
ENSGALG00000004509	2.116	9.58E-05	8.19E-03
ENSGALG00000014176	2.111	1.15E-04	8.65E-03
ENSGALG00000005001	2.110	8.42E-03	8.86E-02
ENSGALG00000006753	2.109	2.86E-02	1.76E-01
ENSGALG00000007208	2.108	2.44E-04	1.18E-02
ENSGALG00000009312	2.108	4.08E-02	2.15E-01
ENSGALG00000004626	2.104	2.33E-04	1.16E-02
ENSGALG00000028580	2.097	2.23E-02	1.54E-01
ENSGALG00000001634	2.097	5.14E-04	1.73E-02
ENSGALG00000023199	2.096	1.33E-03	2.96E-02
ENSGALG00000012311	2.091	2.38E-02	1.58E-01
ENSGALG00000008684	2.088	6.24E-04	1.97E-02
ENSGALG00000010427	2.087	5.68E-03	7.14E-02
ENSGALG00000008443	2.087	1.69E-03	3.45E-02
ENSGALG00000015702	2.084	4.69E-02	2.31E-01
ENSGALG00000016172	2.078	1.60E-02	1.29E-01
ENSGALG00000009650	2.078	2.54E-02	1.63E-01
ENSGALG00000005930	2.074	3.70E-02	2.02E-01
ENSGALG00000003357	2.073	3.67E-02	2.02E-01
ENSGALG00000006512	2.069	3.37E-04	1.35E-02
ENSGALG00000015709	2.067	3.92E-02	2.09E-01
ENSGALG00000006856	2.060	4.02E-02	2.12E-01
ENSGALG00000009565	2.059	2.10E-02	1.50E-01
ENSGALG00000027501	2.058	1.44E-04	8.89E-03
ENSGALG00000015082	2.053	3.35E-04	1.35E-02
ENSGALG00000027503	2.051	2.05E-03	3.87E-02
ENSGALG00000011951	2.039	8.05E-03	8.65E-02
ENSGALG00000009839	2.039	3.14E-04	1.32E-02
ENSGALG00000015770	2.035	4.57E-03	6.46E-02
ENSGALG00000003651	2.028	4.49E-02	2.27E-01
ENSGALG00000022539	2.016	9.29E-04	2.42E-02

ENSGALG00000023424	2.012	4.78E-03	6.62E-02
ENSGALG00000011921	2.008	4.69E-02	2.31E-01
ENSGALG00000011464	2.006	2.91E-04	1.30E-02
ENSGALG00000004436	2.005	1.01E-02	9.79E-02
ENSGALG00000018384	1.996	9.01E-05	7.92E-03
ENSGALG00000001962	1.994	3.01E-02	1.79E-01
ENSGALG00000000146	1.993	3.19E-02	1.85E-01
ENSGALG00000015780	1.993	5.02E-03	6.69E-02
ENSGALG00000006283	1.991	4.89E-02	2.37E-01
ENSGALG00000008606	1.989	5.10E-02	2.44E-01
ENSGALG00000016392	1.989	4.82E-03	6.64E-02
ENSGALG00000011986	1.989	1.93E-02	1.42E-01
ENSGALG00000001803	1.988	6.37E-03	7.50E-02
ENSGALG00000005425	1.987	1.21E-02	1.09E-01
ENSGALG00000003577	1.987	4.34E-02	2.23E-01
ENSGALG00000004088	1.985	4.73E-02	2.32E-01
ENSGALG00000005937	1.984	2.99E-02	1.79E-01
ENSGALG00000004859	1.983	6.35E-03	7.50E-02
ENSGALG00000004618	1.979	2.26E-02	1.54E-01
ENSGALG00000005282	1.978	4.14E-02	2.16E-01
ENSGALG00000008969	1.977	2.05E-03	3.87E-02
ENSGALG00000000318	1.977	6.35E-03	7.50E-02
ENSGALG00000008399	1.975	4.88E-02	2.37E-01
ENSGALG00000026490	1.975	2.73E-02	1.70E-01
ENSGALG00000018373	1.975	3.54E-05	5.95E-03
ENSGALG00000002670	1.973	1.59E-02	1.29E-01
ENSGALG00000002917	1.971	3.12E-02	1.83E-01
ENSGALG00000001525	1.971	1.00E-02	9.79E-02
ENSGALG00000001973	1.967	3.19E-02	1.85E-01
ENSGALG00000016715	1.967	1.74E-03	3.48E-02
ENSGALG00000001250	1.964	1.15E-03	2.78E-02
ENSGALG00000012296	1.964	3.30E-02	1.90E-01
ENSGALG00000012610	1.963	3.52E-02	1.98E-01
ENSGALG00000015003	1.959	9.00E-04	2.41E-02
ENSGALG00000011258	1.956	3.51E-02	1.98E-01
ENSGALG00000015937	1.949	2.12E-02	1.51E-01
ENSGALG00000016555	1.944	4.32E-02	2.23E-01
ENSGALG00000007711	1.944	1.07E-03	2.69E-02
ENSGALG00000005181	1.944	2.00E-02	1.45E-01
ENSGALG00000008285	1.938	8.30E-03	8.79E-02
ENSGALG00000006300	1.937	1.25E-04	8.65E-03

ENSGALG00000001580	1.936	2.56E-03	4.32E-02
ENSGALG00000025996	1.933	1.75E-02	1.36E-01
ENSGALG00000011433	1.924	2.62E-04	1.20E-02
ENSGALG00000016691	1.922	1.18E-03	2.80E-02
ENSGALG00000005148	1.916	7.99E-04	2.30E-02
ENSGALG00000009511	1.912	3.40E-03	5.22E-02
ENSGALG00000008212	1.911	1.57E-02	1.28E-01
ENSGALG00000008469	1.909	6.12E-03	7.44E-02
ENSGALG00000026077	1.908	6.40E-03	7.50E-02
ENSGALG00000008889	1.901	4.35E-02	2.23E-01
ENSGALG00000003246	1.901	3.13E-02	1.84E-01
ENSGALG00000016775	1.899	3.72E-03	5.50E-02
ENSGALG00000015544	1.895	2.17E-04	1.12E-02
ENSGALG00000016686	1.888	4.87E-02	2.37E-01
ENSGALG00000012266	1.883	4.39E-02	2.24E-01
ENSGALG00000022565	1.878	1.09E-02	1.02E-01
ENSGALG00000018634	1.878	3.72E-02	2.03E-01
ENSGALG00000006694	1.877	9.64E-03	9.71E-02
ENSGALG00000005678	1.873	1.56E-02	1.28E-01
ENSGALG00000006728	1.873	5.22E-04	1.74E-02
ENSGALG00000016142	1.871	1.43E-02	1.21E-01
ENSGALG00000011465	1.869	2.35E-02	1.56E-01
ENSGALG00000016491	1.868	3.39E-03	5.22E-02
ENSGALG00000019751	1.866	2.30E-02	1.55E-01
ENSGALG00000006947	1.865	1.92E-02	1.42E-01
ENSGALG00000005723	1.865	5.01E-02	2.41E-01
ENSGALG00000015637	1.860	1.32E-02	1.15E-01
ENSGALG00000012642	1.855	1.90E-02	1.42E-01
ENSGALG00000012613	1.855	4.40E-02	2.24E-01
ENSGALG00000003693	1.851	1.93E-02	1.42E-01
ENSGALG00000012591	1.851	1.06E-02	1.01E-01
ENSGALG00000002644	1.846	1.95E-02	1.42E-01
ENSGALG00000011857	1.842	1.64E-02	1.31E-01
ENSGALG00000016666	1.841	9.68E-03	9.71E-02
ENSGALG00000005554	1.840	2.86E-02	1.76E-01
ENSGALG00000016088	1.838	4.86E-02	2.37E-01
ENSGALG00000012726	1.836	2.07E-03	3.88E-02
ENSGALG00000025745	1.832	1.94E-03	3.75E-02
ENSGALG00000004357	1.828	4.70E-02	2.31E-01
ENSGALG00000021397	1.828	1.15E-02	1.06E-01
ENSGALG00000004034	1.827	3.57E-02	1.99E-01

ENSGALG00000003521	1.824	2.38E-03	4.11E-02
ENSGALG00000000150	1.822	2.21E-03	3.94E-02
ENSGALG00000002941	1.822	1.13E-02	1.04E-01
ENSGALG00000016829	1.820	2.19E-02	1.52E-01
ENSGALG00000010243	1.818	2.11E-02	1.50E-01
ENSGALG00000003197	1.815	2.53E-04	1.20E-02
ENSGALG00000023934	1.815	8.52E-03	8.90E-02
ENSGALG00000015617	1.814	2.06E-03	3.87E-02
ENSGALG00000000474	1.813	4.09E-02	2.15E-01
ENSGALG00000001937	1.813	3.22E-02	1.87E-01
ENSGALG00000011838	1.811	1.67E-02	1.33E-01
ENSGALG00000016289	1.803	9.69E-03	9.71E-02
ENSGALG00000008590	1.802	3.91E-03	5.68E-02
ENSGALG00000011394	1.799	6.82E-03	7.82E-02
ENSGALG00000003815	1.798	5.79E-03	7.21E-02
ENSGALG00000007814	1.798	1.02E-03	2.62E-02
ENSGALG00000011715	1.798	3.06E-02	1.81E-01
ENSGALG00000008432	1.797	2.67E-02	1.69E-01
ENSGALG00000011472	1.796	3.30E-02	1.90E-01
ENSGALG00000009621	1.795	6.99E-04	2.11E-02
ENSGALG00000015805	1.794	3.87E-02	2.07E-01
ENSGALG00000016910	1.789	4.50E-02	2.27E-01
ENSGALG00000001629	1.788	4.22E-02	2.20E-01
ENSGALG00000006775	1.777	1.35E-02	1.16E-01
ENSGALG00000000141	1.773	6.08E-03	7.42E-02
ENSGALG00000003751	1.768	1.76E-02	1.36E-01
ENSGALG00000008940	1.765	2.12E-03	3.89E-02
ENSGALG00000000427	1.761	3.30E-02	1.90E-01
ENSGALG00000014442	1.761	5.74E-04	1.87E-02
ENSGALG00000002647	1.761	2.96E-02	1.79E-01
ENSGALG00000024372	1.760	1.77E-03	3.48E-02
ENSGALG00000026970	1.758	1.81E-02	1.39E-01
ENSGALG00000019716	1.758	3.26E-03	5.08E-02
ENSGALG00000000521	1.756	3.11E-02	1.83E-01
ENSGALG00000003548	1.755	4.32E-02	2.23E-01
ENSGALG00000015917	1.750	4.50E-04	1.62E-02
ENSGALG00000004553	1.750	2.26E-02	1.54E-01
ENSGALG00000004769	1.748	2.83E-03	4.55E-02
ENSGALG00000004158	1.747	1.22E-02	1.10E-01
ENSGALG00000008082	1.746	3.32E-02	1.91E-01
ENSGALG00000001898	1.744	8.10E-03	8.68E-02

ENSGALG00000008806	1.744	1.00E-02	9.79E-02
ENSGALG00000003532	1.741	1.11E-03	2.72E-02
ENSGALG00000006179	1.735	1.75E-02	1.36E-01
ENSGALG00000020922	1.734	4.90E-02	2.37E-01
ENSGALG00000021139	1.733	3.06E-05	5.95E-03
ENSGALG00000015372	1.732	2.34E-02	1.56E-01
ENSGALG00000006175	1.729	3.99E-02	2.12E-01
ENSGALG00000026978	1.729	1.49E-02	1.25E-01
ENSGALG00000013071	1.717	7.00E-04	2.11E-02
ENSGALG00000013537	1.713	3.97E-02	2.11E-01
ENSGALG00000012790	1.709	2.01E-02	1.45E-01
ENSGALG00000010331	1.698	2.18E-02	1.52E-01
ENSGALG00000004005	1.697	1.83E-02	1.39E-01
ENSGALG00000018370	1.695	1.32E-04	8.78E-03
ENSGALG00000002377	1.692	6.35E-03	7.50E-02
ENSGALG00000010770	1.681	1.86E-02	1.40E-01
ENSGALG00000001334	1.677	3.81E-02	2.05E-01
ENSGALG00000001658	1.676	4.37E-03	6.28E-02
ENSGALG00000005114	1.672	7.43E-03	8.14E-02
ENSGALG00000012866	1.666	3.72E-02	2.03E-01
ENSGALG00000004588	1.660	4.28E-02	2.22E-01
ENSGALG00000014450	1.654	9.86E-03	9.78E-02
ENSGALG00000007659	1.652	1.76E-02	1.36E-01
ENSGALG00000004733	1.652	2.74E-03	4.47E-02
ENSGALG00000000608	1.641	2.06E-04	1.10E-02
ENSGALG00000012135	1.640	3.69E-03	5.48E-02
ENSGALG00000003261	1.637	4.74E-02	2.32E-01
ENSGALG00000009598	1.636	3.49E-02	1.97E-01
ENSGALG00000002478	1.635	4.53E-02	2.28E-01
ENSGALG00000011429	1.630	4.27E-02	2.22E-01
ENSGALG00000003678	1.621	9.87E-03	9.78E-02
ENSGALG00000001328	1.618	3.70E-02	2.02E-01
ENSGALG00000006447	1.615	3.35E-02	1.92E-01
ENSGALG00000006744	1.615	1.27E-04	8.65E-03
ENSGALG00000016841	1.615	1.78E-03	3.48E-02
ENSGALG00000004594	1.614	3.10E-04	1.32E-02
ENSGALG00000000498	1.612	9.94E-03	9.79E-02
ENSGALG00000009008	1.603	9.01E-04	2.41E-02
ENSGALG00000005071	1.602	2.31E-02	1.55E-01
ENSGALG00000016340	1.601	2.19E-02	1.52E-01
ENSGALG00000002363	1.598	4.63E-02	2.31E-01

ENSGALG00000005618	1.597	3.59E-02	1.99E-01
ENSGALG00000001049	1.595	4.38E-02	2.24E-01
ENSGALG00000007840	1.591	3.80E-02	2.05E-01
ENSGALG00000018367	1.591	6.15E-05	6.93E-03
ENSGALG00000002552	1.590	6.89E-03	7.84E-02
ENSGALG00000016894	1.587	2.31E-02	1.55E-01
ENSGALG00000000433	1.586	3.64E-02	2.01E-01
ENSGALG00000007705	1.581	2.69E-03	4.44E-02
ENSGALG00000016720	1.577	2.50E-02	1.62E-01
ENSGALG00000001830	1.566	1.90E-03	3.69E-02
ENSGALG00000027068	1.565	1.44E-02	1.22E-01
ENSGALG00000026572	1.553	5.77E-03	7.21E-02
ENSGALG00000018368	1.551	1.22E-04	8.65E-03
ENSGALG00000016231	1.538	3.60E-02	1.99E-01
ENSGALG00000004521	1.534	2.72E-02	1.70E-01
ENSGALG00000006523	1.529	2.90E-02	1.76E-01
ENSGALG00000015030	1.526	4.01E-02	2.12E-01
ENSGALG00000008595	1.525	3.53E-02	1.98E-01
ENSGALG00000007534	1.523	3.74E-02	2.03E-01
ENSGALG00000001007	1.522	2.41E-02	1.59E-01
ENSGALG00000001482	1.518	4.31E-02	2.23E-01
ENSGALG00000004184	1.515	2.52E-03	4.27E-02
ENSGALG00000010023	1.514	2.25E-02	1.54E-01
ENSGALG00000012173	1.504	3.86E-02	2.07E-01
ENSGALG00000016764	1.495	2.51E-02	1.62E-01
ENSGALG00000011708	1.493	1.78E-03	3.48E-02
ENSGALG00000021039	1.490	3.57E-02	1.99E-01
ENSGALG00000028749	1.472	5.09E-04	1.73E-02
ENSGALG00000003578	1.462	3.57E-02	1.99E-01
ENSGALG00000016572	1.462	2.51E-02	1.62E-01
ENSGALG00000013548	1.453	3.17E-02	1.85E-01
ENSGALG00000001992	1.445	4.07E-02	2.15E-01
ENSGALG00000011190	1.442	4.64E-02	2.31E-01
ENSGALG00000008507	1.439	2.71E-02	1.69E-01
ENSGALG00000008586	1.436	1.34E-02	1.16E-01
ENSGALG00000005974	1.433	4.63E-02	2.31E-01
ENSGALG00000004701	1.425	1.67E-02	1.33E-01
ENSGALG00000010825	1.421	3.67E-02	2.02E-01
ENSGALG00000026383	1.413	5.40E-03	6.99E-02
ENSGALG00000003947	1.409	3.14E-02	1.84E-01
ENSGALG00000008437	1.401	4.69E-02	2.31E-01

ENSGALG00000002160	1.393	1.55E-02	1.28E-01
ENSGALG00000000919	1.389	8.25E-04	2.33E-02
ENSGALG00000010671	1.364	1.52E-02	1.27E-01
ENSGALG00000005587	1.356	1.28E-02	1.13E-01
ENSGALG00000013565	1.355	4.42E-02	2.25E-01
ENSGALG00000009250	1.353	3.53E-02	1.98E-01
ENSGALG00000026592	1.346	2.89E-02	1.76E-01
ENSGALG00000016943	1.340	1.63E-02	1.31E-01
ENSGALG00000028721	1.334	1.13E-02	1.04E-01
ENSGALG00000011330	1.321	4.63E-02	2.31E-01
ENSGALG00000015233	1.304	4.51E-02	2.27E-01
ENSGALG00000004848	1.273	4.78E-03	6.62E-02
ENSGALG00000012533	1.273	1.26E-02	1.12E-01
ENSGALG00000003912	1.207	4.48E-02	2.27E-01
ENSGALG00000016855	1.203	4.80E-02	2.34E-01
ENSGALG00000002447	1.196	2.99E-02	1.79E-01
ENSGALG00000009474	1.195	4.66E-02	2.31E-01
ENSGALG00000014155	1.186	4.76E-02	2.32E-01
ENSGALG00000005823	1.167	2.90E-02	1.76E-01
ENSGALG00000010820	1.146	2.27E-02	1.54E-01
ENSGALG00000005955	1.113	4.27E-02	2.22E-01
ENSGALG00000015483	1.108	3.81E-02	2.05E-01
ENSGALG00000015086	1.092	4.75E-02	2.32E-01
ENSGALG00000007699	1.083	1.95E-02	1.42E-01
ENSGALG00000027497	0.991	3.74E-02	2.03E-01
ENSGALG00000007124	0.991	2.17E-02	1.52E-01
ENSGALG00000004725	0.984	4.94E-02	2.38E-01
ENSGALG00000018378	0.945	2.60E-03	4.34E-02
ENSGALG00000018382	0.922	1.74E-02	1.36E-01
ENSGALG00000002522	0.753	4.44E-02	2.25E-01
ENSGALG00000014684	0.663	3.58E-02	1.99E-01
ENSGALG00000008607	-0.604	3.98E-02	2.12E-01
ENSGALG00000006005	-0.607	4.05E-02	2.14E-01
ENSGALG00000008154	-0.673	2.44E-02	1.59E-01
ENSGALG00000029084	-0.714	2.59E-02	1.65E-01
ENSGALG00000003755	-0.752	1.31E-02	1.15E-01
ENSGALG00000004885	-0.760	1.29E-02	1.13E-01
ENSGALG00000002106	-0.762	3.18E-02	1.85E-01
ENSGALG00000016446	-0.784	3.82E-02	2.05E-01
ENSGALG00000001138	-0.815	7.25E-03	8.04E-02
ENSGALG00000012495	-0.817	4.35E-02	2.23E-01

ENSGALG00000017145	-0.840	2.17E-02	1.52E-01
ENSGALG00000002043	-0.846	1.46E-02	1.23E-01
ENSGALG00000015849	-0.870	3.72E-02	2.03E-01
ENSGALG00000011293	-0.873	2.40E-02	1.58E-01
ENSGALG00000023731	-0.886	3.80E-02	2.05E-01
ENSGALG00000011219	-0.915	2.68E-02	1.69E-01
ENSGALG00000010560	-0.915	3.48E-02	1.97E-01
ENSGALG00000006708	-0.939	7.60E-03	8.24E-02
ENSGALG00000028145	-0.968	6.63E-03	7.68E-02
ENSGALG00000028892	-0.978	3.12E-02	1.83E-01
ENSGALG00000016517	-0.988	5.38E-03	6.99E-02
ENSGALG00000023027	-0.996	6.26E-03	7.50E-02
ENSGALG00000026413	-1.000	2.79E-02	1.72E-01
ENSGALG00000003883	-1.004	4.92E-02	2.38E-01
ENSGALG00000010192	-1.014	3.48E-02	1.97E-01
ENSGALG00000026155	-1.014	1.33E-02	1.16E-01
ENSGALG00000001267	-1.054	2.58E-02	1.65E-01
ENSGALG00000007100	-1.055	3.36E-02	1.92E-01
ENSGALG00000005850	-1.055	2.20E-02	1.52E-01
ENSGALG00000028659	-1.061	8.91E-03	9.25E-02
ENSGALG00000004653	-1.079	2.37E-03	4.11E-02
ENSGALG00000002198	-1.081	3.56E-02	1.99E-01
ENSGALG00000010553	-1.099	5.05E-02	2.42E-01
ENSGALG00000009402	-1.101	1.62E-02	1.31E-01
ENSGALG00000008910	-1.104	9.47E-03	9.60E-02
ENSGALG00000004814	-1.122	1.92E-02	1.42E-01
ENSGALG00000016549	-1.126	3.85E-02	2.07E-01
ENSGALG00000017200	-1.132	5.03E-02	2.42E-01
ENSGALG00000013823	-1.137	4.51E-02	2.27E-01
ENSGALG00000004412	-1.142	2.72E-02	1.70E-01
ENSGALG00000004367	-1.146	4.13E-02	2.16E-01
ENSGALG00000003378	-1.168	4.62E-02	2.31E-01
ENSGALG00000006626	-1.178	4.19E-02	2.19E-01
ENSGALG00000027135	-1.201	3.35E-02	1.92E-01
ENSGALG00000025960	-1.227	1.16E-02	1.06E-01
ENSGALG00000026054	-1.240	3.41E-02	1.94E-01
ENSGALG00000011449	-1.264	1.65E-03	3.42E-02
ENSGALG00000004440	-1.273	2.24E-02	1.54E-01
ENSGALG00000013777	-1.274	3.48E-04	1.35E-02
ENSGALG00000005332	-1.286	4.44E-02	2.25E-01
ENSGALG00000002014	-1.297	3.58E-02	1.99E-01

ENSGALG00000023030	-1.298	2.51E-02	1.62E-01
ENSGALG00000016338	-1.309	4.97E-02	2.39E-01
ENSGALG00000017301	-1.333	1.18E-02	1.06E-01
ENSGALG00000027596	-1.346	1.05E-02	1.01E-01
ENSGALG00000028922	-1.351	2.55E-02	1.64E-01
ENSGALG00000004849	-1.363	2.59E-02	1.65E-01
ENSGALG00000027988	-1.370	1.83E-02	1.39E-01
ENSGALG00000010234	-1.373	2.63E-02	1.67E-01
ENSGALG00000012685	-1.409	4.11E-02	2.16E-01
ENSGALG00000014457	-1.412	2.29E-02	1.55E-01
ENSGALG00000026532	-1.423	4.09E-03	5.91E-02
ENSGALG00000027245	-1.476	3.54E-03	5.31E-02
ENSGALG00000026845	-1.490	2.11E-03	3.89E-02
ENSGALG00000004533	-1.508	5.64E-03	7.14E-02
ENSGALG00000007466	-1.546	4.68E-02	2.31E-01
ENSGALG00000022751	-1.553	6.61E-03	7.68E-02
ENSGALG00000021183	-1.587	1.15E-02	1.06E-01
ENSGALG00000012860	-1.603	2.64E-02	1.67E-01
ENSGALG00000020057	-1.618	2.18E-03	3.94E-02
ENSGALG00000007525	-1.630	2.45E-02	1.59E-01
ENSGALG00000005868	-1.639	2.62E-02	1.66E-01
ENSGALG00000014720	-1.687	2.12E-03	3.89E-02
ENSGALG00000003208	-1.706	5.66E-04	1.86E-02
ENSGALG00000003881	-1.717	7.17E-03	8.03E-02
ENSGALG00000026003	-1.724	5.90E-03	7.32E-02
ENSGALG00000004650	-1.759	3.28E-02	1.90E-01
ENSGALG00000027410	-1.761	2.99E-05	5.95E-03
ENSGALG00000028859	-1.763	6.73E-03	7.77E-02
ENSGALG00000017314	-1.772	1.84E-02	1.40E-01
ENSGALG00000011120	-1.772	4.43E-03	6.34E-02
ENSGALG00000027119	-1.806	2.70E-02	1.69E-01
ENSGALG00000025530	-1.821	5.61E-03	7.14E-02
ENSGALG00000020741	-1.824	1.08E-02	1.02E-01
ENSGALG00000005680	-1.830	1.93E-02	1.42E-01
ENSGALG00000012250	-1.884	4.56E-02	2.29E-01
ENSGALG00000022536	-2.047	4.32E-02	2.23E-01
ENSGALG00000026084	-2.067	2.98E-02	1.79E-01
ENSGALG00000010461	-2.131	1.11E-02	1.03E-01
ENSGALG00000006555	-2.152	3.70E-02	NA
ENSGALG00000023271	-2.152	4.32E-02	NA
ENSGALG00000002199	-2.166	1.77E-03	3.48E-02

ENSGALG00000007326	-2.225	8.50E-04	2.37E-02
ENSGALG00000019356	-2.231	1.81E-02	1.39E-01
ENSGALG00000023723	-2.299	2.35E-03	4.11E-02
ENSGALG00000016522	-2.313	4.13E-02	NA
ENSGALG00000007653	-2.380	3.32E-02	NA
ENSGALG00000018276	-2.458	7.54E-03	8.22E-02
ENSGALG00000026583	-2.477	8.20E-03	8.73E-02
ENSGALG00000025953	-2.481	3.92E-02	NA
ENSGALG00000026688	-2.481	3.92E-02	NA
ENSGALG00000021117	-2.489	3.84E-02	NA
ENSGALG00000003286	-2.583	3.12E-02	NA
ENSGALG00000028428	-2.624	1.06E-02	1.01E-01
ENSGALG00000002623	-2.670	1.39E-02	NA
ENSGALG00000027154	-2.682	6.39E-03	7.50E-02
ENSGALG00000003313	-2.740	7.26E-03	8.04E-02
ENSGALG00000027301	-2.787	1.93E-02	NA
ENSGALG00000028149	-2.845	1.72E-02	NA
ENSGALG00000011970	-2.865	1.61E-02	NA

Supplementary Table 8: Summary of differentially expressed genes (DEGs) in Ileum of low and high FCR broilers

gene	log2FoldChange	pvalue	padj
ENSGALG00000015339	1.8512	0.0147	0.9999
ENSGALG00000007581	1.6827	0.0273	0.9999
ENSGALG00000023571	1.5856	0.0384	0.9999
ENSGALG00000006298	1.5786	0.0372	0.9999
ENSGALG00000026710	1.5460	0.0357	0.9999
ENSGALG00000004348	1.5408	0.0401	0.9999
ENSGALG00000008372	1.5279	0.0461	0.9999
ENSGALG00000002778	1.5171	0.0439	0.9999
ENSGALG00000014170	1.5105	0.0470	0.9999
ENSGALG00000009792	1.4938	0.0432	0.9999
ENSGALG00000025818	1.4933	0.0365	0.9999
ENSGALG00000003155	1.4124	0.0350	0.9999
ENSGALG00000011910	1.3425	0.0185	0.9999
ENSGALG00000001332	0.9816	0.0186	0.9999
ENSGALG00000005469	0.6847	0.0290	0.9999
ENSGALG00000004254	-0.8218	0.0498	0.9999
ENSGALG00000000091	-0.8895	0.0473	0.9999
ENSGALG00000003806	-0.9297	0.0352	0.9999
ENSGALG00000012135	-0.9442	0.0445	0.9999
ENSGALG00000004725	-1.0280	0.0366	0.9999
ENSGALG00000007989	-1.1602	0.0237	0.9999
ENSGALG00000016736	-1.1861	0.0495	0.9999
ENSGALG00000011168	-1.2164	0.0470	0.9999
ENSGALG00000028077	-1.2795	0.0343	0.9999
ENSGALG00000002145	-1.2795	0.0388	0.9999
ENSGALG00000004844	-1.3071	0.0289	0.9999
ENSGALG00000014756	-1.3293	0.0426	0.9999
ENSGALG00000010547	-1.3588	0.0355	0.9999
ENSGALG00000001031	-1.3961	0.0432	0.9999
ENSGALG00000011375	-1.4202	0.0459	0.9999
ENSGALG00000011992	-1.4538	0.0323	0.9999
ENSGALG00000012613	-1.4619	0.0508	0.9999
ENSGALG00000010878	-1.4742	0.0397	0.9999
ENSGALG00000015959	-1.4910	0.0510	0.9999
ENSGALG00000027648	-1.4967	0.0507	0.9999
ENSGALG00000026203	-1.4980	0.0486	0.9999
ENSGALG00000011608	-1.5054	0.0490	0.9999
ENSGALG00000007615	-1.5137	0.0481	0.9999

ENSGALG00000008738	-1.5168	0.0087	0.9999
ENSGALG00000016285	-1.5303	0.0456	0.9999
ENSGALG00000007707	-1.5561	0.0414	0.9999
ENSGALG00000008972	-1.5580	0.0398	0.9999
ENSGALG00000015794	-1.5622	0.0287	0.9999
ENSGALG00000013867	-1.6208	0.0262	0.9999
ENSGALG00000005805	-1.6421	0.0148	0.9999
ENSGALG00000014773	-1.6482	0.0262	0.9999
ENSGALG00000010017	-1.6777	0.0251	0.9999
ENSGALG00000026302	-1.7357	0.0206	0.9999
ENSGALG00000000242	-1.7467	0.0190	0.9999
ENSGALG00000009228	-1.7702	0.0180	0.9999
ENSGALG00000002169	-1.7971	0.0134	0.9999
ENSGALG00000011351	-1.8203	0.0055	0.9999
ENSGALG00000016609	-2.0703	0.0053	0.9999
ENSGALG00000004160	-2.0972	0.0056	0.9999

Supplementary Table 9: Summary of differentially expressed genes (DEGs) in Cecum of low and high FCR broilers

gene	log2FoldChange	pvalue	padj
ENSGALG00000009970	4.020	4.12E-05	7.11E-03
ENSGALG00000010276	2.617	1.75E-03	9.79E-02
ENSGALG00000016340	2.300	2.28E-03	1.15E-01
ENSGALG00000007128	2.232	2.07E-06	2.43E-03
ENSGALG00000001359	1.968	1.78E-03	9.79E-02
ENSGALG00000017133	1.966	1.93E-04	2.59E-02
ENSGALG00000009433	1.946	2.02E-03	1.06E-01
ENSGALG00000001832	1.942	6.55E-04	5.28E-02
ENSGALG00000004108	1.941	2.82E-05	6.43E-03
ENSGALG00000008684	1.940	9.02E-03	2.32E-01
ENSGALG00000002197	1.923	7.16E-03	2.07E-01
ENSGALG00000027035	1.905	5.29E-06	2.43E-03
ENSGALG00000010764	1.852	1.13E-02	2.49E-01
ENSGALG00000011708	1.842	1.17E-03	7.83E-02
ENSGALG00000016805	1.823	4.30E-03	1.77E-01
ENSGALG00000010152	1.795	9.73E-03	2.45E-01
ENSGALG00000016289	1.753	1.30E-03	8.28E-02
ENSGALG00000002661	1.695	7.93E-04	5.99E-02
ENSGALG00000013071	1.622	3.19E-05	6.43E-03
ENSGALG00000005945	1.616	1.72E-03	9.79E-02
ENSGALG00000006520	1.594	3.57E-04	3.59E-02
ENSGALG00000013135	1.580	2.36E-02	3.32E-01
ENSGALG00000005001	1.558	2.92E-02	3.48E-01
ENSGALG00000004179	1.537	1.12E-02	2.49E-01
ENSGALG00000011806	1.535	1.59E-02	2.74E-01
ENSGALG00000013464	1.498	2.70E-02	3.41E-01
ENSGALG00000016720	1.455	4.17E-02	3.87E-01
ENSGALG00000005922	1.454	5.70E-04	4.92E-02
ENSGALG00000016796	1.429	5.25E-03	1.87E-01
ENSGALG00000015105	1.416	5.00E-02	4.29E-01
ENSGALG00000009842	1.416	1.38E-02	2.66E-01
ENSGALG00000001603	1.409	2.70E-02	3.41E-01
ENSGALG00000016231	1.407	1.04E-02	2.49E-01
ENSGALG00000010612	1.350	3.47E-02	3.61E-01
ENSGALG00000012247	1.326	1.80E-02	2.98E-01
ENSGALG00000009300	1.318	1.92E-02	3.03E-01
ENSGALG00000014381	1.316	2.29E-02	3.32E-01
ENSGALG00000005425	1.292	2.52E-02	3.40E-01

ENSGALG00000006524	1.259	1.22E-02	2.59E-01
ENSGALG00000015804	1.254	1.22E-02	2.59E-01
ENSGALG00000003381	1.192	6.98E-03	2.07E-01
ENSGALG00000014210	1.170	5.41E-03	1.87E-01
ENSGALG00000019524	1.160	5.09E-02	4.31E-01
ENSGALG00000016337	1.117	1.50E-02	2.71E-01
ENSGALG00000023648	1.113	3.35E-02	3.61E-01
ENSGALG00000001382	1.102	3.07E-02	3.53E-01
ENSGALG00000009677	1.069	3.41E-02	3.61E-01
ENSGALG00000011834	1.056	1.06E-02	2.49E-01
ENSGALG00000009955	1.045	1.39E-02	2.66E-01
ENSGALG00000008094	1.025	7.70E-03	2.08E-01
ENSGALG00000008266	1.021	2.13E-02	3.21E-01
ENSGALG00000013893	0.978	3.84E-03	1.74E-01
ENSGALG00000007917	0.974	3.94E-02	3.78E-01
ENSGALG00000008187	0.972	2.80E-02	3.42E-01
ENSGALG00000008945	0.950	3.74E-02	3.70E-01
ENSGALG00000010115	0.908	2.22E-02	3.27E-01
ENSGALG00000019232	0.896	3.70E-02	3.70E-01
ENSGALG00000012720	0.890	2.71E-02	3.41E-01
ENSGALG00000007439	0.855	2.71E-04	3.05E-02
ENSGALG00000016447	0.842	2.33E-02	3.32E-01
ENSGALG00000014448	0.831	1.48E-02	2.71E-01
ENSGALG00000019635	0.830	3.68E-02	3.70E-01
ENSGALG00000009828	0.815	1.11E-02	2.49E-01
ENSGALG00000004428	0.815	7.94E-03	2.08E-01
ENSGALG00000000218	0.815	7.64E-03	2.08E-01
ENSGALG00000012077	0.795	1.11E-02	2.49E-01
ENSGALG00000009402	0.774	1.36E-02	2.66E-01
ENSGALG00000008784	0.749	1.73E-02	2.95E-01
ENSGALG00000011387	0.742	2.76E-02	3.42E-01
ENSGALG00000010560	0.738	3.52E-02	3.61E-01
ENSGALG00000008500	0.707	3.50E-02	3.61E-01
ENSGALG00000012968	0.651	4.26E-02	3.92E-01
ENSGALG00000006005	0.635	1.93E-02	3.03E-01
ENSGALG00000014709	0.607	3.30E-02	3.61E-01
ENSGALG00000009611	0.586	2.16E-02	3.22E-01
ENSGALG00000003755	0.345	4.09E-02	3.83E-01
ENSGALG00000004247	-0.513	3.45E-02	3.61E-01
ENSGALG00000009919	-0.654	3.38E-02	3.61E-01
ENSGALG00000012295	-0.669	4.30E-02	3.92E-01

ENSGALG00000012929	-0.676	3.41E-02	3.61E-01
ENSGALG00000001577	-0.828	4.57E-02	4.06E-01
ENSGALG00000010501	-0.857	5.35E-04	4.92E-02
ENSGALG00000000099	-0.890	1.59E-02	2.74E-01
ENSGALG00000000402	-0.935	1.39E-02	2.66E-01
ENSGALG00000003412	-0.938	4.93E-02	4.28E-01
ENSGALG00000003513	-1.015	2.59E-02	3.40E-01
ENSGALG00000006512	-1.037	2.98E-03	1.44E-01
ENSGALG00000014720	-1.063	1.43E-02	2.66E-01
ENSGALG00000012213	-1.106	7.76E-03	2.08E-01
ENSGALG00000028197	-1.107	3.79E-02	3.72E-01
ENSGALG00000026229	-1.118	3.52E-02	3.61E-01
ENSGALG00000023824	-1.193	4.07E-02	3.83E-01
ENSGALG00000016106	-1.289	2.46E-02	3.37E-01
ENSGALG00000015086	-1.308	4.84E-03	1.77E-01
ENSGALG00000021139	-1.311	1.02E-02	2.49E-01
ENSGALG00000008135	-1.353	3.89E-02	3.76E-01
ENSGALG00000007131	-1.400	1.55E-02	2.74E-01
ENSGALG00000008459	-1.469	6.46E-03	2.07E-01
ENSGALG00000006472	-1.595	4.74E-02	4.18E-01
ENSGALG00000007119	-1.678	3.06E-02	3.53E-01
ENSGALG00000018380	-1.690	5.01E-02	4.29E-01
ENSGALG00000008325	-1.746	2.03E-02	3.11E-01
ENSGALG00000018382	-1.783	2.64E-02	3.41E-01
ENSGALG00000000903	-1.825	2.88E-02	3.48E-01
ENSGALG00000026875	-1.830	1.81E-02	2.98E-01
ENSGALG00000004338	-1.860	1.03E-03	7.32E-02
ENSGALG00000010706	-1.892	4.40E-02	3.97E-01
ENSGALG00000005535	-1.901	1.36E-02	2.66E-01
ENSGALG00000004975	-1.919	4.74E-03	1.77E-01
ENSGALG00000013441	-1.946	3.90E-03	1.74E-01
ENSGALG00000005805	-2.080	2.95E-05	6.43E-03
ENSGALG00000008547	-2.152	3.45E-02	3.61E-01
ENSGALG00000016855	-2.166	3.02E-02	3.53E-01
ENSGALG00000008803	-2.188	4.54E-02	4.06E-01
ENSGALG00000015965	-2.253	7.41E-05	1.12E-02
ENSGALG00000008139	-2.300	7.21E-03	2.07E-01
ENSGALG00000008936	-2.335	5.63E-03	1.89E-01
ENSGALG00000002500	-2.382	2.44E-02	3.37E-01
ENSGALG00000027681	-2.387	4.32E-02	3.92E-01
ENSGALG00000016920	-2.474	4.40E-03	1.77E-01

ENSGALG00000006050	-2.526	1.96E-02	3.03E-01
ENSGALG00000003849	-2.549	2.36E-02	3.32E-01
ENSGALG00000027871	-2.639	4.01E-02	3.81E-01
ENSGALG00000011120	-2.649	1.92E-02	3.03E-01
ENSGALG00000006118	-2.753	2.77E-02	3.42E-01
ENSGALG00000007821	-2.811	2.78E-04	3.05E-02
ENSGALG00000008099	-2.813	1.32E-02	2.66E-01
ENSGALG00000011259	-3.025	7.06E-03	2.07E-01
ENSGALG00000015556	-3.084	4.89E-02	4.28E-01
ENSGALG00000000546	-3.107	2.55E-02	3.40E-01
ENSGALG00000010719	-3.133	3.52E-02	3.61E-01
ENSGALG00000003194	-3.205	3.87E-02	3.76E-01
ENSGALG00000000993	-3.221	3.68E-02	3.70E-01
ENSGALG00000028063	-3.353	2.94E-02	3.48E-01
ENSGALG00000015448	-3.364	1.83E-02	2.98E-01
ENSGALG00000006740	-3.436	6.04E-06	2.43E-03
ENSGALG00000006420	-3.476	4.27E-03	1.77E-01
ENSGALG00000000242	-3.509	2.58E-02	3.40E-01
ENSGALG00000017329	-3.726	1.42E-02	2.66E-01
ENSGALG00000006032	-3.786	3.24E-02	3.61E-01
ENSGALG00000008978	-4.014	4.77E-03	1.77E-01
ENSGALG00000002030	-4.073	6.50E-03	2.07E-01

Supplementary Table 10: Summary of differentially expressed genes (DEGs) in Liver of low and high FCR broilers

gene	log2FoldChange	pvalue	padj
ENSGALG00000009838	4.804	0.004	0.999
ENSGALG00000005451	4.780	0.003	0.999
ENSGALG00000013920	4.713	0.006	0.999
ENSGALG00000015014	4.551	0.014	0.999
ENSGALG00000009948	4.523	0.022	0.999
ENSGALG00000014991	4.447	0.008	0.999
ENSGALG00000000231	4.421	0.007	0.999
ENSGALG00000008015	4.407	0.026	0.999
ENSGALG00000028473	4.405	0.004	0.999
ENSGALG00000007260	4.321	0.020	0.999
ENSGALG00000003135	4.239	0.017	0.999
ENSGALG00000003849	4.229	0.014	0.999
ENSGALG00000016329	4.213	0.032	0.999
ENSGALG00000003861	4.113	0.037	0.999
ENSGALG00000010137	4.099	0.006	0.999
ENSGALG00000005078	4.080	0.035	0.999
ENSGALG00000006783	4.049	0.030	0.999
ENSGALG00000014004	4.037	0.006	0.999
ENSGALG00000002095	3.973	0.015	0.999
ENSGALG00000015649	3.915	0.047	0.999
ENSGALG00000009801	3.898	0.018	0.999
ENSGALG00000014971	3.890	0.030	0.999
ENSGALG00000006622	3.853	0.034	0.999
ENSGALG00000013911	3.793	0.021	0.999
ENSGALG00000002930	3.785	0.044	0.999
ENSGALG00000016487	3.770	0.017	0.999
ENSGALG00000017070	3.728	0.034	0.999
ENSGALG00000013733	3.637	0.048	0.999
ENSGALG00000016936	3.599	0.049	0.999
ENSGALG00000006050	3.597	0.012	0.999
ENSGALG00000025941	3.560	0.023	0.999
ENSGALG00000019300	3.550	0.014	0.999
ENSGALG00000008871	3.530	0.044	0.999
ENSGALG00000008222	3.505	0.039	0.999
ENSGALG00000017366	3.441	0.028	0.999
ENSGALG00000002647	3.435	0.012	0.999
ENSGALG00000007000	3.408	0.044	0.999
ENSGALG00000007012	3.330	0.022	0.999

ENSGALG00000012916	3.270	0.034	0.999
ENSGALG00000017329	3.166	0.023	0.999
ENSGALG00000001500	3.122	0.017	0.999
ENSGALG00000002862	3.108	0.024	0.999
ENSGALG00000006025	3.106	0.019	0.999
ENSGALG00000015869	3.040	0.019	0.999
ENSGALG00000001986	3.032	0.027	0.999
ENSGALG00000005226	3.023	0.034	0.999
ENSGALG00000004001	2.946	0.037	0.999
ENSGALG00000007326	2.941	0.028	0.999
ENSGALG00000002145	2.912	0.044	0.999
ENSGALG00000006118	2.831	0.041	0.999
ENSGALG00000006049	2.689	0.049	0.999
ENSGALG00000007131	2.679	0.032	0.999
ENSGALG00000012626	2.584	0.046	0.999
ENSGALG00000012339	2.519	0.012	0.999
ENSGALG00000010950	2.499	0.040	0.999
ENSGALG00000015733	2.481	0.018	0.999
ENSGALG00000012579	2.398	0.042	0.999
ENSGALG00000000523	2.318	0.047	0.999
ENSGALG00000011275	2.315	0.041	0.999
ENSGALG00000002618	2.171	0.050	0.999
ENSGALG00000001577	2.142	0.038	0.999
ENSGALG00000001852	2.028	0.012	0.999
ENSGALG00000014840	1.991	0.049	0.999
ENSGALG00000005284	1.774	0.038	0.999
ENSGALG00000003947	1.761	0.036	0.999
ENSGALG00000012754	1.761	0.042	0.999
ENSGALG00000002288	1.656	0.035	0.999
ENSGALG00000002906	1.047	0.013	0.999
ENSGALG00000007386	-1.733	0.044	0.999
ENSGALG00000015086	-2.088	0.027	0.999
ENSGALG00000009350	-2.228	0.015	0.999
ENSGALG00000002199	-2.582	0.041	0.999
ENSGALG00000006039	-2.866	0.021	0.999

Supplementary Table 11: Functional annotations and pathways associated with identified Jejunal DEGs (low vs. high)

Biological processes up-regulated in low FCR

Term	p-value	Fold enrichment	Bonferroni	Benjamini	FDR
GO:0006412~translation	1.82E-26	6.514	3.08E-23	3.08E-23	3.07E-23
GO:0002181~cytoplasmic translation	2.46E-09	11.755	4.16E-06	2.08E-06	4.15E-06
GO:0030855~epithelial cell differentiation	2.61E-04	4.500	3.56E-01	1.37E-01	4.39E-01
GO:0001895~retina homeostasis	8.56E-04	7.328	7.65E-01	3.04E-01	1.43E+00
GO:0006413~translational initiation	8.56E-04	7.328	7.65E-01	3.04E-01	1.43E+00
GO:0043171~peptide catabolic process	1.22E-03	6.840	8.74E-01	3.39E-01	2.04E+00
GO:0015986~ATP synthesis coupled proton transport	1.22E-03	6.840	8.74E-01	3.39E-01	2.04E+00
GO:0030042~actin filament depolymerization	1.81E-03	13.679	9.53E-01	3.99E-01	3.01E+00
GO:0000028~ribosomal small subunit assembly	1.82E-03	8.549	9.54E-01	3.55E-01	3.02E+00
GO:0008283~cell proliferation	4.65E-03	2.332	1.00E+00	6.26E-01	7.56E+00
GO:0006096~glycolytic process	4.96E-03	5.130	1.00E+00	6.06E-01	8.03E+00
GO:0006099~tricarboxylic acid cycle	4.96E-03	5.130	1.00E+00	6.06E-01	8.03E+00
GO:0006414~translational elongation	5.80E-03	9.771	1.00E+00	6.26E-01	9.34E+00
GO:0051764~actin crosslink formation	5.80E-03	9.771	1.00E+00	6.26E-01	9.34E+00
GO:0006406~mRNA export from nucleus	7.64E-03	4.663	1.00E+00	6.92E-01	1.21E+01
GO:0006446~regulation of translational initiation	1.19E-02	5.343	1.00E+00	8.15E-01	1.83E+01
GO:0006094~gluconeogenesis	1.33E-02	4.104	1.00E+00	8.26E-01	2.03E+01
GO:0030036~actin cytoskeleton organization	1.53E-02	2.748	1.00E+00	8.44E-01	2.28E+01
GO:0034314~Arp2/3 complex-mediated actin nucleation	1.74E-02	6.840	1.00E+00	8.62E-01	2.57E+01
GO:0051017~actin filament bundle assembly	1.82E-02	4.750	1.00E+00	8.57E-01	2.67E+01
GO:0032532~regulation of microvillus length	1.89E-02	12.824	1.00E+00	8.49E-01	2.74E+01
GO:0098609~cell-cell adhesion	2.14E-02	3.664	1.00E+00	8.69E-01	3.05E+01
GO:0000462~maturation of SSU-rRNA from tricistronic rRNA transcript (SSU-rRNA, 5.8S rRNA, LSU-rRNA)	2.63E-02	4.275	1.00E+00	9.07E-01	3.62E+01
GO:0022904~respiratory electron transport chain	2.93E-02	5.700	1.00E+00	9.19E-01	3.94E+01
GO:0006913~nucleocytoplasmic transport	2.93E-02	5.700	1.00E+00	9.19E-01	3.94E+01
GO:0071364~cellular response to epidermal growth factor stimulus	2.93E-02	5.700	1.00E+00	9.19E-01	3.94E+01
GO:1900121~negative regulation of receptor binding	3.02E-02	10.259	1.00E+00	9.15E-01	4.04E+01
GO:0051014~actin filament severing	3.02E-02	10.259	1.00E+00	9.15E-01	4.04E+01
GO:0090004~positive regulation of establishment of protein localization to plasma membrane	3.11E-02	4.071	1.00E+00	9.11E-01	4.13E+01
GO:0007015~actin filament organization	3.63E-02	3.206	1.00E+00	9.34E-01	4.63E+01

GO:0006936~muscle contraction	3.63E-02	3.886	1.00E+00	9.26E-01	4.64E+01
GO:0007163~establishment or maintenance of cell polarity	3.65E-02	5.261	1.00E+00	9.19E-01	4.65E+01
GO:0006457~protein folding	3.72E-02	2.192	1.00E+00	9.15E-01	4.72E+01
GO:0050821~protein stabilization	3.73E-02	2.332	1.00E+00	9.07E-01	4.73E+01
GO:0070527~platelet aggregation	4.20E-02	3.717	1.00E+00	9.25E-01	5.15E+01
GO:0039702~viral budding via host ESCRT complex	4.36E-02	8.549	1.00E+00	9.25E-01	5.28E+01
GO:0016477~cell migration	4.40E-02	2.022	1.00E+00	9.21E-01	5.32E+01
GO:0042254~ribosome biogenesis	5.32E-02	4.560	1.00E+00	9.49E-01	6.02E+01
GO:0000027~ribosomal large subunit assembly	5.32E-02	4.560	1.00E+00	9.49E-01	6.02E+01
GO:0051603~proteolysis involved in cellular protein catabolic process	5.48E-02	3.420	1.00E+00	9.49E-01	6.13E+01
GO:0006364~rRNA processing	5.62E-02	2.850	1.00E+00	9.48E-01	6.23E+01
GO:0006122~mitochondrial electron transport, ubiquinol to cytochrome c	5.87E-02	7.328	1.00E+00	9.51E-01	6.39E+01
GO:0042274~ribosomal small subunit biogenesis	5.87E-02	7.328	1.00E+00	9.51E-01	6.39E+01
GO:0030836~positive regulation of actin filament depolymerization	5.87E-02	7.328	1.00E+00	9.51E-01	6.39E+01
GO:0030224~monocyte differentiation	5.87E-02	7.328	1.00E+00	9.51E-01	6.39E+01
GO:0030216~keratinocyte differentiation	6.19E-02	3.288	1.00E+00	9.54E-01	6.59E+01
GO:0043154~negative regulation of cysteine-type endopeptidase activity involved in apoptotic process	6.19E-02	3.288	1.00E+00	9.54E-01	6.59E+01
GO:0042273~ribosomal large subunit biogenesis	6.28E-02	4.275	1.00E+00	9.52E-01	6.65E+01
GO:0098792~xenophagy	6.94E-02	2.394	1.00E+00	9.62E-01	7.02E+01
GO:0010501~RNA secondary structure unwinding	6.94E-02	3.166	1.00E+00	9.59E-01	7.02E+01
GO:0043534~blood vessel endothelial cell migration	7.53E-02	6.412	1.00E+00	9.66E-01	7.33E+01
GO:0055117~regulation of cardiac muscle contraction	7.53E-02	6.412	1.00E+00	9.66E-01	7.33E+01
GO:0003382~epithelial cell morphogenesis	7.53E-02	6.412	1.00E+00	9.66E-01	7.33E+01
GO:0042026~protein refolding	7.53E-02	6.412	1.00E+00	9.66E-01	7.33E+01
GO:1900026~positive regulation of substrate adhesion-dependent cell spreading	8.40E-02	3.800	1.00E+00	9.75E-01	7.72E+01
GO:0001525~angiogenesis	8.75E-02	1.859	1.00E+00	9.77E-01	7.87E+01
GO:0000463~maturation of LSU-rRNA from tricistronic rRNA transcript (SSU-rRNA, 5.8S rRNA, LSU-rRNA)	9.32E-02	5.700	1.00E+00	9.80E-01	8.08E+01
GO:0022408~negative regulation of cell-cell adhesion	9.32E-02	5.700	1.00E+00	9.80E-01	8.08E+01
GO:0032968~positive regulation of transcription elongation from RNA polymerase II promoter	9.32E-02	5.700	1.00E+00	9.80E-01	8.08E+01
GO:0007283~spermatogenesis	9.39E-02	1.924	1.00E+00	9.79E-01	8.10E+01
GO:0000902~cell morphogenesis	9.59E-02	2.443	1.00E+00	9.79E-01	8.17E+01

KEGG pathways up-regulated in low FCR

Term	p-value	Fold enrichment	Bonferroni	Benjamini	FDR
gga03010:Ribosome	1.67E-34	6.401	2.29E-32	2.29E-32	1.96E-31
gga00190:Oxidative phosphorylation	1.54E-06	2.895	2.11E-04	1.05E-04	1.81E-03
gga03320:PPAR signaling pathway	4.63E-04	3.038	6.15E-02	2.09E-02	5.43E-01
gga04260:Cardiac muscle contraction	1.87E-03	2.774	2.27E-01	6.23E-02	2.18E+00
gga00071:Fatty acid degradation	3.07E-03	3.492	3.43E-01	8.07E-02	3.55E+00
gga03040:Spliceosome	6.63E-03	2.073	5.98E-01	1.41E-01	7.52E+00
gga00620:Pyruvate metabolism	1.40E-02	3.012	8.55E-01	2.41E-01	1.53E+01
gga01230:Biosynthesis of amino acids	1.45E-02	2.387	8.64E-01	2.21E-01	1.57E+01
gga00010:Glycolysis / Gluconeogenesis	1.55E-02	2.510	8.82E-01	2.11E-01	1.67E+01
gga04141:Protein processing in endoplasmic reticulum	1.73E-02	1.754	9.09E-01	2.13E-01	1.86E+01
gga01200:Carbon metabolism	3.37E-02	1.867	9.91E-01	3.48E-01	3.32E+01
gga04520:Adherens junction	3.60E-02	2.071	9.93E-01	3.42E-01	3.50E+01
gga01130:Biosynthesis of antibiotics	4.24E-02	1.548	9.97E-01	3.67E-01	3.99E+01
gga03013:RNA transport	4.62E-02	1.661	9.98E-01	3.70E-01	4.26E+01
gga00020:Citrate cycle (TCA cycle)	6.19E-02	2.743	1.00E+00	4.42E-01	5.28E+01

Biological Processes down-regulated in high FCR

Term	p-value	Fold enrichment	Bonferroni	Benjamini	FDR
GO:0055114~oxidation-reduction process	0.014	7.821	0.923	0.923	15.532
GO:0044030~regulation of DNA methylation	0.020	97.761	0.977	0.850	22.057
GO:0007188~adenylate cyclase-modulating G-protein coupled receptor signaling pathway	0.086	21.725	1.000	0.997	67.444

Supplementary Table 12: Functional annotations and pathways associated with identified Ileal DEGs (low vs. high)

Annotation table for up-regulated genes in low FCR

ID	Gene	COG_ONTOLOGY	GOTERM_BP	GOTERM_CC	GOTERM_MF	INTERPRO	KEGG_PATHWAY	PIR_SUPERFAMILY	SMART
ENSGALG00000008372	5'-3' exoribonuclease 2(XRN2)	DNA replication, recombination, and repair / Cell division and chromosome partitioning / Translation,	GO:0000738~DNA catabolic process, exonucleolytic,GO:0006353~DNA-templated transcription, termination,GO:0006355~regulation of transcription, DNA-templated,GO:0006397~mRNA processing,GO:0007283~spermatogenesis,GO:0016049~cell growth,GO:0090503~RNA phosphodiester bond hydrolysis, exonucleolytic,	GO:0005730~nucleolus,GO:0016020~membrane,GO:0016235~aggresome,	GO:0000175~3'-5'-exoribonuclease activity,GO:0001147~transcription termination site sequence-specific DNA binding,GO:0004534~5'-3' exoribonuclease activity,GO:0008409~5'-3' exonuclease activity,GO:0044822~poly(A) RNA binding,GO:0046872~metal ion binding,	IPR004859:Putative 5-3 exonuclease,IPR017151:5'-3' exoribonuclease 2,IPR027073:5'-3' exoribonuclease,	gga03008:Ribosome biogenesis in eukaryotes,gga03018:RNA degradation,	PIRSF037239:5'-3' exonuclease, Xrn2 type,	
ENSGALG00000004348	BRCA1 associated ATM activator 1(BRAT1)		GO:0001934~positive regulation of protein phosphorylation,GO:0006006~glucose metabolic process,GO:0006915~apoptotic process,GO:0006974~cellular response to DNA damage stimulus,GO:0008283~cell proliferation,GO:0010212~response to ionizing radiation,GO:0016049~cell growth,GO:0016477~cell migration,GO:0051646~mitochondrion localization,	GO:0005634~nucleus,GO:0005737~cytoplasm,GO:0016020~membrane,		IPR011989:Armadillo-like helical,IPR016024:Armadillo-type fold,			
ENSGALG00000026710	DnaJ heat shock protein family (Hsp40) member		GO:0000301~retrograde transport, vesicle recycling within Golgi,GO:0006890~retrograde vesicle-mediated transport, Golgi to ER,GO:0007030~Golgi	GO:0017119~Golgi transport complex,		IPR001623:DnaJ domain,IPR018961:DnaJ homologue, subfamily C, member 28,			SM00271:DnaJ,

	C28(DNAJ C28)		organization,GO:0048213~ Golgi vesicle prefusion complex stabilization,			conserved domain,				
ENSGALG 000000119 10	MNAT CDK-activating kinase assembly factor 1(MNAT1)		GO:0000079~regulation of cyclin-dependent protein serine/threonine kinase activity,GO:0006281~DNA repair,GO:0006357~regulation of transcription from RNA polymerase II promoter,GO:0006366~transcription from RNA polymerase II promoter,GO:0007049~cell cycle,GO:0007512~adult heart development,GO:0021591~ ventricular system development,GO:0045944~ positive regulation of transcription from RNA polymerase II promoter,GO:0051592~response to calcium ion,	GO:0005675~holo TFIIH complex,GO:0005737~cytoplasm,	GO:0008094~DNA-dependent ATPase activity,GO:0008353~RNA polymerase II carboxy-terminal domain kinase activity,	IPR003903:Ubiquitin interacting motif,IPR004575:Cdk-activating kinase assembly factor MAT1/Tfb3,IPR013083:Zinc finger, RING/FYVE/PHD-type,IPR015877:Cdk-activating kinase assembly factor MAT1, centre,IPR017907:Zinc finger, RING-type, conserved site,	gga03022:Basal transcription factors,gga03420:Nucleotide excision repair,	PIRSF003338:CDK-activating kinase assembly factor MAT1,	
ENSGALG 000000062 98	autophagy related 4B, cysteine peptidase(ATG4B)		GO:0000045~autophagosome assembly,GO:0000422~mitophagy,GO:0006501~C-terminal protein lipidation,GO:0006612~protein targeting to membrane,GO:0015031~protein transport,GO:0016485~protein processing,GO:0044804~nucleophagy,GO:0051697~protein delipidation,	GO:0005737~cytoplasm,GO:0005829~cytosol,	GO:0004197~cysteine-type endopeptidase activity,	IPR005078:Peptidase C54,	gga04140:Regulation of autophagy,		
ENSGALG 000000027 78	chromosome 26 open reading frame, human C6orf106(C		GO:0016236~macroautophagy,	GO:0000407~pre-autophagosomal structure,GO:0005776~autophagosome,	GO:0043130~ubiquitin binding,	IPR009060:UBA-like,			

	26H6ORF106)								
ENSGALG00000005469	cytochrome P-450 2C45(CYP2C45)	Secondary metabolites biosynthesis, transport, and catabolism,			GO:0005506~iron ion binding,GO:0016712~oxidoreductase activity, acting on paired donors, with incorporation or reduction of molecular oxygen, reduced flavin or flavoprotein as one donor, and incorporation of one atom of oxygen,GO:0019825~oxygen binding,GO:0020037~heme binding,	IPR001128:Cytochrome P450,IPR002401:Cytochrome P450, E-class, group I,IPR008067:Cytochrome P450, E-class, group I, CYP2A-like,IPR017972:Cytochrome P450, conserved site,	gga00140:Steroid hormone biosynthesis,gga00590:Arachidonic acid metabolism,gga00591:Linoleic acid metabolism,gga00830:Retinol metabolism,gga01100:Metabolic pathways,		
ENSGALG00000007581	forty-two-three domain containing 1(FYTTD1)		GO:0006406~mRNA export from nucleus,	GO:0005654~nucleoplasm,GO:0016607~nuclear speck,	GO:0003729~mRNA binding,	IPR009782:Protein of unknown function DUF1346,			
ENSGALG00000014170	monoacylglycerol O-acyltransferase 2(MOGAT2)						gga00561:Glycerolipid metabolism,		
ENSGALG00000025818	potassium two pore domain channel subfamily K member 5(KCNK5)		GO:0030322~stabilization of membrane potential,GO:0071805~potassium ion transmembrane transport,	GO:0005887~integral component of plasma membrane,	GO:0005249~voltage-gated potassium channel activity,GO:0022841~potassium ion leak channel activity,	IPR003092:Two pore domain potassium channel, TASK family,IPR003280:Two pore domain potassium channel,IPR013099:Ion transport 2,			
ENSGALG00000001332	retinoblastoma-like 1						gga04110:Cell cycle,gga04350:TGF-beta		

	(p107)(RBL 1)						signaling pathway,		
ENSGALG 00000015339	ribosomal protein L24(RPL24)		GO:0000027~ribosomal large subunit assembly,GO:0006412~translation,GO:1902626~assembly of large subunit precursor of preribosome,	GO:0016020~membrane,GO:0022625~cytosolic large ribosomal subunit,GO:0070062~extracellular exosome,	GO:0003735~structural constituent of ribosome,GO:0044822~poly(A) RNA binding,	IPR000988:Ribosomal protein L24e-related,IPR011017:TRASH domain,IPR023442:Ribosomal protein L24e, conserved site,	gga03010:Ribosome,		SM00746:TRASH,
ENSGALG 00000003155	serine racemase(SRR)		GO:0006563~L-serine metabolic process,GO:0032496~response to lipopolysaccharide,GO:0042866~pyruvate biosynthetic process,GO:0051289~protein homotetramerization,GO:0070179~D-serine biosynthetic process,	GO:0005737~cytoplasm,GO:0043025~neuronal cell body,	GO:0000287~magnesium ion binding,GO:0003941~L-serine ammonia-lyase activity,GO:0005509~calcium ion binding,GO:0005524~ATP binding,GO:0008721~D-serine ammonia-lyase activity,GO:0016594~glycine binding,GO:0018114~threonine racemase activity,GO:0030170~pyridoxal phosphate binding,GO:0030378~serine racemase activity,	IPR000634:Serine/threonine dehydratase, pyridoxal-phosphate-binding site,IPR001926:Tryptophan synthase beta subunit-like PLP-dependent enzymes superfamily,	gga00260:Glycine, serine and threonine metabolism,		
ENSGALG 00000023571	tripartite motif containing 59(TRIM59)		GO:0042384~cilium assembly,GO:0043124~negative regulation of I-kappaB kinase/NF-kappaB signaling,GO:0045087~innate immune response,GO:0046597~negative regulation of viral entry into host cell,	GO:0005783~endoplasmic reticulum,GO:0005789~endoplasmic reticulum membrane,GO:0005813~centrosome,GO:0005929~cilium,GO:0016021~integral component of membrane,GO:0030992~intraciliary transport particle B,	GO:0008270~zinc ion binding,GO:0061630~ubiquitin protein ligase activity,	IPR000315:Zinc finger, B-box,IPR001841:Zinc finger, RING-type,IPR013083:Zinc finger, RING/FYVE/PHD-type,IPR017907:Zinc finger, RING-			SM00184:RING,

						type, conserved site,IPR027370:RING-type zinc-finger, LisH dimerisation motif,			
ENSGALG00000009792	uncharacterized LOC422442 (LOC422442)			GO:0016021~integral component of membrane,		IPR007237:CD20-like,			

Annotation table for down-regulated genes in low FCR

ID	Gene Name	GOTERM_BP_DIRECT	GOTERM_CC	GOTERM_MF	INTERPRO	KEGG_PATHWAY	PIR_SUPERFAMILY	SMART
ENSGALG00000011992	E1A binding protein p300(EP300)	GO:0000122~negative regulation of transcription from RNA polymerase II promoter,GO:0001666~response to hypoxia,GO:0001756~somitogenesis,GO:0006990~positive regulation of transcription from RNA polymerase II promoter involved in unfolded protein response,GO:0007507~heart development,GO:0007519~skeletal muscle tissue development,GO:0007623~circadian rhythm,GO:0009887~organ morphogenesis,GO:0018076~N-terminal peptidyl-lysine acetylation,GO:0030183~B cell differentiation,GO:0030220~platelet formation,GO:0030324~lung development,GO:0032092~positive regulation of protein binding,GO:0034644~cellular response to UV,GO:0035855~megakaryocyte development,GO:0042771~intrinsic apoptotic signaling pathway in response to DNA damage by	GO:0000123~histone acetyltransferase complex,GO:0005667~transcription factor complex,GO:0005737~cytoplasm,	GO:0000979~RNA polymerase II core promoter sequence-specific DNA binding,GO:0001228~transcriptional activator activity, RNA polymerase II transcription regulatory region sequence-specific binding,GO:0003684~damaged DNA binding,GO:0003713~transcription coactivator activity,GO:0004402~histone acetyltransferase activity,GO:0004468~lysine N-acetyltransferase activity, acting on acetyl phosphate as donor,GO:0008270~zinc ion binding,GO:0031490~chromatin DNA binding,GO:0097157~pre-mRNA intronic binding,	IPR000197:Zinc finger, TAZ-type,IPR000433:Zinc finger, ZZ-type,IPR001487:Bromodomain,IPR003101:Coactivator CBP, KIX domain,IPR009110:Nuclear receptor coactivator, interlocking,IPR010303:Domain of unknown function DUF902, CREBbp,IPR013178:Histone H3-K56 acetyltransferase, RTT109,IPR014744:Nuclear receptor coactivator, CREB-bp-like, interlocking,IP	gga04068:FoxO signaling pathway,gga04110:Cell cycle,gga04310:Wnt signaling pathway,gga04330:Notch signaling pathway,gga04350:TGF-beta signaling pathway,gga04520:Adherens junction,gga04630:Jak-STAT signaling pathway,gga04916:Melanogenesis,gga05164:Influenza A,gga05168:Herpes simplex infection,		SM00291:ZnF_ZZ,SM00297:BROMO,SM00551:ZnF_TAZ,SM01250:SM01250,

		p53 class mediator,GO:0043627~response to estrogen,GO:0043923~positive regulation by host of viral transcription,GO:0043967~histone H4 acetylation,GO:0043969~histone H2B acetylation,GO:0045444~fat cell differentiation,GO:0050821~protein stabilization,GO:0051091~positive regulation of sequence-specific DNA binding transcription factor activity,GO:0060765~regulation of androgen receptor signaling pathway,GO:0090043~regulation of tubulin deacetylation,			R018359:Bromodomain, conserved site,			
ENSGALG00000004160	GTPase activating protein (SH3 domain) binding protein 1(G3BP1)	GO:0006810~transport,GO:0090090~negative regulation of canonical Wnt signaling pathway,	GO:0005829~cytosol, GO:0005925~focal adhesion,GO:0010494~cytoplasmic stress granule,GO:0030529~intracellular ribonucleoprotein complex,	GO:0000166~nucleotide binding,GO:0003729~mRNA binding,	IPR000504:RNA recognition motif domain,IPR002075:Nuclear transport factor 2,IPR012677:Nucleotide-binding, alpha-beta plait,IPR018222:Nuclear transport factor 2, Eukaryote,			SM00360:RRM,
ENSGALG00000007615	RAB11A, member RAS oncogene family(RAB11A)	GO:0000910~cytokinesis,GO:0006887~exocytosis,GO:0007080~mitotic metaphase plate congression,GO:0007264~small GTPase mediated signal transduction,GO:0010634~positive regulation of epithelial cell migration,GO:0010796~regulation of multivesicular body size,GO:0010971~positive regulation of G2/M transition of mitotic cell cycle,GO:0015031~protein transport,GO:0030953~astral microtubule organization,GO:0031175~neur	GO:0000922~spindle pole,GO:0005739~mitochondrion,GO:0005771~multivesicular body,GO:0005802~trans-Golgi network,GO:0005813~centrosome,GO:0005828~kinetochore microtubule,GO:0005886~plasma membrane,GO:0030424~axon,GO:0032154~cleavage furrow,GO:0043234~protein	GO:0003924~GTPase activity,GO:0005525~GTP binding,GO:0008017~microtubule binding,GO:0031489~myosin V binding,	IPR001806:Small GTPase superfamily,IPR005225:Small GTP-binding protein domain,IPR027417:P-loop containing nucleoside triphosphate hydrolase,	gga04144:Endocytosis,		

		on projection development,GO:0032402~melanosome transport,GO:0036258~multivesicular body assembly,GO:0045773~positive regulation of axon extension,GO:0060627~regulation of vesicle-mediated transport,GO:0072594~establishment of protein localization to organelle,GO:0072659~protein localization to plasma membrane,GO:0090150~establishment of protein localization to membrane,GO:0090307~mitotic spindle assembly,GO:1990182~exosomal secretion,	complex,GO:0045335~phagocytic vesicle,GO:0048471~perinuclear region of cytoplasm,GO:0055037~recycling endosome,GO:0055038~recycling endosome membrane,GO:0070062~extracellular exosome,					
ENSGALG00000026302	SRP receptor beta subunit(SRPRB)	GO:0007264~small GTPase mediated signal transduction,	GO:0005881~cytoplasmic microtubule,GO:0016021~integral component of membrane,	GO:0005525~GTP binding,	IPR019009:Signal recognition particle receptor, beta subunit,IPR024156:Small GTPase superfamily, ARF type,IPR027417:P-loop containing nucleoside triphosphate hydrolase,	gga03060:Protein export,		
ENSGALG00000010878	USO1 vesicle transport factor(USO1)	GO:0006886~intracellular protein transport,GO:0006888~ER to Golgi vesicle-mediated transport,GO:0007030~Golgi organization,GO:0045056~transcytosis,GO:0048211~Golgi vesicle docking,GO:0048280~vesicle fusion with Golgi apparatus,GO:0061025~membrane fusion,	GO:0000139~Golgi membrane,GO:0005730~nucleolus,GO:0005783~endoplasmic reticulum,GO:0005795~Golgi stack,GO:0005829~cytosol,GO:0012507~ER to Golgi transport vesicle membrane,GO:0048471~perinuclear region of cytoplasm,	GO:0008565~protein transporter activity,GO:0044822~poly(A) RNA binding,	IPR000225:Armadillo,IPR006953:Vesicle tethering protein Uso1/P115-like, head domain,IPR006955:Uso1/p115-like vesicle tethering protein, C-terminal,IPR016024:Armadillo-type fold,IPR024095			SM00185:ARM,

					:Vesicle tethering protein p115-like,			
ENSGALG00000010547	Yip1 domain family member 4(YIPF4)		GO:0005783~endoplasmic reticulum,GO:0005794~Golgi apparatus,GO:0005886~plasma membrane,GO:0016021~integral component of membrane,		IPR006977:Yip1 domain,			
ENSGALG00000012135	activating transcription factor 4(ATF4)	GO:0006094~gluconeogenesis, GO:0006357~regulation of transcription from RNA polymerase II promoter,GO:0010575~positive regulation of vascular endothelial growth factor production,GO:0032922~circadian regulation of gene expression,GO:0034198~cellular response to amino acid starvation,GO:0036091~positive regulation of transcription from RNA polymerase II promoter in response to oxidative stress,GO:0036499~PERK-mediated unfolded protein response,GO:0042149~cellular response to glucose starvation,GO:0042789~mRNA transcription from RNA polymerase II promoter,GO:0043065~positive regulation of apoptotic process,GO:0043525~positive regulation of neuron apoptotic process,GO:0045943~positive regulation of transcription from RNA polymerase I promoter,GO:0070059~intrinsic apoptotic signaling pathway in response to endoplasmic reticulum stress,GO:1903204~negative regulation of oxidative stress-induced neuron death,GO:1990440~positive	GO:0005634~nucleus, GO:0005737~cytoplasm,GO:0034399~nuclear periphery,GO:0043005~neuron projection,GO:1990037~Lewy body core,GO:1990589~ATF4-CREB1 transcription factor complex,GO:1990590~ATF1-ATF4 transcription factor complex,GO:1990617~CHOP-ATF4 complex,	GO:0000977~RNA polymerase II regulatory region sequence-specific DNA binding,GO:0000978~RNA polymerase II core promoter proximal region sequence-specific DNA binding,GO:0000981~RNA polymerase II transcription factor activity, sequence-specific DNA binding,GO:0001046~core promoter sequence-specific DNA binding,GO:0001077~transcriptional activator activity, RNA polymerase II core promoter proximal region sequence-specific binding,GO:0003700~transcription factor activity, sequence-specific DNA binding,GO:0043565~sequence-specific DNA binding,	IPR004827:Basic-leucine zipper domain,	gga04010:MAPK signaling pathway,gga04141:Protein processing in endoplasmic reticulum,gga04261:Adrenergic signaling in cardiomyocytes,gga04912:GnRH signaling pathway,		SM00338:BRLZ,

		regulation of transcription from RNA polymerase II promoter in response to endoplasmic reticulum stress,						
ENSGALG00000007989	activity-dependent neuroprotector homeobox(ADNP)	GO:0043524~negative regulation of neuron apoptotic process,	GO:0005615~extracellular space,GO:0005634~nucleus,	GO:0003677~DNA binding,GO:0003682~chromatin binding,GO:0046872~metal ion binding,	IPR001356:Homeodomain,IPR007087:Zinc finger, C2H2,IPR009057:Homeodomain-like,IPR015880:Zinc finger, C2H2-like,			SM00355:ZnF_C2H2,SM00389:HOX,
ENSGALG00000004725	aldehyde dehydrogenase 2 family (mitochondrial)(ALDH2)					gga00010:Glycolysis / Gluconeogenesis,gga00040:Pentose and glucuronate interconversions,gga00053:Ascorbate and aldarate metabolism,gga00071:Fatty acid degradation,gga00280:Valine, leucine and isoleucine degradation,gga00310:Lysine degradation,gga00330:Arginine and proline metabolism,gga00340:Histidine metabolism,gga00380:Tryptophan metabolism,gga00410:beta-Alanine metabolism,gga00561:Glycerolipid metabolism,gga00620:Pyruvate metabolism,gga		

						01100:Metabolic pathways,gga01130:Biosynthesis of antibiotics,		
ENSGALG00000008738	alpha-aspartyl dipeptidase(LOC424109)			GO:0008233~peptidase activity,GO:0008236~serine-type peptidase activity,	IPR005320:Peptidase S51,			
ENSGALG00000002145	calmodulin regulated spectrin-associated protein family, member 2(CAMSAP2)	GO:0000226~microtubule cytoskeleton organization,GO:0031175~neuron projection development,GO:0033043~regulation of organelle organization,	GO:0005813~centrosome,		IPR001715:Calponin homology domain,IPR011033:PRC-barrel-like,IPR014797:CKK domain,IPR022613:Calmodulin-regulated spectrin-associated protein, CH domain,			SM01051:SM01051,
ENSGALG00000016609	centromere protein O(CENPO)	GO:0034508~centromere complex assembly,	GO:0000776~kinetochore,GO:0005634~nucleus,GO:0005654~nucleoplasm,		IPR018464:Centromere protein Cenp-O,			
ENSGALG00000011168	chromosome 1 open reading frame, human C12orf29(C12ORF29)	GO:0002244~hematopoietic progenitor cell differentiation,						
ENSGALG00000016736	cytoplasmic FMR1 interacting protein 1(CYFIP1)	GO:0016601~Rac protein signal transduction,GO:0030032~lamellipodium assembly,GO:0048675~axon extension,GO:0050890~cognition,GO:2000601~positive regulation of Arp2/3 complex-mediated actin nucleation,	GO:0005845~mRNA cap binding complex,GO:0005925~focal adhesion,GO:0030027~lamellipodium,GO:0031209~SCAR complex,GO:0043005~neuron projection,GO:0048471~perinuclear region of cytoplasm,GO:0070062~extracellular exosome,		IPR008081:Cytoplasmic FMR1-interacting,IPR009828:Protein of unknown function DUF1394,	gga03013:RNA transport,gga04810:Regulation of actin cytoskeleton,	PIRSF008153:cytoplasmic FMR1-interacting protein,	
ENSGALG00000000242	early B-cell factor 2(EBF2)	GO:0001709~cell fate determination,GO:0006351~transcription, DNA-	GO:0005634~nucleus,	GO:0000978~RNA polymerase II core promoter proximal region	IPR002909:Cell surface receptor IPT/TIG,IPR00			SM00429:IPT,

		templated,GO:0006355~regulation of transcription, DNA-templated,GO:0007275~multicellular organism development,GO:0035563~positive regulation of chromatin binding,GO:0045944~positive regulation of transcription from RNA polymerase II promoter,GO:0050873~brown fat cell differentiation,GO:0060612~adipose tissue development,		sequence-specific DNA binding,GO:0001077~transcriptional activator activity, RNA polymerase II core promoter proximal region sequence-specific binding,GO:0003677~DNA binding,GO:0003682~chromatin binding,GO:0046872~metal ion binding,	3523:Transcription factor COE,IPR013783:Immunoglobulin-like fold,IPR014756:Immunoglobulin E-set,IPR018350:Transcription factor COE, conserved site,			
ENSGALG00000014773	erbb2 interacting protein(ERBB2IP)	GO:0006605~protein targeting,GO:0032088~negative regulation of NF-kappaB transcription factor activity,GO:0032495~response to muramyl dipeptide,GO:0032496~response to lipopolysaccharide,GO:0070433~negative regulation of nucleotide-binding oligomerization domain containing 2 signaling pathway,GO:0071356~cellular response to tumor necrosis factor,GO:0071638~negative regulation of monocyte chemotactic protein-1 production,	GO:0005654~nucleoplasm,GO:0005737~cytoplasm,GO:0016323~basolateral plasma membrane,GO:0030056~hemidesmosome,		IPR001478:PDZ domain,IPR001611:Leucine-rich repeat,IPR003591:Leucine-rich repeat, typical subtype,	gga04621:NOD-like receptor signaling pathway,		SM00228:PDZ,SM00369:LRR_TYP,
ENSGALG00000026203	family with sequence similarity 174, member A(FAM174A)		GO:0016021~integral component of membrane,		IPR009565:Protein of unknown function DUF1180,			
ENSGALG00000012613	fructose-1,6-bisphosphatase 1(FBP1)	GO:0006001~fructose catabolic process,GO:0006094~gluconeogenesis,	GO:0005829~cytosol,	GO:0042132~fructose 1,6-bisphosphate 1-phosphatase activity,	IPR000146:Fructose-1,6-bisphosphatase class 1/Sedoheptulose-1,7-bisphosphatase,	gga00010:Glycolysis / Gluconeogenesis,gga00030:Pentose phosphate pathway,gga00051:Fructose and mannose metabolism,gga01100:Metabolic pathways,gga01130:Biosynthesi		

						s of antibiotics,gga01200:Carbon metabolism,gga04910:Insulin signaling pathway,		
ENSGALG00000016285	glycerol kinase(GK)	GO:0006071~glycerol metabolic process,GO:0006641~triglyceride metabolic process,GO:0046167~glycerol-3-phosphate biosynthetic process,	GO:0005739~mitochondrion,	GO:0004370~glycerol kinase activity,	IPR005999:Glycerol kinase,IPR018483:Carbohydrate kinase, FGGY, conserved site,IPR018484:Carbohydrate kinase, FGGY, N-terminal,IPR018485:Carbohydrate kinase, FGGY, C-terminal,	gga00561:Glycerolipid metabolism,gga01100:Metabolic pathways,gga03320:PPAR signaling pathway,	PIRSF000538:keto-sugar kinase,	
ENSGALG00000001031	heat shock transcription factor family member 5(HSF5)		GO:0005634~nucleus,	GO:0003700~transcription factor activity, sequence-specific DNA binding,GO:0043565~sequence-specific DNA binding,	IPR000232:Heat shock factor (HSF)-type, DNA-binding,IPR011991:Winged helix-turn-helix DNA-binding domain,			SM00415:HSF,
ENSGALG00000000091	integrator complex subunit 4(INTS4)	GO:0016180~snRNA processing,	GO:0032039~integrator complex,		IPR011989:Armadillo-like helical,IPR016024:Armadillo-type fold,IPR026003:HEAT repeat associated with sister chromatid cohesion protein,			
ENSGALG00000011608	inverted formin, FH2 and WH2 domain containing(INF2)	GO:0030036~actin cytoskeleton organization,GO:0090140~regulation of mitochondrial fission,	GO:0048471~perinuclear region of cytoplasm,		IPR003124:WH2 domain,IPR009408:Formin Homology 1,IPR010472:Diaphanous			SM00498:FH2, SM01139:SM01139,SM01140:SM01140,

					FH3,IPR010473:Diaphanous GTPase-binding,IPR014768:GTPase-binding/formin homology 3,IPR015425:Actin-binding FH2,IPR016024:Armadillo-type fold,			
ENSGALG00000008972	mitofusin 1(MFN1)	GO:0008053~mitochondrial fusion,	GO:0005741~mitochondrial outer membrane,GO:0016021~integral component of membrane,	GO:0003924~GTPase activity,GO:0005525~GTP binding,	IPR006884:Fzo/mitofusin HR2 domain,IPR022812:Dynamin,IPR027088:Mitofusin-1,IPR027094:Mitofusin family,IPR027417:P-loop containing nucleoside triphosphate hydrolase,			
ENSGALG00000010017	multiple coagulation factor deficiency 2(MCFD2)			GO:0005509~calcium ion binding,	IPR002048:EF-hand domain,IPR011992:EF-hand-like domain,IPR018247:EF-Hand 1, calcium-binding site,			
ENSGALG00000014756	peptidylprolyl isomerase domain and WD repeat containing 1(PPWD1)	GO:0006457~protein folding,	GO:0071013~catalytic step 2 spliceosome,	GO:0003755~peptidyl-prolyl cis-trans isomerase activity,	IPR001680:WD40 repeat,IPR002130:Cyclophilin-like peptidyl-prolyl cis-trans isomerase domain,IPR015943:WD40/YVTN repeat-like-containing domain,IPR017986:WD40-repeat-containing			SM00320:WD40,

					domain,IPR024936:Cyclophilin-type peptidyl-prolyl cis-trans isomerase,			
ENSGALG00000005805	phospholipase C, delta 1(PLCD1)	GO:0001525~angiogenesis,GO:0016042~lipid catabolic process,GO:0035556~intracellular signal transduction,GO:0042127~regulation of cell proliferation,	GO:0005829~cytosol,GO:0070062~extracellular exosome,	GO:0001786~phosphatidylserine binding,GO:0004435~phosphatidylinositol phospholipase C activity,GO:0004871~signal transducer activity,GO:0005509~calcium ion binding,GO:0070300~phosphatidic acid binding,GO:1901981~phosphatidylinositol phosphate binding,	IPR000008:C2 calcium-dependent membrane targeting,IPR000909:Phospholipase C, phosphatidylinositol-specific , X domain,IPR001192:Phosphoinositide phospholipase C,IPR001711:Phospholipase C, phosphatidylinositol-specific, Y domain,IPR001849:Pleckstrin homology domain,IPR002048:EF-hand domain,IPR011992:EF-hand-like domain,IPR011993:Pleckstrin homology-like domain,IPR015359:Phospholipase C, phosphoinositol-specific, EF-hand-like,IPR017946:PLC-like phosphodiesterase, TIM beta/alpha-barrel domain,IPR018247:EF-Hand 1, calcium-binding site,	gga00562:Inositol phosphate metabolism,gga01100:Metabolic pathways,gga04020:Calcium signaling pathway,gga04070:Phosphatidylinositol signaling system,		SM00148:PLCXc,SM00149:PLCYc,SM00233:PH,SM00239:C2,

ENSGALG00000002169	pleckstrin homology domain containing, family B (evectins) member 2(PLEKHB2)	GO:0045595~regulation of cell differentiation,	GO:0016021~integral component of membrane,GO:0055038~recycling endosome membrane,		IPR001849:Pleckstrin homology domain,IPR011993:Pleckstrin homology-like domain,			SM00233:PH,
ENSGALG00000009228	poly (ADP-ribose) polymerase 1(PARP1)	GO:0006273~lagging strand elongation,GO:0006302~double-strand break repair,GO:0006471~protein ADP-ribosylation,GO:0032869~cellular response to insulin stimulus,GO:0034599~cellular response to oxidative stress,GO:0043504~mitochondrial DNA repair,GO:0051103~DNA ligation involved in DNA repair,GO:0070212~protein poly-ADP-ribosylation,GO:1903827~regulation of cellular protein localization,GO:1990966~ATP generation from poly-ADP-D-ribose,	GO:0000784~nuclear chromosome, telomeric region,GO:0005634~nucleus,GO:0005635~nuclear envelope,GO:0005667~transcription factor complex,GO:0005730~nucleolus,GO:0005737~cytoplasm,GO:0005739~mitochondrion, GO:0016020~membrane,	GO:0003677~DNA binding,GO:0003910~DNA ligase (ATP) activity,GO:0003950~NAD+ ADP-ribosyltransferase activity,GO:0008270~zinc ion binding,GO:0044822~poly(A) RNA binding,GO:0051287~NAD binding,	IPR001357:BRCT domain,IPR001510:Zinc finger, PARP-type,IPR004102:Poly(ADP-ribose) polymerase, regulatory domain,IPR008288:NAD+ ADP-ribosyltransferase,IPR008893: WGR domain,IPR012317:Poly(ADP-ribose) polymerase, catalytic domain,IPR012982:PADR1,	gga03410:Base excision repair,	PIRSF000489:poly(ADP-ribose) polymerase,	SM00292:BRCT,SM00773:WGR,SM01335:SM01335,SM01336:SM01336,
ENSGALG00000013867	protein tyrosine phosphatase, non-receptor type 2(PTPN2)	GO:0008285~negative regulation of cell proliferation,GO:0008286~insulin receptor signaling pathway,GO:0010804~negative regulation of tumor necrosis factor-mediated signaling pathway,GO:0010888~negative regulation of lipid storage,GO:0030183~B cell differentiation,GO:0030217~T cell differentiation,GO:0030218~erythrocyte differentiation,GO:0042059~negative regulation of epidermal growth factor receptor signaling pathway,GO:0042512~negative regulation of tyrosine	GO:0005634~nucleus, GO:0005783~endoplasmic reticulum,GO:0005793~endoplasmic reticulum-Golgi intermediate compartment,GO:0005886~plasma membrane,GO:0016021~integral component of membrane,	GO:0004725~protein tyrosine phosphatase activity,	IPR000242:Protein-tyrosine phosphatase, receptor/non-receptor type,IPR000387:Protein-tyrosine/Dual specificity phosphatase,IPR003595:Protein-tyrosine phosphatase, catalytic,IPR012265:Protein-tyrosine phosphatase, non-receptor type-		PIRSF000926:non-receptor tyrosine-protein phosphatase, 1/2 types,	SM00194:PTPc,SM00404:PTPc_motif,

		phosphorylation of Stat1 protein,GO:0042524~negative regulation of tyrosine phosphorylation of Stat5 protein,GO:0042527~negative regulation of tyrosine phosphorylation of Stat6 protein,GO:0042593~glucose homeostasis,GO:0045650~negative regulation of macrophage differentiation,GO:0045722~positive regulation of gluconeogenesis,GO:0046627~negative regulation of insulin receptor signaling pathway,GO:0050728~negative regulation of inflammatory response,GO:0050860~negative regulation of T cell receptor signaling pathway,GO:0050922~negative regulation of chemotaxis,GO:0060336~negative regulation of interferon-gamma-mediated signaling pathway,GO:0060339~negative regulation of type I interferon-mediated signaling pathway,GO:0061099~negative regulation of protein tyrosine kinase activity,GO:0070104~negative regulation of interleukin-6-mediated signaling pathway,GO:0070373~negative regulation of ERK1 and ERK2 cascade,GO:1902202~regulation of hepatocyte growth factor receptor signaling pathway,GO:1902206~negative regulation of interleukin-2-mediated signaling pathway,GO:1902212~negative regulation of prolactin signaling pathway,GO:1902215~negative regulation of interleukin-4-mediated signaling pathway,GO:1902227~negative regulation of macrophage colony-stimulating factor			1/2,IPR016130: Protein-tyrosine phosphatase, active site,			

		signaling pathway,GO:1902233~negative regulation of positive thymic T cell selection,GO:1902237~positive regulation of endoplasmic reticulum stress-induced intrinsic apoptotic signaling pathway,GO:1903899~positive regulation of PERK-mediated unfolded protein response,GO:2000587~negative regulation of platelet-derived growth factor receptor-beta signaling pathway,						
ENSGALG00000003806	ras homolog family member A(RHOA)	GO:0007266~Rho protein signal transduction,GO:0021762~substantia nigra development,GO:0031532~actin cytoskeleton reorganization,GO:0032467~positive regulation of cytokinesis,GO:0035385~Roundabout signaling pathway,GO:0038027~apolipoprotein A-I-mediated signaling pathway,GO:0043123~positive regulation of I-kappaB kinase/NF-kappaB signaling,GO:0043149~stress fiber assembly,GO:0043297~apical junction assembly,GO:0043542~endothelial cell migration,GO:0044319~wound healing, spreading of cells,GO:0045666~positive regulation of neuron differentiation,GO:0050919~negative chemotaxis,GO:0051493~regulation of cytoskeleton organization,GO:0051496~positive regulation of stress fiber assembly,GO:0060193~positive regulation of lipase activity,GO:0060317~cardiac epithelial to mesenchymal transition,GO:0071902~positive	GO:0005768~endosome,GO:0005829~cytosol,GO:0005925~focal adhesion,GO:0005938~cell cortex,GO:0031234~extrinsic component of cytoplasmic side of plasma membrane,GO:0032154~cleavage furrow,GO:0043296~apical junction complex,GO:0070062~extracellular exosome,	GO:0003924~GTPase activity,GO:0005525~GTP binding,	IPR001806:Small GTPase superfamily,IPR005225:Small GTP-binding protein domain,IPR027417:P-loop containing nucleoside triphosphate hydrolase,	gga04144:Endocytosis,gga04270:Vascular smooth muscle contraction,gga04310:Wnt signaling pathway,gga04350:TGF-beta signaling pathway,gga04510:Focal adhesion,gga04520:Adherens junction,gga04530:Tight junction,gga04810:Regulation of actin cytoskeleton,		

		regulation of protein serine/threonine kinase activity,GO:0090051~negative regulation of cell migration involved in sprouting angiogenesis,GO:0090307~mitotic spindle assembly,GO:0097498~endothelial tube lumen extension,GO:1902766~skeletal muscle satellite cell migration,GO:1903673~mitotic cleavage furrow formation,GO:2000290~regulation of myotome development,						
ENSGALG00000011375	ring finger protein 25(RNF25)	GO:0051092~positive regulation of NF-kappaB transcription factor activity,	GO:0005634~nucleus, GO:0005829~cytosol,	GO:0008270~zinc ion binding,GO:0061630~ubiquitin protein ligase activity,	IPR001841:Zinc finger, RING-type,IPR013083:Zinc finger, RING/FYVE/PHD-type,			SM00184:RING,
ENSGALG00000004844	sema domain, transmembrane domain (TM), and cytoplasmic domain, (semaphorin) 6D(SEMA6D)	GO:0001755~neural crest cell migration,GO:0014911~positive regulation of smooth muscle cell migration,GO:0014912~negative regulation of smooth muscle cell migration,GO:0021591~ventricular system development,GO:0030335~positive regulation of cell migration,GO:0048843~negative regulation of axon extension involved in axon guidance,GO:0071526~semaphorin-plexin signaling pathway,	GO:0005887~integral component of plasma membrane,	GO:0030215~semaphorin receptor binding,GO:0045499~chemorepellent activity,	IPR001627:Semaphorin/CD100 antigen,IPR002165:Plexin,IPR015943:WD40/YVTN repeat-like-containing domain,IPR016201:Plexin-like fold,IPR027231:Semaphorin,			SM00630:Sema,
ENSGALG00000015959	small ArfGAP 1(SMAP1)	GO:0045648~positive regulation of erythrocyte differentiation,GO:2000369~regulation of clathrin-mediated endocytosis,	GO:0005737~cytoplasm,	GO:0005096~GTPase activator activity,	IPR001164:ArfGTPase activating protein,	gga04144:Endocytosis,		SM00105:ArfGap,
ENSGALG00000015794	sorting nexin 16(SNX16)	GO:0006622~protein targeting to lysosome,GO:0008333~endosome to lysosome transport,GO:0045022~early endosome to late endosome transport,	GO:0005769~early endosome,GO:0005770~late endosome,GO:0005829~cytosol,GO:0031313~extrinsic component of endosome membrane,	GO:0035091~phosphatidylinositol binding,	IPR001683:Phox homologous domain,			SM00312:PX,

ENSGALG00000007707	trafficking protein particle complex 4(TRAPPC4)	GO:0006810~transport,GO:0006888~ER to Golgi vesicle-mediated transport,GO:0016358~dendrite development,	GO:0005795~Golgi stack,GO:0008021~synaptic vesicle,GO:0030008~TRAPP complex,GO:0030425~dendrite,	GO:0017112~Rab guanyl-nucleotide exchange factor activity,	IPR007233:Sybindin-like protein,IPR011012:Longin-like domain,			SM01399:SM01399,
ENSGALG00000004254	transmembrane protein 120B(TMEM120B)	GO:0045444~fat cell differentiation,GO:0051291~protein heterooligomerization,	GO:0005637~nuclear inner membrane,GO:0016021~integral component of membrane,		IPR012926:TMPIT-like,			

Supplementary Table 13: Functional annotations and pathways associated with identified Cecal DEGs (low vs. high)

Annotation table for up-regulated genes in low FCR

ID	Gene Name	GOTERM_BP	GOTERM_CC	GOTERM_MF	INTERPRO	KEGG PATHWAY	PIR SUPERFAMILY	SMART
ENSGALG00000009955	ATP binding cassette subfamily G member 5(ABCG5)	GO:0007588~excretion,GO:0010949~negative regulation of intestinal phytosterol absorption,GO:0033344~cholesterol efflux,GO:0042632~cholesterol homeostasis,GO:0045796~negative regulation of intestinal cholesterol absorption,	GO:0016021~integral component of membrane,GO:0016324~apical plasma membrane,GO:0043190~ATP-binding cassette (ABC) transporter complex,GO:0043235~receptor complex,	GO:0005524~ATP binding,GO:0017127~cholesterol transporter activity,GO:0042626~ATPase activity, coupled to transmembrane movement of substances,	IPR003439:ABC transporter-like,IPR003593:AAA+ ATPase domain,IPR013525:ABC-2 type transporter,IPR017871:ABC transporter, conserved site,IPR027417:P-loop containing nucleoside triphosphate hydrolase,	gga02010:ABC transporters,		SM00382:AAA,
ENSGALG00000009433	BCL2 associated athanogene 3(BAG3)	GO:0008625~extrinsic apoptotic signaling pathway via death domain receptors,GO:0010664~negative regulation of striated muscle cell apoptotic process,GO:0050821~protein stabilization,GO:0071260~cellular response to mechanical stimulus,GO:0097192~extrinsic apoptotic signaling pathway in absence of ligand,	GO:0005829~cytosol,GO:0005886~plasma membrane,GO:0030018~Z disc,	GO:0000774~adenyl-nucleotide exchange factor activity,	IPR001202:WW domain,IPR003103:BAG domain,			SM00264:BAG,SM00456:WW,
ENSGALG00000005001	CCHC-type zinc finger, nucleic acid binding protein(CNBP)	GO:0006351~transcription, DNA-templated,GO:0006355~regulation of transcription, DNA-templated,GO:0008284~positive regulation of cell proliferation,GO:0045944~positive regulation of transcription from	GO:0005634~nucleus,GO:0005783~endoplasmic reticulum,GO:0005829~cytosol,	GO:0003677~DNA binding,GO:0008270~zinc ion binding,GO:0044822~poly(A) RNA binding,	IPR001878:Zinc finger, CCHC-type,			SM00343:ZnF_C2HC,

		RNA polymerase II promoter,						
ENSGALG00000016720	CD2-associated protein(CD2AP)	GO:0032911~negative regulation of transforming growth factor beta1 production,GO:1900182~positive regulation of protein localization to nucleus,GO:2000249~regulation of actin cytoskeleton reorganization,	GO:0001726~ruffle,GO:0005730~nucleolus,GO:0005737~cytoplasm,GO:0005886~plasma membrane,GO:0005911~cell-cell junction,GO:0031941~filamentous actin,GO:0070062~extracellular exosome,		IPR001452:Src homology-3 domain,			SM00326:SH3,
ENSGALG00000012247	DEAD (Asp-Glu-Ala-Asp) box helicase 17(DDX17)	GO:0010501~RNA secondary structure unwinding,GO:0045893~positive regulation of transcription, DNA-templated,		GO:0003676~nucleic acid binding,GO:0004004~ATP-dependent RNA helicase activity,GO:0005524~ATP binding,	IPR000629:RNA helicase, ATP-dependent, DEAD-box, conserved site,IPR001650:Helicase, C-terminal,IPR011545:DNA/RNA helicase, DEAD/DEAH box type, N-terminal,IPR014001:Helicase, superfamily 1/2, ATP-binding domain,IPR014014:RNA helicase, DEAD-box type, Q motif,IPR027417:P-loop containing nucleoside triphosphate hydrolase,			SM00487:DEXDc,SM00490:HELICc,
ENSGALG00000016231	DEAD (Asp-Glu-Ala-Asp) box polypeptide 3, X-linked(DDX3X)	GO:0006413~translational initiation,GO:0007059~chromosome segregation,GO:0010468~regulation of gene expression,GO:0010501~RNA secondary structure unwinding,	GO:0036464~cytoplasmic ribonucleoprotein granule,	GO:0003676~nucleic acid binding,GO:0004004~ATP-dependent RNA helicase activity,GO:0004386~helicase activity,GO:0005524~ATP binding,	IPR000629:RNA helicase, ATP-dependent, DEAD-box, conserved site,IPR001650:Helicase, C-terminal,IPR011545:DNA/RNA helicase, DEAD/DEAH box type, N-terminal,IPR014001:Helicase, superfamily 1/2, ATP-binding domain,IPR014014:RNA helicase, DEAD-	gga04622:RIG-I-like receptor signaling pathway,		SM00487:DEXDc,SM00490:HELICc,

					box type, Q motif,IPR027417:P-loop containing nucleoside triphosphate hydrolase,			
ENSGALG00000010764	F-box protein 8(FBXO8)	GO:0006511~ubiquitin-dependent protein catabolic process,GO:0032012~regulation of ARF protein signal transduction,	GO:0000151~ubiquitin ligase complex,	GO:0005086~ARF guanyl-nucleotide exchange factor activity,	IPR000904:SEC7-like,IPR001810:F-box domain, cyclin-like,IPR023394:SEC7-like, alpha orthogonal bundle,			SM00222:Sec7,
ENSGALG00000010276	GC-rich promoter binding protein 1 like 1(GPBP1L1)	GO:0006351~transcription, DNA-templated,GO:0006355~regulation of transcription, DNA-templated,GO:0045893~positive regulation of transcription, DNA-templated,	GO:0005634~nucleus,	GO:0003700~transcription factor activity, sequence-specific DNA binding,				
ENSGALG00000003381	HECT and RLD domain containing E3 ubiquitin protein ligase family member 1(HERC1)	GO:0010507~negative regulation of autophagy,GO:0021702~cerebellar Purkinje cell differentiation,GO:0031175~neuron projection development,GO:0050885~neuromuscular process controlling balance,		GO:0004842~ubiquitin-protein transferase activity,GO:0016874~ligase activity,	IPR000408:Regulator of chromosome condensation, RCC1,IPR000569:HECT,IPR001680:WD40 repeat,IPR001870:B30.2/SPRY domain,IPR003877:SPla/RYanodine receptor SPRY,IPR009091:Regulator of chromosome condensation 1/beta-lactamase-inhibitor protein II,IPR013320:Concanavalin A-like lectin/glucanase, subgroup,IPR015943:WD40/YVTN repeat-like-containing domain,IPR017986:WD40-repeat-containing domain,IPR019775:	gga04120:Ubiquitin mediated proteolysis,		SM00119:HECTc,SM00320:WD40,SM00449:SPRY,

					WD40 repeat, conserved site,			
ENSGALG00000001359	NAD kinase(NADK)	GO:0006741~NADP biosynthetic process,GO:0019674~NAD metabolic process,		GO:0003951~NAD+ kinase activity,	IPR002504:Inorganic polyphosphate/ATP-NAD kinase, predicted,IPR016064:ATP-NAD kinase-like domain,IPR017437:ATP-NAD kinase, PpnK-type, all-beta,IPR017438:Inorganic polyphosphate/ATP-NAD kinase, domain 1,	gga00760:Nicotinate and nicotinamide metabolism,gga01100:Metabolic pathways,		
ENSGALG00000006524	NOC3 like DNA replication regulator(NOC3L)	GO:0045444~fat cell differentiation,	GO:0005634~nucleus,GO:0005730~nucleolus,GO:0016607~nuclear speck,	GO:0003682~chromatin binding,GO:0044822~poly(A) RNA binding,	IPR005612:CCAAT-binding factor,IPR011501:Nucleolar complex-associated,IPR016024:Armadillo-type fold,IPR016903:Nucleolar complex-associated protein 3,		PIRSF028977:nucleolar complex-associated protein 3,	
ENSGALG00000005425	O-linked N-acetylglucosamine (GlcNAc) transferase(OGT)				IPR011990:Tetratricopeptide-like helical,IPR013026:Tetratricopeptide repeat-containing domain,IPR019734:Tetratricopeptide repeat,	gga00514:Other types of O-glycan biosynthesis,gga04931:Insulin resistance,		SM00028:TPR,
ENSGALG00000015105	PC4 and SFRS1 interacting protein 1(PSIP1)	GO:0000395~mRNA 5'-splice site recognition,GO:0006351~transcription, DNA-templated,GO:0006979~response to oxidative stress,GO:0009408~response to heat,GO:0045944~positive regulation of transcription from RNA polymerase II promoter,	GO:0005634~nucleus,GO:0005654~nucleoplasm,GO:0005720~nuclear heterochromatin,GO:0035327~transcriptionally active chromatin,	GO:0001105~RNA polymerase II transcription coactivator activity,GO:0003682~chromatin binding,GO:0033613~activating transcription factor binding,GO:0044822~poly(A) RNA binding,GO:0097100~supercoiled DNA binding,	IPR000313:PWWP,IPR021567:Lens epithelium-derived growth factor (LEDGF),			SM00293:PWWP,

ENSGALG00000016340	Putative eukaryotic translation initiation factor 2 subunit 3-like protein(EIF2S3L)	GO:0001731~formation of translation preinitiation complex,	GO:0005622~intracellular,	GO:0003743~translation initiation factor activity,GO:0003924~GTPase activity,GO:0005525~GTP binding,	IPR000795:Elongation factor, GTP-binding domain,IPR004161:Translation elongation factor EFTu/EF1A, domain 2,IPR009000:Translation elongation/initiation factor/Ribosomal, beta-barrel,IPR009001:Translation elongation factor EF1A/initiation factor IF2gamma, C-terminal,IPR015256:Translation initiation factor 2, gamma subunit, C-terminal,IPR027417:P-loop containing nucleoside triphosphate hydrolase,	gga03013:RNA transport,		
ENSGALG00000009842	Ras association domain family member 3(RASSF3)	GO:0007165~signal transduction,GO:0042981~regulation of apoptotic process,	GO:0005737~cytoplasm,GO:0005886~plasma membrane,		IPR000159:Ras-association,IPR011524:SARAH domain,			SM00314:RA,
ENSGALG00000007128	SH3 domain binding glutamate rich protein like(SH3BGRL)		GO:0005615~extracellular space,GO:0070062~extracellular exosome,		IPR006993:SH3-binding, glutamic acid-rich protein,IPR012336:Thioredoxin-like fold,		PIRSF008142:SH3 domain-binding glutamic acid-rich-like protein,	
ENSGALG00000009677	Sjogren syndrome antigen B(SSB)	GO:0006396~RNA processing,GO:0075522~IRES-dependent viral translational initiation,GO:1903608~protein localization to cytoplasmic stress granule,	GO:0000784~nuclear chromosome, telomeric region,GO:0030529~intracellular ribonucleoprotein complex,	GO:0000166~nucleotide binding,GO:0044822~poly(A) RNA binding,	IPR000504:RNA recognition motif domain,IPR002344:Lupus La protein,IPR006630:RNA-binding protein Lupus La,IPR011991:Winged helix-turn-helix DNA-binding domain,IPR012677:Nucleotide-binding, alpha-beta plait,IPR014886:RNA-binding motif,			SM00360:RRM, SM00715:LA,

ENSGALG00000014709	Ski2 like RNA helicase 2(SKIV2L2)	GO:0006401~RNA catabolic process,		GO:0003723~RNA binding,GO:0003724~RNA helicase activity,GO:0005524~ATP binding,	IPR001650:Helicase, C-terminal,IPR011545:DNA/RNA helicase, DEAD/DEAH box type, N-terminal,IPR012961:DSH, C-terminal,IPR014001:Helicase, superfamily 1/2, ATP-binding domain,IPR016438:RNA helicase, ATP-dependent, SK12/DOB1,IPR025696:rRNA-processing arch domain,IPR027417:P-loop containing nucleoside triphosphate hydrolase,	gga03018:RNA degradation,	PIRSF005198:antiviral helicase, SKI2 type,	SM00487:DEXDc,SM00490:HELICc,SM01142:SM01142,
ENSGALG00000016337	TatD DNase domain containing 1(TATDN1)	GO:0006308~DNA catabolic process,	GO:0005654~nucleoplasm,	GO:0004536~deoxyribonuclease activity,GO:0016888~endodeoxyribonuclease activity, producing 5'-phosphomonoesters,	IPR001130:TatD family,IPR018228:Deoxyribonuclease, TatD-related, conserved site,		PIRSF005902:Mg-dependent DNase, TatD type,	
ENSGALG00000013135	UDP-N-acetyl-alpha-D-galactosamine:polypeptide N-acetylgalactosaminyltransferase 1 (GalNAc-T1)(GALNT1)	GO:0006493~protein O-linked glycosylation,GO:0018242~protein O-linked glycosylation via serine,GO:0018243~protein O-linked glycosylation via threonine,	GO:0000139~Golgi membrane,GO:0016021~integral component of membrane,GO:0048471~perinuclear region of cytoplasm,	GO:0004653~polypeptide N-acetylgalactosaminyltransferase activity,GO:0030145~manganese ion binding,GO:0030246~carbohydrate binding,	IPR000772:Ricin B lectin domain,IPR001173:Glycosyl transferase, family 2,	gga00512:Mucin type O-Glycan biosynthesis,gga01100:Metabolic pathways,		SM00458:RICIN,
ENSGALG00000023648	WD repeat-containing protein 92(HZGJ)			GO:0005509~calcium ion binding,	IPR002048:EF-hand domain,IPR011992:EF-hand-like domain,IPR018247:EF-Hand 1, calcium-binding site,	gga04010:MAPK signaling pathway,gga04020:Calcium signaling pathway,gga04114:Oocyte meiosis,gga04310:Wnt signaling pathway,gga04370:VEGF		SM00054:EFh,

						signaling pathway,		
ENSGALG00000013464	acidic (leucine-rich) nuclear phosphoprotein 32 family, member E(ANP32E)	GO:0006334~nucleosome assembly,GO:0006913~nucleocytoplasmic transport,GO:0016569~covalent chromatin modification,GO:0042981~regulation of apoptotic process,GO:0043086~negative regulation of catalytic activity,GO:0043486~histone exchange,	GO:0000812~Swr1 complex,GO:0005634~nucleus,GO:0005737~cytoplasm,GO:0016023~cytoplasmic, membrane-bounded vesicle,GO:0031410~cytoplasmic vesicle,	GO:0019212~phosphatase inhibitor activity,GO:0042393~histone binding,	IPR001611:Leucine-rich repeat,			
ENSGALG00000004179	anaphase promoting complex subunit 5(ANAPC5)	GO:0007067~mitotic nuclear division,GO:0051301~cell division,GO:0070979~protein K11-linked ubiquitination,	GO:0005680~anaphase-promoting complex,		IPR011990:Tetratricopeptide-like helical,IPR026000:Anaphase-promoting complex subunit 5,TPR-containing domain,	gga04110:Cell cycle,gga04114:Oocyte meiosis,gga04120:Ubiquitin mediated proteolysis,gga04914:Progesterone-mediated oocyte maturation,		
ENSGALG00000013071	caldesmon 1(CALD1)	GO:0006936~muscle contraction,GO:1903611~negative regulation of calcium-dependent ATPase activity,	GO:0005623~cell,GO:0005856~cytoskeleton,GO:0005886~plasma membrane,GO:0030016~myofibril,GO:0030478~actin cap,	GO:0003779~actin binding,GO:0005516~calmodulin binding,GO:0044548~S100 protein binding,GO:0045159~myosin II binding,	IPR006017:Caldesmon,IPR006018:Caldesmon/lymphocyte specific protein,	gga04270:Vascular smooth muscle contraction,		
ENSGALG00000005945	calnexin(CANX)	GO:0006457~protein folding,GO:0048488~synaptic vesicle endocytosis,	GO:0005783~endoplasmic reticulum,GO:0005789~endoplasmic reticulum membrane,GO:0016021~integral component of membrane,GO:0043209~myelin sheath,GO:0044233~ER-mitochondrion membrane contact site,GO:0045202~synapse,GO:0070062~extracellular exosome,	GO:0005509~calcium ion binding,GO:0044822~poly(A) RNA binding,	IPR001580:Calreticulin/calnexin,IPR009033:Calreticulin/calnexin, P domain,IPR013320:Concanavalin A-like lectin/glucanase, subgroup,IPR018124:Calreticulin/calnexin, conserved site,	gga04141:Protein processing in endoplasmic reticulum,gga04145:Phagosome,		

ENSGALG00000001382	cell division cycle 2-like 1 (PITSLRE proteins)(CDC2L1)	GO:0007088~regulation of mitotic nuclear division,GO:2001234~negative regulation of apoptotic signaling pathway,	GO:0005634~nucleus,	GO:0004672~protein kinase activity,GO:0005524~ATP binding,	IPR000719:Protein kinase, catalytic domain,IPR008271:Serine/threonine-protein kinase, active site,IPR011009:Protein kinase-like domain,			SM00220:S_TKc,
ENSGALG00000014448	cell division cycle 37 like 1(CDC37L1)	GO:0006457~protein folding,GO:0050821~protein stabilization,	GO:0005737~cytoplasm,	GO:0031072~heat shock protein binding,GO:0051082~unfolded protein binding,GO:0051087~chaperone binding,	IPR004918:Cdc37,IPR013874:Cdc37, Hsp90 binding,			SM01070:SM01070,
ENSGALG00000004108	cell division cycle and apoptosis regulator 1(CCAR1)	GO:0006355~regulation of transcription, DNA-templated,GO:0008284~positive regulation of cell proliferation,GO:0030335~positive regulation of cell migration,		GO:0001047~core promoter binding,GO:0003714~transcription corepressor activity,GO:0030374~ligand-dependent nuclear receptor transcription coactivator activity,GO:0044822~poly(A) RNA binding,	IPR003034:SAP domain,IPR011992:EF-hand-like domain,IPR012340:Nucleic acid-binding, OB-fold,IPR025223:S1-like RNA binding domain,IPR025224:DBC1/CARP1,IPR025954:DBC1/CARP1 catalytically inactive NUDIX hydrolase domain,			SM00513:SAP,SM01122:SM01122,
ENSGALG00000016796	chromosome 1 open reading frame, human C2orf49(C1H2ORF49)	GO:0009952~anterior/posterior pattern specification,GO:0042981~regulation of apoptotic process,GO:0048598~embryonic morphogenesis,GO:0060027~convergent extension involved in gastrulation,GO:0060062~Spemann organizer formation at the dorsal lip of the blastopore,	GO:0072669~tRNA-splicing ligase complex,		IPR024887:Ashwin,			
ENSGALG00000019232	cilia and flagella associated protein 44(CFAP44)				IPR015943:WD40/YVTN repeat-like-containing domain,IPR017986:WD40-repeat-containing domain,			

ENSGALG00000008266	collagen, type IV, alpha 6(COL4A6)	GO:0030198~extracellular matrix organization,GO:0071230~cellular response to amino acid stimulus,	GO:0005587~collagen type IV trimer,	GO:0005201~extracellular matrix structural constituent,	IPR001442:Collagen IV, non-collagenous,IPR008160:Collagen triple helix repeat,IPR016187:C-type lectin fold,	gga04510:Focal adhesion,gga04512:ECM-receptor interaction,		SM00111:C4,
ENSGALG00000016289	dystonin(DST)							
ENSGALG00000017133	eukaryotic translation elongation factor 1 alpha lysine methyltransferase 1(EEF1AKMT1)	GO:0018022~peptidyl-lysine methylation,	GO:0005737~cytoplasm,GO:0070062~extracellular exosome,	GO:0003676~nucleic acid binding,GO:0016279~protein-lysine N-methyltransferase activity,	IPR002052:DNA methylase, N-6 adenine-specific, conserved site,IPR019369:DNA methylase, N-6 adenine-specific, eukaryotic,			
ENSGALG00000010560	eukaryotic translation initiation factor 2 alpha kinase 2(EIF2AK2)	GO:0000186~activation of MAPKK activity,GO:0008285~negative regulation of cell proliferation,GO:0009615~response to virus,GO:0009636~response to toxic substance,GO:0010998~regulation of translational initiation by eIF2 alpha phosphorylation,GO:0017148~negative regulation of translation,GO:0033689~negative regulation of osteoblast proliferation,GO:0034198~cellular response to amino acid starvation,GO:0035455~response to interferon-alpha,GO:0042981~regulation of apoptotic process,GO:0045071~negative regulation of viral genome replication,GO:0045087~innate immune response,GO:0046777	GO:0016020~membrane,GO:0048471~perinuclear region of cytoplasm,	GO:0003725~double-stranded RNA binding,GO:0004674~protein serine/threonine kinase activity,GO:0004694~eukaryotic translation initiation factor 2alpha kinase activity,GO:0005524~ATP binding,GO:0044822~poly(A) RNA binding,	IPR000719:Protein kinase, catalytic domain,IPR008271:Serine/threonine-protein kinase, active site,IPR011009:Protein kinase-like domain,IPR014720:Double-stranded RNA-binding-like domain,IPR017441:Protein kinase, ATP binding site,	gga04141:Protein processing in endoplasmic reticulum,gga05164:Influenza A,gga05168:Herpes simplex infection,		SM00220:S_TKc,SM00358:DSRM,

		~protein autophosphorylation,GO:0051092~positive regulation of NF-kappaB transcription factor activity,						
ENSGALG00000009611	eukaryotic translation initiation factor 2 alpha kinase 4(EIF2AK4)	GO:0036492~eiF2alpha phosphorylation in response to endoplasmic reticulum stress,GO:0060733~regulation of eIF2 alpha phosphorylation by amino acid starvation,GO:0070417~cellular response to cold,		GO:0004694~eukaryotic translation initiation factor 2alpha kinase activity,GO:0005524~ATP binding,	IPR000719:Protein kinase, catalytic domain,IPR004154:Anticodon-binding,IPR006575:RWD domain,IPR008271:Serine/threonine-protein kinase, active site,IPR011009:Protein kinase-like domain,IPR016135:Ubiquitin-conjugating enzyme/RWD-like,IPR017441:Protein kinase, ATP binding site,IPR024435:Histidyl tRNA synthetase-related domain,	gga04141:Protein processing in endoplasmic reticulum,gga05164:Influenza A,gga05168:Herpes simplex infection,		SM00220:S_TKc,SM00591:RWD,
ENSGALG00000008187	eukaryotic translation initiation factor 3 subunit J(EIF3J)	GO:0001731~formation of translation preinitiation complex,GO:0002181~cytoplasmic translation,GO:0006446~regulation of translational initiation,	GO:0005852~eukaryotic translation initiation factor 3 complex,GO:0016282~eukaryotic 43S preinitiation complex,GO:0033290~eukaryotic 48S preinitiation complex,	GO:0003743~translation initiation factor activity,	IPR013906:Eukaryotic translation initiation factor 3 subunit J,IPR023194:Eukaryotic translation initiation factor 3-like domain,	gga03013:RNA transport,		
ENSGALG00000008684	eukaryotic translation initiation factor 4A2(EIF4A2)	GO:0006413~translational initiation,GO:0006446~regulation of translational initiation,GO:0010468~regulation of gene expression,GO:0010501~RNA secondary structure unwinding,GO:1900260~negative regulation of RNA-directed RNA polymerase activity,	GO:0048471~perinuclear region of cytoplasm,	GO:0003743~translation initiation factor activity,GO:0004004~ATP-dependent RNA helicase activity,GO:0005524~ATP binding,GO:0044822~poly(A) RNA binding,	IPR000629:RNA helicase, ATP-dependent, DEAD-box, conserved site,IPR001650:Helicase, C-terminal,IPR011545:DNA/RNA helicase, DEAD/DEAH box type, N-terminal,IPR014001:Helicase, superfamily 1/2, ATP-binding domain,IPR014014:R	gga03013:RNA transport,		SM00487:DEXDc,SM00490:HELICc,

					NA helicase, DEAD-box type, Q motif,IPR027417:P-loop containing nucleoside triphosphate hydrolase,			
ENSGALG00000019524	family with sequence similarity 206 member A(FAM206A)		GO:0016607~nuclear speck,GO:0030425~dendrite,		IPR011053:Single hybrid motif,			
ENSGALG00000014210	glucosamine-6-phosphate deaminase 2(GNPDA2)	GO:0005975~carbohydrate metabolic process,GO:0006044~N-acetylglucosamine metabolic process,GO:0006046~N-acetylglucosamine catabolic process,GO:0006048~UDP-N-acetylglucosamine biosynthetic process,GO:0019262~N-acetylneuraminate catabolic process,	GO:0005634~nucleus,GO:0005737~cytoplasm,	GO:0004342~glucosamine-6-phosphate deaminase activity,GO:0016787~hydrolase activity,	IPR004547:Glucosamine-6-phosphate isomerase,IPR006148:Glucosamine/galactosamine-6-phosphate isomerase,IPR018321:Glucosamine-6-phosphate isomerase, conserved site,	gga00520:Amino sugar and nucleotide sugar metabolism,gga01100:Metabolic pathways,		
ENSGALG00000008094	heat shock protein family D (Hsp60) member 1(HSPD1)	GO:0002368~B cell cytokine production,GO:0002755~MyD88-dependent toll-like receptor signaling pathway,GO:0002842~positive regulation of T cell mediated immune response to tumor cell,GO:0006919~activation of cysteine-type endopeptidase activity involved in apoptotic process,GO:0006986~response to unfolded protein,GO:0009409~response to cold,GO:0032727~positive regulation of interferon-alpha production,GO:0032729~positive regulation	GO:0005615~extracellular space,GO:0005737~cytoplasm,GO:0005743~mitochondrial inner membrane,GO:0005759~mitochondrial matrix,GO:0005769~early endosome,GO:0005829~cytosol,GO:0005886~plasma membrane,GO:0005905~clathrin-coated pit,GO:0009986~cell surface,GO:0019907~cyclin-dependent protein kinase activating kinase holoenzyme complex,GO:0030135~coated vesicle,GO:0030141~secretory granule,GO:0031012~e	GO:0001530~lipopolysaccharide binding,GO:0002039~p53 binding,GO:0003725~double-stranded RNA binding,GO:0005515~protein binding,GO:0005524~ATP binding,GO:0031625~ubiquitin protein ligase binding,GO:0044822~poly(A) RNA binding,GO:0051087~chaperone binding,	IPR001844:Chaperonin Cpn60,IPR002423:Chaperonin Cpn60/TCP-1,IPR018370:Chaperonin Cpn60, conserved site,IPR027409:GroEL-like apical domain,IPR027413:GroEL-like equatorial domain,	gga03018:RNA degradation,		

		of interferon-gamma production,GO:0032733~positive regulation of interleukin-10 production,GO:0032735~positive regulation of interleukin-12 production,GO:0032755~positive regulation of interleukin-6 production,GO:0042026~protein refolding,GO:0042100~B cell proliferation,GO:0042110~T cell activation,GO:0043032~positive regulation of macrophage activation,GO:0043066~negative regulation of apoptotic process,GO:0048291~isotype switching to IgG isotypes,GO:0050821~protein stabilization,GO:0050870~positive regulation of T cell activation,	xtracellular matrix,GO:0043025~neuronal cell body,GO:0043209~myelin sheath,GO:0046696~lipopolysaccharide receptor complex,GO:0070062~extracellular exosome,					
ENSGALG00000014381	heterogeneous nuclear ribonucleoprotein A/B(HNRNPAB)			GO:0000166~nucleotide binding,GO:0003676~nucleic acid binding,	IPR000504:RNA recognition motif domain,IPR012677:Nucleotide-binding, alpha-beta plait,IPR012956:CARG-binding factor, N-terminal,			SM00360:RRM,
ENSGALG00000004428	histamine H3 receptor-like(LOC427545)	GO:0001505~regulation of neurotransmitter levels,GO:0007197~adenylate cyclase-inhibiting G-protein coupled acetylcholine receptor signaling pathway,GO:0007207~phospholipase C-activating G-protein coupled acetylcholine	GO:0016021~integral component of membrane,GO:0045202~synapse,	GO:0004969~histamine receptor activity,GO:0016907~G-protein coupled acetylcholine receptor activity,	IPR000276:G protein-coupled receptor, rhodopsin-like,IPR003980:Histamine H3 receptor,IPR017452:GPCR, rhodopsin-like, 7TM,	gga04080:Neuroactive ligand-receptor interaction,		

		receptor signaling pathway,GO:0007271~synaptic transmission, cholinergic,						
ENSGALG00000007917	laminin, beta 4(LAMB4)					gga04510:Focal adhesion,gga04512:ECM-receptor interaction,		
ENSGALG00000009402	leucine rich repeat containing 34(LRRC34)				IPR001611:Leucine-rich repeat,			
ENSGALG00000015804	listerin E3 ubiquitin protein ligase 1(LTN1)	GO:0051865~protein autoubiquitination,		GO:0008270~zinc ion binding,GO:0016874~ligase activity,GO:0061630~ubiquitin protein ligase activity,	IPR001841:Zinc finger, RING-type,IPR011016:Zinc finger, RING-CH-type,IPR013083:Zinc finger, RING/FYVE/PHD-type,IPR016024:Armadillo-type fold,			SM00744:RINGv,
ENSGALG00000006005	mediator complex subunit 31(MED31)	GO:0006357~regulation of transcription from RNA polymerase II promoter,GO:0048147~negative regulation of fibroblast proliferation,GO:0060173~limb development,	GO:0016592~mediator complex,GO:0070847~core mediator complex,	GO:0001104~RNA polymerase II transcription cofactor activity,GO:0003713~transcription coactivator activity,	IPR008831:Mediator complex, subunit Med31,			
ENSGALG00000011806	mucin 13, cell surface associated(MUC13)		GO:0016021~integral component of membrane,					
ENSGALG00000011708	myosin light chain kinase(MYLK)	GO:0002230~positive regulation of defense response to virus by host,GO:0006468~protein phosphorylation,GO:0006939~smooth muscle contraction,GO:0030335~positive regulation of cell migration,GO:0032060~bleb assembly,GO:0051928~positive regulation of calcium ion	GO:0001725~stress fiber,GO:0005737~cytoplasm,GO:0005829~cytosol,GO:0016020~membrane,GO:0030027~lamellipodium,GO:0032154~cleavage furrow,GO:0070062~extracellular exosome,	GO:0004672~protein kinase activity,GO:0004687~myosin light chain kinase activity,GO:0005516~calmodulin binding,GO:0005524~ATP binding,GO:0046872~metal ion binding,	IPR000719:Protein kinase, catalytic domain,IPR003598:Immunoglobulin subtype 2,IPR003599:Immunoglobulin subtype,IPR003961:Fibronectin, type III,IPR007110:Immunoglobulin-like domain,IPR008271:Serine/threonine-protein kinase, active site,IPR011009:Protein kinase-like	gga04020:Calcium signaling pathway,gga04270:Vascular smooth muscle contraction,gga04510:Focal adhesion,gga04810:Regulation of actin cytoskeleton,		SM00060:FN3,SM00220:S_TKc,SM00408:IGc2,SM00409:IG,

		transport,GO:0060414~aorta smooth muscle tissue morphogenesis,GO:0071476~cellular hypotonic response,GO:0090303~positive regulation of wound healing,GO:0098779~mitophagy in response to mitochondrial depolarization,GO:0098792~xenophagy,			domain,IPR013098:Immunoglobulin I-set,IPR013783:Immunoglobulin-like fold,IPR017441:Protein kinase, ATP binding site,			
ENSGALG00000006520	myosin, heavy chain 11, smooth muscle(MYH11)	GO:0006939~smooth muscle contraction,GO:0030241~skeletal muscle myosin thick filament assembly,GO:0048251~elastic fiber assembly,GO:0048739~cardiac muscle fiber development,GO:0098779~mitophagy in response to mitochondrial depolarization,	GO:0016459~myosin complex,GO:0030016~myofibril,GO:0032982~myosin filament,GO:0070062~extracellular exosome,	GO:0003774~motor activity,GO:0003779~actin binding,GO:0005515~protein binding,GO:0005516~calmodulin binding,GO:0005524~ATP binding,GO:0008307~structural constituent of muscle,	IPR000048:IQ motif, EF-hand binding site,IPR001609:Myosin head, motor domain,IPR002928:Myosin tail,IPR004009:Myosin, N-terminal, SH3-like,IPR027401:Myosin-like IQ motif-containing domain,IPR027417:P-loop containing nucleoside triphosphate hydrolase,	gga04530:Tight junction,		SM00015:IQ,SM00242:MYSc,
ENSGALG00000008945	nexilin (F actin binding protein)(NEXN)	GO:0030334~regulation of cell migration,GO:0048739~cardiac muscle fiber development,GO:0051493~regulation of cytoskeleton organization,	GO:0005925~focal adhesion,GO:0030018~Z disc,	GO:0008307~structural constituent of muscle,GO:0051015~actin filament binding,	IPR003599:Immunoglobulin subtype,IPR007110:Immunoglobulin-like domain,IPR013098:Immunoglobulin I-set,IPR013783:Immunoglobulin-like fold,			SM00409:IG,
ENSGALG00000001603	nuclear prelamin A recognition factor(NARF)		GO:0005638~lamin filament,		IPR003149:Iron hydrogenase, small subunit-like,IPR004108:Iron hydrogenase, large subunit, C-terminal,IPR009016:Iron hydrogenase,			SM00902:SM00902,
ENSGALG00000002197	nucleophosmin (nucleolar phosphoprotein	GO:0006281~DNA repair,GO:0006334~nucleosome assembly,GO:0006913	GO:0005634~nucleus,GO:0005654~nucleoplasm,GO:0005730~nucleolus,GO:0005737~cytopla	GO:0003713~transcription coactivator activity,GO:0003723~RNA	IPR004301:Nucleoplasmin,IPR024057:Nucleoplasmin core domain,			

	B23, numatrin)(NPM1)	~nucleocytoplasmic transport,GO:0007098~centrosome cycle,GO:0007569~cell aging,GO:0008104~protein localization,GO:0008284~positive regulation of cell proliferation,GO:0008285~negative regulation of cell proliferation,GO:0010826~negative regulation of centrosome duplication,GO:0032071~regulation of endodeoxyribonuclease activity,GO:0043066~negative regulation of apoptotic process,GO:0044387~negative regulation of protein kinase activity by regulation of protein phosphorylation,GO:0045727~positive regulation of translation,GO:0045893~positive regulation of transcription, DNA-templated,GO:0046599~regulation of centriole replication,GO:0051092~positive regulation of NF-kappaB transcription factor activity,GO:0051259~protein oligomerization,GO:0060699~regulation of endoribonuclease activity,GO:0060735~regulation of eIF2 alpha phosphorylation	sm,GO:0005813~centrosome,GO:0005925~focal adhesion,GO:0016020~membrane,GO:0030529~intracellular ribonucleoprotein complex,GO:0031616~spindle pole centrosome,	binding,GO:0004860~protein kinase inhibitor activity,GO:0043023~ribosomal large subunit binding,GO:0043024~ribosomal small subunit binding,GO:0044822~poly(A) RNA binding,GO:0051059~NF-kappaB binding,GO:0051082~unfolded protein binding,				

		by dsRNA,GO:1902751~positive regulation of cell cycle G2/M phase transition,						
ENSGALG00000012720	nucleoporin 153kDa(NUP153)	GO:0006405~RNA export from nucleus,GO:0006606~protein import into nucleus,GO:0046832~negative regulation of RNA export from nucleus,GO:0051292~nuclear pore complex assembly,	GO:0005730~nucleolus,GO:0005737~cytoplasm,GO:0031965~nuclear membrane,GO:0034399~nuclear periphery,GO:0042405~nuclear inclusion body,GO:0044613~nuclear pore central transport channel,GO:0044615~nuclear pore nuclear basket,	GO:0005487~nucleocytoplasmic transporter activity,GO:0008139~nuclear localization sequence binding,GO:0008270~zinc ion binding,GO:0017056~structural constituent of nuclear pore,	IPR001876:Zinc finger, RanBP2-type,IPR013913:Nucleoporin, Nup153-like,IPR018892:Retro-transposon transporting, conserved site,	gga03013:RNA transport,		SM00547:ZnF_RBZ,
ENSGALG00000009300	numb homolog (Drosophila)(NUMB)	GO:0007409~axonogenesis,GO:0021670~lateral ventricle development,GO:0021849~neuroblast division in subventricular zone,GO:0030335~positive regulation of cell migration,GO:0030862~positive regulation of polarized epithelial cell differentiation,GO:0034332~adherens junction organization,GO:0045746~negative regulation of Notch signaling pathway,GO:0050769~positive regulation of neurogenesis,GO:0060487~lung epithelial cell differentiation,GO:1903077~negative regulation of protein localization to plasma membrane,	GO:0005634~nucleus,GO:0005769~early endosome,GO:0005925~focal adhesion,GO:0016323~basolateral plasma membrane,GO:0019897~extrinsic component of plasma membrane,GO:0030136~clathrin-coated vesicle,GO:0045177~apical part of cell,		IPR006020:Phosphotyrosine interaction domain,IPR010449:NUMB domain,IPR011993:Pleckstrin homology-like domain,IPR016698:Numb/numb-like,	gga04330:Notch signaling pathway,	PIRSF017607:numb/numb-like protein,	SM00462:PTB,
ENSGALG00000001832	phytanoyl-CoA 2-hydroxylase		GO:0005739~mitochondrion,		IPR003961:Fibronectin, type			

	interacting protein-like(PHYHIPL)				III,IPR013783:Immunoglobulin-like fold,			
ENSGALG00000012077	poly(ADP-ribose) polymerase family member 9(PARP9)	GO:0006302~double-strand break repair,GO:0060330~regulation of response to interferon-gamma,	GO:0005654~nucleoplasm,GO:0005739~mitochondrion,GO:0016020~membrane,	GO:0003950~NAD+ ADP-ribosyltransferase activity,	IPR002589:Appr-1-p processing,IPR012317:Poly(ADP-ribose) polymerase, catalytic domain,			SM00506:A1pp,
ENSGALG00000011387	polymerase (RNA) II (DNA directed) polypeptide B, 140kDa(POLR2B)	GO:0006351~transcription, DNA-templated,GO:0006366~transcription from RNA polymerase II promoter,	GO:0000784~nuclear chromosome, telomeric region,GO:0005665~DNA-directed RNA polymerase II, core complex,GO:0016020~membrane,	GO:0003677~DNA binding,GO:0003682~chromatin binding,GO:0003899~DNA-directed RNA polymerase activity,GO:0032549~ribonucleoside binding,GO:0044822~poly(A) RNA binding,	IPR007120:DNA-directed RNA polymerase, subunit 2, domain 6,IPR007121:RNA polymerase, beta subunit, conserved site,IPR007641:RNA polymerase Rpb2, domain 7,IPR007642:RNA polymerase Rpb2, domain 2,IPR007644:RNA polymerase, beta subunit, protrusion,IPR007645:RNA polymerase Rpb2, domain 3,IPR007646:RNA polymerase Rpb2, domain 4,IPR007647:RNA polymerase Rpb2, domain 5,IPR014724:RNA polymerase Rpb2, OB-fold,IPR015712:DNA-directed RNA polymerase, subunit 2,	gga00230:Purine metabolism,gga00240:Pyrimidine metabolism,gga01100:Metabolic pathways,gga03020:RNA polymerase,		
ENSGALG00000016447	protein disulfide isomerase family A member 6(PDIA6)	GO:0006457~protein folding,GO:0034976~response to endoplasmic reticulum stress,GO:0043277~apoptotic cell clearance,GO:0045454~cell redox homeostasis,	GO:0005783~endoplasmic reticulum,GO:0005793~endoplasmic reticulum-Golgi intermediate compartment,GO:0070062~extracellular exosome,	GO:0003756~protein disulfide isomerase activity,	IPR005788:Disulphide isomerase,IPR012336:Thioredoxin-like fold,IPR013766:Thioredoxin domain,IPR017937:Thioredoxin, conserved site,	gga04141:Protein processing in endoplasmic reticulum,		

ENSGALG00000010612	protein kinase D3(PRKD3)	GO:0018105~peptidyl-serine phosphorylation,GO:0089700~protein kinase D signaling,	GO:0005634~nucleus,GO:0005654~nucleoplasm,GO:0005737~cytoplasm,GO:0005886~plasma membrane,	GO:0004674~protein serine/threonine kinase activity,GO:0004697~protein kinase C activity,GO:0005524~ATP binding,GO:0046872~metal ion binding,	IPR000719:Protein kinase, catalytic domain,IPR001849:Pleckstrin homology domain,IPR002219:Protein kinase C-like, phorbol ester/diacylglycerol binding,IPR008271:Serine/threonine-protein kinase, active site,IPR011009:Protein kinase-like domain,IPR011993:Pleckstrin homology-like domain,IPR015727:Protein kinase C mu-related,IPR017441:Protein kinase, ATP binding site,IPR020454:Diacylglycerol/phorbol-ester binding,		PIRSF000552:protein kinase C, mu/nu/D2 types,	SM00109:C1,SM00220:S_TKc,SM00233:PH,
ENSGALG00000009828	protein phosphatase 2, regulatory subunit B', alpha(PPP2R5A)	GO:0007165~signal transduction,GO:0035307~positive regulation of protein dephosphorylation,GO:0090005~negative regulation of establishment of protein localization to plasma membrane,GO:0090219~negative regulation of lipid kinase activity,	GO:0000159~protein phosphatase type 2A complex,GO:0016020~membrane,GO:0030018~Z disc,GO:0031430~M band,	GO:0008601~protein phosphatase type 2A regulator activity,	IPR002554:Protein phosphatase 2A, regulatory B subunit, B56,IPR016024:Armadillo-type fold,	gga03015:mRNA surveillance pathway,gga04114:Oocyte meiosis,gga04261:Adrenergic signaling in cardiomyocytes,	PIRSF028043:serine/threonine-protein phosphatase 2A, subunit B56,	
ENSGALG00000009970	protein phosphatase, Mg2+/Mn2+ dependent, 1B(PPM1B)	GO:0032688~negative regulation of interferon-beta production,GO:0035970~peptidyl-threonine dephosphorylation,GO:0042347~negative regulation of NF-kappaB import into nucleus,GO:0050687~negative regulation of defense response to virus,		GO:0000287~magnesium ion binding,GO:0004722~protein serine/threonine phosphatase activity,GO:0030145~manganese ion binding,	IPR000222:Protein phosphatase 2C, manganese/magnesium aspartate binding site,IPR001932:Protein phosphatase 2C (PP2C)-like,IPR012911:Protein serine/threonine phosphatase 2C, C-terminal,IPR015655:Protein phosphatase 2C,	gga04010:MAPK signaling pathway,		SM00332:PP2Cc,

ENSGALG00000008500	rabenosyn, RAB effector(RBSN)	GO:0000011~vacuole inheritance,GO:0006895~Golgi to endosome transport,GO:0006896~Golgi to vacuole transport,GO:0006897~endocytosis,GO:0034058~endosomal vesicle fusion,GO:0034498~early endosome to Golgi transport,GO:0090160~Golgi to lysosome transport,GO:1903358~regulation of Golgi organization,	GO:0005829~cytosol,GO:0010009~cytoplasmic side of endosome membrane,GO:0070062~extracellular exosome,	GO:0017137~Rab GTPase binding,GO:0046872~metal ion binding,	IPR000306:Zinc finger, FYVE-type,IPR007087:Zinc finger, C2H2,IPR011011:Zinc finger, FYVE/PHD-type,IPR013083:Zinc finger, RING/FYVE/PHD-type,IPR017455:Zinc finger, FYVE-related,IPR021565:FYVE-finger-containing Rab5 effector protein rabenosyn-5,	gga04144:Endocytosis,		SM00064:FYVE,
ENSGALG00000012968	required for meiotic nuclear division 1 homolog(RMND1)	GO:0070131~positive regulation of mitochondrial translation,	GO:0005739~mitochondrion,		IPR003734:Protein of unknown function DUF155,			
ENSGALG00000027035	ribosomal protein L21(RPL21)	GO:0006412~translation,	GO:0005730~nucleolus,GO:0016020~membrane,GO:0022625~cytosolic large ribosomal subunit,	GO:0003735~structural constituent of ribosome,GO:0044822~poly(A) RNA binding,	IPR001147:Ribosomal protein L21e,IPR008991:Translation protein SH3-like domain,IPR018259:Ribosomal protein L21e, conserved site,	gga03010:Ribosome,		
ENSGALG00000005922	ribosomal protein L5(RPL5)	GO:0000027~ribosomal large subunit assembly,GO:0006412~translation,	GO:0022625~cytosolic large ribosomal subunit,	GO:0003735~structural constituent of ribosome,GO:0008097~5S rRNA binding,	IPR005485:Ribosomal protein L5 eukaryotic/L18 archaeal,IPR025607:Ribosomal protein L5 eukaryotic/L18 archaeal, C-terminal,	gga03010:Ribosome,		
ENSGALG00000000218	signal recognition particle 19(SRP19)					gga03060:Protein export,		
ENSGALG00000007439	slowmo homolog 2 (Drosophila)(SLMO2)		GO:0005758~mitochondrial intermembrane space,	GO:1990050~phosphatidic acid transporter activity,	IPR006797:PRELI/MSF1,			
ENSGALG00000010115	sodium channel and clathrin linker 1(SCLT1)	GO:0042384~cilium assembly,	GO:0005814~centriole,GO:0070062~extracellular exosome,GO:0097539~ciliary transition fiber,					
ENSGALG00000016805	sulfotransferase family, cytosolic, 1C, member 3(SULT1C3)	GO:0006790~sulfur compound metabolic process,	GO:0005737~cytoplasm,	GO:0004027~alcohol sulfotransferase activity,GO:0004062~	IPR000863:Sulfotransferase domain,IPR027417:P-loop containing			

				aryl sulfotransferase activity,	nucleoside triphosphate hydrolase,			
ENSGALG00000010152	tetraspanin 8(TSPAN8)	GO:0007166~cell surface receptor signaling pathway,	GO:0005887~integral component of plasma membrane,GO:0070062~extracellular exosome,		IPR000301:Tetraspanin,IPR008952:Tetraspanin, EC2 domain,IPR018499:Tetraspanin/Peripherin,IPR018503:Tetraspanin, conserved site,		PIRSF002419:tetraspanin,	
ENSGALG00000002661	tyrosine 3-monooxygenase/tryptophan 5-monooxygenase activation protein epsilon(YWHAE)	GO:0001764~neuron migration,GO:0006605~protein targeting,GO:0021762~substantia nigra development,GO:0021766~hippocampus development,GO:0021987~cerebral cortex development,GO:0060306~regulation of membrane repolarization,GO:0098609~cell-cell adhesion,GO:1901016~regulation of potassium ion transmembrane transporter activity,GO:1902309~negative regulation of peptidyl-serine dephosphorylation,	GO:0005737~cytoplasm,GO:0005739~mitochondrion,GO:0005913~cell-cell adherens junction,GO:0005925~focal adhesion,GO:0016020~membrane,GO:0070062~extracellular exosome,	GO:0015459~potassium channel regulator activity,GO:0019904~protein domain specific binding,GO:0023026~MHC class II protein complex binding,GO:0031625~ubiquitin protein ligase binding,GO:0042826~histone deacetylase binding,GO:0044325~ion channel binding,GO:0044822~poly(A) RNA binding,GO:0046982~protein heterodimerization activity,GO:0050815~phosphoserine binding,GO:0098641~cadherin binding involved in cell-cell adhesion,	IPR000308:14-3-3 protein,IPR023409:14-3-3 protein, conserved site,IPR023410:14-3-3 domain,	gga04110:Cell cycle,gga04114:Oocyte meiosis,	PIRSF000868:14-3-3 protein,	SM00101:14_3_3,

Biological processes up-regulated in low FCR

Term	p-value	Fold Enrichment	Bonferroni	Benjamini	FDR
GO:0010501~RNA secondary structure unwinding	0.012	17.532	0.908	0.908	14.105
GO:0048739~cardiac muscle fiber development	0.012	157.789	0.914	0.706	14.449
GO:0042981~regulation of apoptotic process	0.021	6.644	0.985	0.752	23.429
GO:0050821~protein stabilization	0.063	7.172	1.000	0.960	55.884
GO:0006913~nucleocytoplasmic transport	0.072	26.298	1.000	0.947	60.815
GO:0006413~translational initiation	0.084	22.541	1.000	0.943	66.484
GO:0001731~formation of translation preinitiation complex	0.084	22.541	1.000	0.943	66.484
GO:0006939~smooth muscle contraction	0.084	22.541	1.000	0.943	66.484
GO:0006457~protein folding	0.085	6.069	1.000	0.916	66.910
GO:0006446~regulation of translational initiation	0.095	19.724	1.000	0.914	71.334

Annotation table for down-regulated genes in low FCR

ID	Gene Name	GOTERM BP	GOTERM CC	GOTERM MF	INTERPRO	KEGG PATHWAY	PIR SUPERFAMILY	SMART
ENSGALG00000003194	ATPase, H+ transporting, lysosomal V0 subunit a2(ATP6V0A2)	GO:0007035~vacuolar acidification,GO:0015986~ATP synthesis coupled proton transport,GO:0015991~ATP hydrolysis coupled proton transport,GO:0070072~vacuolar proton-transporting V-type ATPase complex assembly,	GO:0000220~vacuolar proton-transporting V-type ATPase, V0 domain,GO:0001669~acrosomal vesicle,GO:0005765~lysosomal membrane,GO:0005886~plasma membrane,GO:0005925~focal adhesion,GO:0016021~integral component of membrane,GO:0016471~vacuo	GO:0015078~hydrogen ion transmembrane transporter activity,GO:0016787~hydrolase activity,GO:0046961~proton-transporting ATPase activity, rotational mechanism,GO:0051117~ATPase binding,	IPR002490:ATPase, V0 complex, subunit 116kDa,IPR026028:ATPase, V0 complex, subunit 116kDa, eukaryotic,	gga00190:Oxidative phosphorylation,gga01100:Metabolic pathways,gga04142:Lysosome,gga04145:Phagosome,	PIRSF001293: V-type proton ATPase 116 kda subunit a isoform 1,	

			lar proton-transporting V-type ATPase complex,					
ENSGALG00000000546	F-box only protein 38-like(LOC426699)	GO:0010976~positive regulation of neuron projection development,	GO:0005634~nucleus,GO:0005737~cytoplasm,		IPR001810:F-box domain, cyclin-like,			
ENSGALG00000017329	FCH and double SH3 domains 2(FCHSD2)	GO:0007274~neuromuscular synaptic transmission,GO:0030833~regulation of actin filament polymerization,	GO:0031594~neuromuscular junction,GO:0055037~recycling endosome,		IPR001060:FCH domain,IPR001452:Src homology-3 domain,			SM00055:FCH,SM00326:SH3,
ENSGALG00000002500	GDP-mannose pyrophosphorylase B(GMPPB)	GO:0009058~biosynthetic process,	GO:0005739~mitochondrion,GO:0070062~extracellular exosome,	GO:0016779~nucleotidyltransferase activity,	IPR001451:Bacterial transferase hexapeptide repeat,IPR005835:Nucleotidyl transferase,IPR018357:Hexapeptide transferase, conserved site,	gga00051:Fructose and mannose metabolism,gga00520:Amino sugar and nucleotide sugar metabolism,gga01100:Metabolic pathways,		
ENSGALG00000000993	GTPase activating protein and VPS9 domains 1(GAPVD1)	GO:0007165~signal transduction,GO:0051223~regulation of protein transport,	GO:0005829~cytosol,	GO:0005085~guanyl-nucleotide exchange factor activity,	IPR001936:Ras GTPase-activating protein,IPR003123:Vacuolar sorting protein 9,IPR008936:Rho GTPase activation protein,			SM00167:VPS9,
ENSGALG00000015965	KIAA1429(KIAA1429)	GO:0080009~mRNA methylation,	GO:0016607~nuclear speck,GO:0036396~MIS complex,	GO:0044822~poly(A) RNA binding,	IPR026736:Protein virilizer,			
ENSGALG00000016920	LIM domain 7(LMO7)					gga04520:Adherens junction,		
ENSGALG00000027871	MAD2L1 binding protein(MAD2L1BP)	GO:0007093~mitotic cell cycle checkpoint,GO:0007096~regulation of exit from mitosis,	GO:0005634~nucleus,GO:0005737~cytoplasm,		IPR009511:Mad1/Cdc20-bound-Mad2 binding protein,			
ENSGALG00000004247	MORN repeat containing 3(MORN3)		GO:0005634~nucleus,		IPR003409:MORN motif,			SM00698:MORN,
ENSGALG00000018382	NADH dehydrogenase subunit 1(ND1)	GO:0045333~cellular respiration,	GO:0005743~mitochondrial inner membrane,GO:0005747~mitochondrial respiratory chain	GO:0003954~NADH dehydrogenase activity,GO:0008137~NADH	IPR001694:NADH:ubiquinone oxidoreductas	gga00190:Oxidative phosphorylation,gga01100:Metabolic pathways,		

			complex I,GO:0016021~integral component of membrane,	dehydrogenase (ubiquinone) activity,	e, subunit 1/F420H2 oxidoreductase subunit H,IPR018086: NADH:ubiquinone oxidoreductase, subunit 1, conserved site,			
ENSGALG00000016106	NudC domain containing 1(NUDCD1)		GO:0005634~nucleus,GO:0005737~cytoplasm,		IPR007052:CS-like domain,IPR008978:HSP20-like chaperone,			
ENSGALG00000008135	SATB homeobox 2(SATB2)	GO:0000122~negative regulation of transcription from RNA polymerase II promoter,GO:0001764~neuron migration,GO:0002076~osteoblast development,GO:0006338~chromatin remodeling,GO:0006351~transcription, DNA-templated,GO:0006355~regulation of transcription, DNA-templated,GO:0006357~regulation of transcription from RNA polymerase II promoter,GO:0009880~embryonic pattern specification,GO:0021902~commitment of neuronal cell to specific neuron type in forebrain,GO:0045944~positive regulation of transcription from RNA polymerase II promoter,GO:0048704~embryonic skeletal system morphogenesis,GO:0051216~cartilage	GO:0000118~histone deacetylase complex,GO:0005634~nucleus,GO:0005667~transcription factor complex,GO:0005737~cytoplasm,	GO:0003677~DNA binding,GO:0003682~chromatin binding,GO:0043565~sequence-specific DNA binding,	IPR001356:Homeodomain,IPR003350:Homeodomain protein CUT,IPR009057:Homeodomain-like,IPR010982:Lambda repressor-like, DNA-binding domain,			SM00389:HOX,SM01109:SM01109,

		development,GO:0060021~palate development,GO:0071310~cellular response to organic substance,						
ENSGALG00000010501	SNW domain containing 1(SNW1)	GO:0000122~negative regulation of transcription from RNA polymerase II promoter,GO:0000398~mRNA splicing, via spliceosome,GO:0030511~positive regulation of transforming growth factor beta receptor signaling pathway,GO:0042771~intrinsic apoptotic signaling pathway in response to DNA damage by p53 class mediator,GO:0043923~positive regulation by host of viral transcription,GO:0045944~positive regulation of transcription from RNA polymerase II promoter,GO:0048026~positive regulation of mRNA splicing, via spliceosome,GO:0048384~retinoic acid receptor signaling pathway,GO:0048385~regulation of retinoic acid receptor signaling pathway,GO:0051571~positive regulation of histone H3-K4 methylation,GO:0070564~positive regulation of vitamin D receptor signaling pathway,GO:0071300~cellular response to retinoic acid,	GO:0008024~cyclin/CDK positive transcription elongation factor complex,GO:0016363~nuclear matrix,GO:0071013~catalytic step 2 spliceosome,GO:0071146~SMAD3-SMAD4 protein complex,	GO:0003713~transcription coactivator activity,GO:0003714~transcription corepressor activity,GO:0044822~poly(A) RNA binding,	IPR004015:SKI-interacting protein SKIP, SNW domain,IPR017862:SKI-interacting protein, SKIP,	gga03040:Spliceosome,gga04330:Notch signaling pathway,		
ENSGALG00000000903	TERF2 interacting protein(TERF2IP)	GO:0000723~telomere maintenance,GO:0006351~transcription, DNA-	GO:0000781~chromosome, telomeric region,GO:0005634~nucleus,G	GO:0003677~DNA binding,GO:0098505~G-rich strand	IPR001357:BRCT domain,IPR00			

		templated,GO:0006355~regulation of transcription, DNA-templated,GO:0010569~regulation of double-strand break repair via homologous recombination,GO:0010833~telomere maintenance via telomere lengthening,GO:0031848~protection from non-homologous end joining at telomere,GO:0032205~negative regulation of telomere maintenance,GO:0033138~positive regulation of peptidyl-serine phosphorylation,GO:0043123~positive regulation of I-kappaB kinase/NF-kappaB signaling,GO:0048239~negative regulation of DNA recombination at telomere,GO:0051092~positive regulation of NF-kappaB transcription factor activity,GO:0070198~protein localization to chromosome, telomeric region,GO:1901985~positive regulation of protein acetylation,	O:0005635~nuclear envelope,GO:0005654~nucleoplasm,GO:0005737~cytoplasm,GO:0030870~Mre11 complex,GO:0070187~telosome,	telomeric DNA binding,	9057:Homeodomain-like,IPR015010:Rap1 Myb domain,IPR021661:Rap1 - C-terminal,			
ENSGALG00000006420	abhydrolase domain containing 17C(ABHD17C)			GO:0016787~hydrolase activity,	IPR022742:Alpha/beta hydrolase, N-terminal,			
ENSGALG00000015556	actin filament associated protein 1(AFAP1)	GO:0009966~regulation of signal transduction,GO:0018108~peptidyl-tyrosine phosphorylation,GO:0018109~peptidyl-arginine phosphorylation,GO:00	GO:0005737~cytoplasm,GO:0005856~cytoskeleton,GO:0005886~plasma membrane,GO:0005925~focal adhesion,	GO:0003779~actin binding,GO:0005515~protein binding,GO:0017124~SH3 domain binding,GO:0042169~SH2 domain binding,	IPR001849:Pleckstrin homology domain,IPR011993:Pleckstrin homology-like domain,			SM00233:PH,

		51493~regulation of cytoskeleton organization,						
ENSGALG00000016855	arginine and glutamate rich 1(ARGLU1)	GO:0098609~cell-cell adhesion,	GO:0005654~nucleoplasm,GO:0005739~mitochondrion,GO:0005913~cell-cell adherens junction,	GO:0098641~cadherin binding involved in cell-cell adhesion,				
ENSGALG00000008459	bone morphogenetic protein receptor type II(BMPR2)	GO:0001707~mesoderm formation,GO:0001935~endothelial cell proliferation,GO:0001938~positive regulation of endothelial cell proliferation,GO:0001946~lymphangiogenesis,GO:0001974~blood vessel remodeling,GO:0002063~chondrocyte development,GO:0003085~negative regulation of systemic arterial blood pressure,GO:0006366~transcription from RNA polymerase II promoter,GO:0009267~cellular response to starvation,GO:0009952~anterior/posterior pattern specification,GO:0010595~positive regulation of endothelial cell migration,GO:0010862~positive regulation of pathway-restricted SMAD protein phosphorylation,GO:0014916~regulation of lung blood pressure,GO:0030166~proteoglycan biosynthetic process,GO:0030308~negative regulation of cell growth,GO:0030501~positive regulation of bone mineralization,GO:0030	GO:0005615~extracellular space,GO:0005887~integral component of plasma membrane,GO:0005901~caveola,GO:0009986~cell surface,GO:0016021~integral component of membrane,	GO:0004675~transmembrane receptor protein serine/threonine kinase activity,GO:0004702~receptor signaling protein serine/threonine kinase activity,GO:0005524~ATP binding,GO:0098821~BMP receptor activity,	IPR000333:Activin type II/Transforming growth factor-beta II receptor,IPR000472:TGF-beta receptor/activin receptor, type I/II,IPR000719:Protein kinase, catalytic domain,IPR011009:Protein kinase-like domain,IPR015770:Bone morphogenic protein type II receptor,IPR017441:Protein kinase, ATP binding site,	gga04060:Cytokine-cytokine receptor interaction,gga04350:TGF-beta signaling pathway,		

		513~positive regulation of BMP signaling pathway,GO:0045669~positive regulation of osteoblast differentiation,GO:0045906~negative regulation of vasoconstriction,GO:0045944~positive regulation of transcription from RNA polymerase II promoter,GO:0048010~vascular endothelial growth factor receptor signaling pathway,GO:0048286~lung alveolus development,GO:0048842~positive regulation of axon extension involved in axon guidance,GO:0060173~limb development,GO:0060350~endochondral bone morphogenesis,GO:0060836~lymphatic endothelial cell differentiation,GO:0060840~artery development,GO:0060841~venous blood vessel development,GO:0061036~positive regulation of cartilage development,GO:0061298~retina vasculature development in camera-type eye,GO:0072577~endothelial cell apoptotic process,GO:1902731~negative regulation of chondrocyte proliferation,GO:2000279~negative regulation of DNA biosynthetic process,							

ENSGALG00000004338	collagen, type XVIII, alpha 1(COL18A1)	GO:0001525~angiogenesis,GO:0001886~endothelial cell morphogenesis,GO:0007155~cell adhesion,GO:0008284~positive regulation of cell proliferation,GO:0030198~extracellular matrix organization,GO:0030335~positive regulation of cell migration,GO:2000353~positive regulation of endothelial cell apoptotic process,	GO:0005578~proteinaceous extracellular matrix,GO:0005581~collagen trimer,GO:0005604~basement membrane,GO:0005615~extracellular space,GO:0070062~extracellular exosome,	GO:0005198~structural molecule activity,	IPR001791:Laminin G domain,IPR008160:Collagen triple helix repeat,IPR010515:Collagenase NC10/endostatin,IPR013320:Concanavalin A-like lectin/glucanase, subgroup,IPR016186:C-type lectin-like,IPR016187:C-type lectin fold,				SM00210:TSPN,SM00282:LamG,
ENSGALG00000012213	cornichon family AMPA receptor auxiliary protein 1(CNIH1)	GO:0006810~transport,	GO:0016021~integral component of membrane,		IPR003377:Cornichon,				SM01398:SM01398,
ENSGALG00000012295	cyclin-dependent kinase-like 1 (CDC2-related kinase)(CDKL1)		GO:0070062~extracellular exosome,	GO:0004674~protein serine/threonine kinase activity,GO:0005524~ATP binding,	IPR000719:Protein kinase, catalytic domain,IPR008271:Serine/threonine-protein kinase, active site,IPR011009:Protein kinase-like domain,IPR017441:Protein kinase, ATP binding site,				SM00220:S_TKc,
ENSGALG00000006050	cytochrome P450 family 2 subfamily R member 1(CYP2R1)			GO:0005506~iron ion binding,GO:0008395~steroid hydroxylase activity,GO:0016705~oxidoreductase activity, acting on paired donors, with incorporation or reduction of	IPR001128:Cytochrome P450,IPR002401:Cytochrome P450, E-class, group I,IPR017972:Cytochrome P450, conserved site,	gga00100:Steroid biosynthesis,gga01100:Metabolic pathways,			

				molecular oxygen,GO:0020037~heme binding,GO:0030343~vitamin D3 25-hydroxylase activity,				
ENSGALG00000008325	cytoskeleton associated protein 5(CKAP5)	GO:0007051~spindle organization,GO:0030951~establishment or maintenance of microtubule cytoskeleton polarity,GO:0051297~centrosome organization,	GO:0000922~spindle pole,GO:0000930~gamma-tubulin complex,GO:0005813~centrosome,GO:0016020~membrane,GO:0035371~microtubule plus-end,		IPR011989:Armadillo-like helical,IPR016024:Armadillo-type fold,IPR024395:CLASP N-terminal domain,			
ENSGALG00000007119	diacylglycerol lipase beta(DAGLB)	GO:0007405~neuroblast proliferation,GO:0019369~arachidonic acid metabolic process,GO:0042136~neurotransmitter biosynthetic process,	GO:0005765~lysosomal membrane,GO:0005886~plasma membrane,	GO:0016787~hydrolase activity,	IPR002921:Lipase, class 3,			
ENSGALG00000012929	dynamin 1-like(DNM1L)	GO:0000266~mitochondrial fission,GO:0001836~release of cytochrome c from mitochondria,GO:0003374~dynamin polymerization involved in mitochondrial fission,GO:0008637~apoptotic mitochondrial changes,GO:0016559~peroxisome fission,GO:0032459~regulation of protein oligomerization,GO:0043653~mitochondrial fragmentation involved in apoptotic process,GO:0050714~positive regulation of protein secretion,GO:0051289~protein homotetramerization,GO:0060047~heart	GO:0005741~mitochondrial outer membrane,GO:0005777~peroxisome,GO:0005794~Golgi apparatus,GO:0005829~cytosol,GO:0005903~brush border,GO:0015630~microtubule cytoskeleton,GO:0031966~mitochondrial membrane,GO:0043234~protein complex,GO:0048471~perinuclear region of cytoplasm,	GO:0003924~GTPase activity,GO:0005525~GTP binding,GO:0008017~microtubule binding,	IPR000375:Dynamin central domain,IPR001401:Dynamin, GTPase domain,IPR003130:Dynamin GTPase effector,IPR019762:Dynamin, GTPase region, conserved site,IPR020850:GTPase effector domain, GED,IPR022812:Dynamin,IPR027417:P-loop containing nucleoside triphosphate hydrolase,			SM00053:DYNc,SM00302:GED,

		contraction,GO:0061025~membrane fusion,GO:0070266~necroptotic process,GO:0070584~mitochondrion morphogenesis,GO:0070585~protein localization to mitochondrion,GO:0090141~positive regulation of mitochondrial fission,GO:0090149~mitochondrial membrane fission,GO:0090200~positive regulation of release of cytochrome c from mitochondria,GO:1900063~regulation of peroxisome organization,GO:1903146~regulation of mitophagy,GO:1903578~regulation of ATP metabolic process,GO:2001244~positive regulation of intrinsic apoptotic signaling pathway,						
ENSGALG00000000242	early B-cell factor 2(EBF2)	GO:0001709~cell fate determination,GO:0006351~transcription, DNA-templated,GO:0006355~regulation of transcription, DNA-templated,GO:0007275~multicellular organism development,GO:0035563~positive regulation of chromatin binding,GO:0045944~positive regulation of transcription from RNA polymerase II promoter,GO:0050873~brown fat cell differentiation,GO:0060	GO:0005634~nucleus,	GO:0000978~RNA polymerase II core promoter proximal region sequence-specific DNA binding,GO:0001077~transcriptional activator activity, RNA polymerase II core promoter proximal region sequence-specific binding,GO:0003677~DNA binding,GO:0003682~chromatin binding,GO:0046872~metal ion binding,	IPR002909:Cell surface receptor IPT/TIG,IPR003523:Transcription factor COE,IPR013783:Immunoglobulin-like fold,IPR014756:Immunoglobulin E-set,IPR018350:Transcription factor COE, conserved site,			SM00429:IPT,

		612~adipose tissue development,						
ENSGALG00000008139	echinoderm microtubule associated protein like 6(EML6)				IPR001680:WD40 repeat,IPR005108:HELP,IPR011047:Quinonprotein alcohol dehydrogenase-like superfamily,IPR015943:WD40/YVTN repeat-like-containing domain,IPR017986:WD40-repeat-containing domain,IPR019775:WD40 repeat, conserved site,IPR024977:Anaphase-promoting complex subunit 4, WD40 domain,			SM00320:WD40,
ENSGALG00000008936	ectonucleoside triphosphate diphosphohydrolase 8-like(ENTPD8L)		GO:0016021~integral component of membrane,	GO:0016787~hydrolase activity,	IPR000407:Nucleoside phosphatase GDA1/CD39,	gga00230:Purine metabolism,gga00240:Pyrimidine metabolism,		
ENSGALG00000006512	heat shock 70kDa protein 8(HSPA8)	GO:0006892~post-Golgi vesicle-mediated transport,GO:0031647~regulation of protein stability,GO:0042026~protein refolding,GO:0045892~negative regulation of transcription, DNA-templated,GO:0046034~ATP metabolic process,GO:0048026~positive regulation of mRNA splicing, via	GO:0000151~ubiquitin ligase complex,GO:0000974~Prp19 complex,GO:0005634~nucleus,GO:0005737~cytoplasm,GO:0005770~late endosome,GO:0005829~cytosol,GO:0005925~focal adhesion,GO:0016020~membrane,GO:0030529~intracellular ribonucleoprotein complex,GO:0043209~myelin sheath,GO:0070062~extracellular	GO:0001786~phosphatidylserine binding,GO:0005524~ATP binding,GO:0042623~ATPase activity, coupled,GO:0044822~poly(A) RNA binding,	IPR013126:Heat shock protein 70 family,IPR018181:Heat shock protein 70, conserved site,	gga03040:Spliceosome,gga04010:MAPK signaling pathway,gga04141:Protein processing in endoplasmic reticulum,gga04144:Endocytosis,gga05164:Influenza A,		

		spliceosome,GO:0051085~chaperone mediated protein folding requiring cofactor,GO:0051726~regulation of cell cycle,GO:0061024~membrane organization,GO:0061738~late endosomal microautophagy,GO:0072318~clathrin coat disassembly,GO:1902904~negative regulation of fibril organization,	exosome,GO:0072562~blood microparticle,					
ENSGALG00000008978	integrin, alpha 4 (antigen CD49D, alpha 4 subunit of VLA-4 receptor)(ITGA4)	GO:0003366~cell-matrix adhesion involved in ameboidal cell migration,GO:0007229~integrin-mediated signaling pathway,GO:0034113~heterotypic cell-cell adhesion,GO:0034446~substrate adhesion-dependent cell spreading,GO:0035987~endodermal cell differentiation,GO:0043113~receptor clustering,GO:0050901~leukocyte tethering or rolling,GO:0090074~negative regulation of protein homodimerization activity,	GO:0005925~focal adhesion,GO:0009986~cell surface,GO:0034669~integrin alpha4-beta7 complex,GO:0070062~extracellular exosome,		IPR000413:Integrin alpha chain,IPR013517:FG-GAP repeat,IPR013519:Integrin alpha beta-propellor,IPR013649:Integrin alpha-2,IPR018184:Integrin alpha chain, C-terminal cytoplasmic region, conserved site,	gga04510:Focal adhesion,gga04512:ECM-receptor interaction,gga04514:Cell adhesion molecules (CAMs),gga04672:Intestinal immune network for IgA production,gga04810:Regulation of actin cytoskeleton,		SM00191:Int_alpha,
ENSGALG00000006032	interleukin 13 receptor, alpha 1(IL13RA1)					gga04060:Cytokine-cytokine receptor interaction,gga04630:Jak-STAT signaling pathway,		
ENSGALG00000013441	inversin(INVS)	GO:0007275~multicellular organism development,GO:0016055~Wnt signaling pathway,GO:0090090~negative regulation of canonical Wnt signaling pathway,	GO:0005737~cytoplasm,GO:0005856~cytoskeleton,	GO:0005516~calmodulin binding,	IPR000048:IQ motif, EF-hand binding site,IPR002110:Ankyrin repeat,IPR020683:Ankyrin repeat-			SM00015:IQ, SM00248:ANK,

					containing domain,			
ENSGALG00000003513	kelch like family member 20(KLHL20)	GO:0006895~Golgi to endosome transport,GO:0015031~protein transport,GO:0016567~protein ubiquitination,GO:0035455~response to interferon-alpha,GO:0042787~protein ubiquitination involved in ubiquitin-dependent protein catabolic process,GO:0043066~negative regulation of apoptotic process,GO:0043161~proteasome-mediated ubiquitin-dependent protein catabolic process,GO:1990390~protein K33-linked ubiquitination,	GO:0005737~cytoplasm,GO:0005802~trans-Golgi network,GO:0005829~cytosol,GO:0016605~PML body,GO:0031463~Cul3-RING ubiquitin ligase complex,GO:0048471~perinuclear region of cytoplasm,	GO:0004842~ubiquitin-protein transferase activity,GO:0019964~interferon-gamma binding,	IPR000210:BTB/POZ-like,IPR006652:Kelch repeat type 1,IPR011043:Galactose oxidase/kelch, beta-propeller,IPR011333:BTB/POZ fold,IPR011705:BTB/Kelch-associated,IPR015916:Galactose oxidase, beta-propeller,IPR017096:Kelch-like protein, gigaxonin,		PIRSF037037:kelch-like protein, gigaxonin type,	SM00225:BTB,SM00612:Kelch,SM00875:SM00875,
ENSGALG00000023824	lysosomal acid lipase/cholesteryl ester hydrolase-like(LOC428958)	GO:0016042~lipid catabolic process,		GO:0016788~hydrolase activity, acting on ester bonds,	IPR000073:Alpha/beta hydrolase fold-1,IPR025483:Lipase, eukaryotic,		PIRSF000862:lipase, eukaryotic type,	
ENSGALG00000028063	lysyl oxidase(LOX)	GO:0007507~heart development,GO:0030199~collagen fibril organization,GO:0030324~lung development,GO:0035904~aorta development,GO:0048251~elastic fiber assembly,GO:0055114~oxidation-reduction process,	GO:0005578~proteinaceous extracellular matrix,GO:0005581~collagen trimer,GO:0005615~extracellular space,	GO:0004720~protein-lysine 6-oxidase activity,GO:0005507~copper ion binding,	IPR001695:Lysyl oxidase,IPR019828:Lysyl oxidase, conserved site,			
ENSGALG00000000402	lysyl oxidase-like 2(LOXL2)	GO:0001666~response to hypoxia,GO:0001837~epithelial to mesenchymal	GO:0005604~basement membrane,GO:0005615~extracellular space,GO:0005634~nucleus,GO:0005654~nucleoplasm,GO:0	GO:0003682~chromatin binding,GO:0003714~transcription corepressor	IPR001190:Speract/scavenger receptor,IPR001695:Lysyl			SM00202:SR,

		transition,GO:0001935~endothelial cell proliferation,GO:0002040~sprouting angiogenesis,GO:0006351~transcription, DNA-templated,GO:0006898~receptor-mediated endocytosis,GO:0016569~covalent chromatin modification,GO:0016570~histone modification,GO:0018277~protein deamination,GO:0030199~collagen fibril organization,GO:0032332~positive regulation of chondrocyte differentiation,GO:0043542~endothelial cell migration,GO:0045892~negative regulation of transcription, DNA-templated,GO:0046688~response to copper ion,GO:0055114~oxidation-reduction process,	005694~chromosome,GO:0016020~membrane,	activity,GO:0004720~protein-lysine 6-oxidase activity,GO:0005044~scavenger receptor activity,GO:0005507~copper ion binding,GO:0070492~oligosaccharide binding,	oxidase,IPR017448:Speract/scavenger receptor-related,IPR019828:Lysyl oxidase, conserved site,			
ENSGALG00000004975	magnesium transporter NIPA2-like(LOC769755)	GO:0015693~magnesium ion transport,	GO:0016020~membrane,GO:0016021~integral component of membrane,	GO:0015095~magnesium ion transmembrane transporter activity,	IPR008521:Magnesium transporter NIPA,			
ENSGALG00000010719	nei like DNA glycosylase 3(NEIL3)	GO:0006284~base-excision repair,GO:0006289~nucleotide-excision repair,	GO:0005654~nucleoplasm,	GO:0000405~bubble DNA binding,GO:0003684~damaged DNA binding,GO:0003690~double-stranded DNA binding,GO:0003697~single-stranded DNA binding,GO:0003906~DNA-(apurinic or apyrimidinic site) lyase activity,GO:0008270~zinc ion binding,GO:001910	IPR000214:Zinc finger, DNA glycosylase/AP lyase-type,IPR001876:Zinc finger, RanBP2-type,IPR010666:Zinc finger, GRF-type,IPR010979:Ribosomal protein S13-like, H2TH,IPR012319:DNA	gga03410:Base excision repair,		SM00547:ZnF_RBZ,SM00898:SM00898,SM01232:SM01232,

				4~DNA N-glycosylase activity,	glycosylase/AP lyase, catalytic domain,IPR015886:DNA glycosylase/AP lyase, H2TH DNA-binding,IPR015887:DNA glycosylase/AP lyase, zinc finger domain, DNA-binding site,			
ENSGALG00000015086	oxysterol binding protein-like 1A(OSBPL1A)	GO:0006869~lipid transport,	GO:0005654~nucleoplasm,GO:0005770~late endosome,GO:0070062~extracellular exosome,		IPR000648:Oxysterol-binding protein,IPR001849:Pleckstrin homology domain,IPR002110:Ankyrin repeat,IPR011993:Pleckstrin homology-like domain,IPR018494:Oxysterol-binding protein, conserved site,IPR020683:Ankyrin repeat-containing domain,			SM00233:PH,SM00248:ANK,
ENSGALG00000005805	phospholipase C, delta 1(PLCD1)	GO:0001525~angiogenesis,GO:0016042~lipid catabolic process,GO:0035556~intracellular signal transduction,GO:0042127~regulation of cell proliferation,	GO:0005829~cytosol,GO:0070062~extracellular exosome,	GO:0001786~phosphatidylserine binding,GO:0004435~phosphatidylinositol phospholipase C activity,GO:0004871~signal transducer activity,GO:0005509~calcium ion binding,GO:0070300~phosphatidic acid binding,GO:1901981~phosphatidylinosi	IPR000008:C2 calcium-dependent membrane targeting,IPR000909:Phospholipase C, phosphatidylinositol-specific, X domain,IPR001192:Phosphoinositide phospholipase	gga00562:Inositol phosphate metabolism,gga01100:Metabolic pathways,gga04020:Calcium signaling pathway,gga04070:Phosphatidylinositol signaling system,		SM00148:PLCXc,SM00149:PLCYc,SM00233:PH,SM00239:C2,

				tol phosphate binding,	C,IPR001711:Phospholipase C, phosphatidylinositol-specific, Y domain,IPR001849:Pleckstrin homology domain,IPR002048:EF-hand domain,IPR011992:EF-hand-like domain,IPR011993:Pleckstrin homology-like domain,IPR015359:Phospholipase C, phosphoinositol-specific, EF-hand-like,IPR017946:PLC-like phosphodiesterase, TIM beta/alpha-barrel domain,IPR018247:EF-Hand 1, calcium-binding site,			
ENSGALG00000009919	potassium voltage-gated channel modifier subfamily G member 3(KCNG3)	GO:0051260~protein homooligomerization,	GO:0005783~endoplasmic reticulum,GO:0008076~voltage-gated potassium channel complex,GO:0016021~integral component of membrane,	GO:0005251~delayed rectifier potassium channel activity,	IPR000210:BTB/POZ-like,IPR003131:Potassium channel tetramerisation-type BTB domain,IPR003968:Potassium channel, voltage dependent, Kv,IPR003971:Potassium channel, voltage			SM00225:BTB,

					dependent, Kv9,IPR005821:Ion transport domain,IPR011333:BTB/POZ fold,IPR027359:Voltage-dependent potassium channel, four helix bundle domain,			
ENSGALG00000006472	potassium voltage-gated channel subfamily Q member 1(KCNQ1)					gga04261:Adrenergic signaling in cardiomyocytes,		
ENSGALG00000011259	retinal pigment epithelium-specific protein 65kDa(RPE65)	GO:0001895~retina homeostasis,GO:0007468~regulation of rhodopsin gene expression,GO:0008286~insulin receptor signaling pathway,GO:0042572~retinol metabolic process,GO:0042574~retinal metabolic process,GO:0050908~detection of light stimulus involved in visual perception,GO:0055114~oxidation-reduction process,GO:0060042~retina morphogenesis in camera-type eye,GO:0071257~cellular response to electrical stimulus,	GO:0005783~endoplasmic reticulum,GO:0005886~plasma membrane,GO:0016020~membrane,GO:0031090~organelle membrane,	GO:0001786~phosphatidylserine binding,GO:0004744~retinal isomerase activity,GO:0016702~oxidoreductase activity, acting on single donors with incorporation of molecular oxygen, incorporation of two atoms of oxygen,GO:0031210~phosphatidylcholine binding,GO:0046872~metal ion binding,GO:0052884~all-trans-retinyl-palmitate hydrolase, 11-cis retinol forming activity,GO:0052885~all-trans-retinyl-ester hydrolase, 11-cis retinol forming activity,GO:1901612~cardiolipin binding,	IPR004294:Carotenoid oxygenase,	gga00830:Retinol metabolism,		
ENSGALG00000011120	solute carrier family 4, sodium	GO:0009416~response to light	GO:0005887~integral component of plasma	GO:0005452~inorganic anion	IPR003020:Bicarbonate			

	bicarbonate transporter, member 10(SLC4A10)	stimulus,GO:0009791~post-embryonic development,GO:0015701~bicarbonate transport,GO:0021860~pyramidal neuron development,GO:0035264~multicellular organism growth,GO:0035641~locomotory exploration behavior,GO:0048854~brain morphogenesis,GO:0051453~regulation of intracellular pH,GO:0098656~anion transmembrane transport,GO:1902600~hydrogen ion transmembrane transport,	membrane,GO:0016323~basolateral plasma membrane,GO:0043025~neuronal cell body,GO:0097440~apical dendrite,GO:0097441~basilar dendrite,GO:0097442~CA3 pyramidal cell dendrite,	exchanger activity,GO:0008509~anion transmembrane transporter activity,GO:0015301~anion:anion antiporter activity,	transporter, eukaryotic,IPR003024:Sodium bicarbonate cotransporter,IPR011531:Bicarbonate transporter, C-terminal,IPR013769:Band 3 cytoplasmic domain,IPR016152:Phosphotransferase/anion transporter,			
ENSGALG00000010706	teneurin transmembrane protein 3(TENM3)	GO:0000902~cell morphogenesis,GO:0007157~heterophilic cell-cell adhesion via plasma membrane cell adhesion molecules,GO:0007165~signal transduction,GO:0048593~camera-type eye morphogenesis,GO:0048666~neuron development,GO:0097264~self proteolysis,	GO:0005887~integral component of plasma membrane,GO:0016021~integral component of membrane,GO:0043005~neuron projection,	GO:0042803~protein homodimerization activity,GO:0046982~protein heterodimerization activity,GO:0050839~cell adhesion molecule binding,	IPR000742:Epidermal growth factor-like domain,IPR006530:YD repeat,IPR008969:Carboxypeptidase-like, regulatory domain,IPR009471:Teneurin intracellular, N-terminal,IPR011042:Six-bladed beta-propeller, TolB-like,IPR013032:EGF-like, conserved site,IPR022385:Rhs repeat-associated core,			SM00181:EGF,
ENSGALG00000015448	thymocyte selection				IPR009071:High mobility			SM00398:HMG,

	associated high mobility group box(TOX)				group (HMG) box domain,			
ENSGALG00000003849	transient receptor potential cation channel, subfamily M, member 1(TRPM1)	GO:0007216~G-protein coupled glutamate receptor signaling pathway,GO:0007601~visual perception,GO:0046548~retinal rod cell development,GO:0051262~protein tetramerization,GO:0060402~calcium ion transport into cytosol,GO:0071482~cellular response to light stimulus,	GO:0016021~integral component of membrane,GO:0035841~new growing cell tip,	GO:0005262~calcium channel activity,	IPR005821:Ion transport domain,			
ENSGALG00000005535	treacle ribosome biogenesis factor 1(TCOF1)				IPR003993:Treacher Collins syndrome, treacle,IPR006594:LisH dimerisation motif,IPR017859:Treacle-like, Treacher Collins Syndrome,	gga03008:Ribosome biogenesis in eukaryotes,		SM00667:LisH,
ENSGALG00000008099	tripartite motif containing 8(TRIM8)	GO:0010508~positive regulation of autophagy,GO:0019827~stem cell population maintenance,GO:0032897~negative regulation of viral transcription,GO:0043123~positive regulation of I-kappaB kinase/NF-kappaB signaling,GO:0045087~innate immune response,GO:0046597~negative regulation of viral entry into host cell,GO:0051092~positive regulation of NF-kappaB transcription factor activity,GO:1900182~p	GO:0005622~intracellular,GO:0005829~cytosol,GO:0016605~PML body,	GO:0008270~zinc ion binding,	IPR001841:Zinc finger, RING-type,IPR013083:Zinc finger, RING/FYVE/PHD-type,IPR017907:Zinc finger, RING-type, conserved site,			SM00184:RING,

		ositive regulation of protein localization to nucleus,GO:1902187~negative regulation of viral release from host cell,						
ENSGALG00000026875	uncharacterized LOC428186(LOC428186)							
ENSGALG00000008547	zinc finger, BED-type containing 4(ZBED4)	GO:0006357~regulation of transcription from RNA polymerase II promoter,	GO:0005634~nucleus,GO:0005737~cytoplasm,	GO:0000977~RNA polymerase II regulatory region sequence-specific DNA binding,GO:0003700~transcription factor activity, sequence-specific DNA binding,	IPR003656:Zinc finger, BED-type predicted,IPR008906:HAT dimerisation,IPR012337:Ribonuclease H-like domain,			SM00614:ZnF_BED,

Supplementary Table 14: eQTls identified in Duodenum of low and high FCR broilers

chr	Ref	Alt	pos	id	NegLog10_p_Value	Gene	Location
4	G	A	33493534	rs739281432	1.811627256	CDKL2	intron
4	G	T	33493536	rs731824742	1.811627256	CDKL2	intron
4	C	G	33493544	rs313558088	1.811627256	CDKL2	intron
3	G	A	31344039	rs316497105	1.745682121	EIF2AK2	upstream
4	A	G	33493943	rs738401365	1.694058784	CDKL2	intron
4	C	A	33493947	rs741004252	1.694058784	CDKL2	intron
4	G	T	33493973	rs314887600	1.472730199	CDKL2	intron
4	G	A	33493977	rs317109468	1.472730199	CDKL2	intron
4	G	T	33494004	rs738418882	1.472730199	CDKL2	intron
4	A	G	33494006	rs730944916	1.472730199	CDKL2	intron
3	G	A	31345086	rs736774899	1.455746105	EIF2AK2	upstream
3	T	C	31343215	rs316268789	1.330775335	EIF2AK2	upstream
4	T	C	33494644	rs312995198	1.319906838	CDKL2	intron
3	T	C	31360756	rs741254257	1.193294397	EIF2AK2	intron
4	G	A	33470929	rs13514923	1.193294397	CDKL2	Downstream
2	G	A	41818789	rs312409429	1.175236095	ENSGALG00000011530	3'UTR
3	G	A	31342621	rs16249161	1.175236095	EIF2AK2	upstream
3	A	G	31342800	rs16249162	1.175236095	EIF2AK2	upstream
3	C	G	31342831	rs16249163	1.175236095	EIF2AK2	upstream
1	C	T	175515025	rs313603894	1.163534327	GTF3A	intron
1	A	G	175515038	rs734238663	1.163534327	GTF3A	intron
1	T	C	175515044	rs739083009	1.163534327	GTF3A	intron
1	C	A	175515054	rs731266678	1.163534327	GTF3A	intron
1	C	A	175515055	rs736020742	1.163534327	GTF3A	intron
4	G	A	33497221	rs14449638	1.152464471	CDKL2	intron
4	A	G	33496277	rs317083035	1.117702576	CDKL2	intron
4	G	T	33493818	rs14449627	1.05450697	CDKL2	intron
3	A	G	31360009	rs14336055	1.051248307	EIF2AK2	intron
4	G	C	33470551	rs735091870	1.051248307	CDKL2	Downstream
4	A	G	33470578	rs316096516	1.051248307	CDKL2	Downstream
4	G	A	33493842	rs14449630	1.051248307	CDKL2	intron
4	C	T	33493866	rs14449632	1.051248307	CDKL2	intron
3	C	T	31345175	rs734117298	1.040127027	EIF2AK2	upstream
3	A	G	31344100	rs314187342	1.025652722	EIF2AK2	upstream
3	A	G	31344778	rs313156744	1.025652722	EIF2AK2	upstream
4	C	T	33495392	rs736913598	1.025652722	CDKL2	intron
4	C	T	33494545	rs14449633	1.015919651	CDKL2	intron
4	T	C	33494553	rs14449634	1.015919651	CDKL2	intron
4	A	G	33495714	rs313597978	1.010150071	CDKL2	intron

4	T	C	33494678	rs738049792	1.006374543	CDKL2	intron
4	A	G	33494680	rs731746034	1.006374543	CDKL2	intron
3	A	G	31361758	rs317327414	0.989401421	EIF2AK2	intron
3	C	T	31341896	rs732979069	0.982117511	EIF2AK2	upstream
4	G	A	33471405	rs313465155	0.982117511	CDKL2	Downstream
4	T	C	33476361	rs13514939	0.982117511	CDKL2	intron
4	G	C	33476367	rs13514940	0.982117511	CDKL2	intron
4	A	T	33476375	rs13514941	0.982117511	CDKL2	intron
3	G	A	31343887	rs737208454	0.951334318	EIF2AK2	upstream
3	T	C	31341962	rs13722631	0.937110171	EIF2AK2	upstream
3	A	G	31341322	rs16249157	0.900680427	EIF2AK2	upstream
4	C	T	33493731	rs312654406	0.900680427	CDKL2	intron
4	A	G	33495545	rs16388203	0.900680427	CDKL2	intron
3	A	G	31357694	rs730896723	0.897461823	EIF2AK2	synonymus
4	T	A	33499891	rs317772076	0.884156536	CDKL2	synonymus
3	A	C	31345035	rs740806908	0.880787805	EIF2AK2	upstream
3	T	C	31347388	rs314000779	0.858476827	EIF2AK2	5'UTR
3	C	T	31342620	rs312973864	0.857636384	EIF2AK2	upstream
3	C	G	31344055	rs316671698	0.857636384	EIF2AK2	upstream
3	A	G	31344091	rs315054227	0.857636384	EIF2AK2	upstream
4	G	A	33495364	rs16388201	0.857636384	CDKL2	intron
3	A	G	31344656	rs739175623	0.85496093	EIF2AK2	upstream
1	G	A	175510913	rs732716059	0.755143376	GTF3A	Downstream
3	A	G	31360663	rs737186652	0.755143376	EIF2AK2	intron
3	A	G	31360809	rs313475668	0.755143376	EIF2AK2	intron
3	C	T	31363573	rs739016424	0.755143376	EIF2AK2	intron
4	T	A	33470389	_Variant_193	0.755143376	CDKL2	Downstream
4	T	A	33470390	rs314435328	0.755143376	CDKL2	Downstream
3	A	G	31343416	rs15316355	0.749837397	EIF2AK2	upstream
4	A	T	33503789	rs317349835	0.749837397	CDKL2	synonymus
4	G	A	33513449	rs316763856	0.749837397	CDKL2	upstream
4	A	T	33493992	rs317986004	0.725208629	CDKL2	intron
3	C	G	31360105	rs732186284	0.722330588	EIF2AK2	intron
3	C	T	31344320	rs731509297	0.717186237	EIF2AK2	upstream
1	G	A	175514666	rs316498108	0.686336881	GTF3A	splice region, intron
3	A	G	16377734	rs313482784	0.686336881	PARP1	synonymus
3	C	T	16391277	rs315080437	0.681187826	PARP1	downstream
3	G	A	31344637	rs735760013	0.673882259	EIF2AK2	upstream
3	T	C	31344675	rs313820933	0.65942818	EIF2AK2	upstream
3	G	T	31353604	rs14336041	0.65942818	EIF2AK2	Non-synonymous

4	G	A	33480755	rs313715704	0.65942818	CDKL2	intron
3	C	T	16377749	rs315105112	0.655412704	PARP1	synonymus
4	A	G	33504732	rs317018980	0.655412704	CDKL2	synonymus
2	G	A	41818427	rs316216202	0.648343264	ENSGALG00000011530	3'UTR
4	A	C	33467673	rs736361027	0.648343264	CDKL2	Downstream
4	A	T	33467674	rs741407834	0.648343264	CDKL2	Downstream
4	T	C	33467681	rs16388137	0.648343264	CDKL2	Downstream
4	T	C	33467719	rs316774601	0.648343264	CDKL2	Downstream
4	A	G	33467818	rs733107678	0.648343264	CDKL2	Downstream
4	T	G	33467826	rs732410827	0.648343264	CDKL2	Downstream
4	A	G	33467843	rs313865738	0.648343264	CDKL2	Downstream
1	C	G	175512717	rs317821573	0.648270723	GTF3A	Downstream
1	G	A	175513963	rs314391248	0.648270723	GTF3A	Downstream
4	G	T	33493782	rs313666211	0.648270723	CDKL2	intron
4	T	G	33495472	rs741717056	0.648270723	CDKL2	intron
3	T	C	31343956	rs312536250	0.609596168	EIF2AK2	upstream
3	G	A	31357685	rs739745650	0.609596168	EIF2AK2	synonymus
3	C	T	31366120	rs315770213	0.609596168	EIF2AK2	Downstream
3	T	C	31368218	rs15316377	0.609596168	EIF2AK2	Downstream
4	A	G	33493307	rs315470838	0.609596168	CDKL2	intron
2	A	T	41818241	rs314563558	0.604207409	ENSGALG00000011530	3'UTR
3	C	T	31343240	rs15316353	0.604207409	EIF2AK2	upstream
4	C	T	33497229	rs738081525	0.599862657	CDKL2	intron
4	C	T	33497235	rs731987595	0.599862657	CDKL2	intron
4	A	G	33497236	rs735606937	0.599862657	CDKL2	intron
4	C	T	33494823	rs314807385	0.59239989	CDKL2	intron
3	T	A	31344390	rs315904023	0.581379566	EIF2AK2	upstream
4	G	T	33502355	rs740034101	0.576737516	CDKL2	Non-synonymous
4	C	A	33502356	rs732533563	0.576737516	CDKL2	Non-synonymous
4	A	G	33476272	_Variant_219	0.567555116	CDKL2	Downstream
3	G	C	16373300	rs731871955	0.563325921	PARP1	synonymus
3	T	A	31341975	rs15316351	0.560640358	EIF2AK2	upstream
3	G	C	31344290	rs741621760	0.560640358	EIF2AK2	upstream
3	T	C	31344877	rs739962808	0.552706721	EIF2AK2	upstream
4	T	G	33469670	rs312716751	0.552706721	CDKL2	Downstream
4	G	A	33494717	rs316894783	0.552706721	CDKL2	intron
3	C	T	31361567	rs316283228	0.545079858	EIF2AK2	intron
2	G	T	41818387	rs317905830	0.512841964	ENSGALG00000011530	3'UTR
2	C	T	41818852	rs313274976	0.512841964	ENSGALG00000011530	3'UTR
3	T	C	31345030	rs732518300	0.512841964	EIF2AK2	upstream

3	G	C	31345031	rs737382316	0.512841964	EIF2AK2	upstream
4	A	G	33474292	rs13514932	0.512841964	CDKL2	Downstream
4	A	G	33467586	rs313283547	0.510728528	CDKL2	Downstream
4	T	C	33467609	_Variant_167	0.510728528	CDKL2	Downstream
4	G	T	33469721	_Variant_186	0.510728528	CDKL2	Downstream
4	C	T	33475839	rs16388168	0.510728528	CDKL2	Downstream
4	C	T	33476223	rs733214590	0.510728528	CDKL2	Downstream
4	A	G	33476226	rs735304003	0.510728528	CDKL2	Downstream
3	C	T	31363660	rs733169603	0.489821133	EIF2AK2	intron
1	A	G	175511149	rs10724909	0.468498323	GTF3A	Downstream
4	A	G	33479165	rs313320742	0.468498323	CDKL2	intron
4	T	A	33492428	rs317608009	0.465853133	CDKL2	intron
1	G	T	175510993	rs740836983	0.462067031	GTF3A	Downstream
1	G	A	175511261	rs315405106	0.462067031	GTF3A	Downstream
4	C	G	33478041	rs317286975	0.462067031	CDKL2	intron
4	G	A	33517928	_Variant_310	0.462067031	CDKL2	upstream
4	T	A	33495862	rs314518660	0.461201906	CDKL2	intron
3	C	T	31344957	rs318119927	0.455906737	EIF2AK2	upstream
3	G	A	31341963	rs741262588	0.448286851	EIF2AK2	upstream
4	C	T	33470868	rs13514922	0.448286851	CDKL2	Downstream
4	G	A	33495760	rs16388204	0.448286851	CDKL2	intron
3	T	C	16391059	_Variant_42	0.447354508	PARP1	synonymus
4	A	T	33485914	rs731202310	0.447354508	CDKL2	intron
3	A	G	31372009	rs731333307	0.439230039	EIF2AK2	Downstream
3	G	A	31363574	_Variant_130	0.411218509	EIF2AK2	
4	C	T	33467450	rs16388132	0.407652908	CDKL2	Downstream
4	A	T	33467451	rs16388133	0.407652908	CDKL2	Downstream
4	T	C	33467459	rs314246404	0.407652908	CDKL2	Downstream
4	G	A	33474283	rs16388167	0.407652908	CDKL2	Downstream
4	T	C	33491172	rs14449622	0.407652908	CDKL2	intron
4	T	C	33493376	rs731578296	0.395482502	CDKL2	intron
4	G	A	33493377	rs733659860	0.395482502	CDKL2	intron
4	A	G	33494758	rs739526193	0.394928119	CDKL2	intron
4	T	A	33494936	rs735450656	0.394928119	CDKL2	intron
4	G	C	33494938	rs740092645	0.394928119	CDKL2	intron
4	G	A	33494943	rs315560906	0.394928119	CDKL2	intron
4	G	A	33494951	rs14449635	0.394928119	CDKL2	intron
4	G	A	33495012	rs317374389	0.394928119	CDKL2	intron
3	C	T	31361465	rs737548515	0.39392724	EIF2AK2	intron
4	T	C	33467755	rs313791190	0.389738178	CDKL2	Downstream
2	T	C	41818853	rs317398453	0.382772943	ENSGALG00000011530	3'UTR

2	C	T	41818862	rs740067322	0.382772943	ENSGALG00000011530	3'UTR
1	C	T	175509032	rs734991243	0.369363471	GTF3A	Downstream
3	C	T	31344371	rs314995835	0.369363471	EIF2AK2	upstream
1	G	A	175514176	rs15515614	0.366306687	GTF3A	Non-synonymous
3	G	A	31343776	rs313839287	0.348141634	EIF2AK2	upstream
3	T	C	31364297	rs315118162	0.348141634	EIF2AK2	3'UTR
4	T	C	33495900	rs741449832	0.348141634	CDKL2	intron
4	G	A	33467545	rs317049949	0.347085034	CDKL2	Downstream
4	G	A	33467605	rs315379307	0.347085034	CDKL2	Downstream
4	T	C	33467616	rs16388135	0.347085034	CDKL2	Downstream
4	A	G	33475812	rs312417454	0.347085034	CDKL2	Downstream
4	C	T	33487005	rs318193738	0.347085034	CDKL2	intron
1	C	T	175514504	rs315015517	0.344893284	GTF3A	intron
3	T	C	31363393	rs738170751	0.344893284	EIF2AK2	intron
4	A	G	33495973	rs733953895	0.344893284	CDKL2	intron
3	C	T	31360692	rs740709777	0.33966801	EIF2AK2	intron
3	C	T	31360694	rs733185813	0.33966801	EIF2AK2	intron
4	C	T	33493128	rs312383803	0.33966801	CDKL2	intron
4	T	C	33494322	rs16388198	0.338280054	CDKL2	intron
4	C	T	33494325	rs16388199	0.338280054	CDKL2	intron
3	G	A	31345108	rs315978358	0.335052526	EIF2AK2	upstream
4	C	T	33503864	rs738442004	0.315049155	CDKL2	synonymus
3	T	C	31363407	rs14336058	0.314774918	EIF2AK2	intron
3	C	T	16379935	rs735743845	0.310217403	PARP1	synonymus
3	A	G	16376233	rs317155120	0.305317452	PARP1	synonymus
4	A	G	33493612	rs316333834	0.275067959	CDKL2	intron
4	G	A	33470916	rs317051424	0.269949717	CDKL2	Downstream
3	T	C	31344069	rs316340316	0.266647911	EIF2AK2	upstream
3	G	A	31344183	rs732150839	0.266647911	EIF2AK2	upstream
3	G	A	31361932	rs314111928	0.266647911	EIF2AK2	intron
3	G	A	31364850	rs315403226	0.266647911	EIF2AK2	3'UTR
3	C	T	31369406	rs29008798	0.266647911	EIF2AK2	Downstream
4	A	G	33469809	rs314605290	0.24328231	CDKL2	Downstream
4	G	A	33469824	rs734779385	0.24328231	CDKL2	Downstream
4	A	C	33469833	rs317159563	0.24328231	CDKL2	Downstream
4	C	T	33469838	rs314345437	0.24328231	CDKL2	Downstream
3	A	C	31342044	rs733888504	0.210963789	EIF2AK2	upstream
4	C	T	33499666	rs317524366	0.210963789	CDKL2	synonymus
3	A	G	31357706	rs735694119	0.206415541	EIF2AK2	synonymus
4	T	C	33467611	rs732925591	0.206415541	CDKL2	Downstream
4	A	C	33469780	rs739222682	0.206415541	CDKL2	Downstream

4	T	G	33495223	rs312447565	0.206415541	CDKL2	intron
4	C	T	33495228	rs314737416	0.206415541	CDKL2	intron
4	T	C	33495378	rs16388202	0.206415541	CDKL2	intron
3	A	G	31343711	rs313385895	0.196687368	EIF2AK2	upstream
4	C	T	33480599	rs313444807	0.194472012	CDKL2	intron
4	G	A	33493464	rs312408641	0.194472012	CDKL2	intron
3	T	C	31347330	rs731630930	0.187058993	EIF2AK2	5'UTR
4	A	G	33513386	rs13514978	0.187058993	CDKL2	upstream
3	C	T	31364878	rs312995116	0.178800624	EIF2AK2	3'UTR
4	T	A	33475772	rs740987796	0.178800624	CDKL2	Downstream
2	A	G	41818873	rs732519590	0.173344643	ENSGALG00000011530	3'UTR
2	C	T	41817835	rs14169621	0.171797849	ENSGALG00000011530	3'UTR
3	G	C	16376684	rs733903178	0.171797849	PARP1	synonymus
3	C	G	31345071	rs734702484	0.171797849	EIF2AK2	upstream
3	T	C	31354410	rs740631179	0.171797849	EIF2AK2	synonymus
3	C	T	31364656	rs314705118	0.171797849	EIF2AK2	3'UTR
3	A	C	31364667	rs16249185	0.171797849	EIF2AK2	3'UTR
4	G	C	33474715	rs314616470	0.166186551	CDKL2	Downstream
4	G	A	33479805	rs14449610	0.166186551	CDKL2	intron
1	T	G	175514192	rs318191934	0.163844424	GTF3A	Non-synonymous
4	C	T	33468058	rs16388139	0.163844424	CDKL2	Downstream
4	G	A	33493276	rs316368835	0.163844424	CDKL2	intron
4	C	A	33495247	rs317622833	0.163844424	CDKL2	intron
2	C	A,T	41818166	rs315957945	0.157456394	ENSGALG00000011530	3'UTR
4	T	G	33493848	rs14449631	0.157456394	CDKL2	intron
3	C	A	31343483	rs15316358	0.155652693	EIF2AK2	upstream
2	A	G	41818777	rs316370658	0.151972259	ENSGALG00000011530	3'UTR
3	G	A	31344078	rs733930386	0.151972259	EIF2AK2	upstream
4	T	C	33469681	rs16388145	0.151972259	CDKL2	Downstream
4	C	T	33470837	rs315003445	0.145270828	CDKL2	Downstream
3	T	C	31341432	rs733845130	0.127151558	EIF2AK2	upstream
3	G	A	31341433	rs738674211	0.127151558	EIF2AK2	upstream
4	A	G	33493221	rs314963935	0.122460167	CDKL2	intron
4	A	G	33493230	rs315432224	0.122460167	CDKL2	intron
3	C	T	31343815	rs317106792	0.119906068	EIF2AK2	upstream
4	T	C	33503861	rs312502808	0.119906068	CDKL2	synonymus
1	C	A	175514403	rs317782905	0.107620563	GTF3A	intron
3	C	T	31343840	rs735028817	0.100662774	EIF2AK2	upstream
3	A	G	31343841	rs739849698	0.100662774	EIF2AK2	upstream
3	A	C	31343878	rs316062775	0.100662774	EIF2AK2	upstream
4	A	G	33493951	rs317772062	0.100636158	CDKL2	intron

3	C	T	16376263	rs313536476	0.081236683	PARP1	synonymus
3	G	A	31363955	rs741024440	0.081236683	EIF2AK2	Non-synonymous
4	C	T	33493721	rs314417085	0.076844738	CDKL2	intron
1	T	A	175508361	rs740668532	0.075700455	GTF3A	Downstream
1	A	G	175509055	rs317026062	0.075700455	GTF3A	Downstream
4	C	T	33513509	rs741559862	0.075700455	CDKL2	upstream
3	T	C	31363706	rs314414086	0.071851898	EIF2AK2	intron
3	T	C	31341987	rs314817541	0.062089336	EIF2AK2	upstream
3	A	T	31343797	rs317624632	0.041157534	EIF2AK2	upstream
3	G	A	31371650	rs737244408	0.028307771	EIF2AK2	Downstream
3	G	A	31371671	rs740708184	0.028307771	EIF2AK2	Downstream
3	G	A	31372000	rs29006318	0.028307771	EIF2AK2	Downstream
1	A	G	175514340	rs15515616	0	GTF3A	intron
3	A	G	31341897	rs314316320	0	EIF2AK2	upstream
3	C	T	31342903	rs738254600	0	EIF2AK2	upstream
3	C	T	31343068	rs732144254	0	EIF2AK2	upstream
3	C	G	31344482	rs314424715	0	EIF2AK2	upstream
3	G	A	31357754	rs739148168	0	EIF2AK2	synonymus
3	A	G	31361418	rs314594695	0	EIF2AK2	intron
3	A	G	31362425	rs316488701	0	EIF2AK2	synonymus
3	A	T	31364257	rs16249182	0	EIF2AK2	3'UTR
3	C	T	31364883	rs315722598	0	EIF2AK2	3'UTR
3	G	A	31365023	rs736973924	0	EIF2AK2	3'UTR
3	A	C	31365185	rs734169824	0	EIF2AK2	Downstream
3	G	A	31365992	rs16249190	0	EIF2AK2	Downstream
4	T	C	33469694	rs313476678	0	CDKL2	Downstream
4	G	A	33479743	rs313740581	0	CDKL2	intron
4	A	G	33479831	rs736518682	0	CDKL2	intron
4	C	T	33479832	rs741098137	0	CDKL2	intron
4	A	T	33479840	rs317979204	0	CDKL2	intron
4	G	A	33498590	rs14449642	0	CDKL2	synonymus
4	G	A	33513409	rs13514979	0	CDKL2	upstream
1	A	G	175511108	rs317085968		GTF3A	Downstream
1	T	C	175512687	rs10727308		GTF3A	Downstream
1	A	G	175514302	rs315834671		GTF3A	splice region, intron
3	G	C	31343867	rs16249164		EIF2AK2	upstream
3	T	C	31344152	rs314580361		EIF2AK2	upstream
3	A	G	31344294	rs317417657		EIF2AK2	upstream
3	C	G	31344297	rs732091112		EIF2AK2	upstream
3	G	A	31344312	rs734170475		EIF2AK2	upstream

3	C	T	31344317	rs739000982		EIF2AK2	upstream
3	T	C	31344709	rs316950561		EIF2AK2	upstream
3	T	C	31363462	rs14336059		EIF2AK2	intron
3	G	T	31365218	rs13722634		EIF2AK2	Downstream
3	G	A	31365231	rs13722635		EIF2AK2	Downstream
3	T	C	31365243	rs13722636		EIF2AK2	Downstream
3	T	C	31365292	rs13722637		EIF2AK2	Downstream
3	A	G	31365396	rs16249187		EIF2AK2	Downstream
3	T	C	31365829	rs16249189		EIF2AK2	Downstream
3	A	C	31365871	rs316179869		EIF2AK2	Downstream
3	C	T	31366074	rs16249191		EIF2AK2	Downstream
3	T	C	31371749	rs313731389		EIF2AK2	Downstream
4	C	T	33467360	rs16388131		CDKL2	Downstream
4	C	A	33467610	rs16388134		CDKL2	Downstream
4	C	T	33467635	rs16388136		CDKL2	Downstream
4	A	C	33467699	rs315669146		CDKL2	Downstream
4	G	A	33468027	rs16388138		CDKL2	Downstream
4	C	T	33470375	rs316832338		CDKL2	Downstream
4	G	A	33470631	rs312281095		CDKL2	Downstream
4	A	G	33474064	rs13514930		CDKL2	Downstream
4	A	G	33475776	rs13514937		CDKL2	Downstream
4	T	C	33475785	rs13514938		CDKL2	Downstream
4	G	A	33475841	rs16388169		CDKL2	Downstream
4	G	A	33475903	rs314017022		CDKL2	Downstream
4	C	T	33475905	rs739592716		CDKL2	Downstream
4	T	C	33476109	rs312602790		CDKL2	Downstream
4	A	G	33476144	rs733633129		CDKL2	Downstream
4	C	G	33476315	rs312842566		CDKL2	synonymus

Supplementary Table 15: eQTls identified in Ileum of low and high FCR broilers

chr	Ref	Alt	pos	snp_id	NegLog10 p-value	Gene	Location
7	A	C	13183971	rs317374719	1.918156122	ENSGALG00000008738	intron_variant
7	T	C	13187016	rs737034318	1.918156122	ENSGALG00000008738	intron_variant
7	C	T	13187020	rs315868610	1.918156122	ENSGALG00000008738	intron_variant
7	A	T	13187021	rs732851317	1.918156122	ENSGALG00000008738	intron_variant
7	C	T	13187051	rs736429536	1.918156122	ENSGALG00000008738	intron_variant
7	C	T	13184476	rs730965526	1.738597689	ENSGALG00000008738	intron_variant
7	C	T	13185159	rs737662720	1.730567189	ENSGALG00000008738	intron_variant
7	A	G	13186220	rs312536923	1.700390493	ENSGALG00000008738	intron_variant
7	C	T	13184778	rs16586532	1.53616176	ENSGALG00000008738	intron_variant
13	C	A	12231794	rs315635567	1.455746105	G3BP1	upstream_gene_variant
7	G	A	13186650	rs316285728	1.428758204	ENSGALG00000008738	intron_variant
7	T	G	13184339	rs738577016	1.366060143	ENSGALG00000008738	intron_variant
7	A	G	13184801	_Variant_385	1.366060143	ENSGALG00000008738	intron_variant
7	G	C	13186223	rs313809209	1.355360527	ENSGALG00000008738	intron_variant
13	C	G	12204487	rs741384794	1.350424807	G3BP1	intron_variant
7	G	C	13183243	rs313955853	1.275567297	ENSGALG00000008738	intron_variant
7	C	T	13181168	_Variant_316	1.269025468	ENSGALG00000008738	upstream_gene_variant
13	A	G	12204430	rs14061491	1.265045211	G3BP1	intron_variant
13	A	C	12226065	_Variant_58	1.226605337	G3BP1	upstream_gene_variant
7	C	T	13187005	rs316314873	1.184180377	ENSGALG00000008738	intron_variant
13	T	G	12204155	_Variant_25	1.175236095	G3BP1	intron_variant
13	G	A	12204917	rs313082555	1.09246113	G3BP1	intron_variant
7	A	T	13187244	_Variant_434	1.09246113	ENSGALG00000008738	intron_variant
13	A	C	12228497	rs317037679	1.05450697	G3BP1	upstream_gene_variant
7	A	G	13182939	_Variant_335	1.05450697	ENSGALG00000008738	intron_variant
13	T	A	12228659	rs741748532	1.025652722	G3BP1	upstream_gene_variant
13	T	A	12228661	rs316591800	1.025652722	G3BP1	upstream_gene_variant
7	C	T	13192824	rs318101770	1.025652722	ENSGALG00000008738	downstream_gene_variant
13	G	A	12229490	rs736780578	1.015919651	G3BP1	upstream_gene_variant
3	G	A	105083969	rs312318809	1.015919651	CENPO	downstream_gene_variant
7	C	T	13182591	rs317078358	1.010150071	ENSGALG00000008738	intron_variant
7	A	G	13185714	rs738511531	1.010150071	ENSGALG00000008738	intron_variant
7	G	A	13182363	rs315045117	0.982117511	ENSGALG00000008738	intron_variant
13	T	A	12203999	_Variant_23	0.951334318	G3BP1	intron_variant
13	G	A	12228738	_Variant_84	0.951334318	G3BP1	upstream_gene_variant
13	C	A	12228802	rs14997804	0.951334318	G3BP1	upstream_gene_variant
13	A	G	12228803	rs14997805	0.951334318	G3BP1	upstream_gene_variant
7	C	T	13177761	_Variant_311	0.940615934	ENSGALG00000008738	upstream_gene_variant
7	C	T	13177770	_Variant_312	0.940615934	ENSGALG00000008738	upstream_gene_variant

7	A	C	13177856	rs733513890	0.940615934	ENSGALG00000008738	upstream_gene_variant
7	G	A	13184241	rs15847038	0.940615934	ENSGALG00000008738	intron_variant
7	A	G	13184878	_Variant_390	0.940615934	ENSGALG00000008738	synonymous_variant
7	A	G	13185426	rs14609451	0.940615934	ENSGALG00000008738	intron_variant
7	G	A	13185822	rs317609878	0.940615934	ENSGALG00000008738	intron_variant
7	G	A	13189265	rs312737397	0.940615934	ENSGALG00000008738	downstream_gene_variant
7	G	A	13190408	rs731918051	0.940615934	ENSGALG00000008738	downstream_gene_variant
7	C	T	13190486	rs316915036	0.940615934	ENSGALG00000008738	downstream_gene_variant
7	T	C	13190505	rs316452607	0.940615934	ENSGALG00000008738	downstream_gene_variant
7	T	C	13190520	rs316973452	0.940615934	ENSGALG00000008738	downstream_gene_variant
7	A	G	13190628	rs318122505	0.940615934	ENSGALG00000008738	downstream_gene_variant
7	G	T	13190636	rs317303614	0.940615934	ENSGALG00000008738	downstream_gene_variant
7	C	T	13191222	rs313736983	0.940615934	ENSGALG00000008738	downstream_gene_variant
7	G	A	13191265	rs315053490	0.940615934	ENSGALG00000008738	downstream_gene_variant
7	C	T	13191270	rs314884706	0.940615934	ENSGALG00000008738	downstream_gene_variant
7	A	T	13191335	rs16586552	0.940615934	ENSGALG00000008738	downstream_gene_variant
7	C	T	13191494	rs316279693	0.940615934	ENSGALG00000008738	downstream_gene_variant
7	T	C	13192678	rs317615937	0.940615934	ENSGALG00000008738	downstream_gene_variant
7	C	T	13193331	rs733097745	0.940615934	ENSGALG00000008738	downstream_gene_variant
7	T	C	13183526	rs315767605	0.937110171	ENSGALG00000008738	intron_variant
13	C	T	12229146	_Variant_95	0.929587896	G3BP1	upstream_gene_variant
7	C	T	13172561	rs316310234	0.897461823	ENSGALG00000008738	upstream_gene_variant
7	G	A	13192832	rs317481659	0.857636384	ENSGALG00000008738	downstream_gene_variant
13	T	C	12203229	rs316133931	0.847059963	G3BP1	intron_variant
13	G	C	12209103	rs313564023	0.847059963	G3BP1	synonymous_variant
13	T	C	12228098	rs317030568	0.847059963	G3BP1	upstream_gene_variant
3	A	G	105086136	rs14409178	0.847059963	CENPO	3_prime_UTR_variant
3	T	C	105086204	rs313207176	0.847059963	CENPO	3_prime_UTR_variant
7	T	C	13183022	rs313629155	0.847059963	ENSGALG00000008738	synonymous_variant
7	T	G	13184025	rs316922111	0.847059963	ENSGALG00000008738	intron_variant
7	G	A	13186612	rs738655432	0.847059963	ENSGALG00000008738	intron_variant
7	G	T	13186619	rs315333862	0.847059963	ENSGALG00000008738	intron_variant
7	A	G	13186718	rs316065091	0.847059963	ENSGALG00000008738	intron_variant
7	C	A	13188355	rs16586542	0.847059963	ENSGALG00000008738	downstream_gene_variant
7	C	T	13188716	rs317515891	0.847059963	ENSGALG00000008738	downstream_gene_variant
7	T	G	13188823	rs318033386	0.847059963	ENSGALG00000008738	downstream_gene_variant
7	A	G	13189120	rs16586545	0.847059963	ENSGALG00000008738	downstream_gene_variant
7	G	T	13189144	rs16586546	0.847059963	ENSGALG00000008738	downstream_gene_variant
13	C	T	12226104	rs14061542	0.843951128	G3BP1	upstream_gene_variant
3	C	T	105086970	rs317659921	0.843951128	CENPO	3_prime_UTR_variant
3	G	A	105087088	rs312776651	0.843951128	CENPO	3_prime_UTR_variant

7	A	G	13183426	rs315279623	0.819604589	ENSGALG00000008738	intron_variant
13	C	T	12229068	rs739162507	0.762067084	G3BP1	upstream_gene_variant
7	C	G	13182819	rs318123197	0.762067084	ENSGALG00000008738	intron_variant
7	C	T	13182826	rs312370064	0.762067084	ENSGALG00000008738	intron_variant
7	A	G	13186391	rs313763367	0.762067084	ENSGALG00000008738	intron_variant
13	A	G	12228577	rs315531983	0.735503077	G3BP1	upstream_gene_variant
7	T	C	13191666	_Variant_488	0.720264031	ENSGALG00000008738	downstream_gene_variant
7	G	T	13185756	_Variant_396	0.71789328	ENSGALG00000008738	intron_variant
7	C	T	13185762	_Variant_397	0.71789328	ENSGALG00000008738	intron_variant
7	G	A	13185779	_Variant_398	0.71789328	ENSGALG00000008738	intron_variant
13	T	A	12228620	rs14997801	0.717186237	G3BP1	upstream_gene_variant
7	G	A	13182174	_Variant_318	0.717186237	ENSGALG00000008738	synonymous_variant
3	T	C	105090388	_Variant_146	0.713382367	CENPO	synonymous_variant
13	G	A	12229126	rs740919315	0.673882259	G3BP1	upstream_gene_variant
5	C	T	49385873	_Variant_176	0.673882259	HSP90AA1	downstream_gene_variant
5	G	A	49385879	rs317486660	0.673882259	HSP90AA1	downstream_gene_variant
7	G	A	13182841	rs312555759	0.673882259	ENSGALG00000008738	intron_variant
13	A	G	12231487	_Variant_108	0.65942818	G3BP1	upstream_gene_variant
5	A	G	49384001	rs315024783	0.648343264	HSP90AA1	downstream_gene_variant
3	A	G	105087014	rs314307565	0.644372624	CENPO	3_prime_UTR_variant
7	C	T	13183471	rs737527690	0.621396344	ENSGALG00000008738	intron_variant
7	A	C	13184429	rs741360799	0.616963137	ENSGALG00000008738	intron_variant
7	C	T	13173862	rs316994610	0.609596168	ENSGALG00000008738	upstream_gene_variant
13	T	G	12228603	rs14997800	0.601230181	G3BP1	upstream_gene_variant
3	T	C	105084723	rs740846282	0.601230181	CENPO	downstream_gene_variant
7	G	A	13186894	rs317173199	0.601230181	ENSGALG00000008738	intron_variant
13	T	C	12206046	rs14061496	0.59239989	G3BP1	intron_variant
7	G	A	13178599	_Variant_314	0.59239989	ENSGALG00000008738	upstream_gene_variant
7	C	T	13184844	_Variant_388	0.59239989	ENSGALG00000008738	intron_variant
7	T	C	13184847	_Variant_389	0.59239989	ENSGALG00000008738	intron_variant
7	T	C	13184896	rs314332288	0.59239989	ENSGALG00000008738	synonymous_variant
7	C	T	13186485	rs315063847	0.59239989	ENSGALG00000008738	intron_variant
7	T	C	13186496	rs314891517	0.59239989	ENSGALG00000008738	intron_variant
7	G	C	13188747	rs316626027	0.59239989	ENSGALG00000008738	downstream_gene_variant
7	C	T	13193258	rs314142767	0.59239989	ENSGALG00000008738	downstream_gene_variant
7	C	T	13193343	rs736596526	0.59239989	ENSGALG00000008738	downstream_gene_variant
5	T	G	49396765	rs317426857	0.581379566	HSP90AA1	upstream_gene_variant
7	C	T	13184729	rs733294219	0.580256582	ENSGALG00000008738	intron_variant
13	A	C	12200602	rs316733646	0.576737516	G3BP1	downstream_gene_variant
13	C	T	12203319	_Variant_20	0.576737516	G3BP1	intron_variant
13	C	T	12203420	rs736706324	0.576737516	G3BP1	intron_variant

3	G	A	105087312	_Variant_142	0.576737516	CENPO	synonymous_variant
3	G	C	105090985	rs16338596	0.569618263	CENPO	synonymous_variant
3	A	C	105091830	rs13525116	0.569618263	CENPO	synonymous_variant
13	A	C	12205583	rs13547776	0.567555116	G3BP1	intron_variant
7	A	G	13184158	rs318038515	0.567555116	ENSGALG00000008738	intron_variant
13	G	A	12227811	rs312849596	0.560640358	G3BP1	upstream_gene_variant
13	T	C	12212380	rs14061506	0.557063636	G3BP1	intron_variant
5	G	A	49387401	rs736960234	0.557063636	HSP90AA1	downstream_gene_variant
5	C	A	49387404	rs318104139	0.557063636	HSP90AA1	downstream_gene_variant
13	C	T	12227647	rs317532891	0.552706721	G3BP1	upstream_gene_variant
7	T	C	13186724	rs731809026	0.552706721	ENSGALG00000008738	intron_variant
7	C	T	13186913	rs736032610	0.552706721	ENSGALG00000008738	intron_variant
7	G	A	13186971	rs317617765	0.552706721	ENSGALG00000008738	intron_variant
13	A	G	12201635	rs734683942	0.510728528	G3BP1	downstream_gene_variant
13	G	C	12201926	rs737908733	0.510728528	G3BP1	downstream_gene_variant
13	G	A	12201959	rs314755576	0.510728528	G3BP1	downstream_gene_variant
13	A	G	12205181	rs15702617	0.510728528	G3BP1	synonymous_variant
3	G	A	105086404	rs315048462	0.510728528	CENPO	3_prime_UTR_variant
3	T	C	105086985	rs737274277	0.489821133	CENPO	3_prime_UTR_variant
3	T	C	105090943	_Variant_149	0.485204123	CENPO	synonymous_variant
3	G	A	105091993	rs313388939	0.465853133	CENPO	5_prime_UTR_variant
13	C	A	12204804	rs313456461	0.462067031	G3BP1	intron_variant
13	T	C	12205444	rs731312872	0.462067031	G3BP1	intron_variant
7	T	C	13186325	rs314211831	0.462067031	ENSGALG00000008738	intron_variant
13	C	T	12231525	rs735984625	0.461201906	G3BP1	upstream_gene_variant
13	T	A	12211255	rs316980898	0.455906737	G3BP1	intron_variant
7	T	C	13183479	rs740830906	0.448286851	ENSGALG00000008738	intron_variant
7	G	A	13183513	rs315062118	0.448286851	ENSGALG00000008738	intron_variant
7	G	A	13184671	_Variant_375	0.448286851	ENSGALG00000008738	intron_variant
7	G	A	13186083	rs739525900	0.448286851	ENSGALG00000008738	intron_variant
3	T	C	105086633	rs13525109	0.447354508	CENPO	3_prime_UTR_variant
5	T	C	49383924	rs15725926	0.447354508	HSP90AA1	downstream_gene_variant
7	T	C	13183264	rs739390876	0.447354508	ENSGALG00000008738	intron_variant
7	A	G	13183286	rs736568427	0.447354508	ENSGALG00000008738	intron_variant
7	T	C	13183301	rs16586530	0.447354508	ENSGALG00000008738	intron_variant
7	G	A	13187081	rs739973619	0.447354508	ENSGALG00000008738	intron_variant
7	C	A	13190345	rs15847040	0.447354508	ENSGALG00000008738	downstream_gene_variant
13	G	A	12205847	rs741417979	0.412877211	G3BP1	intron_variant
7	C	G	13182566	rs736769783	0.412877211	ENSGALG00000008738	intron_variant
7	T	G	13182567	rs741612993	0.412877211	ENSGALG00000008738	intron_variant
13	T	C	12201234	rs736881025	0.394928119	G3BP1	downstream_gene_variant

3	G	A	105086679	rs14409180	0.394928119	CENPO	3_prime_UTR_variant
7	T	G	13183270	rs733102434	0.394928119	ENSGALG00000008738	intron_variant
7	G	C	13186565	rs315408485	0.394928119	ENSGALG00000008738	intron_variant
13	T	C	12231477	_Variant_107	0.389738178	G3BP1	upstream_gene_variant
13	C	A	12202064	rs312735652	0.366306687	G3BP1	downstream_gene_variant
13	G	A	12228153	rs313143341	0.366306687	G3BP1	upstream_gene_variant
7	G	A	13177740	rs315980331	0.366306687	ENSGALG00000008738	upstream_gene_variant
13	G	A	12202025	_Variant_13	0.35163148	G3BP1	downstream_gene_variant
7	C	T	13187469	rs313320384	0.35163148	ENSGALG00000008738	3_prime_UTR_variant
7	T	G	13187888	rs316072144	0.35163148	ENSGALG00000008738	downstream_gene_variant
7	C	A	13189457	rs16586547	0.35163148	ENSGALG00000008738	downstream_gene_variant
7	T	C	13189458	rs16586548	0.35163148	ENSGALG00000008738	downstream_gene_variant
7	A	G	13189817	rs314934181	0.35163148	ENSGALG00000008738	downstream_gene_variant
7	G	A	13193130	rs313451213	0.35163148	ENSGALG00000008738	downstream_gene_variant
13	G	A	12205987	rs14061494	0.348141634	G3BP1	intron_variant
13	A	T	12227792	rs313762912	0.347085034	G3BP1	upstream_gene_variant
5	A	T	49389330	rs731985471	0.347085034	HSP90AA1	missense_variant
3	T	C	105084290	rs731643809	0.344893284	CENPO	downstream_gene_variant
3	T	C	105089336	_Variant_144	0.344893284	CENPO	synonymous_variant
3	C	T	105089366	rs14409187	0.344893284	CENPO	synonymous_variant
3	C	T	105090848	rs738836886	0.344893284	CENPO	missense_variant
13	G	A	12231179	rs313348283	0.33966801	G3BP1	upstream_gene_variant
5	T	C	49385100	rs740503686	0.33966801	HSP90AA1	downstream_gene_variant
5	C	T	49385803	_Variant_174	0.33966801	HSP90AA1	downstream_gene_variant
7	G	T	13182422	rs16586528	0.33966801	ENSGALG00000008738	intron_variant
7	C	T	13182698	rs317684007	0.338280054	ENSGALG00000008738	intron_variant
7	C	T	13183811	rs14609447	0.338280054	ENSGALG00000008738	intron_variant
13	A	G	12204599	rs740491705	0.335052526	G3BP1	intron_variant
7	G	A	13184059	rs14609450	0.335052526	ENSGALG00000008738	intron_variant
3	T	C	105087339	rs16338582	0.325499423	CENPO	synonymous_variant
5	T	A	49389367	_Variant_218	0.325499423	HSP90AA1	intron_variant
5	C	A	49389368	_Variant_219	0.325499423	HSP90AA1	intron_variant
5	G	C	49389378	rs741557487	0.325499423	HSP90AA1	intron_variant
5	G	T	49389402	rs735268288	0.325499423	HSP90AA1	intron_variant
5	G	A	49389406	rs738732742	0.325499423	HSP90AA1	intron_variant
5	T	A	49389446	rs735139131	0.325499423	HSP90AA1	missense_variant
7	C	G	13184695	rs314546881	0.315049155	ENSGALG00000008738	intron_variant
13	C	A	12228294	rs732331694	0.314774918	G3BP1	upstream_gene_variant
13	G	A	12228295	rs737156415	0.314774918	G3BP1	upstream_gene_variant
13	A	G	12228304	rs740537663	0.314774918	G3BP1	upstream_gene_variant
5	A	G	49384656	_Variant_166	0.314774918	HSP90AA1	downstream_gene_variant

5	C	T	49392896	rs317414882	0.314774918	HSP90AA1	intron_variant
13	G	C	12212633	rs313081782	0.310217403	G3BP1	intron_variant
13	G	A	12227934	rs738882439	0.310217403	G3BP1	upstream_gene_variant
13	C	T	12203273	rs740287981	0.305317452	G3BP1	intron_variant
13	C	T	12228004	rs313011460	0.305317452	G3BP1	upstream_gene_variant
13	G	A	12204903	_Variant_37	0.282957841	G3BP1	intron_variant
5	C	T	49385051	_Variant_170	0.269949717	HSP90AA1	downstream_gene_variant
13	T	C	12229325	rs14997813	0.266647911	G3BP1	upstream_gene_variant
5	C	T	49383918	rs740603577	0.266647911	HSP90AA1	downstream_gene_variant
5	A	C	49388290	rs15725974	0.266647911	HSP90AA1	intron_variant
5	G	A	49389204	rs733456186	0.266647911	HSP90AA1	missense_variant
5	C	T	49389237	_Variant_211	0.266647911	HSP90AA1	missense_variant
5	C	A	49389287	rs734979139	0.266647911	HSP90AA1	synonymous_variant
5	G	A	49389470	rs737573963	0.266647911	HSP90AA1	synonymous_variant
5	C	A	49389588	rs741506174	0.266647911	HSP90AA1	intron_variant
5	A	C	49391209	rs733454904	0.266647911	HSP90AA1	intron_variant
5	T	C	49391931	rs14546543	0.266647911	HSP90AA1	intron_variant
5	G	A	49391932	rs14546544	0.266647911	HSP90AA1	intron_variant
5	T	C	49393935	_Variant_300	0.266647911	HSP90AA1	5_prime_UTR_variant
5	C	A	49393939	rs737831046	0.266647911	HSP90AA1	5_prime_UTR_variant
7	T	C	13184166	_Variant_360	0.266647911	ENSGALG00000008738	intron_variant
7	T	C	13184767	rs736070198	0.266647911	ENSGALG00000008738	intron_variant
7	C	T	13184815	_Variant_386	0.266647911	ENSGALG00000008738	intron_variant
7	C	T	13186563	rs737876392	0.266647911	ENSGALG00000008738	intron_variant
7	T	G	13188345	_Variant_441	0.266647911	ENSGALG00000008738	downstream_gene_variant
7	A	G	13188481	_Variant_444	0.266647911	ENSGALG00000008738	downstream_gene_variant
7	G	A	13188649	_Variant_446	0.266647911	ENSGALG00000008738	downstream_gene_variant
7	A	G	13190676	rs317436133	0.266647911	ENSGALG00000008738	downstream_gene_variant
7	C	T	13191347	rs315881126	0.266647911	ENSGALG00000008738	downstream_gene_variant
7	T	G	13191690	_Variant_489	0.266647911	ENSGALG00000008738	downstream_gene_variant
7	T	C	13193120	rs15847047	0.266647911	ENSGALG00000008738	downstream_gene_variant
7	A	G	13193182	rs15847049	0.266647911	ENSGALG00000008738	downstream_gene_variant
13	T	C	12205676	rs14061493	0.255608647	G3BP1	synonymous_variant
7	C	G	13182888	rs731085320	0.255608647	ENSGALG00000008738	intron_variant
7	T	C	13182899	rs738529475	0.255608647	ENSGALG00000008738	intron_variant
7	T	C	13182964	_Variant_337	0.255608647	ENSGALG00000008738	intron_variant
3	G	A	105086845	rs16338579	0.253222111	CENPO	3_prime_UTR_variant
3	T	C	105086862	rs16338580	0.253222111	CENPO	3_prime_UTR_variant
3	C	T	105090459	rs316512832	0.24328231	CENPO	missense_variant
5	G	C	49385824	_Variant_175	0.210963789	HSP90AA1	downstream_gene_variant
7	C	T	13186865	rs316196522	0.210963789	ENSGALG00000008738	splice_region_variant,intron_variant

13	T	A	12204753	rs315475692	0.208480162	G3BP1	intron_variant
13	A	G	12228058	rs317500282	0.206415541	G3BP1	upstream_gene_variant
5	A	G	49387521	rs734715072	0.206415541	HSP90AA1	downstream_gene_variant
5	C	T	49387816	rs314485553	0.206415541	HSP90AA1	downstream_gene_variant
5	G	A	49388343	_Variant_192	0.206415541	HSP90AA1	intron_variant
5	C	T	49388411	_Variant_196	0.206415541	HSP90AA1	intron_variant
5	C	T	49388413	_Variant_197	0.206415541	HSP90AA1	intron_variant
5	A	C	49388426	rs735741264	0.206415541	HSP90AA1	intron_variant
5	C	T	49388694	rs740976636	0.206415541	HSP90AA1	synonymous_variant
5	T	C	49388795	rs736761990	0.206415541	HSP90AA1	intron_variant
5	T	G	49388799	rs738215441	0.206415541	HSP90AA1	intron_variant
5	A	G	49388822	rs739289475	0.206415541	HSP90AA1	intron_variant
5	C	T	49388848	rs741565463	0.206415541	HSP90AA1	intron_variant
5	T	C	49388987	rs740841031	0.206415541	HSP90AA1	synonymous_variant
5	T	C	49389071	rs738048759	0.206415541	HSP90AA1	synonymous_variant
5	C	A	49389241	rs741739607	0.206415541	HSP90AA1	missense_variant
5	C	T	49389273	rs732874649	0.206415541	HSP90AA1	splice_donor_variant
5	A	G	49389544	rs734001621	0.206415541	HSP90AA1	intron_variant
5	G	A	49389724	_Variant_235	0.206415541	HSP90AA1	intron_variant
5	T	C	49389759	_Variant_236	0.206415541	HSP90AA1	missense_variant
5	T	C	49389768	_Variant_237	0.206415541	HSP90AA1	missense_variant
5	T	C	49389770	_Variant_238	0.206415541	HSP90AA1	synonymous_variant
5	T	G	49389776	_Variant_239	0.206415541	HSP90AA1	synonymous_variant
5	A	C	49389777	_Variant_240	0.206415541	HSP90AA1	missense_variant
5	T	A	49389781	_Variant_241	0.206415541	HSP90AA1	missense_variant
5	G	A	49389814	_Variant_244	0.206415541	HSP90AA1	missense_variant
5	C	G	49390216	rs739609697	0.206415541	HSP90AA1	intron_variant
5	G	C	49390229	rs736235817	0.206415541	HSP90AA1	intron_variant
5	T	C	49390232	rs736969303	0.206415541	HSP90AA1	intron_variant
5	T	C	49390309	_Variant_248	0.206415541	HSP90AA1	intron_variant
5	G	A	49390310	_Variant_249	0.206415541	HSP90AA1	intron_variant
5	T	C	49390316	rs733580582	0.206415541	HSP90AA1	intron_variant
5	T	C	49390339	rs734297659	0.206415541	HSP90AA1	intron_variant
5	C	T	49390508	_Variant_254	0.206415541	HSP90AA1	intron_variant
5	T	G	49390549	rs732651243	0.206415541	HSP90AA1	intron_variant
5	C	T	49391572	rs314794801	0.206415541	HSP90AA1	intron_variant
5	C	T	49391759	rs313747236	0.206415541	HSP90AA1	intron_variant
5	C	T	49395925	rs314980448	0.206415541	HSP90AA1	upstream_gene_variant
7	C	T	13178613	rs312982970	0.206415541	ENSGALG00000008738	upstream_gene_variant
7	G	A	13188848	rs731981524	0.206415541	ENSGALG00000008738	downstream_gene_variant
7	T	G	13191452	rs740789233	0.206415541	ENSGALG00000008738	downstream_gene_variant

13	C	T	12204628	rs737682858	0.198881027	G3BP1	intron_variant
5	A	G	49393044	_Variant_293	0.198881027	HSP90AA1	intron_variant
5	A	C	49393050	_Variant_294	0.198881027	HSP90AA1	intron_variant
5	A	G	49385037	_Variant_169	0.196687368	HSP90AA1	downstream_gene_variant
7	T	G	13184496	rs738525174	0.196687368	ENSGALG00000008738	intron_variant
7	C	A	13184692	_Variant_378	0.196687368	ENSGALG00000008738	intron_variant
7	A	G	13186319	rs16586539	0.196687368	ENSGALG00000008738	intron_variant
7	T	C	13186353	rs16586540	0.196687368	ENSGALG00000008738	intron_variant
5	T	C	49384391	_Variant_164	0.187058993	HSP90AA1	downstream_gene_variant
5	G	A	49384410	rs14546540	0.187058993	HSP90AA1	downstream_gene_variant
5	C	T	49385069	_Variant_171	0.187058993	HSP90AA1	downstream_gene_variant
3	G	A	105086350	rs16338576	0.178800624	CENPO	3_prime_UTR_variant
3	G	A	105086365	rs13525107	0.178800624	CENPO	3_prime_UTR_variant
3	C	G	105086367	rs13525108	0.178800624	CENPO	3_prime_UTR_variant
3	A	G	105086493	_Variant_125	0.178800624	CENPO	3_prime_UTR_variant
3	G	C	105086617	rs740841155	0.178800624	CENPO	3_prime_UTR_variant
3	T	C	105099690	_Variant_153	0.173344643	CENPO	upstream_gene_variant
3	G	A	105099752	rs314268384	0.173344643	CENPO	upstream_gene_variant
5	T	C	49387443	_Variant_180	0.173344643	HSP90AA1	downstream_gene_variant
5	A	G	49387456	_Variant_182	0.173344643	HSP90AA1	downstream_gene_variant
13	A	C	12205828	rs736678229	0.171797849	G3BP1	intron_variant
13	C	T	12206422	_Variant_50	0.171797849	G3BP1	intron_variant
13	C	G	12229939	rs731611359	0.171797849	G3BP1	upstream_gene_variant
3	A	G	105085670	rs10728557	0.171797849	CENPO	downstream_gene_variant
5	T	C	49384247	rs14546538	0.166186551	HSP90AA1	downstream_gene_variant
13	T	C	12201930	rs312895013	0.163844424	G3BP1	downstream_gene_variant
13	G	A	12203157	rs315630742	0.163844424	G3BP1	splice_region_variant,intron_variant
13	T	C	12228157	rs317751883	0.163844424	G3BP1	upstream_gene_variant
13	G	A	12228171	rs314735397	0.163844424	G3BP1	upstream_gene_variant
5	G	A	49388226	rs315530665	0.163844424	HSP90AA1	synonymous_variant
5	A	G	49389797	_Variant_242	0.163844424	HSP90AA1	synonymous_variant
5	G	A	49389808	_Variant_243	0.163844424	HSP90AA1	synonymous_variant
13	G	A	12229364	rs14997814	0.155652693	G3BP1	upstream_gene_variant
5	A	G	49388184	rs316992600	0.155652693	HSP90AA1	synonymous_variant
5	G	C	49388314	rs315063094	0.155652693	HSP90AA1	intron_variant
5	C	T	49388851	rs312465611	0.155652693	HSP90AA1	intron_variant
7	A	C	13184414	rs731764557	0.155652693	ENSGALG00000008738	intron_variant
7	C	A	13184420	rs733857446	0.155652693	ENSGALG00000008738	intron_variant
7	C	T	13189000	rs733557440	0.155652693	ENSGALG00000008738	downstream_gene_variant
13	G	A	12227605	rs3137050	0.151972259	G3BP1	upstream_gene_variant
5	A	C	49383917	rs15725924	0.151972259	HSP90AA1	downstream_gene_variant

5	G	A	49388354	_Variant_193	0.151972259	HSP90AA1	intron_variant
7	T	C	13186902	rs316661698	0.151972259	ENSGALG00000008738	intron_variant
7	C	T	13189579	rs313502613	0.151972259	ENSGALG00000008738	downstream_gene_variant
13	G	A	12228594	rs317837699	0.148046063	G3BP1	upstream_gene_variant
7	C	T	13175330	rs317686448	0.148046063	ENSGALG00000008738	upstream_gene_variant
13	A	C	12228248	rs314617514	0.145270828	G3BP1	upstream_gene_variant
13	A	G	12228771	_Variant_86	0.145270828	G3BP1	upstream_gene_variant
13	C	T	12229102	rs14997809	0.145270828	G3BP1	upstream_gene_variant
13	A	T	12229106	rs14997810	0.145270828	G3BP1	upstream_gene_variant
13	A	T	12229108	rs14997811	0.145270828	G3BP1	upstream_gene_variant
5	T	C	49385091	_Variant_172	0.127151558	HSP90AA1	downstream_gene_variant
5	C	T	49383755	rs14546537	0.122460167	HSP90AA1	downstream_gene_variant
5	G	A	49393021	_Variant_292	0.122460167	HSP90AA1	intron_variant
5	C	G	49393063	_Variant_295	0.122460167	HSP90AA1	intron_variant
5	C	T	49393068	_Variant_296	0.122460167	HSP90AA1	intron_variant
5	A	G	49393077	_Variant_298	0.122460167	HSP90AA1	intron_variant
7	C	A	13183467	rs314380271	0.122460167	ENSGALG00000008738	intron_variant
13	T	A	12206117	_Variant_47	0.1215964	G3BP1	intron_variant
13	T	C	12206135	_Variant_48	0.1215964	G3BP1	intron_variant
13	C	T	12206144	_Variant_49	0.1215964	G3BP1	intron_variant
5	T	C	49385014	_Variant_168	0.119906068	HSP90AA1	downstream_gene_variant
3	C	T	105085147	rs316026866	0.107620563	CENPO	downstream_gene_variant
5	C	T	49383546	rs314637530	0.107620563	HSP90AA1	downstream_gene_variant
13	C	T	12228042	rs736054261	0.100662774	G3BP1	upstream_gene_variant
5	G	A	49392673	rs16508340	0.100662774	HSP90AA1	intron_variant
5	C	T	49392753	rs731102441	0.100662774	HSP90AA1	intron_variant
13	C	T	12230807	rs317728084	0.100636158	G3BP1	upstream_gene_variant
13	G	C	12203940	rs13547774	0.081236683	G3BP1	intron_variant
13	G	A	12204629	rs732212185	0.080557875	G3BP1	intron_variant
13	C	A	12203258	rs314886487	0.076844738	G3BP1	intron_variant
13	A	T	12229060	rs14997808	0.076844738	G3BP1	upstream_gene_variant
7	G	C	13182927	_Variant_333	0.076844738	ENSGALG00000008738	intron_variant
7	A	G	13182928	_Variant_334	0.076844738	ENSGALG00000008738	intron_variant
5	C	T	49401663	rs315460875	0.075700455	HSP90AA1	upstream_gene_variant
5	G	A	49383871	rs314281605	0.062089336	HSP90AA1	downstream_gene_variant
5	G	T	49396691	_Variant_303	0.062089336	HSP90AA1	upstream_gene_variant
5	T	G	49396696	_Variant_304	0.062089336	HSP90AA1	upstream_gene_variant
7	T	C	13183830	rs14609448	0.061851596	ENSGALG00000008738	intron_variant
13	A	G	12206504	rs316699372	0.051253114	G3BP1	intron_variant
7	G	A	13182625	rs317050774	0.049491483	ENSGALG00000008738	intron_variant
13	G	A	12231021	rs313959900	0.046352214	G3BP1	upstream_gene_variant

13	G	C	12231059	rs13547784	0.046352214	G3BP1	upstream_gene_variant
5	T	C	49393094	rs312751115	0.042614301	HSP90AA1	intron_variant
13	T	C	12228745	_Variant_85	0.041157534	G3BP1	upstream_gene_variant
7	A	G	13182942	_Variant_336	0.041157534	ENSGALG00000008738	intron_variant
7	A	G	13187189	_Variant_433	0.030447147	ENSGALG00000008738	intron_variant
13	G	A	12211496	rs734702298	0.027107458	G3BP1	intron_variant
7	C	T	13183477	rs734010369	0.021747088	ENSGALG00000008738	intron_variant
13	C	T	12200831	_Variant_2	0	G3BP1	downstream_gene_variant
13	A	G	12200859	rs314846237	0	G3BP1	downstream_gene_variant
13	C	G	12201075	rs313740279	0	G3BP1	downstream_gene_variant
13	G	A	12201540	_Variant_7	0	G3BP1	downstream_gene_variant
13	A	G	12202620	_Variant_15	0	G3BP1	3_prime_UTR_variant
13	T	C	12211810	rs312929391	0	G3BP1	intron_variant
13	C	T	12227668	rs738353940	0	G3BP1	upstream_gene_variant
13	T	C	12229365	rs734804414	0	G3BP1	upstream_gene_variant
3	A	G	105086177	rs14409179	0	CENPO	3_prime_UTR_variant
3	G	A	105086570	rs16338578	0	CENPO	3_prime_UTR_variant
3	A	G	105086699	rs312587083	0	CENPO	3_prime_UTR_variant
3	T	C	105087122	rs312999260	0	CENPO	3_prime_UTR_variant
5	C	T	49384146	rs737815496	0	HSP90AA1	downstream_gene_variant
5	G	A,T	49387495	_Variant_183	0	HSP90AA1	downstream_gene_variant
5	G	A	49388280	rs15725972	0	HSP90AA1	splice_region_variant,intron_variant
5	T	C	49388306	rs312369666	0	HSP90AA1	intron_variant
5	T	A	49388364	_Variant_194	0	HSP90AA1	intron_variant
5	G	T	49388401	_Variant_195	0	HSP90AA1	intron_variant
5	T	C	49388877	rs312732990	0	HSP90AA1	intron_variant
5	T	A	49388881	rs314731044	0	HSP90AA1	intron_variant
5	G	A	49389032	rs316136543	0	HSP90AA1	synonymous_variant
5	A	G	49389238	rs734170379	0	HSP90AA1	missense_variant
5	G	A	49389293	rs740824466	0	HSP90AA1	synonymous_variant
5	G	C	49389478	rs732823349	0	HSP90AA1	missense_variant
5	G	A	49389480	rs734932557	0	HSP90AA1	missense_variant
5	C	T	49389490	rs737002979	0	HSP90AA1	missense_variant
5	C	A,T	49389504	_Variant_228	0	HSP90AA1	splice_region_variant,intron_variant
5	A	G	49389505	_Variant_229	0	HSP90AA1	splice_region_variant,intron_variant
5	A	G	49389520	rs733306652	0	HSP90AA1	intron_variant
5	C	A	49389626	rs731345705	0	HSP90AA1	intron_variant
5	G	A	49389665	rs741272089	0	HSP90AA1	synonymous_variant
5	T	C	49391073	rs731050038	0	HSP90AA1	intron_variant
5	C	T	49391091	rs315919579	0	HSP90AA1	intron_variant
5	A	C	49391147	rs739096107	0	HSP90AA1	intron_variant

5	A	T	49391154	rs736276718	0	HSP90AA1	intron_variant
5	C	A	49391279	rs314063915	0	HSP90AA1	intron_variant
5	A	G	49391335	rs732599834	0	HSP90AA1	intron_variant
5	G	A	49391342	rs736022284	0	HSP90AA1	intron_variant
5	A	G	49391344	rs736745908	0	HSP90AA1	intron_variant
5	G	C	49391381	rs737527845	0	HSP90AA1	intron_variant
5	A	C	49391382	rs740815397	0	HSP90AA1	intron_variant
5	T	C	49391729	rs313392169	0	HSP90AA1	intron_variant
5	G	A	49391828	rs316167392	0	HSP90AA1	intron_variant
5	A	G	49392222	rs3137996	0	HSP90AA1	synonymous_variant
5	C	T	49392559	rs316827498	0	HSP90AA1	synonymous_variant
5	A	G	49392634	rs16508339	0	HSP90AA1	intron_variant
7	G	T	13182561	rs316257757	0	ENSGALG00000008738	intron_variant
7	C	T	13182586	rs316510881	0	ENSGALG00000008738	intron_variant
7	C	T	13183617	_Variant_351	0	ENSGALG00000008738	intron_variant
7	C	T	13183625	_Variant_352	0	ENSGALG00000008738	intron_variant
7	G	T	13183923	rs14609449	0	ENSGALG00000008738	intron_variant
7	C	A	13184207	_Variant_361	0	ENSGALG00000008738	intron_variant
7	A	G	13184208	_Variant_362	0	ENSGALG00000008738	intron_variant
7	C	T	13184329	rs737333650	0	ENSGALG00000008738	intron_variant
7	C	T	13184393	_Variant_366	0	ENSGALG00000008738	intron_variant
7	A	G	13184394	_Variant_367	0	ENSGALG00000008738	intron_variant
7	A	G	13184399	rs740794674	0	ENSGALG00000008738	intron_variant
7	A	G	13184667	rs312606212	0	ENSGALG00000008738	intron_variant
7	C	T	13184682	rs16586531	0	ENSGALG00000008738	intron_variant
7	A	C	13185782	_Variant_399	0	ENSGALG00000008738	intron_variant
7	G	A	13185791	_Variant_400	0	ENSGALG00000008738	intron_variant
7	G	T	13187179	_Variant_432	0	ENSGALG00000008738	intron_variant
7	C	G	13187545	rs10731610	0	ENSGALG00000008738	3_prime_UTR_variant
7	G	C	13187652	rs741114909	0	ENSGALG00000008738	3_prime_UTR_variant
7	C	T	13188127	rs16586541	0	ENSGALG00000008738	downstream_gene_variant
7	G	A	13188358	rs315814237	0	ENSGALG00000008738	downstream_gene_variant
7	G	A	13188489	rs731984755	0	ENSGALG00000008738	downstream_gene_variant
7	G	C	13188679	rs738606663	0	ENSGALG00000008738	downstream_gene_variant
7	A	T	13189629	rs314076594	0	ENSGALG00000008738	downstream_gene_variant
7	C	A	13189803	rs314347374	0	ENSGALG00000008738	downstream_gene_variant
7	C	T	13189852	rs740487247	0	ENSGALG00000008738	downstream_gene_variant
7	C	A	13189930	rs315219034	0	ENSGALG00000008738	downstream_gene_variant
7	A	G	13190030	rs315477703	0	ENSGALG00000008738	downstream_gene_variant
7	A	G	13190158	rs315971887	0	ENSGALG00000008738	downstream_gene_variant
7	C	G	13190183	rs316509496	0	ENSGALG00000008738	downstream_gene_variant

7	G	C	13190487	rs735382587	0	ENSGALG00000008738	downstream_gene_variant
7	A	T	13190670	rs740713515	0	ENSGALG00000008738	downstream_gene_variant
7	C	T	13191075	rs313196841	0	ENSGALG00000008738	downstream_gene_variant
7	C	T	13191492	rs315331461	0	ENSGALG00000008738	downstream_gene_variant
7	G	A	13192056	rs316190473	0	ENSGALG00000008738	downstream_gene_variant
7	T	C	13192791	rs738390084	0	ENSGALG00000008738	downstream_gene_variant
7	C	T	13193002	rs312739484	0	ENSGALG00000008738	downstream_gene_variant
13	G	C	12201219	_Variant_5		G3BP1	downstream_gene_variant
13	T	C	12201606	_Variant_8		G3BP1	downstream_gene_variant
13	T	C	12204112	rs13547775		G3BP1	intron_variant
13	A	G	12204325	rs14061490		G3BP1	intron_variant
13	T	C	12204475	rs14061492		G3BP1	intron_variant
13	T	A	12204602	rs315823314		G3BP1	intron_variant
13	A	G	12204668	rs312618514		G3BP1	intron_variant
13	T	C	12229907	_Variant_100		G3BP1	upstream_gene_variant
13	G	C	12230941	rs15702661		G3BP1	upstream_gene_variant
3	T	C	105082885	rs13525105		CENPO	downstream_gene_variant
3	T	C	105086099	rs14409177		CENPO	3_prime_UTR_variant
3	A	T	105086728	rs13525110		CENPO	3_prime_UTR_variant
3	T	C	105086732	rs13525111		CENPO	3_prime_UTR_variant
3	C	T	105086734	rs13525112		CENPO	3_prime_UTR_variant
3	G	A	105087209	rs312280125		CENPO	3_prime_UTR_variant
5	G	C	49384720	rs315059855		HSP90AA1	downstream_gene_variant
5	G	A	49387447	_Variant_181		HSP90AA1	downstream_gene_variant
5	C	T	49390428	rs794334272		HSP90AA1	missense_variant
5	T	C	49390478	_Variant_253		HSP90AA1	intron_variant
5	C	T	49390870	rs731050770		HSP90AA1	synonymous_variant
5	A	G	49390903	rs734513900		HSP90AA1	synonymous_variant
5	A	G	49390953	rs794432502		HSP90AA1	intron_variant
5	C	T	49391152	rs317046685		HSP90AA1	intron_variant
5	C	T	49391177	rs313363595		HSP90AA1	intron_variant
5	T	C	49391283	rs316906268		HSP90AA1	intron_variant
5	T	A	49391329	rs312591733		HSP90AA1	intron_variant
5	A	G	49391832	_Variant_278		HSP90AA1	intron_variant
5	T	C	49391833	_Variant_279		HSP90AA1	intron_variant
5	G	A	49392353	_Variant_283		HSP90AA1	intron_variant
5	G	A	49392355	rs16508337		HSP90AA1	intron_variant
5	A	G	49392754	rs313076295		HSP90AA1	intron_variant
5	T	C	49392812	rs315889247		HSP90AA1	intron_variant
5	G	A	49393076	_Variant_297		HSP90AA1	intron_variant
7	T	C	13181171	rs316387998		ENSGALG00000008738	upstream_gene_variant

7	T	C	13184690	rs313477487		ENSGALG00000008738	intron_variant
7	C	T	13184710	rs313509690		ENSGALG00000008738	intron_variant
7	T	C	13184723	rs314082069		ENSGALG00000008738	intron_variant
7	T	C	13184816	_Variant_387		ENSGALG00000008738	intron_variant
7	A	T	13185735	_Variant_395		ENSGALG00000008738	intron_variant
7	T	C	13186079	rs318124171		ENSGALG00000008738	intron_variant
7	C	G	13186178	rs16586538		ENSGALG00000008738	intron_variant
7	A	G	13186593	rs315894426		ENSGALG00000008738	intron_variant
7	C	G	13187267	rs312756266		ENSGALG00000008738	intron_variant
7	C	T	13188853	rs318065071		ENSGALG00000008738	downstream_gene_variant
7	C	G	13190834	rs312816664		ENSGALG00000008738	downstream_gene_variant
7	A	G	13193103	rs15847045		ENSGALG00000008738	downstream_gene_variant

Supplementary Table 16: eQTls identified in Ceca of low and high FCR broilers

chr	Ref	Alt	pos	snp_id	NegLog10 p-Value	Gene	Location
21	A	G	2002983	rs15181433	2.097497634	ENSGALG00000001359	downstream_gene_variant
3	G	A	24822333	rs794597744	2.040554066	PPM1B	downstream_gene_variant
3	C	T	24811667	rs732689598	1.922023958	PPM1B	intron_variant
3	A	G	24811608	rs16240336	1.811627256	PPM1B	intron_variant
21	A	C	1967450	rs317724807	1.694058784	AHCY	downstream_gene_variant
10	C	T	11850703	rs315381519	1.672628747	ABHD17C	intron_variant
3	G	A	24811662	rs740679964	1.672628747	PPM1B	intron_variant
21	T	A	1999838	rs317003286	1.539290116	ENSGALG00000001359	downstream_gene_variant
21	C	T	1987615	_Variant_513	1.512104586	ENSGALG00000001359	intron_variant
21	G	A	1992608	rs741708921	1.512104586	ENSGALG00000001359	intron_variant
21	A	T	1995584	_Variant_534	1.512104586	ENSGALG00000001359	3_prime_UTR_variant
21	A	G	1995641	rs315667733	1.512104586	ENSGALG00000001359	3_prime_UTR_variant
21	T	C	1998302	rs735224699	1.512104586	ENSGALG00000001359	downstream_gene_variant
21	A	G	1999598	_Variant_557	1.512104586	ENSGALG00000001359	downstream_gene_variant
21	A	C	1999618	_Variant_558	1.512104586	ENSGALG00000001359	downstream_gene_variant
21	A	C	1999620	rs15181423	1.512104586	ENSGALG00000001359	downstream_gene_variant
21	C	T	2001925	rs738048421	1.512104586	ENSGALG00000001359	downstream_gene_variant
21	C	G	2002007	rs740325027	1.512104586	ENSGALG00000001359	downstream_gene_variant
21	G	C	2002014	rs732784124	1.512104586	ENSGALG00000001359	downstream_gene_variant
21	T	C	2003569	rs732936078	1.512104586	ENSGALG00000001359	downstream_gene_variant
21	G	A	2004446	_Variant_593	1.512104586	ENSGALG00000001359	downstream_gene_variant
21	G	T	2004480	rs732546063	1.512104586	ENSGALG00000001359	downstream_gene_variant
3	G	A	24809972	rs313943112	1.512104586	PPM1B	intron_variant
3	T	C	24816341	rs741067461	1.512104586	PPM1B	intron_variant
7	C	T	13826038	rs733814084	1.512104586	ITGA4	downstream_gene_variant
7	A	T	13826068	rs732496374	1.512104586	ITGA4	downstream_gene_variant
21	A	T	2002128	rs314038704	1.497305722	ENSGALG00000001359	downstream_gene_variant
3	G	T	24811659	rs737292718	1.472730199	PPM1B	intron_variant
5	C	G	14095079	_Variant_893	1.458430538	ENSGALG00000006740	missense_variant
8	G	A	20095084	rs315798025	1.455746105	GPBP1L1	intron_variant
3	T	C	24819471	rs15301049	1.393360937	PPM1B	3_prime_UTR_variant
10	A	G	11855189	rs317349624	1.361050473	ABHD17C	intron_variant
21	C	T	2002663	rs738060517	1.361050473	ENSGALG00000001359	downstream_gene_variant
3	G	A	24811640	rs317341558	1.361050473	PPM1B	intron_variant
4	T	A	9330391	rs15489961	1.361050473	SH3BGRL	upstream_gene_variant
10	C	T	11850414	rs314604288	1.355360527	ABHD17C	intron_variant
21	T	G	1997644	_Variant_544	1.355360527	ENSGALG00000001359	downstream_gene_variant
7	G	A	13826306	rs738105407	1.355360527	ITGA4	downstream_gene_variant
3	T	C	24812259	_Variant_638	1.345272011	PPM1B	intron_variant

4	A	G	9326695	rs315207889	1.308172415	SH3BGRL	upstream_gene_variant
4	A	C	9326703	rs312997792	1.308172415	SH3BGRL	upstream_gene_variant
8	G	A	27688807	_Variant_1180	1.265045211	RPE65	upstream_gene_variant
21	T	A	2001541	_Variant_563	1.226605337	ENSGALG00000001359	downstream_gene_variant
21	T	C	2001553	_Variant_564	1.226605337	ENSGALG00000001359	downstream_gene_variant
21	T	C	2001559	_Variant_565	1.226605337	ENSGALG00000001359	downstream_gene_variant
21	G	A	2001569	_Variant_566	1.226605337	ENSGALG00000001359	downstream_gene_variant
21	T	C	2001573	rs737929913	1.226605337	ENSGALG00000001359	downstream_gene_variant
4	T	A	9329503	rs737813694	1.219760666	SH3BGRL	upstream_gene_variant
21	A	G	1992480	rs736950409	1.193294397	ENSGALG00000001359	intron_variant
21	A	G	2005347	rs318145183	1.193294397	ENSGALG00000001359	downstream_gene_variant
5	A	T	14141746	rs14518507	1.193294397	ENSGALG00000006740	intron_variant
5	C	T	14142190	_Variant_911	1.193294397	ENSGALG00000006740	intron_variant
8	G	A	27683967	_Variant_1153	1.184180377	RPE65	upstream_gene_variant
8	T	C	27683974	rs732992288	1.184180377	RPE65	upstream_gene_variant
3	C	T	24814445	rs733143271	1.175236095	PPM1B	intron_variant
3	A	G	24814446	rs737479787	1.175236095	PPM1B	intron_variant
3	G	A	24814447	rs740951674	1.175236095	PPM1B	intron_variant
3	G	A	24818992	rs314513795	1.175236095	PPM1B	3_prime_UTR_variant
4	T	C	9322558	rs731671992	1.175236095	SH3BGRL	upstream_gene_variant
4	C	A	9322616	rs732088703	1.175236095	SH3BGRL	upstream_gene_variant
4	A	G	9322660	rs738373439	1.175236095	SH3BGRL	upstream_gene_variant
4	G	A	9323804	rs316805252	1.175236095	SH3BGRL	upstream_gene_variant
4	T	A	9323834	rs312521137	1.175236095	SH3BGRL	upstream_gene_variant
4	G	A	9323886	rs739012791	1.175236095	SH3BGRL	upstream_gene_variant
4	G	A	9324065	rs314107615	1.175236095	SH3BGRL	upstream_gene_variant
7	G	T	13817280	rs15848662	1.175236095	ITGA4	intron_variant
7	C	T	13820931	rs737369171	1.175236095	ITGA4	3_prime_UTR_variant
8	C	T	20106720	rs736700103	1.175236095	GPBP1L1	5_prime_UTR_variant
8	A	G	27688468	rs15941091	1.175236095	RPE65	upstream_gene_variant
1	T	G	117663527	rs313114727	1.163534327	EIF2S3L	downstream_gene_variant
21	T	G	1984863	rs312456283	1.163534327	ENSGALG00000001359	intron_variant
21	T	A	1984876	rs313268795	1.163534327	ENSGALG00000001359	intron_variant
21	G	A	1985897	rs314418526	1.163534327	ENSGALG00000001359	intron_variant
4	A	G	9326656	rs316079226	1.152464471	SH3BGRL	upstream_gene_variant
4	G	A	9326672	rs15489958	1.152464471	SH3BGRL	upstream_gene_variant
21	G	C	2002300	rs312890218	1.110150454	ENSGALG00000001359	downstream_gene_variant
1	C	T	117679024	rs731239811	1.094141974	EIF2S3L	upstream_gene_variant
21	C	T	2005521	rs731666903	1.094141974	ENSGALG00000001359	downstream_gene_variant
3	C	T	24819480	rs15301050	1.09246113	PPM1B	3_prime_UTR_variant
5	A	G	14082491	_Variant_888	1.09246113	ENSGALG00000006740	missense_variant

3	C	A	24820455	_Variant_723	1.079540035	PPM1B	downstream_gene_variant
10	G	A	11853455	_Variant_223	1.05450697	ABHD17C	intron_variant
4	C	A	9326794	rs317398948	1.05450697	SH3BGRL	upstream_gene_variant
4	A	T	9328508	rs317291992	1.05450697	SH3BGRL	upstream_gene_variant
7	T	A	13860248	rs315744464	1.05450697	ITGA4	downstream_gene_variant
3	A	G	24810288	rs318121086	1.051248307	PPM1B	intron_variant
3	A	G	24810318	rs314138627	1.051248307	PPM1B	intron_variant
8	A	G	20080473	rs735121688	1.051248307	ITGA4	downstream_gene_variant
8	T	G	20080507	_Variant_1011	1.051248307	ITGA4	downstream_gene_variant
8	C	A	20082934	rs13682057	1.051248307	ITGA4	downstream_gene_variant
8	G	A	20095017	_Variant_1119	1.051248307	GPBP1L1	intron_variant
7	A	G	13816809	rs316724654	1.041106828	ITGA4	synonymous_variant
3	G	A	24822101	rs314128635	1.025652722	PPM1B	downstream_gene_variant
21	A	G	2002236	_Variant_579	1.023932632	ENSGALG00000001359	downstream_gene_variant
7	A	G	13817525	rs732761073	1.023932632	ITGA4	intron_variant
7	T	A	13817530	rs734785471	1.023932632	ITGA4	intron_variant
3	A	G	24812282	rs315480797	1.00803222	PPM1B	intron_variant
3	G	A	24811724	rs316869807	1.006374543	PPM1B	intron_variant
20	T	G	1677664	rs736650063	0.982117511	AHCY	intron_variant
20	T	A	1863537	rs316618233	0.982117511	AHCY	downstream_gene_variant
21	A	T	1996976	rs313391860	0.982117511	ENSGALG00000001359	downstream_gene_variant
21	C	T	2005027	_Variant_597	0.982117511	ENSGALG00000001359	downstream_gene_variant
21	C	T	2005030	_Variant_598	0.982117511	ENSGALG00000001359	downstream_gene_variant
5	G	C	14146156	_Variant_924	0.982117511	ENSGALG00000006740	intron_variant
7	A	G	13821297	_Variant_975	0.982117511	ITGA4	3_prime_UTR_variant
7	A	G	13821301	rs14609967	0.982117511	ITGA4	3_prime_UTR_variant
7	G	A	13822881	rs14609971	0.982117511	ITGA4	downstream_gene_variant
8	C	T	20080489	rs739085500	0.982117511	ITGA4	downstream_gene_variant
21	T	G	2004794	rs738112672	0.951334318	ENSGALG00000001359	downstream_gene_variant
4	T	G	9326169	rs740052286	0.951334318	SH3BGRL	upstream_gene_variant
4	A	G	9326184	_Variant_828	0.951334318	SH3BGRL	upstream_gene_variant
5	G	A	14141118	_Variant_903	0.951334318	ENSGALG00000006740	intron_variant
8	G	A	27685527	_Variant_1167	0.951334318	RPE65	upstream_gene_variant
1	T	C	117667081	rs13928095	0.933251757	EIF2S3L	intron_variant
10	C	T	11850625	rs312938651	0.929587896	ABHD17C	intron_variant
3	T	A	24819514	rs317458391	0.929587896	PPM1B	3_prime_UTR_variant
7	A	T	13821936	rs315437153	0.929587896	ITGA4	downstream_gene_variant
20	T	C	1671040	_Variant_275	0.916013009	AHCY	intron_variant
20	T	A	1671044	_Variant_276	0.916013009	AHCY	intron_variant
20	C	T	1677749	rs732799709	0.900680427	AHCY	intron_variant
8	G	A	27665081	_Variant_1131	0.900680427	GPBP1L1	upstream_gene_variant

3	C	T	24816522	rs313416711	0.897461823	PPM1B	intron_variant
8	T	C	20084585	rs314309020	0.896680435	ITGA4	downstream_gene_variant
8	A	T	27688767	_Variant_1179	0.896680435	RPE65	upstream_gene_variant
8	A	G	20081270	_Variant_1024	0.884156536	ITGA4	downstream_gene_variant
21	G	T	1997973	rs313882574	0.857636384	ENSGALG00000001359	downstream_gene_variant
3	T	C	24822139	rs15301084	0.857636384	PPM1B	downstream_gene_variant
8	G	A	27690426	rs313227380	0.857636384	RPE65	upstream_gene_variant
20	C	T	1660330	rs741519497	0.85496093	AHCY	intron_variant
21	C	T	1989000	rs315047985	0.85496093	ENSGALG00000001359	intron_variant
3	C	T	24820432	_Variant_720	0.85496093	PPM1B	downstream_gene_variant
3	C	T	24820445	_Variant_722	0.85496093	PPM1B	downstream_gene_variant
4	T	C	9326206	rs315713454	0.85496093	SH3BGRL	upstream_gene_variant
5	C	T	14095441	_Variant_899	0.85496093	ENSGALG00000006740	intron_variant
5	G	A	14095447	rs317843387	0.85496093	ENSGALG00000006740	intron_variant
5	G	C	14145210	_Variant_921	0.85496093	ENSGALG00000006740	intron_variant
3	G	A	24828431	rs313301954	0.849530251	PPM1B	downstream_gene_variant
20	G	C	1759556	rs736712634	0.847059963	AHCY	intron_variant
20	T	A	1759774	_Variant_317	0.847059963	AHCY	intron_variant
20	C	G	1760742	_Variant_342	0.847059963	AHCY	intron_variant
20	C	T	1769422	_Variant_412	0.847059963	AHCY	intron_variant
20	T	C	1769428	rs313902620	0.847059963	AHCY	intron_variant
3	G	A	24812531	rs317160190	0.847059963	PPM1B	intron_variant
3	G	T	24812537	rs14329053	0.847059963	PPM1B	intron_variant
3	G	T	24814172	rs313375800	0.847059963	PPM1B	intron_variant
3	G	A	24814357	rs315382634	0.847059963	PPM1B	intron_variant
3	G	A	24815507	rs29007267	0.847059963	PPM1B	intron_variant
3	C	T	24815909	_Variant_674	0.847059963	PPM1B	intron_variant
3	G	T	24816716	rs315159583	0.847059963	PPM1B	intron_variant
3	A	G	24816871	_Variant_687	0.847059963	PPM1B	intron_variant
3	G	A	24816886	_Variant_688	0.847059963	PPM1B	intron_variant
3	C	T	24816963	rs736433009	0.847059963	PPM1B	intron_variant
3	T	C	24819012	rs316654642	0.847059963	PPM1B	3_prime_UTR_variant
3	G	C	24819280	rs313939993	0.847059963	PPM1B	3_prime_UTR_variant
4	T	C	9327228	_Variant_850	0.847059963	SH3BGRL	upstream_gene_variant
7	G	A	13820216	rs733026901	0.847059963	ITGA4	3_prime_UTR_variant
7	C	T	13820329	_Variant_968	0.847059963	ITGA4	3_prime_UTR_variant
8	T	C	20080682	rs735131857	0.847059963	ITGA4	downstream_gene_variant
8	A	G	20081236	_Variant_1023	0.847059963	ITGA4	downstream_gene_variant
8	A	G	20081314	rs737540304	0.847059963	ITGA4	downstream_gene_variant
8	A	G	27672874	rs316760562	0.847059963	RPE65	downstream_gene_variant
8	A	G	27674948	_Variant_1147	0.847059963	RPE65	downstream_gene_variant

8	A	G	27684325	rs312564949	0.847059963	RPE65	upstream_gene_variant
21	T	C	1985784	rs740686985	0.843951128	ENSGALG00000001359	intron_variant
21	G	A	1985823	_Variant_488	0.843951128	ENSGALG00000001359	intron_variant
1	T	A	117674829	rs13686034	0.837880853	EIF2S3L	intron_variant
4	T	C	9322506	rs732426444	0.826309941	SH3BGRL	upstream_gene_variant
4	C	G	9322852	rs313674244	0.826309941	SH3BGRL	upstream_gene_variant
8	C	T	27685369	rs315163898	0.779010066	RPE65	upstream_gene_variant
10	C	T	11848907	rs14007769	0.762067084	ABHD17C	intron_variant
10	A	G	11850276	rs14007775	0.762067084	ABHD17C	intron_variant
10	G	A	11852933	rs14948622	0.762067084	ABHD17C	intron_variant
10	G	A	11853115	rs315363211	0.762067084	ABHD17C	intron_variant
5	G	A	14145188	_Variant_920	0.762067084	ENSGALG00000006740	intron_variant
5	C	T	14150720	_Variant_932	0.755143376	ENSGALG00000006740	missense_variant
10	G	A	11849346	rs740948243	0.755143376	ABHD17C	intron_variant
10	A	C	11851795	rs313558361	0.755143376	ABHD17C	intron_variant
10	T	A	11853552	rs13545318	0.755143376	ABHD17C	intron_variant
20	G	A	1657429	rs315336976	0.755143376	AHCY	intron_variant
20	T	G	1669436	rs313236105	0.755143376	AHCY	intron_variant
20	C	T	1770192	_Variant_415	0.755143376	AHCY	intron_variant
20	G	A	1770194	_Variant_416	0.755143376	AHCY	intron_variant
20	C	T	1863892	_Variant_467	0.755143376	AHCY	downstream_gene_variant
21	A	G	1985780	rs315196246	0.755143376	ENSGALG00000001359	intron_variant
21	G	A	1999501	rs15181420	0.755143376	ENSGALG00000001359	downstream_gene_variant
3	T	C	24820315	rs313300755	0.755143376	PPM1B	3_prime_UTR_variant
3	A	G	24820466	_Variant_724	0.755143376	PPM1B	downstream_gene_variant
4	A	G	9327420	rs316629352	0.755143376	SH3BGRL	upstream_gene_variant
5	G	T	14095392	_Variant_898	0.755143376	ENSGALG00000006740	intron_variant
5	T	G	14141832	rs14518508	0.755143376	ENSGALG00000006740	intron_variant
7	A	C	13858887	rs731141150	0.755143376	ITGA4	downstream_gene_variant
8	T	C	27667827	rs317846499	0.755143376	GPBP1L1	upstream_gene_variant
1	T	A	117679947	rs13928140	0.749837397	EIF2S3L	upstream_gene_variant
20	C	G	1762828	_Variant_377	0.749837397	AHCY	intron_variant
3	A	G	24829023	rs14329063	0.749837397	PPM1B	downstream_gene_variant
1	A	C	117668416	rs15399190	0.735503077	EIF2S3L	intron_variant
3	T	C	24811643	rs317691317	0.735503077	PPM1B	intron_variant
5	G	A	14081912	rs314068600	0.735503077	ENSGALG00000006740	missense_variant
20	G	T	1770479	_Variant_418	0.722330588	AHCY	intron_variant
20	G	A	1861693	rs734013341	0.720264031	AHCY	3_prime_UTR_variant
3	T	C	24764120	rs16240277	0.720264031	PPM1B	intron_variant
3	T	C	24812900	_Variant_646	0.720264031	PPM1B	intron_variant
4	G	A	9333014	rs316864978	0.720264031	SH3BGRL	upstream_gene_variant

8	T	C	20084938	_Variant_1059	0.720264031	GPBP1L1	downstream_gene_variant
8	C	T	20084766	rs312910498	0.71789328	GPBP1L1	downstream_gene_variant
5	A	G	14141986	rs318132874	0.717186237	ENSGALG00000006740	intron_variant
20	C	T	1661762	rs315042908	0.713382367	AHCY	intron_variant
20	T	G	1860470	rs15169709	0.713382367	AHCY	intron_variant
5	G	T	14081924	_Variant_882	0.713382367	ENSGALG00000006740	missense_variant
8	T	C	20080465	rs739928973	0.713382367	ITGA4	downstream_gene_variant
8	A	G	20082435	rs16638834	0.686336881	ITGA4	downstream_gene_variant
20	C	T	1761551	_Variant_371	0.681187826	AHCY	intron_variant
8	T	C	27665079	_Variant_1130	0.681187826	GPBP1L1	upstream_gene_variant
1	G	T	117668939	_Variant_61	0.675685328	EIF2S3L	intron_variant
4	A	G	9327756	rs315241687	0.675685328	SH3BGRL	upstream_gene_variant
20	G	A	1848380	rs315758053	0.675006791	AHCY	intron_variant
21	G	A	1982010	rs16178422	0.675006791	ENSGALG00000001359	intron_variant
1	A	G	117675039	_Variant_77	0.673882259	EIF2S3L	intron_variant
20	G	A	1657332	rs740112052	0.673882259	AHCY	intron_variant
20	T	C	1669491	rs740197513	0.673882259	AHCY	intron_variant
7	T	C	13859409	rs14609988	0.673882259	ITGA4	downstream_gene_variant
8	G	A	20085478	rs739856208	0.673882259	GPBP1L1	downstream_gene_variant
3	A	G	24810517	rs317737026	0.65942818	PPM1B	intron_variant
3	C	T	24810523	rs317275048	0.65942818	PPM1B	intron_variant
3	T	A	24810528	rs314510141	0.65942818	PPM1B	intron_variant
7	C	T	13843759	rs318037229	0.648343264	ITGA4	downstream_gene_variant
21	C	T	1994393	_Variant_531	0.648270723	ENSGALG00000001359	3_prime_UTR_variant
21	C	T	1994429	rs316685305	0.648270723	ENSGALG00000001359	3_prime_UTR_variant
21	G	T	1996602	rs314798107	0.648270723	ENSGALG00000001359	downstream_gene_variant
21	T	C	1996646	rs315659342	0.648270723	ENSGALG00000001359	downstream_gene_variant
21	A	T	1998035	rs315936207	0.648270723	ENSGALG00000001359	downstream_gene_variant
4	G	T	9317626	rs731561447	0.648270723	SH3BGRL	downstream_gene_variant
4	T	A	9322963	rs312841505	0.648270723	SH3BGRL	upstream_gene_variant
4	A	G	9323040	rs314313485	0.648270723	SH3BGRL	upstream_gene_variant
4	T	C	9323116	rs317614213	0.648270723	SH3BGRL	upstream_gene_variant
4	C	T	9323200	rs316384930	0.648270723	SH3BGRL	upstream_gene_variant
4	A	G	9323444	_Variant_805	0.648270723	SH3BGRL	upstream_gene_variant
4	A	T	9324259	rs739827397	0.648270723	SH3BGRL	upstream_gene_variant
4	G	C	9325083	rs314316011	0.648270723	SH3BGRL	upstream_gene_variant
4	T	C	9326357	rs313025568	0.648270723	SH3BGRL	upstream_gene_variant
5	A	T	14139234	rs316416979	0.648270723	ENSGALG00000006740	intron_variant
5	G	A	14143163	rs14518510	0.648270723	ENSGALG00000006740	intron_variant
20	T	G	1770207	rs315237483	0.648127899	AHCY	intron_variant
4	A	G	9328710	_Variant_857	0.648127899	SH3BGRL	upstream_gene_variant

3	T	C	24810747	rs733854113	0.644372624	PPM1B	intron_variant
1	C	T	117657746	rs314241844	0.621396344		intergenic
8	C	T	20084374	rs316574038	0.621396344	ITGA4	downstream_gene_variant
5	C	G	14082513	_Variant_889	0.620586691	ENSGALG00000006740	missense_variant
5	G	A	14082620	rs741044592	0.620586691	ENSGALG00000006740	missense_variant
7	T	A	13860351	rs736702910	0.61912695	ITGA4	downstream_gene_variant
1	A	G	117668217	rs312450319	0.616963137	EIF2S3L	intron_variant
1	C	A	117668220	rs312507583	0.616963137	EIF2S3L	intron_variant
10	C	T	11847465	rs15580734	0.609596168	ABHD17C	downstream_gene_variant
21	C	G	2003561	_Variant_588	0.609596168	ENSGALG00000001359	downstream_gene_variant
8	C	T	20083857	rs14649989	0.609596168	ITGA4	downstream_gene_variant
20	A	T	1677703	rs737059319	0.604207409	AHCY	intron_variant
20	G	C	1677713	rs734420751	0.604207409	AHCY	intron_variant
8	C	T	20080210	rs314735334	0.604207409	ITGA4	downstream_gene_variant
1	A	T	117677292	rs317183450	0.601230181	EIF2S3L	intron_variant
21	G	A	1997699	_Variant_545	0.601230181	ENSGALG00000001359	downstream_gene_variant
21	C	A	1997708	rs736151333	0.601230181	ENSGALG00000001359	downstream_gene_variant
4	G	A	9326919	rs317410651	0.601230181	SH3BGRL	upstream_gene_variant
8	T	C	20092598	rs16638852	0.601230181	GPBP1L1	intron_variant
8	C	T	20093507	rs314600746	0.601230181	GPBP1L1	intron_variant
21	C	A	1986901	rs314706339	0.59239989	ENSGALG00000001359	intron_variant
21	A	G	2002026	rs315817644	0.59239989	ENSGALG00000001359	downstream_gene_variant
21	A	G	2004252	_Variant_591	0.59239989	ENSGALG00000001359	downstream_gene_variant
21	G	A	2004340	rs317588882	0.59239989	ENSGALG00000001359	downstream_gene_variant
3	G	A	24814289	rs732575941	0.59239989	PPM1B	intron_variant
3	C	T	24814292	rs735974282	0.59239989	PPM1B	intron_variant
3	G	A	24814615	rs316646252	0.59239989	PPM1B	intron_variant
3	G	A	24814887	rs739049376	0.59239989	PPM1B	intron_variant
3	C	G	24815772	rs315247591	0.59239989	PPM1B	intron_variant
3	A	G	24818276	rs313448090	0.59239989	PPM1B	3_prime_UTR_variant
3	T	G	24818554	_Variant_691	0.59239989	PPM1B	3_prime_UTR_variant
3	T	C	24819759	rs15301052	0.59239989	PPM1B	3_prime_UTR_variant
3	G	A	24819832	rs741634567	0.59239989	PPM1B	3_prime_UTR_variant
3	C	T	24819940	_Variant_712	0.59239989	PPM1B	3_prime_UTR_variant
3	T	G	24822125	rs315807997	0.59239989	PPM1B	downstream_gene_variant
3	T	C	24822606	rs15301096	0.59239989	PPM1B	downstream_gene_variant
20	C	G	1763130	_Variant_379	0.581379566	AHCY	intron_variant
20	G	C	1763140	_Variant_380	0.581379566	AHCY	intron_variant
8	G	A	20080513	rs735914112	0.581379566	ITGA4	downstream_gene_variant
8	G	A	20094180	_Variant_1113	0.581379566	GPBP1L1	intron_variant
8	C	T	20094187	rs734383515	0.581379566	GPBP1L1	intron_variant

3	G	T	24821908	rs314229023	0.580256582	PPM1B	downstream_gene_variant
8	A	G	20082550	rs733720505	0.580256582	ITGA4	downstream_gene_variant
8	C	T	27685500	_Variant_1166	0.580256582	RPE65	upstream_gene_variant
3	A	T	24822344	rs794410533	0.579132897	PPM1B	downstream_gene_variant
10	C	T	11854219	_Variant_227	0.576737516	ABHD17C	intron_variant
21	T	A	2001886	rs316006342	0.576737516	ENSGALG00000001359	downstream_gene_variant
3	T	C	24827803	rs315944889	0.576737516	PPM1B	downstream_gene_variant
4	A	G	9326770	rs313364679	0.576737516	SH3BGRL	upstream_gene_variant
20	A	C	1761547	_Variant_370	0.567555116	AHCY	intron_variant
20	C	T	1763238	_Variant_385	0.567555116	AHCY	intron_variant
3	G	A	24764008	rs733842845	0.567555116	PPM1B	intron_variant
3	G	A	24827966	_Variant_765	0.567555116	PPM1B	downstream_gene_variant
5	G	A	14150704	_Variant_931	0.567555116	ENSGALG00000006740	synonymous_variant
5	G	A	14151538	_Variant_933	0.567555116	ENSGALG00000006740	missense_variant
5	G	A	14151584	_Variant_934	0.567555116	ENSGALG00000006740	missense_variant
5	G	A	14152628	_Variant_935	0.567555116	ENSGALG00000006740	missense_variant
1	T	C	117675051	rs13928134	0.563325921	EIF2S3L	intron_variant
1	T	G	117679444	rs312978323	0.563325921	EIF2S3L	upstream_gene_variant
20	T	C	1759437	_Variant_305	0.563325921	AHCY	intron_variant
1	G	A	117665966	rs740035075	0.560640358	EIF2S3L	intron_variant
1	T	C	117667696	rs316113260	0.557063636	EIF2S3L	intron_variant
1	A	G	117677777	_Variant_119	0.557063636	EIF2S3L	intron_variant
10	C	T	11850105	rs318132479	0.552706721	ABHD17C	intron_variant
21	A	G	2000353	_Variant_561	0.552706721	ENSGALG00000001359	downstream_gene_variant
3	C	T	24816448	rs314929387	0.552706721	PPM1B	intron_variant
3	G	A	24816519	rs317009920	0.552706721	PPM1B	intron_variant
3	T	G	24818604	rs735212530	0.552706721	PPM1B	3_prime_UTR_variant
4	T	C	9322748	rs314561951	0.552706721	SH3BGRL	upstream_gene_variant
4	G	A	9325597	rs10728465	0.552706721	SH3BGRL	upstream_gene_variant
5	A	G	14144052	rs312843543	0.552706721	ENSGALG00000006740	intron_variant
5	G	A	14145255	_Variant_922	0.552706721	ENSGALG00000006740	intron_variant
5	T	A	14153377	_Variant_936	0.552706721	ENSGALG00000006740	synonymous_variant
5	T	G	14153389	_Variant_937	0.552706721	ENSGALG00000006740	missense_variant
7	A	G	13818751	_Variant_955	0.552706721	ITGA4	3_prime_UTR_variant
7	G	A	13819252	rs740728604	0.552706721	ITGA4	3_prime_UTR_variant
7	A	G	13819711	rs317107109	0.552706721	ITGA4	3_prime_UTR_variant
7	T	C	13819757	rs317246473	0.552706721	ITGA4	3_prime_UTR_variant
7	A	T	13819806	rs739705428	0.552706721	ITGA4	3_prime_UTR_variant
7	A	G	13819999	rs317641090	0.552706721	ITGA4	3_prime_UTR_variant
7	C	A	13820296	rs312418482	0.552706721	ITGA4	3_prime_UTR_variant
7	A	G	13840660	rs315747709	0.552706721	ITGA4	downstream_gene_variant

8	C	T	20080145	rs315448945	0.552706721	ITGA4	downstream_gene_variant
8	T	C	20083933	_Variant_1050	0.552706721	ITGA4	downstream_gene_variant
8	T	C	20089889	rs10731265	0.552706721	GPBP1L1	3_prime_UTR_variant
10	C	A	11851726	rs314362156	0.512841964	ABHD17C	intron_variant
10	C	T	11853855	rs737891128	0.512841964	ABHD17C	intron_variant
20	C	T	1866335	rs318034552	0.512841964	AHCY	downstream_gene_variant
3	A	G	24764036	_Variant_605	0.512841964	PPM1B	intron_variant
7	C	G	13821160	_Variant_973	0.512841964	ITGA4	3_prime_UTR_variant
7	G	A	13821162	_Variant_974	0.512841964	ITGA4	3_prime_UTR_variant
7	G	A	13845312	rs317387940	0.512841964	ITGA4	downstream_gene_variant
8	G	A	20082902	rs731434645	0.512841964	ITGA4	downstream_gene_variant
8	T	C	27686089	rs315287805	0.512841964	RPE65	upstream_gene_variant
1	A	C	117665976	rs736281441	0.510728528	EIF2S3L	intron_variant
10	T	C	11853095	rs14948625	0.510728528	ABHD17C	intron_variant
20	C	T	1760179	rs739750634	0.510728528	AHCY	intron_variant
21	T	A	1987480	_Variant_507	0.510728528	ENSGALG00000001359	intron_variant
21	A	G	1987489	_Variant_508	0.510728528	ENSGALG00000001359	intron_variant
3	T	C	24812932	rs317875073	0.510728528	PPM1B	intron_variant
3	T	C	24813859	_Variant_654	0.510728528	PPM1B	intron_variant
3	T	C	24815051	rs740911863	0.510728528	PPM1B	intron_variant
3	A	C	24816295	rs731446565	0.510728528	PPM1B	intron_variant
3	C	T	24816298	rs734884109	0.510728528	PPM1B	intron_variant
3	G	A	24816563	rs316399279	0.510728528	PPM1B	intron_variant
3	T	C	24816674	rs739945457	0.510728528	PPM1B	intron_variant
3	G	A	24819118	rs731270877	0.510728528	PPM1B	3_prime_UTR_variant
3	G	A	24819212	rs738846590	0.510728528	PPM1B	3_prime_UTR_variant
3	A	C	24819240	_Variant_698	0.510728528	PPM1B	3_prime_UTR_variant
3	G	T	24819241	_Variant_699	0.510728528	PPM1B	3_prime_UTR_variant
4	A	G	9323207	_Variant_801	0.510728528	SH3BGRL	upstream_gene_variant
4	G	C	9323517	_Variant_806	0.510728528	SH3BGRL	upstream_gene_variant
4	G	A	9323743	_Variant_807	0.510728528	SH3BGRL	upstream_gene_variant
4	G	A	9326301	_Variant_830	0.510728528	SH3BGRL	upstream_gene_variant
4	T	C	9329323	rs14426561	0.510728528	SH3BGRL	upstream_gene_variant
5	A	G	14139712	_Variant_902	0.510728528	ENSGALG00000006740	intron_variant
5	T	A	14141374	rs735801066	0.510728528	ENSGALG00000006740	intron_variant
5	A	G	14144034	_Variant_917	0.510728528	ENSGALG00000006740	intron_variant
5	A	G	14145657	_Variant_923	0.510728528	ENSGALG00000006740	intron_variant
5	T	A	14150596	_Variant_926	0.510728528	ENSGALG00000006740	synonymous_variant
5	C	T	14153464	_Variant_938	0.510728528	ENSGALG00000006740	synonymous_variant
8	T	G	20080911	rs731459816	0.510728528	ITGA4	downstream_gene_variant
8	T	G	27686401	_Variant_1173	0.510728528	RPE65	upstream_gene_variant

8	T	A	27687182	rs313763502	0.510728528	RPE65	upstream_gene_variant
8	T	C	27675603	_Variant_1149	0.501890851	RPE65	3_prime_UTR_variant
8	T	G	27685326	rs735638612	0.501890851	RPE65	upstream_gene_variant
8	G	A	27674644	rs737379832	0.485204123	RPE65	downstream_gene_variant
8	G	T	20080220	rs738738413	0.468498323	ITGA4	downstream_gene_variant
20	T	C	1857773	_Variant_437	0.465853133	AHCY	intron_variant
20	A	G	1857802	rs315111637	0.465853133	AHCY	intron_variant
3	A	C	24821285	_Variant_731	0.465853133	PPM1B	downstream_gene_variant
8	C	T	20093631	_Variant_1109	0.465853133	GPBP1L1	intron_variant
10	G	A	11849260	rs312288884	0.462067031	ABHD17C	intron_variant
5	C	A	14143470	_Variant_914	0.462067031	ENSGALG00000006740	intron_variant
5	A	T	14143679	_Variant_915	0.462067031	ENSGALG00000006740	intron_variant
5	A	G	14143704	_Variant_916	0.462067031	ENSGALG00000006740	intron_variant
5	G	A	14145150	rs315369978	0.462067031	ENSGALG00000006740	intron_variant
20	G	C	1857757	_Variant_436	0.461201906	AHCY	intron_variant
21	C	T	1992839	rs313557837	0.461201906	ENSGALG00000001359	intron_variant
5	A	T	14160865	_Variant_943	0.461201906	ENSGALG00000006740	intron_variant
8	A	G	20082643	rs738206491	0.461201906	ITGA4	downstream_gene_variant
8	C	T	20102220	rs316385983	0.458726744	GPBP1L1	intron_variant
10	A	G	11850765	rs736849020	0.455906737	ABHD17C	intron_variant
10	A	C	11853282	rs734882090	0.455906737	ABHD17C	intron_variant
10	G	A	11853284	rs738385728	0.455906737	ABHD17C	intron_variant
8	G	A	20102649	rs16638866	0.455906737	GPBP1L1	intron_variant
8	G	A	20080369	rs732146146	0.455780924	ITGA4	downstream_gene_variant
8	A	C	20094841	rs314598651	0.455780924	GPBP1L1	intron_variant
8	A	G	20086813	rs315026422	0.448286851	GPBP1L1	downstream_gene_variant
8	A	G	20104388	rs317730870	0.448286851	GPBP1L1	synonymous_variant
1	A	G	117664627	rs738285019	0.447354508	EIF2S3L	downstream_gene_variant
1	C	T	117665080	_Variant_20	0.447354508	EIF2S3L	downstream_gene_variant
1	G	C	117665522	rs731195819	0.447354508	EIF2S3L	3_prime_UTR_variant
1	T	C	117665530	rs734567544	0.447354508	EIF2S3L	3_prime_UTR_variant
1	G	C	117666284	_Variant_37	0.447354508	EIF2S3L	intron_variant
1	A	G	117666390	rs734034037	0.447354508	EIF2S3L	intron_variant
1	G	A	117666795	rs740948925	0.447354508	EIF2S3L	intron_variant
1	T	C	117671078	rs735421047	0.447354508	EIF2S3L	synonymous_variant
1	C	T	117675827	rs14878853	0.447354508	EIF2S3L	intron_variant
1	T	C	117675846	rs316089399	0.447354508	EIF2S3L	intron_variant
1	C	T	117676155	rs737832746	0.447354508	EIF2S3L	intron_variant
1	A	C	117676756	rs734228277	0.447354508	EIF2S3L	intron_variant
10	G	A	11850731	rs314249139	0.447354508	ABHD17C	intron_variant
10	T	C	11850744	rs740845056	0.447354508	ABHD17C	intron_variant

20	T	C	1760972	rs794209407	0.447354508	AHCY	intron_variant
20	C	T	1861274	rs16159454	0.447354508	AHCY	3_prime_UTR_variant
20	C	T	1863681	rs734054804	0.447354508	AHCY	downstream_gene_variant
3	G	A	24810043	rs13720051	0.447354508	PPM1B	intron_variant
3	C	T	24811749	rs734715919	0.447354508	PPM1B	intron_variant
3	T	G	24813229	rs312679846	0.447354508	PPM1B	intron_variant
4	A	G	9326313	_Variant_833	0.447354508	SH3BGRL	upstream_gene_variant
4	C	T	9326378	_Variant_835	0.447354508	SH3BGRL	upstream_gene_variant
4	T	C	9326391	_Variant_836	0.447354508	SH3BGRL	upstream_gene_variant
5	C	G	14150615	_Variant_929	0.447354508	ENSGALG00000006740	missense_variant
5	A	C	14160873	rs14518518	0.447354508	ENSGALG00000006740	intron_variant
8	C	T	20082718	_Variant_1035	0.447354508	ITGA4	downstream_gene_variant
8	A	C	20083579	rs14649988	0.447354508	ITGA4	downstream_gene_variant
8	T	G	20085652	_Variant_1067	0.447354508	GPBP1L1	downstream_gene_variant
8	T	C	20085672	rs739478599	0.447354508	GPBP1L1	downstream_gene_variant
8	G	A	20085673	rs732003761	0.447354508	GPBP1L1	downstream_gene_variant
8	T	C	20092245	rs315405424	0.447354508	GPBP1L1	intron_variant
20	G	A	1669120	rs15169586	0.439230039	AHCY	intron_variant
4	G	A	9322482	rs316633067	0.439230039	SH3BGRL	upstream_gene_variant
1	T	C	117668941	rs738818043	0.430516805	EIF2S3L	intron_variant
3	T	C	24828712	rs14329059	0.412877211	PPM1B	downstream_gene_variant
8	C	G	20082594	rs16638835	0.412877211	ITGA4	downstream_gene_variant
5	T	A	14082428	_Variant_887	0.412428652	ENSGALG00000006740	missense_variant
1	T	C	117679562	rs739603400	0.411218509	EIF2S3L	upstream_gene_variant
5	A	G	14095163	rs736982128	0.411218509	ENSGALG00000006740	missense_variant
5	T	G	14095164	rs741667276	0.411218509	ENSGALG00000006740	missense_variant
7	C	G	13826250	_Variant_983	0.411218509	ITGA4	downstream_gene_variant
7	C	T	13826252	_Variant_984	0.411218509	ITGA4	downstream_gene_variant
3	T	A	24810533	rs316330183	0.406930187	PPM1B	intron_variant
3	C	T	24820425	_Variant_719	0.395482502	PPM1B	downstream_gene_variant
7	C	T	13843337	_Variant_990	0.395482502	ITGA4	downstream_gene_variant
8	A	G	27683894	_Variant_1152	0.395482502	RPE65	upstream_gene_variant
1	G	A	117668130	rs314143250	0.394928119	EIF2S3L	intron_variant
1	G	A	117675813	rs314544558	0.394928119	EIF2S3L	intron_variant
10	G	A	11876882	rs739814944	0.394928119	ABHD17C	synonymous_variant
20	T	C	1672599	rs317749561	0.394928119	AHCY	intron_variant
20	G	C	1760333	rs13630804	0.394928119	AHCY	intron_variant
20	G	T	1863663	_Variant_464	0.394928119	AHCY	downstream_gene_variant
21	T	C	1987047	rs314428026	0.394928119	ENSGALG00000001359	intron_variant
21	T	G	1987403	rs315415923	0.394928119	ENSGALG00000001359	intron_variant
3	A	G	24811752	rs314365935	0.394928119	PPM1B	intron_variant

3	T	G	24811758	rs314980223	0.394928119	PPM1B	intron_variant
3	C	T	24819959	rs16240345	0.394928119	PPM1B	3_prime_UTR_variant
3	T	C	24820192	rs314235426	0.394928119	PPM1B	3_prime_UTR_variant
4	A	C	9317478	rs317693930	0.394928119	SH3BGRL	downstream_gene_variant
4	G	A	9318432	rs317333146	0.394928119	SH3BGRL	synonymous_variant
4	A	C	9323209	rs312795658	0.394928119	SH3BGRL	upstream_gene_variant
4	T	C	9324264	rs314990704	0.394928119	SH3BGRL	upstream_gene_variant
4	A	G	9325583	_Variant_825	0.394928119	SH3BGRL	upstream_gene_variant
8	G	A	20091191	_Variant_1076	0.394928119	GPBP1L1	intron_variant
8	G	A	20107280	rs316652107	0.394928119	GPBP1L1	upstream_gene_variant
8	A	G	27686494	_Variant_1174	0.394928119	RPE65	upstream_gene_variant
8	C	T	27689761	_Variant_1186	0.394928119	RPE65	upstream_gene_variant
1	T	C	117674289	rs314384111	0.389738178	EIF2S3L	intron_variant
1	A	G	117674527	rs317185320	0.389738178	EIF2S3L	intron_variant
3	C	A	24820444	_Variant_721	0.389738178	PPM1B	downstream_gene_variant
8	C	T	20094657	rs315443181	0.389738178	GPBP1L1	intron_variant
8	A	G	27683767	rs313702350	0.389738178	RPE65	upstream_gene_variant
3	A	G	24810463	rs14329047	0.382772943	PPM1B	intron_variant
4	G	A	9328759	_Variant_859	0.382772943	SH3BGRL	upstream_gene_variant
10	C	T	11880734	rs740956064	0.369363471	ABHD17C	upstream_gene_variant
10	A	G	11880898	rs14007817	0.369363471	ABHD17C	upstream_gene_variant
5	G	A	14172293	rs15669676	0.369363471	ENSGALG00000006740	synonymous_variant
21	A	G	2002075	rs312957865	0.368009987	ENSGALG00000001359	downstream_gene_variant
3	C	A	24828872	rs14329062	0.368009987	PPM1B	downstream_gene_variant
1	T	A	117676377	rs734135514	0.366306687	EIF2S3L	intron_variant
1	T	G	117677684	rs318091192	0.366306687	EIF2S3L	intron_variant
20	C	G	1861932	rs315912380	0.366306687	AHCY	3_prime_UTR_variant
20	C	T	1861959	rs316390224	0.366306687	AHCY	3_prime_UTR_variant
3	T	C	24822694	_Variant_756	0.366306687	PPM1B	downstream_gene_variant
3	A	G	24822699	_Variant_757	0.366306687	PPM1B	downstream_gene_variant
3	C	T	24822746	rs312866977	0.366306687	PPM1B	downstream_gene_variant
4	G	A	9326312	rs314079213	0.366306687	SH3BGRL	upstream_gene_variant
8	T	C	20092856	rs16638854	0.366306687	GPBP1L1	intron_variant
8	T	G	20080084	rs314623359	0.365118433	ITGA4	downstream_gene_variant
4	T	A	9330801	_Variant_878	0.35743893	SH3BGRL	upstream_gene_variant
1	G	A	117666637	rs316518789	0.35163148	EIF2S3L	intron_variant
1	A	G	117676621	rs316128870	0.35163148	EIF2S3L	intron_variant
20	A	C	1760318	rs16159297	0.35163148	AHCY	intron_variant
20	A	C	1760319	rs16159298	0.35163148	AHCY	intron_variant
20	G	C	1760321	_Variant_333	0.35163148	AHCY	intron_variant
21	C	T	1987189	rs313016633	0.35163148	ENSGALG00000001359	intron_variant

3	G	A	24813927	rs317749431	0.35163148	PPM1B	intron_variant
3	A	G	24819632	rs15301051	0.35163148	PPM1B	3_prime_UTR_variant
4	G	A	9326308	rs317844239	0.35163148	SH3BGRL	upstream_gene_variant
4	A	T	9329284	rs733914764	0.35163148	SH3BGRL	upstream_gene_variant
8	G	A	20080631	rs312498727	0.35163148	ITGA4	downstream_gene_variant
8	C	A	20092865	rs16638855	0.35163148	GPBP1L1	intron_variant
8	G	A	27673414	rs733132676	0.35163148	RPE65	downstream_gene_variant
8	T	C	27689748	_Variant_1185	0.35163148	RPE65	upstream_gene_variant
10	C	T	11852927	rs313912092	0.348141634	ABHD17C	intron_variant
20	G	C	1763294	_Variant_387	0.348141634	AHCY	intron_variant
3	T	C	24821928	rs316867190	0.348141634	PPM1B	downstream_gene_variant
5	T	C	14153779	rs312983081	0.348141634	ENSGALG00000006740	synonymous_variant
1	A	G	117675999	rs312754260	0.347085034	EIF2S3L	intron_variant
10	T	C	11847735	rs738136048	0.347085034	ABHD17C	3_prime_UTR_variant
21	G	A	1989980	_Variant_518	0.347085034	ENSGALG00000001359	intron_variant
4	G	A	9329321	rs318223610	0.347085034	SH3BGRL	upstream_gene_variant
8	C	T	20083774	rs317494533	0.347085034	ITGA4	downstream_gene_variant
8	C	T	27685535	_Variant_1168	0.347085034	RPE65	upstream_gene_variant
1	A	G	117668411	_Variant_59	0.344893284	EIF2S3L	intron_variant
1	T	A	117677853	_Variant_120	0.344893284	EIF2S3L	intron_variant
1	T	A	117677866	rs737332446	0.344893284	EIF2S3L	intron_variant
20	C	T	1847840	_Variant_432	0.33966801	AHCY	intron_variant
3	C	T	24827897	_Variant_764	0.33966801	PPM1B	downstream_gene_variant
8	A	G	20087304	_Variant_1073	0.33966801	GPBP1L1	downstream_gene_variant
3	C	T	24822460	rs15301094	0.338280054	PPM1B	downstream_gene_variant
7	T	G	13826260	_Variant_985	0.338280054	ITGA4	downstream_gene_variant
1	A	T	117666075	rs313667740	0.335052526	EIF2S3L	intron_variant
5	G	C	14082111	_Variant_885	0.335052526	ENSGALG00000006740	missense_variant
1	G	A	117663583	rs316155294	0.325499423	EIF2S3L	downstream_gene_variant
10	C	A	11849704	rs312573449	0.325499423	ABHD17C	intron_variant
20	A	G	1863610	_Variant_463	0.325499423	AHCY	downstream_gene_variant
4	T	A	9329513	_Variant_873	0.325499423	SH3BGRL	upstream_gene_variant
5	G	A	14082541	_Variant_890	0.315138169	ENSGALG00000006740	synonymous_variant
20	G	C	1678350	rs313471114	0.315049155	AHCY	intron_variant
20	T	C	1761424	rs794698723	0.315049155	AHCY	intron_variant
21	C	G	1986833	rs313151401	0.315049155	ENSGALG00000001359	intron_variant
3	G	A	24822415	rs737736242	0.315049155	PPM1B	downstream_gene_variant
21	C	T	2005042	_Variant_599	0.314774918	ENSGALG00000001359	downstream_gene_variant
21	G	C	2005059	rs315268906	0.314774918	ENSGALG00000001359	downstream_gene_variant
8	T	C	20094635	rs315494856	0.314774918	GPBP1L1	intron_variant
10	C	T	11850750	rs734731962	0.310217403	ABHD17C	intron_variant

10	T	C	11853272	rs740946859	0.310217403	ABHD17C	intron_variant
20	A	T	1761194	_Variant_358	0.310217403	AHCY	intron_variant
20	C	T	1837733	rs741042286	0.310217403	AHCY	synonymus_variant
21	A	G	1989413	rs312453427	0.310217403	ENSGALG00000001359	intron_variant
20	A	T	1761416	rs739752781	0.305317452	AHCY	intron_variant
20	T	C	1763267	_Variant_386	0.305317452	AHCY	intron_variant
8	C	T	27685414	rs317348610	0.305317452	RPE65	upstream_gene_variant
8	T	C	27688212	rs15941089	0.305317452	RPE65	upstream_gene_variant
3	G	T	24822453	rs15301091	0.282957841	PPM1B	downstream_gene_variant
21	G	A	1969432	rs315195811	0.275067959	AHCY	downstream_gene_variant
3	T	C	24810659	_Variant_622	0.275067959	PPM1B	intron_variant
8	C	T	27682632	_Variant_1150	0.275067959	RPE65	upstream_gene_variant
1	A	G	117679924	_Variant_137	0.269949717	EIF2S3L	upstream_gene_variant
10	G	A	11849402	rs316145272	0.269949717	ABHD17C	intron_variant
20	C	T	1770512	_Variant_419	0.269949717	AHCY	intron_variant
20	T	G	1770530	_Variant_420	0.269949717	AHCY	intron_variant
20	T	G	1862079	_Variant_457	0.269949717	AHCY	3_prime_UTR_variant
20	A	G	1862092	rs732606229	0.269949717	AHCY	3_prime_UTR_variant
5	A	G	14142193	rs313544304	0.269949717	ENSGALG00000006740	intron_variant
5	T	G	14159221	rs14518516	0.269949717	ENSGALG00000006740	synonymous_variant
7	G	A	13821106	rs731061263	0.269949717	ITGA4	3_prime_UTR_variant
8	G	C	20082565	rs731858883	0.269949717	ITGA4	downstream_gene_variant
8	T	C	20082679	rs314639479	0.269949717	ITGA4	downstream_gene_variant
8	A	G	20084300	rs317611341	0.269949717	ITGA4	downstream_gene_variant
1	A	G	117665114	_Variant_21	0.266647911	EIF2S3L	downstream_gene_variant
1	G	C	117666127	rs315710045	0.266647911	EIF2S3L	intron_variant
1	G	A	117666251	rs734139917	0.266647911	EIF2S3L	intron_variant
1	C	T	117669996	rs314318934	0.266647911	EIF2S3L	synonymous_variant
1	G	A	117674245	rs10723575	0.266647911	EIF2S3L	synonymous_variant
1	G	A	117675776	rs733893637	0.266647911	EIF2S3L	intron_variant
1	C	G	117676058	rs312685383	0.266647911	EIF2S3L	intron_variant
1	A	G	117676635	rs739482642	0.266647911	EIF2S3L	intron_variant
1	C	T	117676763	rs13686039	0.266647911	EIF2S3L	intron_variant
10	G	A	11847784	_Variant_145	0.266647911	ABHD17C	3_prime_UTR_variant
10	T	C	11847794	_Variant_146	0.266647911	ABHD17C	3_prime_UTR_variant
10	T	C	11847855	rs731412816	0.266647911	ABHD17C	3_prime_UTR_variant
10	G	A	11850218	rs15580739	0.266647911	ABHD17C	intron_variant
10	C	A	11850228	rs316564169	0.266647911	ABHD17C	intron_variant
10	A	C	11850233	rs14007774	0.266647911	ABHD17C	intron_variant
10	A	G	11852317	rs14948617	0.266647911	ABHD17C	intron_variant
20	G	A	1651635	_Variant_243	0.266647911	AHCY	intron_variant

20	C	T	1667951	rs315733699	0.266647911	AHCY	intron_variant
20	A	G	1668282	_Variant_260	0.266647911	AHCY	intron_variant
20	C	T	1760505	_Variant_335	0.266647911	AHCY	intron_variant
20	C	T	1760654	rs316390297	0.266647911	AHCY	intron_variant
20	G	A	1763619	_Variant_392	0.266647911	AHCY	intron_variant
20	A	G	1768867	rs735273274	0.266647911	AHCY	intron_variant
20	C	T	1769372	_Variant_411	0.266647911	AHCY	intron_variant
20	C	T	1858479	_Variant_439	0.266647911	AHCY	synonymous_variant
20	T	A	1862019	_Variant_455	0.266647911	AHCY	3_prime_UTR_variant
20	T	C	1862033	_Variant_456	0.266647911	AHCY	3_prime_UTR_variant
3	A	G	24816657	rs317563875	0.266647911	PPM1B	intron_variant
4	G	A	9323998	rs313176610	0.266647911	SH3BGRL	upstream_gene_variant
7	G	A	13837831	rs313475873	0.266647911	ITGA4	downstream_gene_variant
8	G	A	20084970	rs316271322	0.266647911	GPBP1L1	downstream_gene_variant
8	G	A	20092527	rs16638851	0.266647911	GPBP1L1	intron_variant
8	A	G	20104939	rs14650034	0.266647911	GPBP1L1	synonymous_variant
1	G	A	117680277	rs740030327	0.259560473	EIF2S3L	upstream_gene_variant
1	T	C	117668126	rs318206297	0.255608647	EIF2S3L	intron_variant
3	A	C	24819448	rs317558403	0.255608647	PPM1B	3_prime_UTR_variant
3	C	T	24821557	rs314540270	0.255608647	PPM1B	downstream_gene_variant
8	C	A	27669073	rs316166388	0.255608647	GPBP1L1	upstream_gene_variant
1	T	C	117680195	rs313784248	0.253222111	EIF2S3L	upstream_gene_variant
3	T	C	24827821	rs733144274	0.253222111	PPM1B	downstream_gene_variant
1	A	T	117661002	rs314641082	0.24328231	EIF2S3L	downstream_gene_variant
7	G	A	13843339	_Variant_991	0.24328231	ITGA4	downstream_gene_variant
8	A	G	20080519	rs736638908	0.24328231	ITGA4	downstream_gene_variant
8	G	A	20087165	rs16638842	0.24328231	GPBP1L1	downstream_gene_variant
8	T	C	27667817	_Variant_1136	0.24328231	GPBP1L1	upstream_gene_variant
10	G	A	11880838	_Variant_236	0.210963789	ABHD17C	upstream_gene_variant
20	G	A	1847909	rs734574141	0.210963789	AHCY	intron_variant
21	T	C	1985734	rs313748141	0.210963789	ENSGALG00000001359	intron_variant
21	A	G	1985745	rs312380374	0.210963789	ENSGALG00000001359	intron_variant
1	T	C	117660470	rs316177818	0.208480162	EIF2S3L	downstream_gene_variant
1	C	T	117677227	rs313999184	0.208480162	EIF2S3L	intron_variant
1	A	G	117677705	_Variant_116	0.208480162	EIF2S3L	intron_variant
1	A	G	117677706	_Variant_117	0.208480162	EIF2S3L	intron_variant
4	T	A	9329518	_Variant_874	0.208480162	SH3BGRL	upstream_gene_variant
8	G	A	20082554	rs13682055	0.208480162	ITGA4	downstream_gene_variant
8	A	G	20086659	rs314599402	0.208480162	GPBP1L1	downstream_gene_variant
1	C	T	117666491	rs740155528	0.206415541	EIF2S3L	intron_variant
1	G	C	117676467	rs738114407	0.206415541	EIF2S3L	intron_variant

1	T	C	117677131	rs313087780	0.206415541	EIF2S3L	intron_variant
1	A	G	117677134	rs317353618	0.206415541	EIF2S3L	intron_variant
1	C	G	117677137	rs315634333	0.206415541	EIF2S3L	intron_variant
10	T	C	11849504	rs313312970	0.206415541	ABHD17C	intron_variant
10	A	G	11850000	rs313009633	0.206415541	ABHD17C	intron_variant
10	T	C	11851202	rs14948615	0.206415541	ABHD17C	intron_variant
10	T	C	11851203	rs14948616	0.206415541	ABHD17C	intron_variant
20	T	C	1651851	rs10728018	0.206415541	AHCY	intron_variant
20	C	T	1667796	rs740508100	0.206415541	AHCY	intron_variant
20	A	G	1668627	rs314601552	0.206415541	AHCY	intron_variant
20	T	C	1668669	rs315597578	0.206415541	AHCY	intron_variant
20	G	A	1668864	rs15169584	0.206415541	AHCY	intron_variant
20	A	G	1676600	rs315682391	0.206415541	AHCY	intron_variant
20	C	T	1677087	rs737681871	0.206415541	AHCY	intron_variant
20	G	C	1759762	rs737972394	0.206415541	AHCY	intron_variant
20	T	G	1759843	rs741520339	0.206415541	AHCY	intron_variant
20	T	G	1760045	rs10721946	0.206415541	AHCY	intron_variant
20	A	G	1760125	rs733828039	0.206415541	AHCY	intron_variant
20	T	C	1760138	_Variant_323	0.206415541	AHCY	intron_variant
20	A	G	1760145	rs794646860	0.206415541	AHCY	intron_variant
20	C	T	1760146	rs794193176	0.206415541	AHCY	intron_variant
20	T	C	1760838	_Variant_344	0.206415541	AHCY	intron_variant
20	T	G	1760917	_Variant_347	0.206415541	AHCY	intron_variant
20	A	G	1763567	_Variant_389	0.206415541	AHCY	intron_variant
20	T	A	1763577	_Variant_390	0.206415541	AHCY	intron_variant
20	C	G	1766686	rs15169607	0.206415541	AHCY	intron_variant
20	T	C	1766981	_Variant_398	0.206415541	AHCY	intron_variant
20	C	T	1768129	_Variant_403	0.206415541	AHCY	intron_variant
20	G	A	1861748	rs738770208	0.206415541	AHCY	3_prime_UTR_variant
21	C	G	1986999	rs312286319	0.206415541	ENSGALG00000001359	intron_variant
21	C	T	1987034	rs312918709	0.206415541	ENSGALG00000001359	intron_variant
21	T	C	1987672	rs316995907	0.206415541	ENSGALG00000001359	intron_variant
21	C	T	1990053	rs317073409	0.206415541	ENSGALG00000001359	synonymous_variant
21	A	G	1992158	rs317635434	0.206415541	ENSGALG00000001359	synonymous_variant
21	C	A	1993456	rs741240115	0.206415541	ENSGALG00000001359	3_prime_UTR_variant
21	A	G	1993586	_Variant_529	0.206415541	ENSGALG00000001359	3_prime_UTR_variant
21	G	A	1993671	rs316918734	0.206415541	ENSGALG00000001359	3_prime_UTR_variant
21	A	G	1994679	rs739984372	0.206415541	ENSGALG00000001359	3_prime_UTR_variant
21	T	G	1995985	rs317598584	0.206415541	ENSGALG00000001359	3_prime_UTR_variant
21	T	G	1996810	rs732890214	0.206415541	ENSGALG00000001359	downstream_gene_variant
21	G	A	1996827	rs737619783	0.206415541	ENSGALG00000001359	downstream_gene_variant

21	C	T	1996834	rs740906021	0.206415541	ENSGALG00000001359	downstream_gene_variant
21	A	G	1998175	rs315462547	0.206415541	ENSGALG00000001359	downstream_gene_variant
21	G	A	1998199	rs312536484	0.206415541	ENSGALG00000001359	downstream_gene_variant
21	G	A	1998217	_Variant_551	0.206415541	ENSGALG00000001359	downstream_gene_variant
21	G	C	1998267	_Variant_552	0.206415541	ENSGALG00000001359	downstream_gene_variant
21	G	C	1998277	_Variant_553	0.206415541	ENSGALG00000001359	downstream_gene_variant
21	C	T	1998278	_Variant_554	0.206415541	ENSGALG00000001359	downstream_gene_variant
3	T	C	24786865	rs14328992	0.206415541	PPM1B	5_prime_UTR_variant
3	C	T	24811874	_Variant_636	0.206415541	PPM1B	intron_variant
3	G	T	24812545	_Variant_642	0.206415541	PPM1B	intron_variant
3	G	C	24813471	_Variant_650	0.206415541	PPM1B	intron_variant
3	G	A	24813532	rs16240337	0.206415541	PPM1B	intron_variant
3	A	G	24814405	rs318155081	0.206415541	PPM1B	intron_variant
3	C	T	24814671	rs731496866	0.206415541	PPM1B	intron_variant
3	G	A	24815867	rs794206807	0.206415541	PPM1B	intron_variant
3	G	T	24818986	rs314519587	0.206415541	PPM1B	3_prime_UTR_variant
3	T	C	24819833	rs16240341	0.206415541	PPM1B	3_prime_UTR_variant
3	G	A	24819874	_Variant_710	0.206415541	PPM1B	3_prime_UTR_variant
3	C	G	24819879	_Variant_711	0.206415541	PPM1B	3_prime_UTR_variant
3	G	C	24820220	rs316883168	0.206415541	PPM1B	3_prime_UTR_variant
4	G	A	9325012	rs317947841	0.206415541	SH3BGRL	upstream_gene_variant
7	G	C	13817372	rs15848667	0.206415541	ITGA4	intron_variant
8	A	G	20082762	rs16638837	0.206415541	ITGA4	downstream_gene_variant
8	G	A	20083070	rs10723529	0.206415541	ITGA4	downstream_gene_variant
8	C	T	20083976	rs794438954	0.206415541	ITGA4	downstream_gene_variant
8	C	A	20085560	_Variant_1065	0.206415541	GPBP1L1	downstream_gene_variant
8	C	G	20085561	rs739016020	0.206415541	GPBP1L1	downstream_gene_variant
8	G	C	20091231	_Variant_1077	0.206415541	GPBP1L1	intron_variant
8	A	G	20091236	rs313560275	0.206415541	GPBP1L1	intron_variant
8	A	G	20091796	rs14650009	0.206415541	GPBP1L1	intron_variant
8	G	A	20091946	rs313115519	0.206415541	GPBP1L1	intron_variant
8	A	G	20092012	rs312397156	0.206415541	GPBP1L1	intron_variant
8	C	T	20092191	_Variant_1088	0.206415541	GPBP1L1	intron_variant
8	C	T	20092197	_Variant_1089	0.206415541	GPBP1L1	intron_variant
8	T	A	20092213	_Variant_1090	0.206415541	GPBP1L1	intron_variant
8	T	G	20092214	_Variant_1091	0.206415541	GPBP1L1	intron_variant
8	T	C	20092233	_Variant_1092	0.206415541	GPBP1L1	intron_variant
8	T	A	20092235	_Variant_1093	0.206415541	GPBP1L1	intron_variant
8	T	C	20092239	_Variant_1094	0.206415541	GPBP1L1	intron_variant
8	C	G	20092635	rs16638853	0.206415541	GPBP1L1	intron_variant
8	A	G	20092999	rs313416808	0.206415541	GPBP1L1	intron_variant

8	C	T	27672863	_Variant_1141	0.206415541	RPE65	downstream_gene_variant
8	A	T	27674172	rs15941051	0.206415541	RPE65	downstream_gene_variant
8	T	C	27688190	_Variant_1176	0.206415541	RPE65	upstream_gene_variant
8	C	T	27689612	rs731614401	0.206415541	RPE65	upstream_gene_variant
3	G	A	24810738	_Variant_623	0.202132674	PPM1B	intron_variant
3	A	G	24827818	rs14329058	0.202132674	PPM1B	downstream_gene_variant
1	G	T	117663562	rs318223330	0.198881027	EIF2S3L	downstream_gene_variant
1	G	A	117667623	_Variant_50	0.198881027	EIF2S3L	intron_variant
1	T	C	117667628	_Variant_51	0.198881027	EIF2S3L	intron_variant
10	C	G	11880888	_Variant_237	0.198881027	ABHD17C	upstream_gene_variant
4	C	G	9330823	rs316524575	0.198881027	SH3BGRL	upstream_gene_variant
8	A	G	27685252	rs734748212	0.198881027	RPE65	upstream_gene_variant
10	G	A	11850949	rs316494348	0.196687368	ABHD17C	intron_variant
10	A	G	11851156	_Variant_183	0.196687368	ABHD17C	intron_variant
10	T	C	11851158	_Variant_184	0.196687368	ABHD17C	intron_variant
7	G	A	13818610	_Variant_954	0.196687368	ITGA4	3_prime_UTR_variant
8	T	C	20080602	rs733364184	0.196687368	ITGA4	downstream_gene_variant
8	A	C	20084516	rs313767515	0.194472012	ITGA4	downstream_gene_variant
8	T	C	20093420	rs315021708	0.194472012	GPBP1L1	intron_variant
8	T	C	20097438	rs315405537	0.194472012	GPBP1L1	intron_variant
10	G	A	11855064	rs15580768	0.187058993	ABHD17C	intron_variant
20	T	G	1847879	_Variant_433	0.187058993	AHCY	intron_variant
21	C	G	1985601	rs315294108	0.187058993	ENSGALG00000001359	intron_variant
3	G	A	24820854	_Variant_725	0.187058993	PPM1B	downstream_gene_variant
5	T	C	14081952	rs14518478	0.187058993	ENSGALG00000006740	missense_variant
5	G	A	14172817	rs14518525	0.187058993	ENSGALG00000006740	synonymous_variant
8	G	A	20080486	rs738416276	0.187058993	ITGA4	downstream_gene_variant
8	T	A	20080537	rs735232738	0.187058993	ITGA4	downstream_gene_variant
8	C	T	20087372	rs16638844	0.187058993	GPBP1L1	downstream_gene_variant
4	T	A	9329501	_Variant_870	0.185023565	SH3BGRL	upstream_gene_variant
8	A	G	20101678	rs16638863	0.185023565	GPBP1L1	intron_variant
1	C	A	117666927	rs740446522	0.184842364	EIF2S3L	intron_variant
4	T	C	9326603	rs15489952	0.184842364	SH3BGRL	upstream_gene_variant
4	A	G	9326606	rs15489954	0.184842364	SH3BGRL	upstream_gene_variant
4	A	G	9326610	rs15489956	0.184842364	SH3BGRL	upstream_gene_variant
5	G	A	14082032	_Variant_884	0.184842364	ENSGALG00000006740	missense_variant
8	T	A	20082579	rs13682056	0.184842364	ITGA4	downstream_gene_variant
10	T	A	11853384	_Variant_221	0.178800624	ABHD17C	intron_variant
10	A	T	11853385	_Variant_222	0.178800624	ABHD17C	intron_variant
20	T	G	1651841	_Variant_244	0.178800624	AHCY	intron_variant
20	G	C	1760210	rs730897951	0.178800624	AHCY	intron_variant

20	C	T	1842298	rs3136841	0.178800624	AHCY	synonymus_variant
3	T	C	24813211	_Variant_648	0.178800624	PPM1B	intron_variant
3	T	C	24813548	rs736640601	0.178800624	PPM1B	intron_variant
3	T	G	24814626	rs313614205	0.178800624	PPM1B	intron_variant
3	A	G	24815995	rs315975814	0.178800624	PPM1B	intron_variant
3	C	T	24819851	rs16240342	0.178800624	PPM1B	3_prime_UTR_variant
3	G	T	24822012	rs16240348	0.178800624	PPM1B	downstream_gene_variant
3	G	T	24822020	rs315388155	0.178800624	PPM1B	downstream_gene_variant
3	C	T	24822241	rs15301086	0.178800624	PPM1B	downstream_gene_variant
8	T	C	20083189	rs316763378	0.178800624	ITGA4	downstream_gene_variant
8	T	C	27667250	_Variant_1134	0.178800624	GPBP1L1	upstream_gene_variant
8	G	A	27672822	_Variant_1140	0.178800624	RPE65	downstream_gene_variant
8	A	G	27673391	_Variant_1143	0.178800624	RPE65	downstream_gene_variant
4	T	C	9328801	rs313266566	0.173344643	SH3BGRL	upstream_gene_variant
1	C	G	117677731	rs731374202	0.171797849	EIF2S3L	intron_variant
4	C	T	9326471	rs739096457	0.171797849	SH3BGRL	upstream_gene_variant
1	C	T	117673781	_Variant_66	0.166186551	EIF2S3L	intron_variant
20	G	A	1762884	rs794152307	0.166186551	AHCY	intron_variant
1	C	T	117664165	rs15399185	0.163844424	EIF2S3L	downstream_gene_variant
1	T	C	117664546	_Variant_18	0.163844424	EIF2S3L	downstream_gene_variant
1	C	T	117665494	rs316350574	0.163844424	EIF2S3L	3_prime_UTR_variant
1	A	G	117665825	rs314709245	0.163844424	EIF2S3L	intron_variant
1	G	A	117665962	rs736645568	0.163844424	EIF2S3L	intron_variant
1	A	T	117666888	rs13928093	0.163844424	EIF2S3L	intron_variant
1	A	G	117668017	rs10724353	0.163844424	EIF2S3L	synonymous_variant
1	A	G	117676184	rs317273623	0.163844424	EIF2S3L	intron_variant
1	C	G	117676584	rs15399230	0.163844424	EIF2S3L	intron_variant
1	A	G	117676676	rs13686038	0.163844424	EIF2S3L	intron_variant
1	A	C	117677022	rs315754118	0.163844424	EIF2S3L	intron_variant
20	C	G	1759582	rs738211168	0.163844424	AHCY	intron_variant
20	T	C	1759736	_Variant_315	0.163844424	AHCY	intron_variant
20	T	C	1761088	_Variant_354	0.163844424	AHCY	intron_variant
20	C	G	1761100	_Variant_355	0.163844424	AHCY	intron_variant
20	C	T	1761103	_Variant_356	0.163844424	AHCY	intron_variant
20	A	G	1763587	_Variant_391	0.163844424	AHCY	intron_variant
20	C	T	1766887	rs15169609	0.163844424	AHCY	intron_variant
20	T	C	1768596	rs15169613	0.163844424	AHCY	intron_variant
20	A	G	1786988	rs315502946	0.163844424	AHCY	intron_variant
20	T	A	1858482	rs14268757	0.163844424	AHCY	synonymous_variant
21	A	G	1986357	rs315421843	0.163844424	ENSGALG00000001359	intron_variant
21	C	T	1986381	rs316266183	0.163844424	ENSGALG00000001359	intron_variant

21	A	G	1986397	rs731235665	0.163844424	ENSGALG00000001359	intron_variant
21	C	T	1986403	rs736307167	0.163844424	ENSGALG00000001359	intron_variant
8	A	G	20082375	rs737720269	0.163844424	ITGA4	downstream_gene_variant
8	T	C	20083363	rs735550270	0.163844424	ITGA4	downstream_gene_variant
8	T	C	20091490	rs14650005	0.163844424	GPBP1L1	intron_variant
8	C	T	27668961	rs316169771	0.163844424	GPBP1L1	upstream_gene_variant
8	C	T	27689798	_Variant_1188	0.163844424	RPE65	upstream_gene_variant
1	G	C	117675621	rs314639527	0.157456394	EIF2S3L	intron_variant
1	G	T	117675624	rs313153699	0.157456394	EIF2S3L	splice_region_variant,intron_variant
1	G	A	117664087	_Variant_14	0.155652693	EIF2S3L	downstream_gene_variant
1	G	A	117677066	rs732569350	0.155652693	EIF2S3L	intron_variant
20	C	A	1760162	rs737748453	0.155652693	AHCY	intron_variant
20	G	A	1760729	rs16159303	0.155652693	AHCY	intron_variant
20	G	C	1760834	rs14268625	0.155652693	AHCY	intron_variant
20	C	T	1760863	rs14268626	0.155652693	AHCY	intron_variant
3	A	G	24812636	_Variant_643	0.155652693	PPM1B	intron_variant
3	G	T	24812640	rs731455327	0.155652693	PPM1B	intron_variant
3	T	G	24812641	rs735730542	0.155652693	PPM1B	intron_variant
3	A	G	24814702	rs313190762	0.155652693	PPM1B	intron_variant
4	C	T	9317625	rs318213194	0.155652693	SH3BGRL	downstream_gene_variant
4	T	G	9322932	rs316171029	0.155652693	SH3BGRL	upstream_gene_variant
8	G	A	20080833	_Variant_1020	0.155652693	ITGA4	downstream_gene_variant
8	G	A,C	20081117	rs317997592	0.155652693	ITGA4	downstream_gene_variant
8	C	T	20091459	rs739423203	0.155652693	GPBP1L1	splice_acceptor_variant
8	T	C	20091859	rs16638850	0.155652693	GPBP1L1	intron_variant
8	T	C	20096988	_Variant_1121	0.155652693	GPBP1L1	synonymous_variant
8	G	C	27689376	_Variant_1182	0.155652693	RPE65	upstream_gene_variant
1	T	A	117665690	_Variant_26	0.151972259	EIF2S3L	3_prime_UTR_variant
10	T	C	11852882	rs318116070	0.151972259	ABHD17C	intron_variant
20	C	T	1760690	rs16159302	0.151972259	AHCY	intron_variant
20	G	A	1760902	rs14268627	0.151972259	AHCY	intron_variant
20	C	A	1761063	rs794376205	0.151972259	AHCY	intron_variant
20	G	T	1761065	rs794295635	0.151972259	AHCY	intron_variant
20	A	C	1761066	rs794550974	0.151972259	AHCY	intron_variant
20	T	C	1860685	rs15169710	0.151972259	AHCY	synonymous_variant
20	T	C	1861437	rs16159455	0.151972259	AHCY	3_prime_UTR_variant
20	G	A	1863679	_Variant_465	0.151972259	AHCY	downstream_gene_variant
21	C	G	2003487	_Variant_587	0.151972259	ENSGALG00000001359	downstream_gene_variant
8	T	C	20080118	rs316446487	0.151972259	ITGA4	downstream_gene_variant
8	C	T	20083905	_Variant_1047	0.151972259	ITGA4	downstream_gene_variant
8	A	G	20083906	_Variant_1048	0.151972259	ITGA4	downstream_gene_variant

8	C	G	20083912	_Variant_1049	0.151972259	ITGA4	downstream_gene_variant
8	C	A	20091845	rs314482587	0.151972259	GPBP1L1	intron_variant
8	G	C	20093058	rs314317837	0.151972259	GPBP1L1	intron_variant
8	T	C	27689725	_Variant_1184	0.151972259	RPE65	upstream_gene_variant
8	C	T	27689780	_Variant_1187	0.151972259	RPE65	upstream_gene_variant
20	G	T	1761644	_Variant_373	0.148046063	AHCY	intron_variant
8	G	A	20094703	rs312860974	0.148046063	GPBP1L1	intron_variant
20	C	G	1762531	_Variant_375	0.145270828	AHCY	intron_variant
20	T	G	1762533	rs731898464	0.145270828	AHCY	intron_variant
21	A	G	1986839	rs317977917	0.145270828	ENSGALG00000001359	intron_variant
3	T	A	24821562	rs312990304	0.145270828	PPM1B	downstream_gene_variant
3	T	C	24821602	rs314021186	0.145270828	PPM1B	downstream_gene_variant
3	T	C	24821617	rs316855907	0.145270828	PPM1B	downstream_gene_variant
3	T	G	24821640	rs317664958	0.145270828	PPM1B	downstream_gene_variant
3	G	A	24810115	rs13720052	0.137174456	PPM1B	intron_variant
8	G	A	20085471	rs14649993	0.137174456	GPBP1L1	downstream_gene_variant
10	A	G	11877305	rs10730556	0.127151558	ABHD17C	synonymous_variant
20	T	G	1863954	rs315978308	0.127151558	AHCY	downstream_gene_variant
21	A	C	1968358	rs316650458	0.127151558	AHCY	downstream_gene_variant
3	T	C	24820349	rs316266491	0.127151558	PPM1B	downstream_gene_variant
3	G	A	24820356	rs317446089	0.127151558	PPM1B	downstream_gene_variant
3	A	G	24820961	rs314516041	0.127151558	PPM1B	downstream_gene_variant
3	T	C	24828392	_Variant_767	0.127151558	PPM1B	downstream_gene_variant
8	A	T	20094106	_Variant_1111	0.122460167	GPBP1L1	intron_variant
1	A	G	117679846	_Variant_136	0.1215964	EIF2S3L	upstream_gene_variant
10	A	G	11852766	rs733183851	0.1215964	ABHD17C	intron_variant
10	C	T	11852989	rs317007151	0.1215964	ABHD17C	intron_variant
10	A	T	11853193	rs14948627	0.1215964	ABHD17C	intron_variant
10	T	A	11853200	rs14948628	0.1215964	ABHD17C	intron_variant
10	C	T	11853326	rs315260182	0.1215964	ABHD17C	intron_variant
20	A	C	1677693	_Variant_287	0.1215964	AHCY	intron_variant
20	G	C	1761169	_Variant_357	0.1215964	AHCY	intron_variant
7	A	G	13840910	rs317522770	0.1215964	ITGA4	downstream_gene_variant
8	C	T	20085490	rs312312277	0.1215964	GPBP1L1	downstream_gene_variant
1	C	T	117663645	rs314254910	0.119906068	EIF2S3L	downstream_gene_variant
10	A	G	11849677	rs314178825	0.119906068	ABHD17C	intron_variant
10	G	T	11850838	rs14948612	0.119906068	ABHD17C	intron_variant
10	G	C	11852926	rs316583503	0.119906068	ABHD17C	intron_variant
10	A	G	11853015	rs14948623	0.119906068	ABHD17C	intron_variant
20	C	T	1677912	rs15169599	0.119906068	AHCY	intron_variant
20	A	G	1761324	rs735555744	0.119906068	AHCY	intron_variant

21	T	C	1985188	rs312916127	0.119906068	ENSGALG00000001359	intron_variant
21	C	T	2006432	rs312673425	0.119906068	ENSGALG00000001359	downstream_gene_variant
3	T	C	24822324	rs313666160	0.119906068	PPM1B	downstream_gene_variant
3	C	T	24827968	rs313542330	0.119906068	PPM1B	downstream_gene_variant
4	T	C	9328463	_Variant_854	0.119906068	SH3BGRL	upstream_gene_variant
4	G	C	9328497	_Variant_855	0.119906068	SH3BGRL	upstream_gene_variant
8	G	A	27685210	rs314293048	0.119906068	RPE65	upstream_gene_variant
1	C	T	117679359	rs739827408	0.113679674	EIF2S3L	upstream_gene_variant
1	C	G	117680252	rs731759373	0.113679674	EIF2S3L	upstream_gene_variant
1	A	G	117680254	rs735287572	0.113679674	EIF2S3L	upstream_gene_variant
5	C	T	14082120	rs15669517	0.113679674	ENSGALG00000006740	missense_variant
3	G	A	24821233	_Variant_728	0.107620563	PPM1B	downstream_gene_variant
3	T	C	24821258	_Variant_729	0.107620563	PPM1B	downstream_gene_variant
3	T	C	24821270	_Variant_730	0.107620563	PPM1B	downstream_gene_variant
20	G	A	1761301	rs738548626	0.100662774	AHCY	intron_variant
20	G	A	1761302	rs794108101	0.100662774	AHCY	intron_variant
21	G	A	1981350	rs313473636	0.100662774	ENSGALG00000001359	synonymous_variant
21	T	C	1992954	rs314639212	0.100662774	ENSGALG00000001359	intron_variant
21	C	T	2003297	rs15181435	0.100662774	ENSGALG00000001359	downstream_gene_variant
21	A	T	2003314	rs15181436	0.100662774	ENSGALG00000001359	downstream_gene_variant
21	C	T	2003315	rs15181437	0.100662774	ENSGALG00000001359	downstream_gene_variant
8	C	T	27665135	rs16649663	0.100662774	GPBP1L1	upstream_gene_variant
20	C	T	1657495	_Variant_248	0.100636158	AHCY	intron_variant
20	T	C	1657534	_Variant_249	0.100636158	AHCY	intron_variant
20	A	G	1657557	rs735921861	0.100636158	AHCY	intron_variant
20	C	T	1657558	rs739306430	0.100636158	AHCY	intron_variant
20	A	G	1657559	rs733108593	0.100636158	AHCY	intron_variant
3	C	T	24827845	rs741359032	0.095371752	PPM1B	downstream_gene_variant
5	T	C	14153967	rs317696334	0.095371752	ENSGALG00000006740	missense_variant
1	C	A	117663843	rs312775142	0.081236683	EIF2S3L	downstream_gene_variant
10	G	C	11854297	rs737939835	0.081236683	ABHD17C	intron_variant
20	G	A	1678335	rs733569486	0.081236683	AHCY	intron_variant
20	T	C	1763211	_Variant_383	0.081236683	AHCY	intron_variant
20	G	A	1861902	rs314189407	0.081236683	AHCY	3_prime_UTR_variant
20	G	T	1862320	_Variant_459	0.081236683	AHCY	downstream_gene_variant
20	A	G	1862335	rs313519453	0.081236683	AHCY	downstream_gene_variant
3	T	G	24827782	_Variant_759	0.081236683	PPM1B	downstream_gene_variant
10	G	A	11854628	rs14948633	0.080557875	ABHD17C	intron_variant
20	A	G	1863588	_Variant_462	0.080557875	AHCY	downstream_gene_variant
21	G	C	2002127	rs15181430	0.080557875	ENSGALG00000001359	downstream_gene_variant
3	A	G	24810743	_Variant_624	0.080557875	PPM1B	intron_variant

1	A	G	117668086	rs317345401	0.076844738	EIF2S3L	intron_variant
10	C	T	11849806	rs14007773	0.076844738	ABHD17C	intron_variant
20	A	C	1677887	_Variant_294	0.076844738	AHCY	intron_variant
20	C	T	1677891	_Variant_295	0.076844738	AHCY	intron_variant
20	C	T	1762496	_Variant_374	0.076844738	AHCY	intron_variant
3	A	T	24821636	rs314444240	0.076844738	PPM1B	downstream_gene_variant
4	T	A	9329508	rs739592007	0.076844738	SH3BGRL	upstream_gene_variant
1	G	A	117679381	rs738080997	0.075700455	EIF2S3L	upstream_gene_variant
20	G	T	1669097	_Variant_267	0.075700455	AHCY	intron_variant
20	T	C	1763143	_Variant_381	0.075700455	AHCY	intron_variant
21	C	G	1968231	rs312549129	0.075700455	AHCY	downstream_gene_variant
21	T	C	1992801	rs314801400	0.075700455	ENSGALG00000001359	intron_variant
3	G	A	24821221	_Variant_727	0.075700455	PPM1B	downstream_gene_variant
7	G	T	13843346	rs13737674	0.071851898	ITGA4	downstream_gene_variant
21	A	G	1985045	rs314155112	0.067314594	ENSGALG00000001359	intron_variant
10	C	T	11853257	rs316146184	0.062089336	ABHD17C	intron_variant
20	C	T	1677902	_Variant_296	0.062089336	AHCY	intron_variant
20	G	A	1677903	_Variant_297	0.062089336	AHCY	intron_variant
20	G	A	1761628	_Variant_372	0.062089336	AHCY	intron_variant
5	G	A	14142142	_Variant_910	0.062089336	ENSGALG00000006740	intron_variant
8	C	A	20079987	rs740952260	0.062089336	ITGA4	downstream_gene_variant
1	T	C	117679370	_Variant_129	0.061851596	EIF2S3L	upstream_gene_variant
1	G	C	117679371	_Variant_130	0.061851596	EIF2S3L	upstream_gene_variant
20	T	C	1671082	_Variant_277	0.061851596	AHCY	intron_variant
20	G	A	1671086	rs740456922	0.061851596	AHCY	intron_variant
20	G	A	1677611	rs315056444	0.061851596	AHCY	intron_variant
20	G	C	1759616	rs737690151	0.061851596	AHCY	intron_variant
1	C	T	117679126	rs313150319	0.051253114	EIF2S3L	upstream_gene_variant
20	C	A	1759618	_Variant_313	0.051253114	AHCY	intron_variant
20	G	T	1763212	_Variant_384	0.051253114	AHCY	intron_variant
1	T	C	117679341	rs316108748	0.049491483	EIF2S3L	upstream_gene_variant
3	G	C	24821313	rs15301073	0.049491483	PPM1B	downstream_gene_variant
8	A	G	20080552	rs736832502	0.049491483	ITGA4	downstream_gene_variant
8	G	A	20093637	rs731829690	0.049491483	GPBP1L1	intron_variant
1	T	C	117663636	rs732466055	0.046352214	EIF2S3L	downstream_gene_variant
10	T	C	11884141	rs313853555	0.046352214	ABHD17C	upstream_gene_variant
20	A	G	1763210	_Variant_382	0.046352214	AHCY	intron_variant
21	G	A	2001847	rs315083194	0.046352214	ENSGALG00000001359	downstream_gene_variant
3	T	C	24822440	rs15301089	0.046352214	PPM1B	downstream_gene_variant
4	G	A	9329691	rs315040540	0.046352214	SH3BGRL	upstream_gene_variant
1	C	T	117679787	rs314809471	0.041157534	EIF2S3L	upstream_gene_variant

20	C	G	1677866	_Variant_292	0.041157534	AHCY	intron_variant
20	A	G	1677913	rs15169600	0.041157534	AHCY	intron_variant
20	C	G	1760251	rs16159295	0.041157534	AHCY	intron_variant
20	C	T	1761278	rs316255774	0.041157534	AHCY	intron_variant
20	C	T	1761445	rs736294388	0.041157534	AHCY	intron_variant
4	A	G	9326813	rs738682626	0.041157534	SH3BGRL	upstream_gene_variant
4	G	A	9329794	rs740331318	0.041157534	SH3BGRL	upstream_gene_variant
5	C	T	14153913	_Variant_940	0.041157534	ENSGALG00000006740	missense_variant
8	G	A	20092677	rs312546580	0.041157534	GPBP1L1	intron_variant
8	G	T	27686276	rs317326622	0.041157534	RPE65	upstream_gene_variant
10	C	T	11880758	rs314773878	0.030447147	ABHD17C	upstream_gene_variant
4	A	G	9318373	_Variant_780	0.030447147	SH3BGRL	missense_variant
5	T	G	14141523	rs14518506	0.030447147	ENSGALG00000006740	intron_variant
1	A	G	117678877	rs734230599	0.028307771	EIF2S3L	upstream_gene_variant
10	T	G	11884174	rs14007825	0.028307771	ABHD17C	upstream_gene_variant
10	T	G	11884175	rs14007826	0.028307771	ABHD17C	upstream_gene_variant
20	A	C	1770137	_Variant_414	0.028307771	AHCY	intron_variant
21	A	C	1968329	_Variant_473	0.028307771	AHCY	downstream_gene_variant
20	T	A	1678364	rs740262806	0.027107458	AHCY	intron_variant
8	A	G	27688917	_Variant_1181	0.027107458	RPE65	upstream_gene_variant
20	G	A	1677879	_Variant_293	0.021747088	AHCY	intron_variant
20	C	G	1761454	rs736939780	0.021747088	AHCY	intron_variant
20	G	T	1761457	rs733317663	0.021747088	AHCY	intron_variant
20	T	C	1761533	rs733967720	0.021747088	AHCY	intron_variant
1	T	C	117663676	rs312560920	0	EIF2S3L	downstream_gene_variant
1	C	T	117663935	rs15399183	0	EIF2S3L	downstream_gene_variant
1	T	G	117664131	_Variant_15	0	EIF2S3L	downstream_gene_variant
1	G	A	117664429	rs317793053	0	EIF2S3L	downstream_gene_variant
1	G	A	117665982	rs315617986	0	EIF2S3L	intron_variant
1	T	G	117666357	rs316276794	0	EIF2S3L	intron_variant
1	A	T	117666379	rs315242738	0	EIF2S3L	intron_variant
1	T	G	117666385	rs312544356	0	EIF2S3L	intron_variant
1	A	T	117666917	rs739135485	0	EIF2S3L	intron_variant
1	G	A	117673783	_Variant_67	0	EIF2S3L	intron_variant
1	A	C	117673850	rs315180642	0	EIF2S3L	intron_variant
1	G	A	117674452	_Variant_74	0	EIF2S3L	intron_variant
1	C	T	117675635	rs13928136	0	EIF2S3L	synonymous_variant
1	A	G	117675914	rs313675157	0	EIF2S3L	intron_variant
1	G	C	117676645	rs15399232	0	EIF2S3L	intron_variant
1	A	G	117676720	rs316751281	0	EIF2S3L	intron_variant
1	C	T	117676935	rs13928137	0	EIF2S3L	intron_variant

1	G	A	117676941	rs13928138	0	EIF2S3L	intron_variant
1	T	C	117676958	rs13928139	0	EIF2S3L	intron_variant
1	C	T	117679296	rs316055458	0	EIF2S3L	upstream_gene_variant
1	G	A	117679301	rs313373868	0	EIF2S3L	upstream_gene_variant
1	C	T	117679627	rs736943085	0	EIF2S3L	upstream_gene_variant
10	C	T	11849158	rs316506999	0	ABHD17C	intron_variant
10	A	G	11849547	rs317346812	0	ABHD17C	intron_variant
10	T	G	11849549	rs730912823	0	ABHD17C	intron_variant
10	T	G	11849636	rs316008614	0	ABHD17C	intron_variant
10	T	C	11850033	rs314812048	0	ABHD17C	intron_variant
10	A	G	11850188	rs15580737	0	ABHD17C	intron_variant
10	G	A	11852222	rs317869546	0	ABHD17C	synonymous_variant
10	G	A	11852349	rs14948618	0	ABHD17C	intron_variant
10	C	G	11852370	rs739917006	0	ABHD17C	intron_variant
10	A	C	11852451	rs313751285	0	ABHD17C	intron_variant
10	A	G	11852598	rs317396595	0	ABHD17C	intron_variant
10	A	G	11852677	rs14948620	0	ABHD17C	intron_variant
10	A	T	11852678	rs14948621	0	ABHD17C	intron_variant
10	C	A	11852718	rs315418205	0	ABHD17C	intron_variant
10	C	T	11852784	rs14007776	0	ABHD17C	intron_variant
10	T	C	11853031	rs14948624	0	ABHD17C	intron_variant
10	T	C	11853218	rs316007003	0	ABHD17C	intron_variant
20	G	A	1667737	rs738503268	0	AHCY	intron_variant
20	A	G	1667874	_Variant_257	0	AHCY	intron_variant
20	A	G	1668224	rs312898728	0	AHCY	intron_variant
20	C	T	1668415	rs315413113	0	AHCY	intron_variant
20	G	T	1669657	rs314373001	0	AHCY	intron_variant
20	C	A	1669815	rs15169589	0	AHCY	intron_variant
20	T	C	1669930	rs15169591	0	AHCY	intron_variant
20	C	T	1676633	rs312501655	0	AHCY	intron_variant
20	T	G	1759456	_Variant_306	0	AHCY	intron_variant
20	G	A	1759490	rs431893094	0	AHCY	intron_variant
20	T	G	1759516	rs734701176	0	AHCY	intron_variant
20	G	A	1759588	rs739217027	0	AHCY	intron_variant
20	A	C	1759619	rs736799919	0	AHCY	intron_variant
20	T	G	1759878	rs10731137	0	AHCY	intron_variant
20	G	A	1760041	_Variant_320	0	AHCY	intron_variant
20	A	T,G	1760317	rs16159296	0	AHCY	intron_variant
20	C	T	1760508	_Variant_336	0	AHCY	intron_variant
20	T	C	1760514	rs16159299	0	AHCY	intron_variant
20	A	G	1760658	rs16159301	0	AHCY	intron_variant

20	C	T	1761050	_Variant_349	0	AHCY	intron_variant
20	T	G	1761060	_Variant_350	0	AHCY	intron_variant
20	T	C	1766577	rs317950898	0	AHCY	intron_variant
20	G	A	1766585	rs316123890	0	AHCY	intron_variant
20	G	A	1766599	_Variant_395	0	AHCY	intron_variant
20	T	C	1767043	rs313987705	0	AHCY	intron_variant
20	A	G	1767760	rs312963808	0	AHCY	intron_variant
20	A	G	1767908	rs314656347	0	AHCY	intron_variant
20	G	A	1768025	rs316963286	0	AHCY	intron_variant
20	A	T	1768143	rs734368369	0	AHCY	intron_variant
20	A	G	1768434	rs315127397	0	AHCY	intron_variant
20	G	T	1768616	rs313981260	0	AHCY	intron_variant
20	T	C	1768799	_Variant_409	0	AHCY	intron_variant
20	T	C	1770547	rs315315287	0	AHCY	intron_variant
20	G	A	1771003	rs316378753	0	AHCY	intron_variant
20	G	T	1771254	rs13630806	0	AHCY	intron_variant
20	A	G	1779193	rs314928394	0	AHCY	intron_variant
20	G	A	1781781	_Variant_426	0	AHCY	intron_variant
20	T	C	1781793	rs315881830	0	AHCY	intron_variant
20	T	C	1860902	rs29005645	0	AHCY	3_prime_UTR_variant
20	A	G	1860959	rs732008758	0	AHCY	3_prime_UTR_variant
20	G	C	1861130	rs29005648	0	AHCY	3_prime_UTR_variant
20	T	A	1861395	_Variant_447	0	AHCY	3_prime_UTR_variant
20	G	T	1861862	_Variant_451	0	AHCY	3_prime_UTR_variant
21	A	G	1986319	rs734067603	0	ENSGALG00000001359	intron_variant
21	G	A	1986421	rs317668010	0	ENSGALG00000001359	intron_variant
21	T	C	1986604	rs317209739	0	ENSGALG00000001359	intron_variant
21	T	C	1986750	rs312891117	0	ENSGALG00000001359	intron_variant
21	G	A	1987253	rs313826275	0	ENSGALG00000001359	intron_variant
21	C	T	1987500	rs317834187	0	ENSGALG00000001359	intron_variant
21	C	A	1987552	rs741282316	0	ENSGALG00000001359	intron_variant
21	A	G	1987553	rs734077892	0	ENSGALG00000001359	intron_variant
21	G	A	1987583	_Variant_512	0	ENSGALG00000001359	intron_variant
21	T	G	1989530	rs313278069	0	ENSGALG00000001359	intron_variant
21	C	A	1990015	_Variant_519	0	ENSGALG00000001359	intron_variant
21	C	G	1990024	_Variant_520	0	ENSGALG00000001359	splice_region_variant,intron_variant
21	A	G	1996923	_Variant_542	0	ENSGALG00000001359	downstream_gene_variant
21	T	C	2001046	rs15181427	0	ENSGALG00000001359	downstream_gene_variant
21	G	A	2001982	rs314029243	0	ENSGALG00000001359	downstream_gene_variant
21	G	A	2001996	rs314951637	0	ENSGALG00000001359	downstream_gene_variant
21	A	C	2003480	_Variant_586	0	ENSGALG00000001359	downstream_gene_variant

21	C	T	2004013	rs318016459	0	ENSGALG00000001359	downstream_gene_variant
21	T	C	2004771	_Variant_595	0	ENSGALG00000001359	downstream_gene_variant
3	C	G	24809938	rs13720050	0	PPM1B	intron_variant
3	C	T	24809962	rs16240333	0	PPM1B	intron_variant
3	T	G	24809990	rs16240334	0	PPM1B	intron_variant
3	T	C	24810424	rs14329046	0	PPM1B	intron_variant
3	A	G	24811875	rs13720055	0	PPM1B	intron_variant
3	C	T	24813658	_Variant_653	0	PPM1B	intron_variant
3	C	T	24814865	rs315887975	0	PPM1B	intron_variant
3	G	T	24816294	rs315484831	0	PPM1B	intron_variant
3	T	C	24821791	rs16240347	0	PPM1B	downstream_gene_variant
4	G	C	9317788	rs315268609	0	SH3BGRL	downstream_gene_variant
4	T	C	9322610	rs314394574	0	SH3BGRL	upstream_gene_variant
4	C	T	9323760	rs315352965	0	SH3BGRL	upstream_gene_variant
4	G	A	9323925	rs14426556	0	SH3BGRL	upstream_gene_variant
4	A	T	9324162	rs14426557	0	SH3BGRL	upstream_gene_variant
4	A	C	9324361	_Variant_818	0	SH3BGRL	upstream_gene_variant
4	G	A	9324939	rs313478631	0	SH3BGRL	upstream_gene_variant
4	G	C	9325071	rs314942218	0	SH3BGRL	upstream_gene_variant
4	A	G	9325278	rs313615354	0	SH3BGRL	upstream_gene_variant
4	T	C	9325508	_Variant_824	0	SH3BGRL	upstream_gene_variant
4	G	A	9326514	rs315164777	0	SH3BGRL	upstream_gene_variant
4	A	G	9329418	_Variant_868	0	SH3BGRL	upstream_gene_variant
4	T	A	9329500	_Variant_869	0	SH3BGRL	upstream_gene_variant
5	C	A	14141334	rs315990176	0	ENSGALG00000006740	intron_variant
5	T	C	14149735	rs312939318	0	ENSGALG00000006740	synonymous_variant
5	A	G	14150606	_Variant_927	0	ENSGALG00000006740	missense_variant
5	G	C	14150614	_Variant_928	0	ENSGALG00000006740	missense_variant
5	G	C	14150628	_Variant_930	0	ENSGALG00000006740	missense_variant
7	A	G	13819904	rs317912553	0	ITGA4	3_prime_UTR_variant
7	G	C	13820148	rs312501278	0	ITGA4	3_prime_UTR_variant
7	G	T	13820405	rs312809684	0	ITGA4	3_prime_UTR_variant
7	T	G	13821126	rs13737670	0	ITGA4	3_prime_UTR_variant
7	A	G	13822740	rs316338223	0	ITGA4	downstream_gene_variant
8	A	G	20080656	rs16638818	0	ITGA4	downstream_gene_variant
8	A	G	20082719	rs16638836	0	ITGA4	downstream_gene_variant
8	A	G	20083258	rs14649987	0	ITGA4	downstream_gene_variant
8	T	G	20084893	_Variant_1057	0	GPBP1L1	downstream_gene_variant
8	G	A,C	20084931	_Variant_1058	0	GPBP1L1	downstream_gene_variant
8	G	T	20085514	rs737033804	0	GPBP1L1	downstream_gene_variant
8	T	C	20091598	rs14650007	0	GPBP1L1	intron_variant

8	G	T	20093150	rs317854706	0	GPBP1L1	intron_variant
8	A	G	20093273	rs16638857	0	GPBP1L1	missense_variant
8	C	G	20093369	rs732056670	0	GPBP1L1	intron_variant
8	A	G	20094135	_Variant_1112	0	GPBP1L1	intron_variant
8	G	A	27675010	rs317082753	0	RPE65	downstream_gene_variant
8	A	G	27685124	rs315600324	0	RPE65	upstream_gene_variant
8	G	A	27685138	rs16649677	0	RPE65	upstream_gene_variant
8	T	C	27690787	rs741741877	0	RPE65	upstream_gene_variant
1	G	T	117663732	rs733256805		EIF2S3L	downstream_gene_variant
1	T	C	117663756	rs14878839		EIF2S3L	downstream_gene_variant
1	T	C	117665153	rs312358511		EIF2S3L	downstream_gene_variant
1	T	C	117665901	rs312603165		EIF2S3L	intron_variant
1	C	T	117665980	rs317310905		EIF2S3L	intron_variant
1	G	A	117666290	rs313800268		EIF2S3L	intron_variant
1	A	C	117669867	rs13686029		EIF2S3L	synonymous_variant
1	A	T	117673796	_Variant_68		EIF2S3L	intron_variant
1	C	T	117673803	_Variant_69		EIF2S3L	intron_variant
1	A	C	117673809	_Variant_70		EIF2S3L	intron_variant
1	A	G	117675737	rs13686037		EIF2S3L	synonymous_variant
1	T	C	117677315	rs315283025		EIF2S3L	intron_variant
1	C	T	117677370	_Variant_113		EIF2S3L	intron_variant
1	A	G	117677480	rs15399236		EIF2S3L	intron_variant
10	C	T	11847823	_Variant_147		ABHD17C	3_prime_UTR_variant
10	A	G	11848853	rs14007768		ABHD17C	intron_variant
10	T	C	11848949	rs14007770		ABHD17C	intron_variant
10	A	C	11849001	rs14007771		ABHD17C	intron_variant
10	C	T	11849212	rs314421245		ABHD17C	intron_variant
10	C	A	11849899	rs13545315		ABHD17C	intron_variant
10	G	T	11851965	rs315937541		ABHD17C	intron_variant
10	C	A	11852283	rs316776833		ABHD17C	intron_variant
10	A	T	11852423	rs14948619		ABHD17C	intron_variant
10	A	G	11853356	rs13545317		ABHD17C	intron_variant
10	T	C	11853845	_Variant_225		ABHD17C	intron_variant
20	A	G	1641686	rs315065765		AHCY	upstream_gene_variant
20	T	G	1668442	rs318128777		AHCY	intron_variant
20	A	G	1668938	rs15169585		AHCY	intron_variant
20	G	A	1669475	rs315901328		AHCY	intron_variant
20	T	C	1676801	_Variant_282		AHCY	intron_variant
20	T	C	1676857	rs315292243		AHCY	intron_variant
20	C	T	1677847	_Variant_291		AHCY	intron_variant
20	G	A	1678189	rs315822569		AHCY	intron_variant

20	G	A	1678506	_Variant_304		AHCY	intron_variant
20	T	C	1761395	rs737590850		AHCY	intron_variant
20	G	A	1763357	_Variant_388		AHCY	intron_variant
20	A	G	1768594	rs15169612		AHCY	intron_variant
20	C	T	1771231	_Variant_423		AHCY	intron_variant
20	T	C	1843966	rs315924171		AHCY	synonymus_variant
20	A	G,T	1866368	rs735600689		AHCY	downstream_gene_variant
21	A	G	1985753	rs312972572		ENSGALG00000001359	intron_variant
4	A	G	9317637	rs312634588		SH3BGRL	downstream_gene_variant
4	T	C	9317895	rs316402283		SH3BGRL	downstream_gene_variant
4	C	T	9317971	rs316074127		SH3BGRL	downstream_gene_variant
4	G	C	9318171	rs315338437		SH3BGRL	synonymous_variant
4	G	T	9319233	rs16359134		SH3BGRL	upstream_gene_variant
4	T	G	9322546	rs314512863		SH3BGRL	upstream_gene_variant
4	G	A	9322573	rs315886269		SH3BGRL	upstream_gene_variant
4	T	C	9322582	rs312719643		SH3BGRL	upstream_gene_variant
4	C	T	9322941	rs736147404		SH3BGRL	upstream_gene_variant
4	A	G	9323178	rs313170160		SH3BGRL	upstream_gene_variant
4	G	T	9323214	rs312299346		SH3BGRL	upstream_gene_variant
4	G	A	9323347	rs312954289		SH3BGRL	upstream_gene_variant
4	A	T	9327416	_Variant_851		SH3BGRL	upstream_gene_variant
4	C	T	9328714	_Variant_858		SH3BGRL	upstream_gene_variant
4	T	C	9328791	_Variant_860		SH3BGRL	upstream_gene_variant
4	T	C	9329240	rs14426559		SH3BGRL	upstream_gene_variant
4	G	A	9329271	rs14426560		SH3BGRL	upstream_gene_variant
4	A	G	9329273	rs314586543		SH3BGRL	upstream_gene_variant
5	C	T	14095072	_Variant_892		ENSGALG00000006740	synonymous_variant
5	G	A	14095093	_Variant_894		ENSGALG00000006740	synonymous_variant
5	T	C	14095134	_Variant_895		ENSGALG00000006740	missense_variant
7	C	T	13817321	rs15848664		ITGA4	intron_variant
7	A	G	13817646	rs312618577		ITGA4	splice_region_variant 3_prime_UTR_variant
7	G	A	13818799	_Variant_956		ITGA4	3_prime_UTR_variant
7	T	C	13818819	_Variant_957		ITGA4	3_prime_UTR_variant
7	C	T	13818846	rs315665123		ITGA4	3_prime_UTR_variant
7	A	G	13826245	_Variant_982		ITGA4	downstream_gene_variant
7	C	T	13860222	rs315241024		ITGA4	downstream_gene_variant
8	G	T	20091763	rs14650008		GPBP1L1	intron_variant
8	T	C	27666294	rs16649666		GPBP1L1	upstream_gene_variant
8	C	T	27667259	rs15941014		GPBP1L1	upstream_gene_variant
8	T	C	27685261	rs313833333		RPE65	upstream_gene_variant
8	G	T	27685325	rs314891224		RPE65	upstream_gene_variant

8	G	A	27685418	rs315699739		RPE65	upstream_gene_variant
8	A	G	27685601	rs14657770		RPE65	upstream_gene_variant
8	G	A	27686297	rs314360231		RPE65	upstream_gene_variant

Supplementary Table 17: Significant eQTLs identified in Duodenum of low and high FCR broilers

chr	Ref	Alt	pos	id	NegLog10 p-value	Gene	Location	Log2foldchange (low vs high)	p-value for Log2foldchange (low vs high)
4	G	A	33493534	rs739281432	1.811627256	CDKL2	intron	-1.079359159	0.001354861
4	G	T	33493536	rs731824742	1.811627256	CDKL2	intron	-1.079359159	0.001354861
4	C	G	33493544	rs313558088	1.811627256	CDKL2	intron	-1.079359159	0.001354861
3	G	A	31344039	rs316497105	1.745682121	EIF2AK2	upstream	-1.160689079	0.001543295
4	A	G	33493943	rs738401365	1.694058784	CDKL2	intron	-1.079359159	0.001354861
4	C	A	33493947	rs741004252	1.694058784	CDKL2	intron	-1.079359159	0.001354861
4	G	T	33493973	rs314887600	1.472730199	CDKL2	intron	-1.079359159	0.001354861
4	G	A	33493977	rs317109468	1.472730199	CDKL2	intron	-1.079359159	0.001354861
4	G	T	33494004	rs738418882	1.472730199	CDKL2	intron	-1.079359159	0.001354861
4	A	G	33494006	rs730944916	1.472730199	CDKL2	intron	-1.079359159	0.001354861
3	G	A	31345086	rs736774899	1.455746105	EIF2AK2	upstream	-1.160689079	0.001543295
3	T	C	31343215	rs316268789	1.330775335	EIF2AK2	upstream	-1.160689079	0.001543295

Supplementary Table 18: Significant eQTLs identified in Ileum of low and high FCR broilers

chr	Ref	Alt	pos	snp_id	NegLog10 p_value	Gene	Location	Log2foldchange (low vs high)	p-value for Log2foldchange (low vs high)
7	A	C	13183971	rs317374719	1.918156122	ENSGALG00000008738	Intron variant	-1.516751916	0.008705737
7	T	C	13187016	rs737034318	1.918156122	ENSGALG00000008738	Intron variant	-1.516751916	0.008705737
7	C	T	13187020	rs315868610	1.918156122	ENSGALG00000008738	Intron variant	-1.516751916	0.008705737
7	A	T	13187021	rs732851317	1.918156122	ENSGALG00000008738	Intron variant	-1.516751916	0.008705737
7	C	T	13187051	rs736429536	1.918156122	ENSGALG00000008738	Intron variant	-1.516751916	0.008705737
7	C	T	13184476	rs730965526	1.738597689	ENSGALG00000008738	Intron variant	-1.516751916	0.008705737
7	C	T	13185159	rs737662720	1.730567189	ENSGALG00000008738	Intron variant	-1.516751916	0.008705737
7	A	G	13186220	rs312536923	1.700390493	ENSGALG00000008738	Intron variant	-1.516751916	0.008705737
7	C	T	13184778	rs16586532	1.53616176	ENSGALG00000008738	Intron variant	-1.516751916	0.008705737
13	C	A	12231794	rs315635567	1.455746105	G3BP1	Upstream gene variant	-2.097224757	0.005573983
7	G	A	13186650	rs316285728	1.428758204	ENSGALG00000008738	Intron variant	-1.516751916	0.008705737
7	T	G	13184339	rs738577016	1.366060143	ENSGALG00000008738	Intron variant	-1.516751916	0.008705737
7	A	G	13184801	_Variant_385	1.366060143	ENSGALG00000008738	Intron variant	-1.516751916	0.008705737
7	G	C	13186223	rs313809209	1.355360527	ENSGALG00000008738	Intron variant	-1.516751916	0.008705737
13	C	G	12204487	rs741384794	1.350424807	G3BP1	Intron variant	-2.097224757	0.005573983

Supplementary Table 19: Significant eQTLs identified in Cecum of low and high FCR broilers

chr	Ref	Alt	pos	snp id	NegLog10 p Value	Gene	Location	Log2foldchange (low vs high)	p-value for Log2foldchange (low vs high)
21	A	G	2002983	rs15181433	2.097497634	ENSGALG00000001359	downstream gene variant	1.968077134	0.001782166
3	G	A	24822333	rs794597744	2.040554066	PPM1B	downstream gene variant	4.019963557	4.11953E-05
3	C	T	24811667	rs732689598	1.922023958	PPM1B	intron variant	4.019963557	4.11953E-05
3	A	G	24811608	rs16240336	1.811627256	PPM1B	intron variant	4.019963557	4.11953E-05
21	A	C	1967450	rs317724807	1.694058784	AHCY	downstream gene variant	-4.073171504	0.006496961
10	C	T	11850703	rs315381519	1.672628747	ABHD17C	intron variant	-3.476242006	0.004265429
3	G	A	24811662	rs740679964	1.672628747	PPM1B	intron variant	4.019963557	4.11953E-05
21	T	A	1999838	rs317003286	1.539290116	ENSGALG00000001359	downstream gene variant	1.968077134	0.001782166
21	C	T	1987615	Variant 513	1.512104586	ENSGALG00000001359	intron variant	1.968077134	0.001782166
21	G	A	1992608	rs741708921	1.512104586	ENSGALG00000001359	intron variant	1.968077134	0.001782166
21	A	T	1995584	Variant 534	1.512104586	ENSGALG00000001359	3 prime UTR variant	1.968077134	0.001782166
21	A	G	1995641	rs315667733	1.512104586	ENSGALG00000001359	3 prime UTR variant	1.968077134	0.001782166
21	T	C	1998302	rs735224699	1.512104586	ENSGALG00000001359	downstream gene variant	1.968077134	0.001782166
21	A	G	1999598	Variant 557	1.512104586	ENSGALG00000001359	downstream gene variant	1.968077134	0.001782166
21	A	C	1999618	Variant 558	1.512104586	ENSGALG00000001359	downstream gene variant	1.968077134	0.001782166
21	A	C	1999620	rs15181423	1.512104586	ENSGALG00000001359	downstream gene variant	1.968077134	0.001782166
21	C	T	2001925	rs738048421	1.512104586	ENSGALG00000001359	downstream gene variant	1.968077134	0.001782166
21	C	G	2002007	rs740325027	1.512104586	ENSGALG00000001359	downstream gene variant	1.968077134	0.001782166
21	G	C	2002014	rs732784124	1.512104586	ENSGALG00000001359	downstream gene variant	1.968077134	0.001782166
21	T	C	2003569	rs732936078	1.512104586	ENSGALG00000001359	downstream gene variant	1.968077134	0.001782166

21	G	A	2004446	Variant 593	1.512104586	ENSGALG00000001359	downstream gene variant	1.968077134	0.001782166
21	G	T	2004480	rs732546063	1.512104586	ENSGALG00000001359	downstream gene variant	1.968077134	0.001782166
3	G	A	24809972	rs313943112	1.512104586	PPM1B	intron variant	4.019963557	4.11953E-05
3	T	C	24816341	rs741067461	1.512104586	PPM1B	intron variant	4.019963557	4.11953E-05
7	C	T	13826038	rs733814084	1.512104586	ITGA4	downstream gene variant	-4.014323934	0.004774167
7	A	T	13826068	rs732496374	1.512104586	ITGA4	downstream gene variant	-4.014323934	0.004774167
21	A	T	2002128	rs314038704	1.497305722	ENSGALG00000001359	downstream gene variant	1.968077134	0.001782166
3	G	T	24811659	rs737292718	1.472730199	PPM1B	intron variant	4.019963557	4.11953E-05
5	C	G	14095079	Variant 893	1.458430538	ENSGALG00000006740	missense variant	-3.435790233	6.04308E-06
8	G	A	20095084	rs315798025	1.455746105	GPBP1L1	intron variant	2.61680314	0.001752327
3	T	C	24819471	rs15301049	1.393360937	PPM1B	3 prime UTR variant	4.019963557	4.11953E-05
10	A	G	11855189	rs317349624	1.361050473	ABHD17C	intron variant	-3.476242006	0.004265429
21	C	T	2002663	rs738060517	1.361050473	ENSGALG00000001359	downstream gene variant	1.968077134	0.001782166
3	G	A	24811640	rs317341558	1.361050473	PPM1B	intron variant	4.019963557	4.11953E-05
4	T	A	9330391	rs15489961	1.361050473	SH3BGRL	upstream gene variant	2.232425938	2.0706E-06
10	C	T	11850414	rs314604288	1.355360527	ABHD17C	intron variant	-3.476242006	0.004265429
21	T	G	1997644	Variant 544	1.355360527	ENSGALG00000001359	downstream gene variant	1.968077134	0.001782166
7	G	A	13826306	rs738105407	1.355360527	ITGA4	downstream gene variant	-4.014323934	0.004774167
3	T	C	24812259	Variant 638	1.345272011	PPM1B	intron variant	4.019963557	4.11953E-05

Supplementary Table 20: Functional annotations and pathways associated with identified Liver DEGs (low vs. high)

ID	Gene Name	GOTERM_BP	GOTERM_CC	GOTERM_MF	INTERPRO	KEGG PATHWAY	PIR SUPERFAMILY	SMART
ENSGALG00000016329	1-acylglycerol-3-phosphate O-acyltransferase 5(AGPAT5)	GO:0002244~hematopoietic progenitor cell differentiation,GO:0006639~acylglycerol metabolic process,	GO:0005739~mitochondrion,GO:0016021~integral component of membrane,	GO:0003841~1-acylglycerol-3-phosphate O-acyltransferase activity,	IPR002123:Phospholipid/glycerol acyltransferase,	gga00561:Glycerolipid metabolism,gga00564:Glycerophospholipid metabolism,gga01100:Metabolic pathways,		SM00563:PlsC,
ENSGALG00000000231	BCL2/adenovirus E1B 19kDa interacting protein 3-like(BNIP3L)	GO:0016239~positive regulation of macroautophagy,GO:0035694~mitochondrial protein catabolic process,GO:0043065~positive regulation of apoptotic process,GO:0043066~negative regulation of apoptotic process,GO:0043069~negative regulation of programmed cell death,GO:0051607~defense response to virus,GO:0071456~cellular response to hypoxia,GO:0097345~mitochondrial outer membrane permeabilization,GO:1903146~regulation of mitophagy,GO:1903214~regulation of protein targeting to mitochondrion,	GO:0005635~nuclear envelope,GO:0005740~mitochondrial envelope,GO:0005741~mitochondrial outer membrane,GO:0005783~endoplasmic reticulum,GO:0016021~integral component of membrane,		IPR010548:BNIP3,			

ENSGALG00000011275	DEP domain containing 1(DEPDC1)	GO:0035556~intracellular signal transduction,GO:0045892~negative regulation of transcription, DNA-templated,	GO:0005654~nucleoplasm,GO:0017053~transcriptional repressor complex,		IPR000198:Rho GTPase-activating protein domain,IPR000591:DEP domain,IPR008936:Rho GTPase activation protein,IPR011991:Winged helix-turn-helix DNA-binding domain,			SM00049:DEP,
ENSGALG00000017329	FCH and double SH3 domains 2(FCHSD2)	GO:0007274~neuromuscular synaptic transmission,GO:0030833~regulation of actin filament polymerization,	GO:0031594~neuromuscular junction,GO:0055037~recycling endosome,		IPR001060:FCH domain,IPR001452:Src homology-3 domain,			SM00055:FCH,SM00326:SH3,
ENSGALG00000003861	HECT and RLD domain containing E3 ubiquitin protein ligase 4(HERC4)	GO:0007283~spermatogenesis,GO:0042787~protein ubiquitination involved in ubiquitin-dependent protein catabolic process,	GO:0005634~nucleus,GO:0005737~cytoplasm,	GO:0004842~ubiquitin-protein transferase activity,GO:0016874~ligase activity,	IPR000408:Regulator of chromosome condensation, RCC1,IPR000569:HECT,IPR009091:Regulator of chromosome condensation 1/beta-lactamase-inhibitor protein II,	gga04120:Ubiquitin mediated proteolysis,		SM00119:HECTc,
ENSGALG00000007386	LUC7-like 3 pre-mRNA splicing factor(LUC7L3)	GO:0006376~mRNA splice site selection,	GO:0005654~nucleoplasm,GO:0005685~U1 snRNP,GO:0071004~U2-type prespliceosome,	GO:0003729~mRNA binding,	IPR004882:LUC7-related,			
ENSGALG00000004001	La ribonucleoprotein domain family,	GO:0006413~translational initiation,GO:0008283~cell proliferation,GO:0016239~positive regulation	GO:0005654~nucleoplasm,GO:0005737~cytoplasm,GO:0016020~membrane,GO:0031931~TORC1 complex,	GO:0000339~RNA cap binding,GO:0003730~mRNA 3'-UTR	IPR006607:Protein of unknown function DM15,IPR006630:RNA-binding protein Lupus			SM00684:DM15,SM00715:LA,

	member 1(LARP 1)	of macroautophagy,GO:0031929~TOR signaling,GO:0045070~positive regulation of viral genome replication,		binding,GO:0008494~translation activator activity,GO:0048027~mRNA 5'-UTR binding,	La,IPR011991:Winged helix-turn-helix DNA-binding domain,			
ENSGALG00000015733	MDN1, midasin homolog (yeast)(MDN1)	GO:0000027~ribosomal large subunit assembly,GO:0006364~rRNA processing,	GO:0005730~nucleolus,GO:0005737~cytoplasm,GO:0016020~membrane,GO:0030687~preribosome, large subunit precursor,GO:0045111~intermediate filament cytoskeleton,	GO:0005524~ATP binding,GO:0016887~ATPase activity,	IPR002035:von Willebrand factor, type A,IPR003593:AAA+ ATPase domain,IPR011704:ATPase, dynein-related, AAA domain,IPR012099:Midasin,IPR027417:P-loop containing nucleoside triphosphate hydrolase,	gga03008:Ribosome biogenesis in eukaryotes,	PIRSF010340:midasin,	SM00327:VWA,SM00382:AAA,
ENSGALG00000015649	NOP14 nucleolar protein(NOP14)	GO:0030490~maturation of SSU-rRNA,	GO:0005730~nucleolus,GO:0016020~membrane,GO:0030692~Noc4p-Nop14p complex,GO:0032040~small-subunit processome,	GO:0044822~poly(A) RNA binding,	IPR007276:Nucleolar protein 14,			
ENSGALG00000017070	PDS5, regulator of cohesion maintenance, homolog B (S. cerevisiae)(PDS5B)	GO:0006281~DNA repair,GO:0007064~mitotic sister chromatid cohesion,GO:0008285~negative regulation of cell proliferation,GO:0051301~cell division,	GO:0000785~chromatin,GO:0005634~nucleus,GO:0005654~nucleoplasm,		IPR011989:Armadillo-like helical,IPR016024:Armadillo-type fold,			
ENSGALG00000002862	RRN3 RNA polymerase I transcrip	GO:0006361~transcription initiation from RNA polymerase I promoter,GO:0007000~nucleolus	GO:0005730~nucleolus,	GO:0001164~RNA polymerase I CORE element	IPR007991:RNA polymerase I specific transcription initiation factor			

	tion factor homolog (S. cerevisiae)(RRN3)	organization,GO:0007028~cytoplasm organization,GO:0008283~cell proliferation,GO:0042254~ribosome biogenesis,GO:0045893~positive regulation of transcription, DNA-templated,GO:0048872~homeostasis of number of cells,GO:1902254~negative regulation of intrinsic apoptotic signaling pathway by p53 class mediator,GO:2000142~regulation of DNA-templated transcription, initiation,		sequence-specific DNA binding,	RRN3,IPR016024:Armadillo-type fold,			
ENSGALG00000001500	SLU7 homolog, splicing factor(SLU7)	GO:0000375~RNA splicing, via transesterification reactions,GO:0000380~alternative mRNA splicing, via spliceosome,GO:0000389~mRNA 3'-splice site recognition,GO:0006397~mRNA processing,GO:0006886~intracellular protein transport,GO:0034605~cellular response to heat,	GO:0005681~spliceosomal complex,GO:0005737~cytoplasm,GO:0016020~membrane,GO:0016607~nuclear speck,GO:0030532~small nuclear ribonucleoprotein complex,GO:0071013~catalytic step 2 spliceosome,	GO:0000386~second spliceosomal transesterification activity,GO:0008270~zinc ion binding,GO:0030628~pre-mRNA 3'-splice site binding,GO:0046872~metal ion binding,	IPR021715:Pre-mRNA splicing Prp18-interacting factor,	gga03040:Spliceosome,		
ENSGALG00000002906	TAR DNA binding protein(TARDBP)	GO:0001933~negative regulation of protein phosphorylation,GO:0006351~transcription, DNA-templated,GO:0006355~regulation of	GO:0005634~nucleus,GO:0005654~nucleoplasm,	GO:0000166~nucleotide binding,GO:0001205~transcriptional activator	IPR000504:RNA recognition motif domain,IPR012677:Nucleotide-binding, alpha-beta plait,			SM00360:RRM,

		transcription, DNA-templated,GO:0006397~mRNA processing,GO:0008380~RNA splicing,GO:0030264~nuclear fragmentation involved in apoptotic nuclear change,GO:0043922~negative regulation by host of viral transcription,GO:0051726~regulation of cell cycle,GO:0070935~3'-UTR-mediated mRNA stabilization,GO:0071765~nuclear inner membrane organization,		activity, RNA polymerase II distal enhancer sequence-specific binding,GO:0003676~nucleic acid binding,GO:0003677~DNA binding,GO:0003690~double-stranded DNA binding,GO:0003723~RNA binding,GO:0003730~mRNA 3'-UTR binding,				
ENSGALG00000015869	WW domain containing E3 ubiquitin protein ligase 1(WWP1)	GO:0042787~protein ubiquitination involved in ubiquitin-dependent protein catabolic process,GO:0043161~proteasome-mediated ubiquitin-dependent protein catabolic process,GO:0045892~negative regulation of transcription, DNA-templated,	GO:0005634~nucleus,GO:0005737~cytoplasm,GO:0070062~extracellular exosome,	GO:0004842~ubiquitin-protein transferase activity,GO:0016874~ligase activity,GO:0061630~ubiquitin protein ligase activity,	IPR000008:C2 calcium-dependent membrane targeting,IPR000569:HECT,IPR001202:WW domain,IPR024928:E3 ubiquitin-protein ligase, SMURF1 type,	gga04120:Ubiquitin mediated proteolysis,gga04144:Endocytosis,	PIRSF001569:E3 ubiquitin-protein ligase, SMURF1 type,	SM00119:HECTc, SM00239:C2, SM00456:WW,
ENSGALG00000009838	aquarius intron-binding spliceosomal	GO:0000398~mRNA splicing, via spliceosome,	GO:0016020~membrane,GO:0071013~catalytic step 2 spliceosome,	GO:0044822~poly(A) RNA binding,	IPR026300:CWF11 family,IPR027417:P-loop containing nucleoside triphosphate hydrolase,	gga03040:Spliceosome,	PIRSF038901:intron-binding spliceosome	

	factor(AQR)						component aquarius/cwf11,	
ENSGALG00000007012	arrestin domain containing 4(ARRDC4)	GO:0007165~signal transduction,GO:0051443~positive regulation of ubiquitin-protein transferase activity,	GO:0005768~endosome,GO:0005886~plasma membrane,		IPR011021:Arrestin-like, N-terminal,IPR011022:Arrestin C-terminal-like domain,IPR014756:Immunoglobulin E-set,			SM01017:SM01017,
ENSGALG00000012339	atlastin GTPase 1(ATL1)	GO:0007029~endoplasmic reticulum organization,GO:0051260~protein homooligomerization,	GO:0005783~endoplasmic reticulum,GO:0016021~integral component of membrane,	GO:0003924~GTPase activity,GO:0005525~GTP binding,	IPR003191:Guanylate-binding protein, C-terminal,IPR015894:Guanylate-binding protein, N-terminal,IPR027417:P-loop containing nucleoside triphosphate hydrolase,			
ENSGALG00000012579	bromodomain adjacent to zinc finger domain, 2B(BAZ2B)	GO:0006351~transcription, DNA-templated,GO:0006355~regulation of transcription, DNA-templated,	GO:0005634~nucleus,	GO:0003677~DNA binding,GO:0008270~zinc ion binding,	IPR001487:Bromodomain,IPR001739:Methyl-CpG DNA binding,IPR001965:Zinc finger, PHD-type,IPR011011:Zinc finger, FYVE/PHD-type,IPR013083:Zinc finger, RING/FYVE/PHD-type,IPR016024:Armadillo-type fold,IPR016177:DNA-binding, integrase-type,IPR018359:Bromodomain, conserved site,IPR018501:DDT domain superfamily,IPR019787:Zinc finger, PHD-finger,			SM00249:PHD, SM00297:BROMO, SM00391:MBD, SM00571:DDT,
ENSGALG00000002145	calmodulin regulated spectrin-associated	GO:0000226~microtubule cytoskeleton organization,GO:0031175~neuron projection development,GO:0033043~regulation of organelle organization,	GO:0005813~centrosome,		IPR001715:Calponin homology domain,IPR011033:PRC-barrel-like,IPR014797:CKK domain,IPR022613:			SM01051:SM01051,

	protein family, member 2(CAMSAP2)				Calmodulin-regulated spectrin-associated protein, CH domain,			
ENSGALG00000002095	charged multivesicular body protein 4B(CHMP4B)	GO:0000281~mitotic cytokinesis,GO:0000920~cell separation after cytokinesis,GO:0006620~posttranslational protein targeting to membrane,GO:0006914~autophagy,GO:0007034~vacuolar transport,GO:0007080~mitotic metaphase plate congression,GO:0010458~exit from mitosis,GO:0010824~regulation of centrosome duplication,GO:0031468~nuclear envelope reassembly,GO:0036438~maintenance of lens transparency,GO:0039702~viral budding via host ESCRT complex,GO:0051260~protein homooligomerization,GO:0090148~membrane fission,GO:0090611~ubiquitin-independent protein catabolic process via the multivesicular body sorting pathway,GO:0098609~cell-cell adhesion,GO:1901215~negative regulation of neuron death,GO:1901673~regulation of mitotic spindle	GO:0000815~ESCRT III complex,GO:0005635~nuclear envelope,GO:0005829~cytosol,GO:0005913~cell-cell adherens junction,GO:0009898~cytoplasmic side of plasma membrane,GO:0030117~membrane coat,GO:0030496~midbody,GO:0031902~late endosome membrane,GO:0070062~extracellular exosome,	GO:0042803~protein homodimerization activity,GO:0098641~cadherin binding involved in cell-cell adhesion,	IPR005024:Snf7,	gga04144:Endocytosis,		

		assembly,GO:1902188~positive regulation of viral release from host cell,GO:1902902~negative regulation of autophagosome assembly,						
ENSGALG00000009801	chromosome 5 open reading frame, human C15orf41(C5H15ORF41)							
ENSGALG00000014840	complement component 6(C6)	GO:0001970~positive regulation of activation of membrane attack complex,GO:0006955~immune response,GO:0045766~positive regulation of angiogenesis,	GO:0005579~membrane attack complex,GO:0070062~extracellular exosome,		IPR000436:Sushi/SCR/CCP,IPR000884:Thrombospondin, type 1 repeat,IPR001862:Membrane attack complex component/perforin/complement C9,IPR002172:Low-density lipoprotein (LDL) receptor class A repeat,IPR003884:Factor I / membrane attack complex,IPR020863:Membrane attack complex component/perforin domain, conserved site,IPR020864:Membrane attack complex component/perforin (MACPF) domain,IPR023415:Low-density			SM00032:CCP, SM00057:FIMAC, SM00192:LDLa, SM00209:TSP1,SM00457:MACPF,

					lipoprotein (LDL) receptor class A, conserved site,			
ENSGALG00000006050	cytochrome P450 family 2 subfamily R member 1(CYP2R1)			GO:0005506~iron ion binding,GO:0008395~steroid hydroxylase activity,GO:0016705~oxidoreductase activity, acting on paired donors, with incorporation or reduction of molecular oxygen,GO:0020037~heme binding,GO:0030343~vitamin D3 25-hydroxylase activity,	IPR001128:Cytochrome P450,IPR002401:Cytochrome P450, E-class, group I,IPR017972:Cytochrome P450, conserved site,	gga00100:Steroid biosynthesis,gga01100:Metabolic pathways,		
ENSGALG00000017366	dolichyl-phosphate mannosyltransferase polypeptide 1, catalytic subunit(DPM1)	GO:0006487~protein N-linked glycosylation,GO:0006506~GPI anchor biosynthetic process,GO:0019348~dolichol metabolic process,GO:0035269~protein O-linked mannosylation,	GO:0005634~nucleus,GO:0005789~endoplasmic reticulum membrane,GO:0033185~dolichol-phosphate-mannose synthase complex,	GO:0004169~dolichyl-phosphate-mannose-protein mannosyltransferase activity,GO:0004582~dolichyl-phosphate beta-D-mannosyltransferase activity,	IPR001173:Glycosyl transferase, family 2,	gga00510:N-Glycan biosynthesis,gga01100:Metabolic pathways,		

ENSGALG00000012916	drosha ribonuclease III(DROSHA)	GO:0006396~RNA processing,GO:0010586~miRNA metabolic process,GO:0010628~positive regulation of gene expression,GO:0016075~rRNA catabolic process,GO:0030422~production of siRNA involved in RNA interference,GO:0031053~primary miRNA processing,GO:0031054~pre-miRNA processing,GO:0045589~regulation of regulatory T cell differentiation,GO:0050727~regulation of inflammatory response,GO:0050829~defense response to Gram-negative bacterium,GO:0050830~defense response to Gram-positive bacterium,GO:0090502~RNA phosphodiester bond hydrolysis, endonucleolytic,GO:2000628~regulation of miRNA metabolic process,	GO:0005634~nucleus,GO:0070877~microprocessor complex,	GO:0001530~lipopolysaccharide binding,GO:0003723~RNA binding,GO:0003725~double-stranded RNA binding,GO:0004525~ribonuclease III activity,GO:0044822~poly(A) RNA binding,	IPR000999:Ribonuclease III domain,IPR011907:Ribonuclease III,IPR014720:Double-stranded RNA-binding-like domain,	gga03008:Ribosome biogenesis in eukaryotes,		SM00358:DSRM SM00535:RIBOc
ENSGALG00000014004	erythrocyte membrane protein band 4.1-like 2(EPB41L2)					gga04530:Tight junction,		

ENSGALG00000006622	fragile site, folic acid type, rare, fra candidate 1(FRA10AC1)				IPR019129:Folate-sensitive fragile site protein Fra10Ac1,			
ENSGALG00000013733	general transcription factor IIH subunit 5(GTF2H5)	GO:0006281~DNA repair,GO:0006289~nucleotide-excision repair,GO:0006294~nucleotide-excision repair, preincision complex assembly,GO:0006351~transcription, DNA-templated,GO:0006355~regulation of transcription, DNA-templated,GO:0006362~transcription elongation from RNA polymerase I promoter,GO:0006364~rRNA processing,GO:0071480~cellular response to gamma radiation,	GO:0000439~core TFIIH complex,GO:0005654~nucleoplasm,GO:0005730~nucleolus,	GO:0000182~rDNA binding,	IPR009400:TFIIH subunit TTDA/Tfb5,	gga03022:Basal transcription factors,gga03420:Nucleotide excision repair,		SM01395:SM01395,
ENSGALG00000009948	hedgehog interacting protein(HHIP)	GO:0005975~carbohydrate metabolic process,GO:0007224~smoothened signaling pathway,GO:0007405~neuroblast proliferation,GO:0009953~dorsal/ventral pattern formation,GO:0040036~regulation of fibroblast growth factor receptor signaling pathway,GO:0045879~negative regulation of	GO:0005887~integral component of plasma membrane,GO:0009986~cell surface,	GO:0008270~zinc ion binding,GO:0016901~oxidoreductase activity, acting on the CH-OH group of donors, quinone or similar compound as acceptor,GO	IPR000742:Epidermal growth factor-like domain,IPR011041:Soluble quinoprotein glucose/sorbosone dehydrogenase,IPR011042:Six-bladed beta-propeller, TolB-like,IPR012938:Glucose/Sorbosone dehydrogenase,IPR013032:EGF-like,	gga04340:Hedgehog signaling pathway,		SM00181:EGF,

		smoothened signaling pathway,GO:0048705~skeletal system morphogenesis,GO:0060441~epithelial tube branching involved in lung morphogenesis,		:0048038~quinone binding,GO:0097108~hedgehog family protein binding,	conserved site,IPR018143:Folate receptor-like,			
ENSGALG00000005451	helicase, lymphoid-specific(HELLS)	GO:0001655~urogenital system development,GO:0006306~DNA methylation,GO:0006346~methylation-dependent chromatin silencing,GO:0010216~maintenance of DNA methylation,GO:0031508~pericentric heterochromatin assembly,GO:0046651~lymphocyte proliferation,GO:2001243~negative regulation of intrinsic apoptotic signaling pathway,	GO:0005634~nucleus,GO:0005721~pericentric heterochromatin,	GO:0003682~chromatin binding,GO:0005524~ATP binding,	IPR000330:SNF2-related,IPR001650:Helicase, C-terminal,IPR014001:Helicase, superfamily 1/2, ATP-binding domain,IPR027417:P-loop containing nucleoside triphosphate hydrolase,			SM00487:DEXDc, SM00490:HELICc,
ENSGALG00000003947	heterogeneous nuclear ribonucleoprotein H3 (2H9)(HNRNPH3)	GO:0030855~epithelial cell differentiation,	GO:0005654~nucleoplasm,	GO:0000166~nucleotide binding,GO:0044822~poly(A) RNA binding,	IPR000504:RNA recognition motif domain,IPR012677:Nucleotide-binding, alpha-beta plait,			SM00360:RRM,
ENSGALG00000014991	histone deacetylase 2(HDAC2)	GO:0000122~negative regulation of transcription from RNA polymerase II promoter,GO:0006325~chromatin organization,GO:0006344~maintenance of chromatin	GO:0000118~histone deacetylase complex,GO:0005634~nucleus,GO:0005737~cytoplasm,GO:0016580~Sin3 complex,GO:0016581~NuRD complex,GO:0035098~ESC/E(Z) complex,	GO:0000978~RNA polymerase II core promoter proximal region sequence-specific	IPR000286:Histone deacetylase superfamily,IPR003084:Histone deacetylase,IPR023801:Histone deacetylase domain,	gga04110:Cell cycle,gga04330:Notch signaling pathway,	PIRSF037913:histone deacetylase class I, eukaryotic type,	

		silencing,GO:0006351~transcription, DNA-templated,GO:0006355~regulation of transcription, DNA-templated,GO:0008284~positive regulation of cell proliferation,GO:0010870~positive regulation of receptor biosynthetic process,GO:0043044~ATP-dependent chromatin remodeling,GO:0043433~negative regulation of sequence-specific DNA binding transcription factor activity,GO:0045862~positive regulation of proteolysis,GO:0045944~positive regulation of transcription from RNA polymerase II promoter,GO:0070932~histone H3 deacetylation,		DNA binding,GO:0000980~RNA polymerase II distal enhancer sequence-specific DNA binding,GO:0004407~histone deacetylase activity,GO:0008134~transcription factor binding,GO:0032041~NAD-dependent histone deacetylase activity (H3-K14 specific),GO:0044822~poly(A) RNA binding,				
ENSGALG00000002199	kelch like family member 17(KLHL17)	GO:0007420~brain development,GO:0016567~protein ubiquitination,GO:0030036~actin cytoskeleton organization,	GO:0005615~extracellular space,GO:0015629~actin cytoskeleton,GO:0031463~Cul3-RING ubiquitin ligase complex,GO:0032839~dendrite cytoplasm,	GO:0051015~actin filament binding,	IPR000210:BTB/POZ-like,IPR006652:Kelch repeat type 1,IPR011043:Galactose oxidase/kelch, beta-propeller,IPR011333:BTB/POZ fold,IPR011705:BTB/Kelch-associated,IPR015916:Galactose oxidase, beta-propeller,IPR01709		PIRSF037037: kelch-like protein, gigaxonin type,	SM00225:BTB, SM00612:Kelch, SM00875:SM00875,

					6:Kelch-like protein, gigaxonin,			
ENSGALG00000008871	latrophilin 2(LPHN2)	GO:0007166~cell surface receptor signaling pathway,GO:0060317~cardiac epithelial to mesenchymal transition,	GO:0016021~integral component of membrane,	GO:0004930~G-protein coupled receptor activity,GO:0030246~carbohydrate binding,	IPR000203:GPS domain,IPR000832:GPCR, family 2, secretin-like,IPR000922:D-galactoside/L-rhamnose binding SUEL lectin domain,IPR001879:GPCR, family 2, extracellular hormone receptor domain,IPR003112:Olfactomedin-like,IPR003334:GPCR, family 2, latrophilin, C-terminal,IPR003924:GPCR, family 2, latrophilin,IPR017981:GPCR, family 2-like,IPR017983:GPCR, family 2, secretin-like, conserved site,			SM00008:HormR, SM00284:OLF, SM00303:GPS,
ENSGALG00000005284	leucine rich repeat containing				IPR001611:Leucine-rich repeat,IPR003591:Leucine-rich repeat, typical subtype,			SM00369:LRR_TYP,

	39(LRRC39)							
ENSGALG00000001852	leucine-rich repeats and WD repeat domain containing 1(LRWD1)							
ENSGALG00000002288	ligase III, DNA, ATP-dependent(LIG3)	GO:0006260~DNA replication,GO:0006288~base-excision repair, DNA ligation,GO:0006302~double-strand break repair,GO:0006310~DNA recombination,GO:0051103~DNA ligation involved in DNA repair,GO:0071897~DNA biosynthetic process,GO:0090298~negative regulation of mitochondrial DNA replication,	GO:0005634~nucleus,GO:0005739~mitochondrion,	GO:0003677~DNA binding,GO:0003910~DNA ligase (ATP) activity,GO:0005524~ATP binding,GO:0008270~zinc ion binding,	IPR000977:DNA ligase, ATP-dependent,IPR001357:BRCT domain,IPR001510:Zinc finger, PARP-type,IPR012308:DNA ligase, ATP-dependent, N-terminal,IPR012309:DNA ligase, ATP-dependent, C-terminal,IPR012310:DNA ligase, ATP-dependent, central,IPR012340:Nucleic acid-binding, OB-fold,IPR016059:DNA ligase, ATP-dependent, conserved site,	gga03410:Base excision repair,		SM00292:BRCT,SM01336:SM01336,
ENSGALG00000016487	lipid droplet associated hydrolase(LDAH)	GO:0019915~lipid storage,	GO:0005783~endoplasmic reticulum,GO:0005811~lipid particle,	GO:0016298~lipase activity,	IPR019363:Protein of unknown function DUF2305,			
ENSGALG00000005226	myozenin	GO:0030239~myofibril assembly,	GO:0015629~actin cytoskeleton,GO:0030018~Z disc,	GO:0003779~actin binding,GO:	IPR008438:Calcineurin-binding,			

	1(MYOZ1)			0031433~telethonin binding,GO:0051373~FATZ binding,				
ENSGALG00000007000	nuclear receptor subfamily 2, group F, member 2(NR2F2)	GO:0000122~negative regulation of transcription from RNA polymerase II promoter,GO:0001764~neuron migration,GO:0001937~negative regulation of endothelial cell proliferation,GO:0006351~transcription, DNA-templated,GO:0007519~skeletal muscle tissue development,GO:0009566~fertilization,GO:0009952~anterior/posterior pattern specification,GO:0009956~radial pattern formation,GO:0010596~negative regulation of endothelial cell migration,GO:0030522~intracellular receptor signaling pathway,GO:0030900~forebrain development,GO:0043401~steroid hormone mediated signaling pathway,GO:0045736~negative regulation of cyclin-dependent protein serine/threonine kinase activity,GO:0045893~positive regulation of transcription, DNA-templated,GO:0048514	GO:0005634~nucleus,	GO:0001972~retinoic acid binding,GO:0003707~steroid hormone receptor activity,GO:0004879~RNA polymerase II transcription factor activity, ligand-activated sequence-specific DNA binding,GO:0008270~zinc ion binding,GO:0042803~protein homodimerization activity,GO:0043565~sequence-specific DNA binding,	IPR000536:Nuclear hormone receptor, ligand-binding, core,IPR001628:Zinc finger, nuclear hormone receptor-type,IPR001723:Steroid hormone receptor,IPR003068:Transcription factor COUP,IPR013088:Zinc finger, NHR/GATA-type,			SM00399:ZnF_C4, SM00430:HOLI,

		~blood vessel morphogenesis,GO:0060173~limb development,GO:0060849~regulation of transcription involved in lymphatic endothelial cell fate commitment,						
ENSGALG00000005078	nucleoporin 210kDa(NUP210)		GO:0005643~nuclear pore,GO:0016021~integral component of membrane,		IPR003343:Bacterial Ig-like, group 2,IPR008964:Invasin/intimin cell-adhesion,	gga03013:RNA transport,		SM00635:BID_2,
ENSGALG00000015086	oxysterol binding protein-like 1A(OSBPL1A)	GO:0006869~lipid transport,	GO:0005654~nucleoplasm,GO:0005770~late endosome,GO:0070062~extracellular exosome,		IPR000648:Oxysterol-binding protein,IPR001849:Pleckstrin homology domain,IPR002110:Ankyrin repeat,IPR011993:Pleckstrin homology-like domain,IPR018494:Oxysterol-binding protein, conserved site,IPR020683:Ankyrin repeat-containing domain,			SM00233:PH, SM00248:ANK,
ENSGALG00000012754	phenylalanine hydroxylase(PAH)	GO:0006559~L-phenylalanine catabolic process,GO:0009072~aromatic amino acid family metabolic process,	GO:0070062~extracellular exosome,	GO:0004505~phenylalanine 4-monooxygenase activity,GO:0005506~iron ion binding,GO:0016597~amino acid binding,	IPR001273:Aromatic amino acid hydroxylase,IPR002912:ACT domain,IPR005961:Phenylalanine-4-hydroxylase, tetrameric form,IPR018301:Aromatic amino acid hydroxylase, iron/copper binding site,IPR019773:Tyrosine 3-monooxygenase-	gga00360:Phenylalanine metabolism,gga00400:Phenylalanine, tyrosine and tryptophan biosynthesis,gga01100:Metabolic pathways,gga01230:Biosynthesis of amino acids,	PIRSF000336:tyrosine 3-monooxygenase,	

					like,IPR019774:Aromatic amino acid hydroxylase, C-terminal,			
ENSGALG00000007326	phosphomannomutase 2(PMM2)	GO:0009298~GDP-mannose biosynthetic process,	GO:0005737~cytoplasm,GO:0043025~neuronal cell body,GO:0070062~extracellular exosome,	GO:0004615~phosphomannomutase activity,	IPR005002:Eukaryotic phosphomannomutase,IPR006379:HAD-superfamily hydrolase, subfamily IIB,IPR023214:HAD-like domain,	gga00051:Fructose and mannose metabolism,gga00520:Amino sugar and nucleotide sugar metabolism,gga01100:Metabolic pathways,		
ENSGALG00000002647	plastin 1(PLS1)	GO:0001951~intestinal D-glucose absorption,GO:0032532~regulation of microvillus length,GO:0040018~positive regulation of multicellular organism growth,GO:0051017~actin filament bundle assembly,GO:0051639~actin filament network formation,GO:0051764~actin crosslink formation,GO:0090004~positive regulation of establishment of protein localization to plasma membrane,GO:1902896~terminal web assembly,	GO:0005737~cytoplasm,GO:0005884~actin filament,GO:0005903~brush border,GO:0032432~actin filament bundle,GO:0070062~extracellular exosome,GO:1990357~terminal web,	GO:0003779~actin binding,GO:0005509~calcium ion binding,GO:0051015~actin filament binding,	IPR001589:Actinin-type, actin-binding, conserved site,IPR001715:Calponin homology domain,IPR002048:EF-hand domain,IPR011992:EF-hand-like domain,IPR018247:EF-Hand 1, calcium-binding site,			SM00033:CH, SM00054:EFh,
ENSGALG00000002618	potassium channel tetramerization domain containing	GO:0051260~protein homooligomerization,	GO:0005829~cytosol,GO:0005886~plasma membrane,		IPR000210:BTB/POZ-like,IPR003131:Potassium channel tetramerisation-type BTB domain,IPR011333:BTB/POZ fold,			SM00225:BTB,

	7(KCTD 7)								
ENSGALG00000006783	procollagen-lysine, 2-oxoglutarate 5-dioxygenase 2(PLOD2)	GO:0001666~response to hypoxia,GO:0098779~mitophagy in response to mitochondrial depolarization,	GO:0005783~endoplasmic reticulum,GO:0070062~extracellular exosome,	GO:0005506~iron ion binding,GO:0008475~procollagen-lysine 5-dioxygenase activity,GO:0031418~L-ascorbic acid binding,	IPR001006:Procollagen-lysine 5-dioxygenase,IPR005123:Oxoglutarate/iron-dependent dioxygenase,IPR006620:Prolyl 4-hydroxylase, alpha subunit,	gga00310:Lysine degradation,		SM00702:P4Hc,
ENSGALG00000010950	protein phosphatase 4, regulatory subunit 4(PPP4R4)	GO:0032515~negative regulation of phosphoprotein phosphatase activity,GO:0080163~regulation of protein serine/threonine phosphatase activity,	GO:0005737~cytoplasm,GO:0008287~protein serine/threonine phosphatase complex,	GO:0019888~protein phosphatase regulator activity,	IPR011989:Armadillo-like helical,IPR016024:Armadillo-type fold,IPR021133:HEAT, type 2,			
ENSGALG00000025941	regulator of G-protein signaling 20(RGS20)	GO:0006355~regulation of transcription, DNA-templated,GO:0008219~cell death,GO:0009950~dorsal/ventral axis specification,GO:0030154~cell differentiation,GO:0035412~regulation of catenin import into nucleus,GO:0043547~positive regulation of GTPase activity,GO:0071407~cellular response to organic cyclic compound,GO:0090090~negative regulation of canonical Wnt signaling pathway,GO:0090244~Wnt signaling pathway	GO:0005634~nucleus,GO:0005802~trans-Golgi network,GO:0005881~cytoplasmic microtubule,GO:0005886~plasma membrane,GO:0005938~cell cortex,GO:0014069~postsynaptic density,GO:0016023~cytoplasmic, membrane-bounded vesicle,GO:0030877~beta-catenin destruction complex,	GO:0005096~GTPase activator activity,GO:0008013~beta-catenin binding,GO:0019901~protein kinase binding,GO:0070016~armadillo repeat domain binding,	IPR016137:Regulator of G protein signalling superfamily,IPR024066:Regulator of G-protein signaling, domain 1,			SM00315:RGS,

		involved in somitogenesis,						
ENSGALG00000012626	reversion inducing cysteine rich protein with kazal motifs(RECK)	GO:0001955~blood vessel maturation,GO:0010951~negative regulation of endopeptidase activity,GO:0030198~extracellular matrix organization,GO:0030336~negative regulation of cell migration,GO:0035115~embryonic forelimb morphogenesis,GO:0045665~negative regulation of neuron differentiation,GO:0045747~positive regulation of Notch signaling pathway,GO:1904684~negative regulation of metalloendopeptidase activity,	GO:0016020~membrane,	GO:0008191~metalloendopeptidase inhibitor activity,	IPR002350:Kazal domain,			SM00280:KAZAL,
ENSGALG00000000523	serine/threonine kinase 38(STK38)	GO:0018105~peptidyl-serine phosphorylation,GO:0035556~intracellular signal transduction,GO:0043407~negative regulation of MAP kinase activity,	GO:0005622~intracellular,GO:0005737~cytoplasm,GO:0070688~MLL5-L complex,	GO:0000287~magnesium ion binding,GO:0004674~protein serine/threonine kinase activity,GO:0005524~ATP binding,	IPR000719:Protein kinase, catalytic domain,IPR000961:AGC-kinase, C-terminal,IPR008271:Serine/threonine-protein kinase, active site,IPR011009:Protein kinase-like domain,IPR017441:Protein kinase, ATP binding site,IPR017892:Protein kinase, C-terminal,			SM00133:S_TK_X, SM00220:S_TKc,
ENSGALG00000006049	serine/threonine protein kinase	GO:0030033~microvillus assembly,GO:0042542~response to hydrogen	GO:0005634~nucleus,GO:0005737~cytoplasm,GO:0005798~Golgi-associated vesicle,GO:0005813~centrosome,	GO:0000287~magnesium ion binding,GO:	IPR000719:Protein kinase, catalytic domain,IPR011009:Protein kinase-like			SM00220:S_TKc,

	MST4(MST4)	peroxide,GO:0042981~regulation of apoptotic process,GO:0046777~protein autophosphorylation,GO:1903205~regulation of hydrogen peroxide-induced cell death,	GO:0005829~cytosol,GO:0016324~apical plasma membrane,GO:0048471~perinuclear region of cytoplasm,GO:0070062~extracellular exosome,	0004672~protein kinase activity,GO:0004702~receptor signaling protein serine/threonine kinase activity,GO:0005524~ATP binding,	domain,IPR017441:Protein kinase, ATP binding site,				
ENSGALG00000009350	sideroflexin 4(SFXN4)		GO:0005739~mitochondrion,GO:0016021~integral component of membrane,	GO:0015075~ion transmembrane transporter activity,	IPR004686:Tricarboxylate/iron carrier,				
ENSGALG00000014971	solute carrier family 2 (facilitated glucose transporter), member 9(SLC2A9)	GO:0046415~urate metabolic process,	GO:0005886~plasma membrane,GO:0016021~integral component of membrane,	GO:0005355~glucose transmembrane transporter activity,GO:0015143~urate transmembrane transporter activity,	IPR003663:Sugar/inositol transporter,IPR005828:General substrate transporter,IPR005829:Sugar transporter, conserved site,IPR020846:Major facilitator superfamily domain,				
ENSGALG00000008222	solute carrier family 26 (anion exchanger), member 5(SLC26A5)	GO:0007605~sensory perception of sound,GO:0015701~bicarbonate transport,GO:0042391~regulation of membrane potential,GO:0051453~regulation of intracellular pH,GO:1902476~chloride transmembrane transport,	GO:0005887~integral component of plasma membrane,GO:0016323~basolateral plasma membrane,GO:0016328~lateral plasma membrane,	GO:0005254~chloride channel activity,GO:0008271~secondary active sulfate transmembrane transporter activity,GO:0015106~bi	IPR001902:Sulphate anion transporter,IPR002645:STAS domain,IPR011547:Sulphate transporter,IPR018045:Sulphate anion transporter, conserved site,				

				carbonate transmembrane transporter activity,GO:0015116~sulfate transmembrane transporter activity,GO:0015301~anion:anion antiporter activity,GO:0019531~oxalate transmembrane transporter activity,				
ENSGALG00000013920	suppressor of variegation 3-9 homolog 2 (Drosophila)(SUV39H2)	GO:0000122~negative regulation of transcription from RNA polymerase II promoter,GO:0006333~chromatin assembly or disassembly,GO:0006338~chromatin remodeling,GO:0006351~transcription, DNA-templated,GO:0007049~cell cycle,GO:0030154~cell differentiation,GO:0051567~histone H3-K9 methylation,GO:0071456~cellular response to hypoxia,	GO:0000775~chromosome, centromeric region,GO:0000785~chromatin,GO:0005634~nucleus,	GO:0008270~zinc ion binding,GO:0046974~histone methyltransferase activity (H3-K9 specific),	IPR000953:Chromo domain/shadow,IPR001214:SET domain,IPR003616:Post-SET domain,IPR007728:Pre-SET domain,IPR011381:Histone H3-K9 methyltransferase,IPR016197:Chromo domain-like,IPR023779:Chromo domain, conserved site,IPR023780:Chromo domain,	gga00310:Lysine degradation,	PIRSF009343:histone H3-K9 methyltransferase,	SM00298:CHROMO, SM00317:SET, SM00468:PreSET,
ENSGALG00000002930	syntaxin 7(STX7)	GO:0001916~positive regulation of T cell mediated cytotoxicity,GO:0006886~intracellular protein transport,GO:0006906~	GO:0001772~immunological synapse,GO:0005765~lysosomal membrane,GO:0005769~early endosome,GO:0005770~late endosome,GO:0012505~endomembrane	GO:0000149~SNARE binding,GO:0005484~SNAP receptor	IPR000727:Target SNARE coiled-coil domain,IPR006011:Syntaxin, N-terminal,IPR006012:Syntaxin/epimorp	gga04130:SNARE interactions in vesicular transport,gga0		SM00397:t_SNARE, SM00503:SynN,

		vesicle fusion,GO:0048278~vesicle docking,GO:0051640~organelle localization,GO:0070925~organelle assembly,GO:1902685~positive regulation of receptor localization to synapse,GO:1903076~regulation of protein localization to plasma membrane,	system,GO:0016021~integral component of membrane,GO:0030139~endocytic vesicle,GO:0031201~SNARE complex,GO:0042582~azurophil granule,GO:0048471~perinuclear region of cytoplasm,GO:0055037~recycling endosome,GO:0070062~extracellular exosome,GO:0070820~tertiary granule,	activity,GO:0019869~chloride channel inhibitor activity,	hin, conserved site,IPR010989:t-SNARE,	4145:Phagosome,		
ENSGALG00000003849	transient receptor potential cation channel, subfamily M, member 1(TRPM1)	GO:0007216~G-protein coupled glutamate receptor signaling pathway,GO:0007601~visual perception,GO:0046548~retinal rod cell development,GO:0051262~protein tetramerization,GO:0060402~calcium ion transport into cytosol,GO:0071482~cellular response to light stimulus,	GO:0016021~integral component of membrane,GO:0035841~new growing cell tip,	GO:0005262~calcium channel activity,	IPR005821:Ion transport domain,			
ENSGALG00000015014	transportin 1(TNPO1)							
ENSGALG00000016936	tudor domain containing 3(TDRD3)	GO:0006397~mRNA processing,GO:0016569~covalent chromatin modification,GO:1903506~regulation of nucleic acid-templated transcription,	GO:0005634~nucleus,GO:0005737~cytoplasm,GO:0035145~exon-exon junction complex,	GO:0003682~chromatin binding,GO:0003713~transcription coactivator activity,GO:0003723~RNA binding,GO:0035064~m	IPR002999:Tudor domain,IPR009060:UBA-like,IPR010304:Survival motor neuron,IPR013894:Domain of unknown function DUF1767,IPR015940:Ubiquitin-associated/translatio			SM00165:UBA,SM00333:TUDOR,SM01161:SM01161,

				ethylated histone binding,GO:0044822~poly(A) RNA binding,	n elongation factor EF1B, N-terminal, eukaryote,			
ENSGALG00000019300	tyrosine hydroxylase-like(THL)	GO:0009072~aromatic amino acid family metabolic process,		GO:0005506~iron ion binding,GO:0016714~oxidoreductase activity, acting on paired donors, with incorporation or reduction of molecular oxygen, reduced pteridine as one donor, and incorporation of one atom of oxygen,	IPR001273:Aromatic amino acid hydroxylase,IPR018301:Aromatic amino acid hydroxylase, iron/copper binding site,IPR019773:Tyrosine 3-monooxygenase-like,IPR019774:Aromatic amino acid hydroxylase, C-terminal,	gga00350:Tyrosine metabolism,gga01100:Metabolic pathways,	PIRSF000336:tyrosine 3-monooxygenase,	
ENSGALG00000008015	ubiquitin-conjugating enzyme E2 variant 1(UBE2V1)	GO:0006301~postreplication repair,GO:0043123~positive regulation of I-kappaB kinase/NF-kappaB signaling,GO:0051092~positive regulation of NF-kappaB transcription factor activity,GO:0070534~protein K63-linked ubiquitination,	GO:0000151~ubiquitin ligase complex,GO:0005634~nucleus,GO:0005737~cytoplasm,GO:0035370~UBC13-UEV1A complex,GO:0070062~extracellular exosome,	GO:0031625~ubiquitin protein ligase binding,GO:0061630~ubiquitin protein ligase activity,	IPR000608:Ubiquitin-conjugating enzyme, E2,IPR016135:Ubiquitin-conjugating enzyme/RWD-like,			
ENSGALG00000001986	valosin containing			GO:0005524~ATP binding,GO:	IPR003338:CDC48, N-terminal subdomain,IPR003	gga04141:Protein processing in		SM00382:AAA,SM01072:SM01072,

	protein(VCP)			0016787~hydrolase activity,	593:AAA+ ATPase domain,IPR003959:ATPase, AAA-type, core,IPR003960:ATPase, AAA-type, conserved site,IPR004201:CDC48, domain 2,IPR005938:ATPase, AAA-type, CDC48,IPR009010:Aspartate decarboxylase-like domain,IPR015415:Vps4 oligomerisation, C-terminal,IPR027417:P-loop containing nucleoside triphosphate hydrolase,	endoplasmic reticulum,		SM01073:SM01073,
ENSGALG00000006025	xin actin-binding repeat containing 1(XIRP1)	GO:0007507~heart development,GO:0030036~actin cytoskeleton organization,	GO:0030054~cell junction,	GO:0003779~actin binding,	IPR012510:Actin-binding, Xin repeat,			
ENSGALG00000013911	zinc finger CCCH-type, antiviral 1(ZC3HAV1)	GO:0009615~response to virus,GO:0045071~negative regulation of viral genome replication,GO:0061014~positive regulation of mRNA catabolic process,	GO:0005794~Golgi apparatus,	GO:0003950~NAD+ ADP-ribosyltransferase activity,GO:0044822~poly(A) RNA binding,GO:0046872~metal ion binding,	IPR000571:Zinc finger, CCCH-type,IPR004170:WWE domain,IPR012317:Poly(ADP-ribose) polymerase, catalytic domain,			

ENSGALG00000007260	zinc finger E-box binding homeobox 1(ZEB1)	GO:0006351~transcription, DNA-templated,GO:0007389~pattern specification process,GO:0007417~central nervous system development,GO:0008285~negative regulation of cell proliferation,GO:0010464~regulation of mesenchymal cell proliferation,GO:0017015~regulation of transforming growth factor beta receptor signaling pathway,GO:0030857~negative regulation of epithelial cell differentiation,GO:0033081~regulation of T cell differentiation in thymus,GO:0045666~positive regulation of neuron differentiation,GO:0045892~negative regulation of transcription, DNA-templated,GO:0045944~positive regulation of transcription from RNA polymerase II promoter,GO:0048596~embryonic camera-type eye morphogenesis,GO:0048704~embryonic skeletal system morphogenesis,GO:0048752~semicircular canal morphogenesis,GO:0051150~regulation of smooth muscle cell differentiation,GO:0051	GO:0005634~nucleus,GO:0005654~nucleoplasm,GO:0005667~transcription factor complex,GO:0005737~cytoplasm,	GO:0001227~transcriptional repressor activity, RNA polymerase II transcription regulatory region sequence-specific binding,GO:0003682~chromatin binding,GO:0046872~metal ion binding,GO:0070888~E-box binding,	IPR001356:Homeodomain,IPR007087:Zinc finger, C2H2,IPR008598:Drought induced 19/ RING finger protein 114,IPR009057:Homeodomain-like,IPR013087:Zinc finger C2H2-type/integrase DNA-binding domain,IPR015880:Zinc finger, C2H2-like,			SM00355:ZnF_C2H2, SM00389:HOX,

		216~cartilage development,GO:0071230~cellular response to amino acid stimulus,GO:0090103~cochlea morphogenesis,						

Supplementary Table 21: eQTls identified in Liver of low and high FCR broilers

chr	ref	alt	pos	snp_id	NegLog10_p_Value	Gene	Location
3	A	G	57057905	rs740509564	1.52414017	EPB41L2	Non-Synonymus
5	T	C	36457370	rs312273451	1.319906838	AQR	Downstream
1	C	T	7316181	rs794365618	1.014165052	SUV39H2	Upstream
3	C	T	57018841	rs794037360	0.982117511	EPB41L2	Synonymus
3	C	G	57080251	_Variant_38	0.951334318	EPB41L2	Downstream
3	T	C	57080455	_Variant_40	0.951334318	EPB41L2	Downstream
5	A	C	36457992	rs738541835	0.951334318	AQR	Downstream
5	A	G	36457556	rs735644237	0.880787805	AQR	Downstream
6	C	T	16040458	rs314003784	0.880787805	HELLS	3'UTR
3	A	T	57082502	rs731934309	0.85496093	EPB41L2	Downstream
5	C	T	36457591	rs734864315	0.85496093	AQR	Downstream
3	A	G	57079945	rs10728941	0.847059963	EPB41L2	Downstream
3	T	C	57079997	_Variant_37	0.847059963	EPB41L2	Downstream
3	T	G	57080337	rs10722790	0.847059963	EPB41L2	Downstream
3	A	T	57080616	_Variant_41	0.847059963	EPB41L2	Downstream
3	A	G	57080777	_Variant_44	0.847059963	EPB41L2	Downstream
5	T	A	36457914	rs315807977	0.819604589	AQR	Downstream
22	C	T	543304	_Variant_26	0.762067084	BNIP3L	Upstream
3	G	C	57084194	_Variant_48	0.755143376	EPB41L2	Downstream
3	A	T	57084200	_Variant_49	0.755143376	EPB41L2	Downstream
15	A	C	8913126	rs735962820	0.735503077	ENSGALG00000028473	Downstream
15	T	A	8913139	rs312382045	0.735503077	ENSGALG00000028473	Downstream
1	A	G	7327024	rs794333485	0.722330588	SUV39H2	Downstream
5	T	C	31528251	rs731332974	0.722330588	AQR	Downstream
5	A	G	36458017	rs314995363	0.717186237	AQR	Downstream
1	G	C	7316337	rs29006922	0.686336881	SUV39H2	Upstream
5	A	G	36457599	rs317142810	0.686336881	AQR	Downstream
3	G	A	57082356	rs793979234	0.644372624	EPB41L2	Downstream
5	G	A	36457747	rs315614166	0.601230181	AQR	Downstream
5	C	A	36455062	rs313175159	0.581379566	AQR	Downstream
1	G	C	7316614	rs80635205	0.580256582	SUV39H2	Upstream
3	G	A	57017397	rs731395648	0.569618263	EPB41L2	Synonymus
5	G	A	36457534	rs316895964	0.569618263	AQR	Downstream
3	A	G	57075739	rs735415283	0.567555116	EPB41L2	3'UTR
5	T	A	36457980	rs316541435	0.567555116	AQR	Downstream
6	T	C	16040484	rs312828908	0.567555116	HELLS	3'UTR
5	T	A	36457594	_Variant_89	0.512841964	AQR	Downstream
5	A	G	36457082	rs738663853	0.510728528	AQR	Downstream
5	T	C	36457100	rs734621078	0.510728528	AQR	Downstream

22	A	C	526673	_Variant_23	0.462067031	BNIP3L	Gene
22	G	A	526684	_Variant_24	0.462067031	BNIP3L	Gene
3	A	G	57032684	rs317722900	0.461201906	EPB41L2	Synonymus
5	G	A	36457465	rs312924875	0.461201906	AQR	Downstream
1	A	T	7325196	_Variant_13	0.455780924	SUV39H2	Gene
1	C	T	7325198	_Variant_14	0.455780924	SUV39H2	Gene
1	C	T	7316510	rs739382491	0.411218509	SUV39H2	Upstream
3	T	G	64247348	rs740416216	0.411218509	HDAC2	Synonymus
6	T	C	16040401	rs10725765	0.411218509	HELLS	3'UTR
6	C	G	16040410	rs731961573	0.411218509	HELLS	3'UTR
5	A	G	36457101	rs737996131	0.394928119	AQR	Downstream
3	A	G	57080755	rs314335830	0.35163148	EPB41L2	Downstream
1	T	A	7320431	_Variant_11	0.348141634	SUV39H2	Gene
3	A	T	64254585	rs315665735	0.315049155	HDAC2	Synonymus
3	T	C	64260200	rs10724667	0.315049155	HDAC2	Synonymus
1	C	T	7316525	rs29006925	0.310217403	SUV39H2	Upstream
5	T	C	36457337	rs315890893	0.310217403	AQR	Downstream
5	A	G	31472112	rs14531077	0.305317452	AQR	Upstream
1	G	A	7325010	rs315815511	0.269949717	SUV39H2	Non-synonymus
1	T	C	7327619	rs312365413	0.210963789	SUV39H2	Downstream
22	T	C	524233	rs317485010	0.206415541	BNIP3L	3'UTR
3	A	G	64261137	rs735340215	0.178800624	HDAC2	Non-synonymus
5	G	A	36458101	rs317831540	0.178800624	AQR	Downstream
3	A	G	64251242	rs314845742	0.163844424	HDAC2	Synonymus
3	G	A	57048005	rs314670358	0.157456394	EPB41L2	Synonymus
3	G	A	57069966	rs80638423	0.155652693	EPB41L2	Synonymus
22	T	G	543167	rs317599959	0.119906068	BNIP3L	Upstream
1	T	C	7316182	rs741084926	0.107620563	SUV39H2	Upstream
5	C	A	36454988	rs739223212	0.107620563	AQR	Downstream
5	T	A	36454993	rs733085732	0.107620563	AQR	Downstream
5	T	C	36456886	rs316733829	0.107620563	AQR	Downstream
5	C	T	36457417	rs735777576	0.100662774	AQR	Downstream
5	A	G	36454997	rs312734714	0.100636158	AQR	Downstream
5	T	C	36455032	rs740342570	0.100636158	AQR	Downstream
5	T	C	36455033	rs314592654	0.100636158	AQR	Downstream
1	G	A	7316355	rs29006923	0.081236683	SUV39H2	Upstream
1	A	C	7316408	rs29006924	0.081236683	SUV39H2	Upstream
1	C	T	7316602	rs80764606	0.081236683	SUV39H2	Upstream
1	G	A	7316606	rs80583605	0.081236683	SUV39H2	Upstream
5	T	A	31528035	rs316486647	0.081236683	AQR	Synonymus
5	G	C	31528092	rs740254096	0.081236683	AQR	Downstream

5	A	G	31528154	rs316872746	0.081236683	AQR	Downstream
5	T	C	31528524	rs732994297	0.075700455	AQR	Downstream
22	T	A	525347	rs313597604	0.062089336	BNIP3L	3'UTR
5	A	T	31527968	rs737985384	0.062089336	AQR	Non-synonymus
5	T	G	36457330	rs313179586	0.062089336	AQR	Downstream
6	G	A	16031958	rs10721832	0.061851596	HELLS	Downstream
5	C	A	31529767	rs15696264	0.042614301	AQR	Downstream
6	A	G	16040316	rs10728037	0.042614301	HELLS	3'UTR
3	G	A	64254594	rs315662618	0.041157534	HDAC2	Synonymus
5	T	G	31472173	rs732930542	0.041157534	AQR	Upstream
5	A	C	31528028	rs731962992	0.041157534	AQR	Non-synonymus
5	G	A	36457959	rs315385125	0.041157534	AQR	Downstream
6	G	C	16036809	rs733543535	0.041157534	HELLS	Downstream
6	G	A	16047197	rs318092024	0.027107458	HELLS	Synonymus
22	T	G	524943	_Variant_21	0	BNIP3L	Gene
3	G	A	57079872	rs313579491	0	EPB41L2	Downstream
5	T	C	36457068	rs733834901	0	AQR	Downstream
5	A	G	36457069	rs794183374	0	AQR	Downstream
5	G	A	36457107	rs315130906	0	AQR	Downstream
6	A	C	16037035	rs317695693	0	HELLS	Downstream
15	A	T	8910269	rs315463844		ENSGALG00000028473	Downstream
3	A	G	57051453	rs317619687		EPB41L2	Synonymus
3	G	A	57080674	rs318146089		EPB41L2	Downstream
3	G	T	57080792	rs312723950		EPB41L2	Downstream
3	T	C	64261646	rs315656978		HDAC2	Downstream
5	T	C	31528733	rs316631116		AQR	Downstream
6	T	C	16031901	rs317974139		HELLS	Downstream
6	C	T	16037021	rs16547514		HELLS	Downstream
6	T	A	16040300	rs16547546		HELLS	3'UTR

Supplementary Table 22: Significant eQTLs identified in Liver of low and high FCR broilers

chr	ref	alt	pos	snp_id	NegLog10 p_Value	Gene	Location	Log2foldchange (low vs high)	p-value for Log2foldchange (low vs high)
3	A	G	57057905	rs740509564	1.52414017	EPB41L2	Non-Synonymus	4.037453971	0.006393318
5	T	C	36457370	rs312273451	1.319906838	AQR	Downstream	4.804064071	0.004148322

Supplementary Table 23: Enriched QTL traits from the significant eQTLs of Duodenum of low and high FCR broilers

chr	pos	snp_id	Gene	QTL_id
3	31343215	rs316268789	EIF2AK2	Abdominal fat percentage QTL (9417)
3	31343215	rs316268789	EIF2AK2	Abdominal fat weight QTL (9418)
3	31343215	rs316268789	EIF2AK2	Breast muscle percentage QTL (9416)
3	31343215	rs316268789	EIF2AK2	Body weight QTL (1951)
3	31343215	rs316268789	EIF2AK2	Tibia bone mineral density QTL (1950)
3	31343215	rs316268789	EIF2AK2	Head percentage QTL (15598)
3	31343215	rs316268789	EIF2AK2	Body weight (1 day) QTL (7167)
3	31343215	rs316268789	EIF2AK2	Body weight (35 days) QTL (7171)
3	31343215	rs316268789	EIF2AK2	Body weight (41 days) QTL (7174)
3	31343215	rs316268789	EIF2AK2	Pectoralis major weight QTL (1954)
3	31343215	rs316268789	EIF2AK2	Cholesterol level QTL (1955)
3	31343215	rs316268789	EIF2AK2	Abdominal fat weight QTL (1958)
3	31343215	rs316268789	EIF2AK2	Body weight (8 days) QTL (6599)
3	31343215	rs316268789	EIF2AK2	Body weight (46 days) QTL (6600)
3	31343215	rs316268789	EIF2AK2	Body weight (112 days) QTL (6601)
3	31343215	rs316268789	EIF2AK2	Growth (1-8 days) QTL (6602)
3	31343215	rs316268789	EIF2AK2	Growth (8-46 days) QTL (6603)
3	31343215	rs316268789	EIF2AK2	Chest width QTL (9303)
3	31343215	rs316268789	EIF2AK2	Feather pecking QTL (9354)
3	31343215	rs316268789	EIF2AK2	Tibia bone mineral density QTL (24357)
3	31343215	rs316268789	EIF2AK2	Abdominal fat weight QTL (1952)
3	31343215	rs316268789	EIF2AK2	Abdominal fat percentage QTL (17287)
3	31343215	rs316268789	EIF2AK2	Abdominal fat weight QTL (17289)
3	31343215	rs316268789	EIF2AK2	Body weight (14 days) QTL (17273)
3	31343215	rs316268789	EIF2AK2	Body weight (28 days) QTL (17275)
3	31343215	rs316268789	EIF2AK2	Body weight (21 days) QTL (17274)
3	31343215	rs316268789	EIF2AK2	Body weight (35 days) QTL (17276)
3	31343215	rs316268789	EIF2AK2	Antibody titer to LPS antigen QTL (1953)
3	31343215	rs316268789	EIF2AK2	Body weight QTL (1979)
3	31343215	rs316268789	EIF2AK2	Body weight QTL (1980)
3	31343215	rs316268789	EIF2AK2	Body weight (35 days) QTL (7180)
3	31343215	rs316268789	EIF2AK2	Body weight (35 days) QTL (55904)
3	31343215	rs316268789	EIF2AK2	Growth (0-35 days) QTL (55929)
3	31343215	rs316268789	EIF2AK2	Body weight QTL (1957)
3	31344039	rs316497105	EIF2AK2	Abdominal fat percentage QTL (9417)
3	31344039	rs316497105	EIF2AK2	Abdominal fat weight QTL (9418)
3	31344039	rs316497105	EIF2AK2	Breast muscle percentage QTL (9416)
3	31344039	rs316497105	EIF2AK2	Body weight QTL (1951)

3	31344039	rs316497105	EIF2AK2	Tibia bone mineral density QTL (1950)
3	31344039	rs316497105	EIF2AK2	Head percentage QTL (15598)
3	31344039	rs316497105	EIF2AK2	Body weight (1 day) QTL (7167)
3	31344039	rs316497105	EIF2AK2	Body weight (35 days) QTL (7171)
3	31344039	rs316497105	EIF2AK2	Body weight (41 days) QTL (7174)
3	31344039	rs316497105	EIF2AK2	Pectoralis major weight QTL (1954)
3	31344039	rs316497105	EIF2AK2	Cholesterol level QTL (1955)
3	31344039	rs316497105	EIF2AK2	Abdominal fat weight QTL (1958)
3	31344039	rs316497105	EIF2AK2	Body weight (8 days) QTL (6599)
3	31344039	rs316497105	EIF2AK2	Body weight (46 days) QTL (6600)
3	31344039	rs316497105	EIF2AK2	Body weight (112 days) QTL (6601)
3	31344039	rs316497105	EIF2AK2	Growth (1-8 days) QTL (6602)
3	31344039	rs316497105	EIF2AK2	Growth (8-46 days) QTL (6603)
3	31344039	rs316497105	EIF2AK2	Chest width QTL (9303)
3	31344039	rs316497105	EIF2AK2	Feather pecking QTL (9354)
3	31344039	rs316497105	EIF2AK2	Tibia bone mineral density QTL (24357)
3	31344039	rs316497105	EIF2AK2	Abdominal fat weight QTL (1952)
3	31344039	rs316497105	EIF2AK2	Abdominal fat percentage QTL (17287)
3	31344039	rs316497105	EIF2AK2	Abdominal fat weight QTL (17289)
3	31344039	rs316497105	EIF2AK2	Body weight (14 days) QTL (17273)
3	31344039	rs316497105	EIF2AK2	Body weight (28 days) QTL (17275)
3	31344039	rs316497105	EIF2AK2	Body weight (21 days) QTL (17274)
3	31344039	rs316497105	EIF2AK2	Body weight (35 days) QTL (17276)
3	31344039	rs316497105	EIF2AK2	Antibody titer to LPS antigen QTL (1953)
3	31344039	rs316497105	EIF2AK2	Body weight QTL (1979)
3	31344039	rs316497105	EIF2AK2	Body weight QTL (1980)
3	31344039	rs316497105	EIF2AK2	Body weight (35 days) QTL (7180)
3	31344039	rs316497105	EIF2AK2	Body weight (35 days) QTL (55904)
3	31344039	rs316497105	EIF2AK2	Growth (0-35 days) QTL (55929)
3	31344039	rs316497105	EIF2AK2	Body weight QTL (1957)
3	31345086	rs736774899	EIF2AK2	Abdominal fat percentage QTL (9417)
3	31345086	rs736774899	EIF2AK2	Abdominal fat weight QTL (9418)
3	31345086	rs736774899	EIF2AK2	Breast muscle percentage QTL (9416)
3	31345086	rs736774899	EIF2AK2	Body weight QTL (1951)
3	31345086	rs736774899	EIF2AK2	Tibia bone mineral density QTL (1950)
3	31345086	rs736774899	EIF2AK2	Head percentage QTL (15598)
3	31345086	rs736774899	EIF2AK2	Body weight (1 day) QTL (7167)
3	31345086	rs736774899	EIF2AK2	Body weight (35 days) QTL (7171)
3	31345086	rs736774899	EIF2AK2	Body weight (41 days) QTL (7174)
3	31345086	rs736774899	EIF2AK2	Pectoralis major weight QTL (1954)
3	31345086	rs736774899	EIF2AK2	Cholesterol level QTL (1955)

3	31345086	rs736774899	EIF2AK2	Abdominal fat weight QTL (1958)
3	31345086	rs736774899	EIF2AK2	Body weight (8 days) QTL (6599)
3	31345086	rs736774899	EIF2AK2	Body weight (46 days) QTL (6600)
3	31345086	rs736774899	EIF2AK2	Body weight (112 days) QTL (6601)
3	31345086	rs736774899	EIF2AK2	Growth (1-8 days) QTL (6602)
3	31345086	rs736774899	EIF2AK2	Growth (8-46 days) QTL (6603)
3	31345086	rs736774899	EIF2AK2	Chest width QTL (9303)
3	31345086	rs736774899	EIF2AK2	Feather pecking QTL (9354)
3	31345086	rs736774899	EIF2AK2	Tibia bone mineral density QTL (24357)
3	31345086	rs736774899	EIF2AK2	Abdominal fat weight QTL (1952)
3	31345086	rs736774899	EIF2AK2	Abdominal fat percentage QTL (17287)
3	31345086	rs736774899	EIF2AK2	Abdominal fat weight QTL (17289)
3	31345086	rs736774899	EIF2AK2	Body weight (14 days) QTL (17273)
3	31345086	rs736774899	EIF2AK2	Body weight (28 days) QTL (17275)
3	31345086	rs736774899	EIF2AK2	Body weight (21 days) QTL (17274)
3	31345086	rs736774899	EIF2AK2	Body weight (35 days) QTL (17276)
3	31345086	rs736774899	EIF2AK2	Antibody titer to LPS antigen QTL (1953)
3	31345086	rs736774899	EIF2AK2	Body weight QTL (1979)
3	31345086	rs736774899	EIF2AK2	Body weight QTL (1980)
3	31345086	rs736774899	EIF2AK2	Body weight (35 days) QTL (7180)
3	31345086	rs736774899	EIF2AK2	Body weight (35 days) QTL (55904)
3	31345086	rs736774899	EIF2AK2	Growth (0-35 days) QTL (55929)
3	31345086	rs736774899	EIF2AK2	Body weight QTL (1957)
4	33493534	rs739281432	CDKL2	Head percentage QTL (15571)
4	33493534	rs739281432	CDKL2	Marek's disease-related traits QTL (2002)
4	33493534	rs739281432	CDKL2	Conformation score QTL (9382)
4	33493534	rs739281432	CDKL2	Thigh muscle weight QTL (9395)
4	33493534	rs739281432	CDKL2	Drumstick and thigh muscle weight QTL (13404)
4	33493534	rs739281432	CDKL2	Creatine kinase level QTL (1994)
4	33493534	rs739281432	CDKL2	Fear-related behavior QTL (1995)
4	33493534	rs739281432	CDKL2	Egg weight QTL (17063)
4	33493534	rs739281432	CDKL2	Tibia weight QTL (9326)
4	33493534	rs739281432	CDKL2	Breast muscle weight QTL (6690)
4	33493534	rs739281432	CDKL2	Carcass weight QTL (6691)
4	33493534	rs739281432	CDKL2	Body weight (40 days) QTL (6692)
4	33493534	rs739281432	CDKL2	Visceral fat weight QTL (17321)
4	33493534	rs739281432	CDKL2	Average daily gain QTL (24914)
4	33493534	rs739281432	CDKL2	Egg weight QTL (17065)
4	33493534	rs739281432	CDKL2	Age at first egg QTL (14452)
4	33493534	rs739281432	CDKL2	Body weight (day of first egg) QTL (14457)
4	33493534	rs739281432	CDKL2	Body weight (day of first egg) QTL (14464)

4	33493534	rs739281432	CDKL2	Body weight (day of first egg) QTL (14470)
4	33493534	rs739281432	CDKL2	Body weight (168 days) QTL (24875)
4	33493534	rs739281432	CDKL2	Body weight (21 days) QTL (24842)
4	33493534	rs739281432	CDKL2	Body weight (336 days) QTL (24883)
4	33493534	rs739281432	CDKL2	Body weight (42 days) QTL (24855)
4	33493534	rs739281432	CDKL2	Average daily gain QTL (24899)
4	33493534	rs739281432	CDKL2	Average daily gain QTL (24905)
4	33493534	rs739281432	CDKL2	Body weight (84 days) QTL (24866)
4	33493534	rs739281432	CDKL2	Body weight (504 days) QTL (24890)
4	33493534	rs739281432	CDKL2	Average daily gain QTL (24911)
4	33493534	rs739281432	CDKL2	Egg number QTL (17062)
4	33493534	rs739281432	CDKL2	Egg weight QTL (17064)
4	33493534	rs739281432	CDKL2	Tibia width QTL (2035)
4	33493534	rs739281432	CDKL2	Yolk height QTL (36773)
4	33493534	rs739281432	CDKL2	Body weight (35 days) QTL (55905)
4	33493534	rs739281432	CDKL2	Growth (0-35 days) QTL (55930)
4	33493536	rs731824742	CDKL2	Head percentage QTL (15571)
4	33493536	rs731824742	CDKL2	Marek's disease-related traits QTL (2002)
4	33493536	rs731824742	CDKL2	Conformation score QTL (9382)
4	33493536	rs731824742	CDKL2	Thigh muscle weight QTL (9395)
4	33493536	rs731824742	CDKL2	Drumstick and thigh muscle weight QTL (13404)
4	33493536	rs731824742	CDKL2	Creatine kinase level QTL (1994)
4	33493536	rs731824742	CDKL2	Fear-related behavior QTL (1995)
4	33493536	rs731824742	CDKL2	Egg weight QTL (17063)
4	33493536	rs731824742	CDKL2	Tibia weight QTL (9326)
4	33493536	rs731824742	CDKL2	Breast muscle weight QTL (6690)
4	33493536	rs731824742	CDKL2	Carcass weight QTL (6691)
4	33493536	rs731824742	CDKL2	Body weight (40 days) QTL (6692)
4	33493536	rs731824742	CDKL2	Visceral fat weight QTL (17321)
4	33493536	rs731824742	CDKL2	Average daily gain QTL (24914)
4	33493536	rs731824742	CDKL2	Egg weight QTL (17065)
4	33493536	rs731824742	CDKL2	Age at first egg QTL (14452)
4	33493536	rs731824742	CDKL2	Body weight (day of first egg) QTL (14457)
4	33493536	rs731824742	CDKL2	Body weight (day of first egg) QTL (14464)
4	33493536	rs731824742	CDKL2	Body weight (day of first egg) QTL (14470)
4	33493536	rs731824742	CDKL2	Body weight (168 days) QTL (24875)
4	33493536	rs731824742	CDKL2	Body weight (21 days) QTL (24842)
4	33493536	rs731824742	CDKL2	Body weight (336 days) QTL (24883)
4	33493536	rs731824742	CDKL2	Body weight (42 days) QTL (24855)
4	33493536	rs731824742	CDKL2	Average daily gain QTL (24899)
4	33493536	rs731824742	CDKL2	Average daily gain QTL (24905)

4	33493536	rs731824742	CDKL2	Body weight (84 days) QTL (24866)
4	33493536	rs731824742	CDKL2	Body weight (504 days) QTL (24890)
4	33493536	rs731824742	CDKL2	Average daily gain QTL (24911)
4	33493536	rs731824742	CDKL2	Egg number QTL (17062)
4	33493536	rs731824742	CDKL2	Egg weight QTL (17064)
4	33493536	rs731824742	CDKL2	Tibia width QTL (2035)
4	33493536	rs731824742	CDKL2	Yolk height QTL (36773)
4	33493536	rs731824742	CDKL2	Body weight (35 days) QTL (55905)
4	33493536	rs731824742	CDKL2	Growth (0-35 days) QTL (55930)
4	33493544	rs313558088	CDKL2	Head percentage QTL (15571)
4	33493544	rs313558088	CDKL2	Marek's disease-related traits QTL (2002)
4	33493544	rs313558088	CDKL2	Conformation score QTL (9382)
4	33493544	rs313558088	CDKL2	Thigh muscle weight QTL (9395)
4	33493544	rs313558088	CDKL2	Drumstick and thigh muscle weight QTL (13404)
4	33493544	rs313558088	CDKL2	Creatine kinase level QTL (1994)
4	33493544	rs313558088	CDKL2	Fear-related behavior QTL (1995)
4	33493544	rs313558088	CDKL2	Egg weight QTL (17063)
4	33493544	rs313558088	CDKL2	Tibia weight QTL (9326)
4	33493544	rs313558088	CDKL2	Breast muscle weight QTL (6690)
4	33493544	rs313558088	CDKL2	Carcass weight QTL (6691)
4	33493544	rs313558088	CDKL2	Body weight (40 days) QTL (6692)
4	33493544	rs313558088	CDKL2	Visceral fat weight QTL (17321)
4	33493544	rs313558088	CDKL2	Average daily gain QTL (24914)
4	33493544	rs313558088	CDKL2	Egg weight QTL (17065)
4	33493544	rs313558088	CDKL2	Age at first egg QTL (14452)
4	33493544	rs313558088	CDKL2	Body weight (day of first egg) QTL (14457)
4	33493544	rs313558088	CDKL2	Body weight (day of first egg) QTL (14464)
4	33493544	rs313558088	CDKL2	Body weight (day of first egg) QTL (14470)
4	33493544	rs313558088	CDKL2	Body weight (168 days) QTL (24875)
4	33493544	rs313558088	CDKL2	Body weight (21 days) QTL (24842)
4	33493544	rs313558088	CDKL2	Body weight (336 days) QTL (24883)
4	33493544	rs313558088	CDKL2	Body weight (42 days) QTL (24855)
4	33493544	rs313558088	CDKL2	Average daily gain QTL (24899)
4	33493544	rs313558088	CDKL2	Average daily gain QTL (24905)
4	33493544	rs313558088	CDKL2	Body weight (84 days) QTL (24866)
4	33493544	rs313558088	CDKL2	Body weight (504 days) QTL (24890)
4	33493544	rs313558088	CDKL2	Average daily gain QTL (24911)
4	33493544	rs313558088	CDKL2	Egg number QTL (17062)
4	33493544	rs313558088	CDKL2	Egg weight QTL (17064)
4	33493544	rs313558088	CDKL2	Tibia width QTL (2035)
4	33493544	rs313558088	CDKL2	Yolk height QTL (36773)

4	33493544	rs313558088	CDKL2	Body weight (35 days) QTL (55905)
4	33493544	rs313558088	CDKL2	Growth (0-35 days) QTL (55930)
4	33493943	rs738401365	CDKL2	Head percentage QTL (15571)
4	33493943	rs738401365	CDKL2	Marek's disease-related traits QTL (2002)
4	33493943	rs738401365	CDKL2	Conformation score QTL (9382)
4	33493943	rs738401365	CDKL2	Thigh muscle weight QTL (9395)
4	33493943	rs738401365	CDKL2	Drumstick and thigh muscle weight QTL (13404)
4	33493943	rs738401365	CDKL2	Creatine kinase level QTL (1994)
4	33493943	rs738401365	CDKL2	Fear-related behavior QTL (1995)
4	33493943	rs738401365	CDKL2	Egg weight QTL (17063)
4	33493943	rs738401365	CDKL2	Tibia weight QTL (9326)
4	33493943	rs738401365	CDKL2	Breast muscle weight QTL (6690)
4	33493943	rs738401365	CDKL2	Carcass weight QTL (6691)
4	33493943	rs738401365	CDKL2	Body weight (40 days) QTL (6692)
4	33493943	rs738401365	CDKL2	Visceral fat weight QTL (17321)
4	33493943	rs738401365	CDKL2	Average daily gain QTL (24914)
4	33493943	rs738401365	CDKL2	Egg weight QTL (17065)
4	33493943	rs738401365	CDKL2	Age at first egg QTL (14452)
4	33493943	rs738401365	CDKL2	Body weight (day of first egg) QTL (14457)
4	33493943	rs738401365	CDKL2	Body weight (day of first egg) QTL (14464)
4	33493943	rs738401365	CDKL2	Body weight (day of first egg) QTL (14470)
4	33493943	rs738401365	CDKL2	Body weight (168 days) QTL (24875)
4	33493943	rs738401365	CDKL2	Body weight (21 days) QTL (24842)
4	33493943	rs738401365	CDKL2	Body weight (336 days) QTL (24883)
4	33493943	rs738401365	CDKL2	Body weight (42 days) QTL (24855)
4	33493943	rs738401365	CDKL2	Average daily gain QTL (24899)
4	33493943	rs738401365	CDKL2	Average daily gain QTL (24905)
4	33493943	rs738401365	CDKL2	Body weight (84 days) QTL (24866)
4	33493943	rs738401365	CDKL2	Body weight (504 days) QTL (24890)
4	33493943	rs738401365	CDKL2	Average daily gain QTL (24911)
4	33493943	rs738401365	CDKL2	Egg number QTL (17062)
4	33493943	rs738401365	CDKL2	Egg weight QTL (17064)
4	33493943	rs738401365	CDKL2	Tibia width QTL (2035)
4	33493943	rs738401365	CDKL2	Yolk height QTL (36773)
4	33493943	rs738401365	CDKL2	Body weight (35 days) QTL (55905)
4	33493943	rs738401365	CDKL2	Growth (0-35 days) QTL (55930)
4	33493947	rs741004252	CDKL2	Head percentage QTL (15571)
4	33493947	rs741004252	CDKL2	Marek's disease-related traits QTL (2002)
4	33493947	rs741004252	CDKL2	Conformation score QTL (9382)
4	33493947	rs741004252	CDKL2	Thigh muscle weight QTL (9395)

4	33493947	rs741004252	CDKL2	Drumstick and thigh muscle weight QTL (13404)
4	33493947	rs741004252	CDKL2	Creatine kinase level QTL (1994)
4	33493947	rs741004252	CDKL2	Fear-related behavior QTL (1995)
4	33493947	rs741004252	CDKL2	Egg weight QTL (17063)
4	33493947	rs741004252	CDKL2	Tibia weight QTL (9326)
4	33493947	rs741004252	CDKL2	Breast muscle weight QTL (6690)
4	33493947	rs741004252	CDKL2	Carcass weight QTL (6691)
4	33493947	rs741004252	CDKL2	Body weight (40 days) QTL (6692)
4	33493947	rs741004252	CDKL2	Visceral fat weight QTL (17321)
4	33493947	rs741004252	CDKL2	Average daily gain QTL (24914)
4	33493947	rs741004252	CDKL2	Egg weight QTL (17065)
4	33493947	rs741004252	CDKL2	Age at first egg QTL (14452)
4	33493947	rs741004252	CDKL2	Body weight (day of first egg) QTL (14457)
4	33493947	rs741004252	CDKL2	Body weight (day of first egg) QTL (14464)
4	33493947	rs741004252	CDKL2	Body weight (day of first egg) QTL (14470)
4	33493947	rs741004252	CDKL2	Body weight (168 days) QTL (24875)
4	33493947	rs741004252	CDKL2	Body weight (21 days) QTL (24842)
4	33493947	rs741004252	CDKL2	Body weight (336 days) QTL (24883)
4	33493947	rs741004252	CDKL2	Body weight (42 days) QTL (24855)
4	33493947	rs741004252	CDKL2	Average daily gain QTL (24899)
4	33493947	rs741004252	CDKL2	Average daily gain QTL (24905)
4	33493947	rs741004252	CDKL2	Body weight (84 days) QTL (24866)
4	33493947	rs741004252	CDKL2	Body weight (504 days) QTL (24890)
4	33493947	rs741004252	CDKL2	Average daily gain QTL (24911)
4	33493947	rs741004252	CDKL2	Egg number QTL (17062)
4	33493947	rs741004252	CDKL2	Egg weight QTL (17064)
4	33493947	rs741004252	CDKL2	Tibia width QTL (2035)
4	33493947	rs741004252	CDKL2	Yolk height QTL (36773)
4	33493947	rs741004252	CDKL2	Body weight (35 days) QTL (55905)
4	33493947	rs741004252	CDKL2	Growth (0-35 days) QTL (55930)
4	33493973	rs314887600	CDKL2	Head percentage QTL (15571)
4	33493973	rs314887600	CDKL2	Marek's disease-related traits QTL (2002)
4	33493973	rs314887600	CDKL2	Conformation score QTL (9382)
4	33493973	rs314887600	CDKL2	Thigh muscle weight QTL (9395)
4	33493973	rs314887600	CDKL2	Drumstick and thigh muscle weight QTL (13404)
4	33493973	rs314887600	CDKL2	Creatine kinase level QTL (1994)
4	33493973	rs314887600	CDKL2	Fear-related behavior QTL (1995)
4	33493973	rs314887600	CDKL2	Egg weight QTL (17063)
4	33493973	rs314887600	CDKL2	Tibia weight QTL (9326)
4	33493973	rs314887600	CDKL2	Breast muscle weight QTL (6690)

4	33493973	rs314887600	CDKL2	Carcass weight QTL (6691)
4	33493973	rs314887600	CDKL2	Body weight (40 days) QTL (6692)
4	33493973	rs314887600	CDKL2	Visceral fat weight QTL (17321)
4	33493973	rs314887600	CDKL2	Average daily gain QTL (24914)
4	33493973	rs314887600	CDKL2	Egg weight QTL (17065)
4	33493973	rs314887600	CDKL2	Age at first egg QTL (14452)
4	33493973	rs314887600	CDKL2	Body weight (day of first egg) QTL (14457)
4	33493973	rs314887600	CDKL2	Body weight (day of first egg) QTL (14464)
4	33493973	rs314887600	CDKL2	Body weight (day of first egg) QTL (14470)
4	33493973	rs314887600	CDKL2	Body weight (168 days) QTL (24875)
4	33493973	rs314887600	CDKL2	Body weight (21 days) QTL (24842)
4	33493973	rs314887600	CDKL2	Body weight (336 days) QTL (24883)
4	33493973	rs314887600	CDKL2	Body weight (42 days) QTL (24855)
4	33493973	rs314887600	CDKL2	Average daily gain QTL (24899)
4	33493973	rs314887600	CDKL2	Average daily gain QTL (24905)
4	33493973	rs314887600	CDKL2	Body weight (84 days) QTL (24866)
4	33493973	rs314887600	CDKL2	Body weight (504 days) QTL (24890)
4	33493973	rs314887600	CDKL2	Average daily gain QTL (24911)
4	33493973	rs314887600	CDKL2	Egg number QTL (17062)
4	33493973	rs314887600	CDKL2	Egg weight QTL (17064)
4	33493973	rs314887600	CDKL2	Tibia width QTL (2035)
4	33493973	rs314887600	CDKL2	Yolk height QTL (36773)
4	33493973	rs314887600	CDKL2	Body weight (35 days) QTL (55905)
4	33493973	rs314887600	CDKL2	Growth (0-35 days) QTL (55930)
4	33493977	rs317109468	CDKL2	Head percentage QTL (15571)
4	33493977	rs317109468	CDKL2	Marek's disease-related traits QTL (2002)
4	33493977	rs317109468	CDKL2	Conformation score QTL (9382)
4	33493977	rs317109468	CDKL2	Thigh muscle weight QTL (9395)
4	33493977	rs317109468	CDKL2	Drumstick and thigh muscle weight QTL (13404)
4	33493977	rs317109468	CDKL2	Creatine kinase level QTL (1994)
4	33493977	rs317109468	CDKL2	Fear-related behavior QTL (1995)
4	33493977	rs317109468	CDKL2	Egg weight QTL (17063)
4	33493977	rs317109468	CDKL2	Tibia weight QTL (9326)
4	33493977	rs317109468	CDKL2	Breast muscle weight QTL (6690)
4	33493977	rs317109468	CDKL2	Carcass weight QTL (6691)
4	33493977	rs317109468	CDKL2	Body weight (40 days) QTL (6692)
4	33493977	rs317109468	CDKL2	Visceral fat weight QTL (17321)
4	33493977	rs317109468	CDKL2	Average daily gain QTL (24914)
4	33493977	rs317109468	CDKL2	Egg weight QTL (17065)
4	33493977	rs317109468	CDKL2	Age at first egg QTL (14452)
4	33493977	rs317109468	CDKL2	Body weight (day of first egg) QTL (14457)

4	33493977	rs317109468	CDKL2	Body weight (day of first egg) QTL (14464)
4	33493977	rs317109468	CDKL2	Body weight (day of first egg) QTL (14470)
4	33493977	rs317109468	CDKL2	Body weight (168 days) QTL (24875)
4	33493977	rs317109468	CDKL2	Body weight (21 days) QTL (24842)
4	33493977	rs317109468	CDKL2	Body weight (336 days) QTL (24883)
4	33493977	rs317109468	CDKL2	Body weight (42 days) QTL (24855)
4	33493977	rs317109468	CDKL2	Average daily gain QTL (24899)
4	33493977	rs317109468	CDKL2	Average daily gain QTL (24905)
4	33493977	rs317109468	CDKL2	Body weight (84 days) QTL (24866)
4	33493977	rs317109468	CDKL2	Body weight (504 days) QTL (24890)
4	33493977	rs317109468	CDKL2	Average daily gain QTL (24911)
4	33493977	rs317109468	CDKL2	Egg number QTL (17062)
4	33493977	rs317109468	CDKL2	Egg weight QTL (17064)
4	33493977	rs317109468	CDKL2	Tibia width QTL (2035)
4	33493977	rs317109468	CDKL2	Yolk height QTL (36773)
4	33493977	rs317109468	CDKL2	Body weight (35 days) QTL (55905)
4	33493977	rs317109468	CDKL2	Growth (0-35 days) QTL (55930)
4	33494004	rs738418882	CDKL2	Head percentage QTL (15571)
4	33494004	rs738418882	CDKL2	Marek's disease-related traits QTL (2002)
4	33494004	rs738418882	CDKL2	Conformation score QTL (9382)
4	33494004	rs738418882	CDKL2	Thigh muscle weight QTL (9395)
4	33494004	rs738418882	CDKL2	Drumstick and thigh muscle weight QTL (13404)
4	33494004	rs738418882	CDKL2	Creatine kinase level QTL (1994)
4	33494004	rs738418882	CDKL2	Fear-related behavior QTL (1995)
4	33494004	rs738418882	CDKL2	Egg weight QTL (17063)
4	33494004	rs738418882	CDKL2	Tibia weight QTL (9326)
4	33494004	rs738418882	CDKL2	Breast muscle weight QTL (6690)
4	33494004	rs738418882	CDKL2	Carcass weight QTL (6691)
4	33494004	rs738418882	CDKL2	Body weight (40 days) QTL (6692)
4	33494004	rs738418882	CDKL2	Visceral fat weight QTL (17321)
4	33494004	rs738418882	CDKL2	Average daily gain QTL (24914)
4	33494004	rs738418882	CDKL2	Egg weight QTL (17065)
4	33494004	rs738418882	CDKL2	Age at first egg QTL (14452)
4	33494004	rs738418882	CDKL2	Body weight (day of first egg) QTL (14457)
4	33494004	rs738418882	CDKL2	Body weight (day of first egg) QTL (14464)
4	33494004	rs738418882	CDKL2	Body weight (day of first egg) QTL (14470)
4	33494004	rs738418882	CDKL2	Body weight (168 days) QTL (24875)
4	33494004	rs738418882	CDKL2	Body weight (21 days) QTL (24842)
4	33494004	rs738418882	CDKL2	Body weight (336 days) QTL (24883)
4	33494004	rs738418882	CDKL2	Body weight (42 days) QTL (24855)
4	33494004	rs738418882	CDKL2	Average daily gain QTL (24899)

4	33494004	rs738418882	CDKL2	Average daily gain QTL (24905)
4	33494004	rs738418882	CDKL2	Body weight (84 days) QTL (24866)
4	33494004	rs738418882	CDKL2	Body weight (504 days) QTL (24890)
4	33494004	rs738418882	CDKL2	Average daily gain QTL (24911)
4	33494004	rs738418882	CDKL2	Egg number QTL (17062)
4	33494004	rs738418882	CDKL2	Egg weight QTL (17064)
4	33494004	rs738418882	CDKL2	Tibia width QTL (2035)
4	33494004	rs738418882	CDKL2	Yolk height QTL (36773)
4	33494004	rs738418882	CDKL2	Body weight (35 days) QTL (55905)
4	33494004	rs738418882	CDKL2	Growth (0-35 days) QTL (55930)
4	33494006	rs730944916	CDKL2	Head percentage QTL (15571)
4	33494006	rs730944916	CDKL2	Marek's disease-related traits QTL (2002)
4	33494006	rs730944916	CDKL2	Conformation score QTL (9382)
4	33494006	rs730944916	CDKL2	Thigh muscle weight QTL (9395)
4	33494006	rs730944916	CDKL2	Drumstick and thigh muscle weight QTL (13404)
4	33494006	rs730944916	CDKL2	Creatine kinase level QTL (1994)
4	33494006	rs730944916	CDKL2	Fear-related behavior QTL (1995)
4	33494006	rs730944916	CDKL2	Egg weight QTL (17063)
4	33494006	rs730944916	CDKL2	Tibia weight QTL (9326)
4	33494006	rs730944916	CDKL2	Breast muscle weight QTL (6690)
4	33494006	rs730944916	CDKL2	Carcass weight QTL (6691)
4	33494006	rs730944916	CDKL2	Body weight (40 days) QTL (6692)
4	33494006	rs730944916	CDKL2	Visceral fat weight QTL (17321)
4	33494006	rs730944916	CDKL2	Average daily gain QTL (24914)
4	33494006	rs730944916	CDKL2	Egg weight QTL (17065)
4	33494006	rs730944916	CDKL2	Age at first egg QTL (14452)
4	33494006	rs730944916	CDKL2	Body weight (day of first egg) QTL (14457)
4	33494006	rs730944916	CDKL2	Body weight (day of first egg) QTL (14464)
4	33494006	rs730944916	CDKL2	Body weight (day of first egg) QTL (14470)
4	33494006	rs730944916	CDKL2	Body weight (168 days) QTL (24875)
4	33494006	rs730944916	CDKL2	Body weight (21 days) QTL (24842)
4	33494006	rs730944916	CDKL2	Body weight (336 days) QTL (24883)
4	33494006	rs730944916	CDKL2	Body weight (42 days) QTL (24855)
4	33494006	rs730944916	CDKL2	Average daily gain QTL (24899)
4	33494006	rs730944916	CDKL2	Average daily gain QTL (24905)
4	33494006	rs730944916	CDKL2	Body weight (84 days) QTL (24866)
4	33494006	rs730944916	CDKL2	Body weight (504 days) QTL (24890)
4	33494006	rs730944916	CDKL2	Average daily gain QTL (24911)
4	33494006	rs730944916	CDKL2	Egg number QTL (17062)
4	33494006	rs730944916	CDKL2	Egg weight QTL (17064)
4	33494006	rs730944916	CDKL2	Tibia width QTL (2035)

4	33494006	rs730944916	CDKL2	Yolk height QTL (36773)
4	33494006	rs730944916	CDKL2	Body weight (35 days) QTL (55905)
4	33494006	rs730944916	CDKL2	Growth (0-35 days) QTL (55930)

Supplementary Table 24: Enriched QTL traits from the significant eQTLs of Ileum of low and high FCR broilers

chr	pos	snp_id	Gene	QTL_id
7	13183971	rs317374719	ENSGALG00000008738	Abdominal fat weight QTL (2140)
7	13183971	rs317374719	ENSGALG00000008738	Abdominal fat weight QTL (12629)
7	13183971	rs317374719	ENSGALG00000008738	Skin fat weight QTL (12639)
7	13183971	rs317374719	ENSGALG00000008738	Fat distribution QTL (12644)
7	13183971	rs317374719	ENSGALG00000008738	Drumstick and thigh muscle weight QTL (13397)
7	13183971	rs317374719	ENSGALG00000008738	Drumstick and thigh muscle weight QTL (13407)
7	13183971	rs317374719	ENSGALG00000008738	Body weight (112 days) QTL (6626)
7	13183971	rs317374719	ENSGALG00000008738	Body weight (200 days) QTL (6627)
7	13183971	rs317374719	ENSGALG00000008738	Body weight QTL (2136)
7	13183971	rs317374719	ENSGALG00000008738	Average daily gain QTL (24915)
7	13183971	rs317374719	ENSGALG00000008738	Wing weight QTL (2137)
7	13183971	rs317374719	ENSGALG00000008738	Thigh muscle weight QTL (2139)
7	13183971	rs317374719	ENSGALG00000008738	Thigh weight QTL (2141)
7	13183971	rs317374719	ENSGALG00000008738	Drumstick weight QTL (2142)
7	13183971	rs317374719	ENSGALG00000008738	Carcass weight QTL (2143)
7	13183971	rs317374719	ENSGALG00000008738	Drumstick muscle weight QTL (2144)
7	13183971	rs317374719	ENSGALG00000008738	Breast muscle weight QTL (2145)
7	13183971	rs317374719	ENSGALG00000008738	Body weight QTL (2149)
7	13183971	rs317374719	ENSGALG00000008738	Body weight QTL (2151)
7	13183971	rs317374719	ENSGALG00000008738	Body weight QTL (2153)
7	13183971	rs317374719	ENSGALG00000008738	Body weight QTL (2147)
7	13183971	rs317374719	ENSGALG00000008738	Body weight QTL (2148)
7	13183971	rs317374719	ENSGALG00000008738	Body weight QTL (2150)
7	13183971	rs317374719	ENSGALG00000008738	Body weight QTL (2146)
7	13183971	rs317374719	ENSGALG00000008738	Body weight QTL (2158)
7	13183971	rs317374719	ENSGALG00000008738	Body weight QTL (2160)
7	13183971	rs317374719	ENSGALG00000008738	Intestine length QTL (7166)
7	13183971	rs317374719	ENSGALG00000008738	Body weight (35 days) QTL (17308)
7	13183971	rs317374719	ENSGALG00000008738	Body weight (14 days) QTL (17305)
7	13183971	rs317374719	ENSGALG00000008738	Body weight (77 days) QTL (17314)
7	13184339	rs738577016	ENSGALG00000008738	Abdominal fat weight QTL (2140)
7	13184339	rs738577016	ENSGALG00000008738	Abdominal fat weight QTL (12629)
7	13184339	rs738577016	ENSGALG00000008738	Skin fat weight QTL (12639)
7	13184339	rs738577016	ENSGALG00000008738	Fat distribution QTL (12644)
7	13184339	rs738577016	ENSGALG00000008738	Drumstick and thigh muscle weight QTL (13397)

7	13184339	rs738577016	ENSGALG00000008738	Drumstick and thigh muscle weight QTL (13407)
7	13184339	rs738577016	ENSGALG00000008738	Body weight (112 days) QTL (6626)
7	13184339	rs738577016	ENSGALG00000008738	Body weight (200 days) QTL (6627)
7	13184339	rs738577016	ENSGALG00000008738	Body weight QTL (2136)
7	13184339	rs738577016	ENSGALG00000008738	Average daily gain QTL (24915)
7	13184339	rs738577016	ENSGALG00000008738	Wing weight QTL (2137)
7	13184339	rs738577016	ENSGALG00000008738	Thigh muscle weight QTL (2139)
7	13184339	rs738577016	ENSGALG00000008738	Thigh weight QTL (2141)
7	13184339	rs738577016	ENSGALG00000008738	Drumstick weight QTL (2142)
7	13184339	rs738577016	ENSGALG00000008738	Carcass weight QTL (2143)
7	13184339	rs738577016	ENSGALG00000008738	Drumstick muscle weight QTL (2144)
7	13184339	rs738577016	ENSGALG00000008738	Breast muscle weight QTL (2145)
7	13184339	rs738577016	ENSGALG00000008738	Body weight QTL (2149)
7	13184339	rs738577016	ENSGALG00000008738	Body weight QTL (2151)
7	13184339	rs738577016	ENSGALG00000008738	Body weight QTL (2153)
7	13184339	rs738577016	ENSGALG00000008738	Body weight QTL (2147)
7	13184339	rs738577016	ENSGALG00000008738	Body weight QTL (2148)
7	13184339	rs738577016	ENSGALG00000008738	Body weight QTL (2150)
7	13184339	rs738577016	ENSGALG00000008738	Body weight QTL (2146)
7	13184339	rs738577016	ENSGALG00000008738	Body weight QTL (2158)
7	13184339	rs738577016	ENSGALG00000008738	Body weight QTL (2160)
7	13184339	rs738577016	ENSGALG00000008738	Intestine length QTL (7166)
7	13184339	rs738577016	ENSGALG00000008738	Body weight (35 days) QTL (17308)
7	13184339	rs738577016	ENSGALG00000008738	Body weight (14 days) QTL (17305)
7	13184339	rs738577016	ENSGALG00000008738	Body weight (77 days) QTL (17314)
7	13184476	rs730965526	ENSGALG00000008738	Abdominal fat weight QTL (2140)
7	13184476	rs730965526	ENSGALG00000008738	Abdominal fat weight QTL (12629)
7	13184476	rs730965526	ENSGALG00000008738	Skin fat weight QTL (12639)
7	13184476	rs730965526	ENSGALG00000008738	Fat distribution QTL (12644)
7	13184476	rs730965526	ENSGALG00000008738	Drumstick and thigh muscle weight QTL (13397)
7	13184476	rs730965526	ENSGALG00000008738	Drumstick and thigh muscle weight QTL (13407)
7	13184476	rs730965526	ENSGALG00000008738	Body weight (112 days) QTL (6626)
7	13184476	rs730965526	ENSGALG00000008738	Body weight (200 days) QTL (6627)
7	13184476	rs730965526	ENSGALG00000008738	Body weight QTL (2136)
7	13184476	rs730965526	ENSGALG00000008738	Average daily gain QTL (24915)
7	13184476	rs730965526	ENSGALG00000008738	Wing weight QTL (2137)
7	13184476	rs730965526	ENSGALG00000008738	Thigh muscle weight QTL (2139)
7	13184476	rs730965526	ENSGALG00000008738	Thigh weight QTL (2141)
7	13184476	rs730965526	ENSGALG00000008738	Drumstick weight QTL (2142)

7	13184476	rs730965526	ENSGALG00000008738	Carcass weight QTL (2143)
7	13184476	rs730965526	ENSGALG00000008738	Drumstick muscle weight QTL (2144)
7	13184476	rs730965526	ENSGALG00000008738	Breast muscle weight QTL (2145)
7	13184476	rs730965526	ENSGALG00000008738	Body weight QTL (2149)
7	13184476	rs730965526	ENSGALG00000008738	Body weight QTL (2151)
7	13184476	rs730965526	ENSGALG00000008738	Body weight QTL (2153)
7	13184476	rs730965526	ENSGALG00000008738	Body weight QTL (2147)
7	13184476	rs730965526	ENSGALG00000008738	Body weight QTL (2148)
7	13184476	rs730965526	ENSGALG00000008738	Body weight QTL (2150)
7	13184476	rs730965526	ENSGALG00000008738	Body weight QTL (2146)
7	13184476	rs730965526	ENSGALG00000008738	Body weight QTL (2158)
7	13184476	rs730965526	ENSGALG00000008738	Body weight QTL (2160)
7	13184476	rs730965526	ENSGALG00000008738	Intestine length QTL (7166)
7	13184476	rs730965526	ENSGALG00000008738	Body weight (35 days) QTL (17308)
7	13184476	rs730965526	ENSGALG00000008738	Body weight (14 days) QTL (17305)
7	13184476	rs730965526	ENSGALG00000008738	Body weight (77 days) QTL (17314)
7	13184778	rs16586532	ENSGALG00000008738	Abdominal fat weight QTL (2140)
7	13184778	rs16586532	ENSGALG00000008738	Abdominal fat weight QTL (12629)
7	13184778	rs16586532	ENSGALG00000008738	Skin fat weight QTL (12639)
7	13184778	rs16586532	ENSGALG00000008738	Fat distribution QTL (12644)
7	13184778	rs16586532	ENSGALG00000008738	Drumstick and thigh muscle weight QTL (13397)
7	13184778	rs16586532	ENSGALG00000008738	Drumstick and thigh muscle weight QTL (13407)
7	13184778	rs16586532	ENSGALG00000008738	Body weight (112 days) QTL (6626)
7	13184778	rs16586532	ENSGALG00000008738	Body weight (200 days) QTL (6627)
7	13184778	rs16586532	ENSGALG00000008738	Body weight QTL (2136)
7	13184778	rs16586532	ENSGALG00000008738	Average daily gain QTL (24915)
7	13184778	rs16586532	ENSGALG00000008738	Wing weight QTL (2137)
7	13184778	rs16586532	ENSGALG00000008738	Thigh muscle weight QTL (2139)
7	13184778	rs16586532	ENSGALG00000008738	Thigh weight QTL (2141)
7	13184778	rs16586532	ENSGALG00000008738	Drumstick weight QTL (2142)
7	13184778	rs16586532	ENSGALG00000008738	Carcass weight QTL (2143)
7	13184778	rs16586532	ENSGALG00000008738	Drumstick muscle weight QTL (2144)
7	13184778	rs16586532	ENSGALG00000008738	Breast muscle weight QTL (2145)
7	13184778	rs16586532	ENSGALG00000008738	Body weight QTL (2149)
7	13184778	rs16586532	ENSGALG00000008738	Body weight QTL (2151)
7	13184778	rs16586532	ENSGALG00000008738	Body weight QTL (2153)
7	13184778	rs16586532	ENSGALG00000008738	Body weight QTL (2147)
7	13184778	rs16586532	ENSGALG00000008738	Body weight QTL (2148)
7	13184778	rs16586532	ENSGALG00000008738	Body weight QTL (2150)
7	13184778	rs16586532	ENSGALG00000008738	Body weight QTL (2146)

7	13184778	rs16586532	ENSGALG00000008738	Body weight QTL (2158)
7	13184778	rs16586532	ENSGALG00000008738	Body weight QTL (2160)
7	13184778	rs16586532	ENSGALG00000008738	Intestine length QTL (7166)
7	13184778	rs16586532	ENSGALG00000008738	Body weight (35 days) QTL (17308)
7	13184778	rs16586532	ENSGALG00000008738	Body weight (14 days) QTL (17305)
7	13184778	rs16586532	ENSGALG00000008738	Body weight (77 days) QTL (17314)
7	13184801	_Variant_385	ENSGALG00000008738	Abdominal fat weight QTL (2140)
7	13184801	_Variant_385	ENSGALG00000008738	Abdominal fat weight QTL (12629)
7	13184801	_Variant_385	ENSGALG00000008738	Skin fat weight QTL (12639)
7	13184801	_Variant_385	ENSGALG00000008738	Fat distribution QTL (12644)
7	13184801	_Variant_385	ENSGALG00000008738	Drumstick and thigh muscle weight QTL (13397)
7	13184801	_Variant_385	ENSGALG00000008738	Drumstick and thigh muscle weight QTL (13407)
7	13184801	_Variant_385	ENSGALG00000008738	Body weight (112 days) QTL (6626)
7	13184801	_Variant_385	ENSGALG00000008738	Body weight (200 days) QTL (6627)
7	13184801	_Variant_385	ENSGALG00000008738	Body weight QTL (2136)
7	13184801	_Variant_385	ENSGALG00000008738	Average daily gain QTL (24915)
7	13184801	_Variant_385	ENSGALG00000008738	Wing weight QTL (2137)
7	13184801	_Variant_385	ENSGALG00000008738	Thigh muscle weight QTL (2139)
7	13184801	_Variant_385	ENSGALG00000008738	Thigh weight QTL (2141)
7	13184801	_Variant_385	ENSGALG00000008738	Drumstick weight QTL (2142)
7	13184801	_Variant_385	ENSGALG00000008738	Carcass weight QTL (2143)
7	13184801	_Variant_385	ENSGALG00000008738	Drumstick muscle weight QTL (2144)
7	13184801	_Variant_385	ENSGALG00000008738	Breast muscle weight QTL (2145)
7	13184801	_Variant_385	ENSGALG00000008738	Body weight QTL (2149)
7	13184801	_Variant_385	ENSGALG00000008738	Body weight QTL (2151)
7	13184801	_Variant_385	ENSGALG00000008738	Body weight QTL (2153)
7	13184801	_Variant_385	ENSGALG00000008738	Body weight QTL (2147)
7	13184801	_Variant_385	ENSGALG00000008738	Body weight QTL (2148)
7	13184801	_Variant_385	ENSGALG00000008738	Body weight QTL (2150)
7	13184801	_Variant_385	ENSGALG00000008738	Body weight QTL (2146)
7	13184801	_Variant_385	ENSGALG00000008738	Body weight QTL (2158)
7	13184801	_Variant_385	ENSGALG00000008738	Body weight QTL (2160)
7	13184801	_Variant_385	ENSGALG00000008738	Intestine length QTL (7166)
7	13184801	_Variant_385	ENSGALG00000008738	Body weight (35 days) QTL (17308)
7	13184801	_Variant_385	ENSGALG00000008738	Body weight (14 days) QTL (17305)
7	13184801	_Variant_385	ENSGALG00000008738	Body weight (77 days) QTL (17314)
7	13185159	rs737662720	ENSGALG00000008738	Abdominal fat weight QTL (2140)
7	13185159	rs737662720	ENSGALG00000008738	Abdominal fat weight QTL (12629)
7	13185159	rs737662720	ENSGALG00000008738	Skin fat weight QTL (12639)
7	13185159	rs737662720	ENSGALG00000008738	Fat distribution QTL (12644)

7	13185159	rs737662720	ENSGALG00000008738	Drumstick and thigh muscle weight QTL (13397)
7	13185159	rs737662720	ENSGALG00000008738	Drumstick and thigh muscle weight QTL (13407)
7	13185159	rs737662720	ENSGALG00000008738	Body weight (112 days) QTL (6626)
7	13185159	rs737662720	ENSGALG00000008738	Body weight (200 days) QTL (6627)
7	13185159	rs737662720	ENSGALG00000008738	Body weight QTL (2136)
7	13185159	rs737662720	ENSGALG00000008738	Average daily gain QTL (24915)
7	13185159	rs737662720	ENSGALG00000008738	Wing weight QTL (2137)
7	13185159	rs737662720	ENSGALG00000008738	Thigh muscle weight QTL (2139)
7	13185159	rs737662720	ENSGALG00000008738	Thigh weight QTL (2141)
7	13185159	rs737662720	ENSGALG00000008738	Drumstick weight QTL (2142)
7	13185159	rs737662720	ENSGALG00000008738	Carcass weight QTL (2143)
7	13185159	rs737662720	ENSGALG00000008738	Drumstick muscle weight QTL (2144)
7	13185159	rs737662720	ENSGALG00000008738	Breast muscle weight QTL (2145)
7	13185159	rs737662720	ENSGALG00000008738	Body weight QTL (2149)
7	13185159	rs737662720	ENSGALG00000008738	Body weight QTL (2151)
7	13185159	rs737662720	ENSGALG00000008738	Body weight QTL (2153)
7	13185159	rs737662720	ENSGALG00000008738	Body weight QTL (2147)
7	13185159	rs737662720	ENSGALG00000008738	Body weight QTL (2148)
7	13185159	rs737662720	ENSGALG00000008738	Body weight QTL (2150)
7	13185159	rs737662720	ENSGALG00000008738	Body weight QTL (2146)
7	13185159	rs737662720	ENSGALG00000008738	Body weight QTL (2158)
7	13185159	rs737662720	ENSGALG00000008738	Body weight QTL (2160)
7	13185159	rs737662720	ENSGALG00000008738	Intestine length QTL (7166)
7	13185159	rs737662720	ENSGALG00000008738	Body weight (35 days) QTL (17308)
7	13185159	rs737662720	ENSGALG00000008738	Body weight (14 days) QTL (17305)
7	13185159	rs737662720	ENSGALG00000008738	Body weight (77 days) QTL (17314)
7	13186220	rs312536923	ENSGALG00000008738	Abdominal fat weight QTL (2140)
7	13186220	rs312536923	ENSGALG00000008738	Abdominal fat weight QTL (12629)
7	13186220	rs312536923	ENSGALG00000008738	Skin fat weight QTL (12639)
7	13186220	rs312536923	ENSGALG00000008738	Fat distribution QTL (12644)
7	13186220	rs312536923	ENSGALG00000008738	Drumstick and thigh muscle weight QTL (13397)
7	13186220	rs312536923	ENSGALG00000008738	Drumstick and thigh muscle weight QTL (13407)
7	13186220	rs312536923	ENSGALG00000008738	Body weight (112 days) QTL (6626)
7	13186220	rs312536923	ENSGALG00000008738	Body weight (200 days) QTL (6627)
7	13186220	rs312536923	ENSGALG00000008738	Body weight QTL (2136)
7	13186220	rs312536923	ENSGALG00000008738	Average daily gain QTL (24915)
7	13186220	rs312536923	ENSGALG00000008738	Wing weight QTL (2137)
7	13186220	rs312536923	ENSGALG00000008738	Thigh muscle weight QTL (2139)
7	13186220	rs312536923	ENSGALG00000008738	Thigh weight QTL (2141)

7	13186220	rs312536923	ENSGALG00000008738	Drumstick weight QTL (2142)
7	13186220	rs312536923	ENSGALG00000008738	Carcass weight QTL (2143)
7	13186220	rs312536923	ENSGALG00000008738	Drumstick muscle weight QTL (2144)
7	13186220	rs312536923	ENSGALG00000008738	Breast muscle weight QTL (2145)
7	13186220	rs312536923	ENSGALG00000008738	Body weight QTL (2149)
7	13186220	rs312536923	ENSGALG00000008738	Body weight QTL (2151)
7	13186220	rs312536923	ENSGALG00000008738	Body weight QTL (2153)
7	13186220	rs312536923	ENSGALG00000008738	Body weight QTL (2147)
7	13186220	rs312536923	ENSGALG00000008738	Body weight QTL (2148)
7	13186220	rs312536923	ENSGALG00000008738	Body weight QTL (2150)
7	13186220	rs312536923	ENSGALG00000008738	Body weight QTL (2146)
7	13186220	rs312536923	ENSGALG00000008738	Body weight QTL (2158)
7	13186220	rs312536923	ENSGALG00000008738	Body weight QTL (2160)
7	13186220	rs312536923	ENSGALG00000008738	Intestine length QTL (7166)
7	13186220	rs312536923	ENSGALG00000008738	Body weight (35 days) QTL (17308)
7	13186220	rs312536923	ENSGALG00000008738	Body weight (14 days) QTL (17305)
7	13186220	rs312536923	ENSGALG00000008738	Body weight (77 days) QTL (17314)
7	13186223	rs313809209	ENSGALG00000008738	Abdominal fat weight QTL (2140)
7	13186223	rs313809209	ENSGALG00000008738	Abdominal fat weight QTL (12629)
7	13186223	rs313809209	ENSGALG00000008738	Skin fat weight QTL (12639)
7	13186223	rs313809209	ENSGALG00000008738	Fat distribution QTL (12644)
7	13186223	rs313809209	ENSGALG00000008738	Drumstick and thigh muscle weight QTL (13397)
7	13186223	rs313809209	ENSGALG00000008738	Drumstick and thigh muscle weight QTL (13407)
7	13186223	rs313809209	ENSGALG00000008738	Body weight (112 days) QTL (6626)
7	13186223	rs313809209	ENSGALG00000008738	Body weight (200 days) QTL (6627)
7	13186223	rs313809209	ENSGALG00000008738	Body weight QTL (2136)
7	13186223	rs313809209	ENSGALG00000008738	Average daily gain QTL (24915)
7	13186223	rs313809209	ENSGALG00000008738	Wing weight QTL (2137)
7	13186223	rs313809209	ENSGALG00000008738	Thigh muscle weight QTL (2139)
7	13186223	rs313809209	ENSGALG00000008738	Thigh weight QTL (2141)
7	13186223	rs313809209	ENSGALG00000008738	Drumstick weight QTL (2142)
7	13186223	rs313809209	ENSGALG00000008738	Carcass weight QTL (2143)
7	13186223	rs313809209	ENSGALG00000008738	Drumstick muscle weight QTL (2144)
7	13186223	rs313809209	ENSGALG00000008738	Breast muscle weight QTL (2145)
7	13186223	rs313809209	ENSGALG00000008738	Body weight QTL (2149)
7	13186223	rs313809209	ENSGALG00000008738	Body weight QTL (2151)
7	13186223	rs313809209	ENSGALG00000008738	Body weight QTL (2153)
7	13186223	rs313809209	ENSGALG00000008738	Body weight QTL (2147)
7	13186223	rs313809209	ENSGALG00000008738	Body weight QTL (2148)
7	13186223	rs313809209	ENSGALG00000008738	Body weight QTL (2150)

7	13186223	rs313809209	ENSGALG00000008738	Body weight QTL (2146)
7	13186223	rs313809209	ENSGALG00000008738	Body weight QTL (2158)
7	13186223	rs313809209	ENSGALG00000008738	Body weight QTL (2160)
7	13186223	rs313809209	ENSGALG00000008738	Intestine length QTL (7166)
7	13186223	rs313809209	ENSGALG00000008738	Body weight (35 days) QTL (17308)
7	13186223	rs313809209	ENSGALG00000008738	Body weight (14 days) QTL (17305)
7	13186223	rs313809209	ENSGALG00000008738	Body weight (77 days) QTL (17314)
7	13186650	rs316285728	ENSGALG00000008738	Abdominal fat weight QTL (2140)
7	13186650	rs316285728	ENSGALG00000008738	Abdominal fat weight QTL (12629)
7	13186650	rs316285728	ENSGALG00000008738	Skin fat weight QTL (12639)
7	13186650	rs316285728	ENSGALG00000008738	Fat distribution QTL (12644)
7	13186650	rs316285728	ENSGALG00000008738	Drumstick and thigh muscle weight QTL (13397)
7	13186650	rs316285728	ENSGALG00000008738	Drumstick and thigh muscle weight QTL (13407)
7	13186650	rs316285728	ENSGALG00000008738	Body weight (112 days) QTL (6626)
7	13186650	rs316285728	ENSGALG00000008738	Body weight (200 days) QTL (6627)
7	13186650	rs316285728	ENSGALG00000008738	Body weight QTL (2136)
7	13186650	rs316285728	ENSGALG00000008738	Average daily gain QTL (24915)
7	13186650	rs316285728	ENSGALG00000008738	Wing weight QTL (2137)
7	13186650	rs316285728	ENSGALG00000008738	Thigh muscle weight QTL (2139)
7	13186650	rs316285728	ENSGALG00000008738	Thigh weight QTL (2141)
7	13186650	rs316285728	ENSGALG00000008738	Drumstick weight QTL (2142)
7	13186650	rs316285728	ENSGALG00000008738	Carcass weight QTL (2143)
7	13186650	rs316285728	ENSGALG00000008738	Drumstick muscle weight QTL (2144)
7	13186650	rs316285728	ENSGALG00000008738	Breast muscle weight QTL (2145)
7	13186650	rs316285728	ENSGALG00000008738	Body weight QTL (2149)
7	13186650	rs316285728	ENSGALG00000008738	Body weight QTL (2151)
7	13186650	rs316285728	ENSGALG00000008738	Body weight QTL (2153)
7	13186650	rs316285728	ENSGALG00000008738	Body weight QTL (2147)
7	13186650	rs316285728	ENSGALG00000008738	Body weight QTL (2148)
7	13186650	rs316285728	ENSGALG00000008738	Body weight QTL (2150)
7	13186650	rs316285728	ENSGALG00000008738	Body weight QTL (2146)
7	13186650	rs316285728	ENSGALG00000008738	Body weight QTL (2158)
7	13186650	rs316285728	ENSGALG00000008738	Body weight QTL (2160)
7	13186650	rs316285728	ENSGALG00000008738	Intestine length QTL (7166)
7	13186650	rs316285728	ENSGALG00000008738	Body weight (35 days) QTL (17308)
7	13186650	rs316285728	ENSGALG00000008738	Body weight (14 days) QTL (17305)
7	13186650	rs316285728	ENSGALG00000008738	Body weight (77 days) QTL (17314)
7	13187016	rs737034318	ENSGALG00000008738	Abdominal fat weight QTL (2140)
7	13187016	rs737034318	ENSGALG00000008738	Abdominal fat weight QTL (12629)
7	13187016	rs737034318	ENSGALG00000008738	Skin fat weight QTL (12639)

7	13187016	rs737034318	ENSGALG00000008738	Fat distribution QTL (12644)
7	13187016	rs737034318	ENSGALG00000008738	Drumstick and thigh muscle weight QTL (13397)
7	13187016	rs737034318	ENSGALG00000008738	Drumstick and thigh muscle weight QTL (13407)
7	13187016	rs737034318	ENSGALG00000008738	Body weight (112 days) QTL (6626)
7	13187016	rs737034318	ENSGALG00000008738	Body weight (200 days) QTL (6627)
7	13187016	rs737034318	ENSGALG00000008738	Body weight QTL (2136)
7	13187016	rs737034318	ENSGALG00000008738	Average daily gain QTL (24915)
7	13187016	rs737034318	ENSGALG00000008738	Wing weight QTL (2137)
7	13187016	rs737034318	ENSGALG00000008738	Thigh muscle weight QTL (2139)
7	13187016	rs737034318	ENSGALG00000008738	Thigh weight QTL (2141)
7	13187016	rs737034318	ENSGALG00000008738	Drumstick weight QTL (2142)
7	13187016	rs737034318	ENSGALG00000008738	Carcass weight QTL (2143)
7	13187016	rs737034318	ENSGALG00000008738	Drumstick muscle weight QTL (2144)
7	13187016	rs737034318	ENSGALG00000008738	Breast muscle weight QTL (2145)
7	13187016	rs737034318	ENSGALG00000008738	Body weight QTL (2149)
7	13187016	rs737034318	ENSGALG00000008738	Body weight QTL (2151)
7	13187016	rs737034318	ENSGALG00000008738	Body weight QTL (2153)
7	13187016	rs737034318	ENSGALG00000008738	Body weight QTL (2147)
7	13187016	rs737034318	ENSGALG00000008738	Body weight QTL (2148)
7	13187016	rs737034318	ENSGALG00000008738	Body weight QTL (2150)
7	13187016	rs737034318	ENSGALG00000008738	Body weight QTL (2146)
7	13187016	rs737034318	ENSGALG00000008738	Body weight QTL (2158)
7	13187016	rs737034318	ENSGALG00000008738	Body weight QTL (2160)
7	13187016	rs737034318	ENSGALG00000008738	Intestine length QTL (7166)
7	13187016	rs737034318	ENSGALG00000008738	Body weight (35 days) QTL (17308)
7	13187016	rs737034318	ENSGALG00000008738	Body weight (14 days) QTL (17305)
7	13187016	rs737034318	ENSGALG00000008738	Body weight (77 days) QTL (17314)
7	13187020	rs315868610	ENSGALG00000008738	Abdominal fat weight QTL (2140)
7	13187020	rs315868610	ENSGALG00000008738	Abdominal fat weight QTL (12629)
7	13187020	rs315868610	ENSGALG00000008738	Skin fat weight QTL (12639)
7	13187020	rs315868610	ENSGALG00000008738	Fat distribution QTL (12644)
7	13187020	rs315868610	ENSGALG00000008738	Drumstick and thigh muscle weight QTL (13397)
7	13187020	rs315868610	ENSGALG00000008738	Drumstick and thigh muscle weight QTL (13407)
7	13187020	rs315868610	ENSGALG00000008738	Body weight (112 days) QTL (6626)
7	13187020	rs315868610	ENSGALG00000008738	Body weight (200 days) QTL (6627)
7	13187020	rs315868610	ENSGALG00000008738	Body weight QTL (2136)
7	13187020	rs315868610	ENSGALG00000008738	Average daily gain QTL (24915)
7	13187020	rs315868610	ENSGALG00000008738	Wing weight QTL (2137)
7	13187020	rs315868610	ENSGALG00000008738	Thigh muscle weight QTL (2139)

7	13187020	rs315868610	ENSGALG00000008738	Thigh weight QTL (2141)
7	13187020	rs315868610	ENSGALG00000008738	Drumstick weight QTL (2142)
7	13187020	rs315868610	ENSGALG00000008738	Carcass weight QTL (2143)
7	13187020	rs315868610	ENSGALG00000008738	Drumstick muscle weight QTL (2144)
7	13187020	rs315868610	ENSGALG00000008738	Breast muscle weight QTL (2145)
7	13187020	rs315868610	ENSGALG00000008738	Body weight QTL (2149)
7	13187020	rs315868610	ENSGALG00000008738	Body weight QTL (2151)
7	13187020	rs315868610	ENSGALG00000008738	Body weight QTL (2153)
7	13187020	rs315868610	ENSGALG00000008738	Body weight QTL (2147)
7	13187020	rs315868610	ENSGALG00000008738	Body weight QTL (2148)
7	13187020	rs315868610	ENSGALG00000008738	Body weight QTL (2150)
7	13187020	rs315868610	ENSGALG00000008738	Body weight QTL (2146)
7	13187020	rs315868610	ENSGALG00000008738	Body weight QTL (2158)
7	13187020	rs315868610	ENSGALG00000008738	Body weight QTL (2160)
7	13187020	rs315868610	ENSGALG00000008738	Intestine length QTL (7166)
7	13187020	rs315868610	ENSGALG00000008738	Body weight (35 days) QTL (17308)
7	13187020	rs315868610	ENSGALG00000008738	Body weight (14 days) QTL (17305)
7	13187020	rs315868610	ENSGALG00000008738	Body weight (77 days) QTL (17314)
7	13187021	rs732851317	ENSGALG00000008738	Abdominal fat weight QTL (2140)
7	13187021	rs732851317	ENSGALG00000008738	Abdominal fat weight QTL (12629)
7	13187021	rs732851317	ENSGALG00000008738	Skin fat weight QTL (12639)
7	13187021	rs732851317	ENSGALG00000008738	Fat distribution QTL (12644)
7	13187021	rs732851317	ENSGALG00000008738	Drumstick and thigh muscle weight QTL (13397)
7	13187021	rs732851317	ENSGALG00000008738	Drumstick and thigh muscle weight QTL (13407)
7	13187021	rs732851317	ENSGALG00000008738	Body weight (112 days) QTL (6626)
7	13187021	rs732851317	ENSGALG00000008738	Body weight (200 days) QTL (6627)
7	13187021	rs732851317	ENSGALG00000008738	Body weight QTL (2136)
7	13187021	rs732851317	ENSGALG00000008738	Average daily gain QTL (24915)
7	13187021	rs732851317	ENSGALG00000008738	Wing weight QTL (2137)
7	13187021	rs732851317	ENSGALG00000008738	Thigh muscle weight QTL (2139)
7	13187021	rs732851317	ENSGALG00000008738	Thigh weight QTL (2141)
7	13187021	rs732851317	ENSGALG00000008738	Drumstick weight QTL (2142)
7	13187021	rs732851317	ENSGALG00000008738	Carcass weight QTL (2143)
7	13187021	rs732851317	ENSGALG00000008738	Drumstick muscle weight QTL (2144)
7	13187021	rs732851317	ENSGALG00000008738	Breast muscle weight QTL (2145)
7	13187021	rs732851317	ENSGALG00000008738	Body weight QTL (2149)
7	13187021	rs732851317	ENSGALG00000008738	Body weight QTL (2151)
7	13187021	rs732851317	ENSGALG00000008738	Body weight QTL (2153)
7	13187021	rs732851317	ENSGALG00000008738	Body weight QTL (2147)
7	13187021	rs732851317	ENSGALG00000008738	Body weight QTL (2148)

7	13187021	rs732851317	ENSGALG00000008738	Body weight QTL (2150)
7	13187021	rs732851317	ENSGALG00000008738	Body weight QTL (2146)
7	13187021	rs732851317	ENSGALG00000008738	Body weight QTL (2158)
7	13187021	rs732851317	ENSGALG00000008738	Body weight QTL (2160)
7	13187021	rs732851317	ENSGALG00000008738	Intestine length QTL (7166)
7	13187021	rs732851317	ENSGALG00000008738	Body weight (35 days) QTL (17308)
7	13187021	rs732851317	ENSGALG00000008738	Body weight (14 days) QTL (17305)
7	13187021	rs732851317	ENSGALG00000008738	Body weight (77 days) QTL (17314)
7	13187051	rs736429536	ENSGALG00000008738	Abdominal fat weight QTL (2140)
7	13187051	rs736429536	ENSGALG00000008738	Abdominal fat weight QTL (12629)
7	13187051	rs736429536	ENSGALG00000008738	Skin fat weight QTL (12639)
7	13187051	rs736429536	ENSGALG00000008738	Fat distribution QTL (12644)
7	13187051	rs736429536	ENSGALG00000008738	Drumstick and thigh muscle weight QTL (13397)
7	13187051	rs736429536	ENSGALG00000008738	Drumstick and thigh muscle weight QTL (13407)
7	13187051	rs736429536	ENSGALG00000008738	Body weight (112 days) QTL (6626)
7	13187051	rs736429536	ENSGALG00000008738	Body weight (200 days) QTL (6627)
7	13187051	rs736429536	ENSGALG00000008738	Body weight QTL (2136)
7	13187051	rs736429536	ENSGALG00000008738	Average daily gain QTL (24915)
7	13187051	rs736429536	ENSGALG00000008738	Wing weight QTL (2137)
7	13187051	rs736429536	ENSGALG00000008738	Thigh muscle weight QTL (2139)
7	13187051	rs736429536	ENSGALG00000008738	Thigh weight QTL (2141)
7	13187051	rs736429536	ENSGALG00000008738	Drumstick weight QTL (2142)
7	13187051	rs736429536	ENSGALG00000008738	Carcass weight QTL (2143)
7	13187051	rs736429536	ENSGALG00000008738	Drumstick muscle weight QTL (2144)
7	13187051	rs736429536	ENSGALG00000008738	Breast muscle weight QTL (2145)
7	13187051	rs736429536	ENSGALG00000008738	Body weight QTL (2149)
7	13187051	rs736429536	ENSGALG00000008738	Body weight QTL (2151)
7	13187051	rs736429536	ENSGALG00000008738	Body weight QTL (2153)
7	13187051	rs736429536	ENSGALG00000008738	Body weight QTL (2147)
7	13187051	rs736429536	ENSGALG00000008738	Body weight QTL (2148)
7	13187051	rs736429536	ENSGALG00000008738	Body weight QTL (2150)
7	13187051	rs736429536	ENSGALG00000008738	Body weight QTL (2146)
7	13187051	rs736429536	ENSGALG00000008738	Body weight QTL (2158)
7	13187051	rs736429536	ENSGALG00000008738	Body weight QTL (2160)
7	13187051	rs736429536	ENSGALG00000008738	Intestine length QTL (7166)
7	13187051	rs736429536	ENSGALG00000008738	Body weight (35 days) QTL (17308)
7	13187051	rs736429536	ENSGALG00000008738	Body weight (14 days) QTL (17305)
7	13187051	rs736429536	ENSGALG00000008738	Body weight (77 days) QTL (17314)
13	12204487	rs741384794	G3BP1	Body weight (63 days) QTL (9444)
13	12204487	rs741384794	G3BP1	Shank length QTL (9445)

13	12204487	rs741384794	G3BP1	Glucose level QTL (9446)
13	12204487	rs741384794	G3BP1	Breast muscle percentage QTL (9447)
13	12204487	rs741384794	G3BP1	Shank diameter QTL (9448)
13	12204487	rs741384794	G3BP1	Femur bending strength QTL (6774)
13	12204487	rs741384794	G3BP1	Femur bending strength QTL (6775)
13	12204487	rs741384794	G3BP1	Body weight (46 days) QTL (6645)
13	12204487	rs741384794	G3BP1	Growth (1-8 days) QTL (6646)
13	12204487	rs741384794	G3BP1	Thigh muscle weight QTL (6741)
13	12204487	rs741384794	G3BP1	Body weight (84 days) QTL (24870)
13	12204487	rs741384794	G3BP1	Body weight (42 days) QTL (24859)
13	12204487	rs741384794	G3BP1	Age at first egg QTL (14446)
13	12204487	rs741384794	G3BP1	Age at first egg QTL (14454)
13	12204487	rs741384794	G3BP1	Body weight (day of first egg) QTL (14472)
13	12204487	rs741384794	G3BP1	Average daily gain QTL (24902)
13	12204487	rs741384794	G3BP1	Drumstick and thigh weight QTL (17103)
13	12204487	rs741384794	G3BP1	Breast muscle weight QTL (17094)
13	12204487	rs741384794	G3BP1	Carcass weight QTL (17115)
13	12204487	rs741384794	G3BP1	Tibia length QTL (2309)
13	12204487	rs741384794	G3BP1	Body weight QTL (2310)
13	12204487	rs741384794	G3BP1	Abdominal fat weight QTL (12630)
13	12204487	rs741384794	G3BP1	Skin fat weight QTL (12640)
13	12204487	rs741384794	G3BP1	Breast muscle weight QTL (13390)
13	12204487	rs741384794	G3BP1	Drumstick muscle weight QTL (13393)
13	12204487	rs741384794	G3BP1	Drumstick and thigh muscle weight QTL (13398)
13	12204487	rs741384794	G3BP1	Drumstick and thigh muscle weight QTL (13409)
13	12204487	rs741384794	G3BP1	Body weight (21 days) QTL (9769)
13	12204487	rs741384794	G3BP1	Body weight (42 days) QTL (9770)
13	12204487	rs741384794	G3BP1	Body weight (63 days) QTL (9771)
13	12204487	rs741384794	G3BP1	Growth (21-42 days) QTL (9772)
13	12204487	rs741384794	G3BP1	Skin fat weight QTL (2313)
13	12204487	rs741384794	G3BP1	Drumstick weight QTL (2319)
13	12204487	rs741384794	G3BP1	Drumstick muscle weight QTL (2320)
13	12204487	rs741384794	G3BP1	Heart weight QTL (2316)
13	12204487	rs741384794	G3BP1	Thigh weight QTL (2318)
13	12204487	rs741384794	G3BP1	Breast muscle weight QTL (2317)
13	12204487	rs741384794	G3BP1	Carcass weight QTL (17089)
13	12231794	rs315635567	G3BP1	Body weight (63 days) QTL (9444)
13	12231794	rs315635567	G3BP1	Shank length QTL (9445)
13	12231794	rs315635567	G3BP1	Glucose level QTL (9446)
13	12231794	rs315635567	G3BP1	Breast muscle percentage QTL (9447)

13	12231794	rs315635567	G3BP1	Shank diameter QTL (9448)
13	12231794	rs315635567	G3BP1	Femur bending strength QTL (6774)
13	12231794	rs315635567	G3BP1	Femur bending strength QTL (6775)
13	12231794	rs315635567	G3BP1	Body weight (46 days) QTL (6645)
13	12231794	rs315635567	G3BP1	Growth (1-8 days) QTL (6646)
13	12231794	rs315635567	G3BP1	Thigh muscle weight QTL (6741)
13	12231794	rs315635567	G3BP1	Body weight (84 days) QTL (24870)
13	12231794	rs315635567	G3BP1	Body weight (42 days) QTL (24859)
13	12231794	rs315635567	G3BP1	Age at first egg QTL (14446)
13	12231794	rs315635567	G3BP1	Age at first egg QTL (14454)
13	12231794	rs315635567	G3BP1	Body weight (day of first egg) QTL (14472)
13	12231794	rs315635567	G3BP1	Average daily gain QTL (24902)
13	12231794	rs315635567	G3BP1	Drumstick and thigh weight QTL (17103)
13	12231794	rs315635567	G3BP1	Breast muscle weight QTL (17094)
13	12231794	rs315635567	G3BP1	Carcass weight QTL (17115)
13	12231794	rs315635567	G3BP1	Tibia length QTL (2309)
13	12231794	rs315635567	G3BP1	Body weight QTL (2310)
13	12231794	rs315635567	G3BP1	Abdominal fat weight QTL (12630)
13	12231794	rs315635567	G3BP1	Skin fat weight QTL (12640)
13	12231794	rs315635567	G3BP1	Breast muscle weight QTL (13390)
13	12231794	rs315635567	G3BP1	Drumstick muscle weight QTL (13393)
13	12231794	rs315635567	G3BP1	Drumstick and thigh muscle weight QTL (13398)
13	12231794	rs315635567	G3BP1	Drumstick and thigh muscle weight QTL (13409)
13	12231794	rs315635567	G3BP1	Body weight (21 days) QTL (9769)
13	12231794	rs315635567	G3BP1	Body weight (42 days) QTL (9770)
13	12231794	rs315635567	G3BP1	Body weight (63 days) QTL (9771)
13	12231794	rs315635567	G3BP1	Growth (21-42 days) QTL (9772)
13	12231794	rs315635567	G3BP1	Skin fat weight QTL (2313)
13	12231794	rs315635567	G3BP1	Drumstick weight QTL (2319)
13	12231794	rs315635567	G3BP1	Drumstick muscle weight QTL (2320)
13	12231794	rs315635567	G3BP1	Heart weight QTL (2316)
13	12231794	rs315635567	G3BP1	Thigh weight QTL (2318)
13	12231794	rs315635567	G3BP1	Breast muscle weight QTL (2317)
13	12231794	rs315635567	G3BP1	Carcass weight QTL (17089)

Supplementary Table 25: Enriched QTL traits from the significant eQTLs of Cecum of low and high FCR broilers

chr	pos	snp_id	Gene	QTL_id
3	24809972	rs313943112	PPM1B	Abdominal fat percentage QTL (9417)
3	24809972	rs313943112	PPM1B	Abdominal fat weight QTL (9418)
3	24809972	rs313943112	PPM1B	Breast muscle percentage QTL (9416)
3	24809972	rs313943112	PPM1B	Age at first egg QTL (14445)
3	24809972	rs313943112	PPM1B	Age at first egg QTL (14451)
3	24809972	rs313943112	PPM1B	Bone mineral density QTL (6765)
3	24809972	rs313943112	PPM1B	Bone mineral density QTL (6766)
3	24809972	rs313943112	PPM1B	Abdominal fat weight QTL (1947)
3	24809972	rs313943112	PPM1B	Body weight (84 days) QTL (24864)
3	24809972	rs313943112	PPM1B	Body weight (336 days) QTL (24881)
3	24809972	rs313943112	PPM1B	Body weight (504 days) QTL (24889)
3	24809972	rs313943112	PPM1B	Body weight (42 days) QTL (24852)
3	24809972	rs313943112	PPM1B	Average daily gain QTL (24896)
3	24809972	rs313943112	PPM1B	Age at sexual maturity QTL (16808)
3	24809972	rs313943112	PPM1B	Bone mineral density QTL (9376)
3	24809972	rs313943112	PPM1B	Body weight QTL (1951)
3	24809972	rs313943112	PPM1B	Tibia bone mineral density QTL (1950)
3	24809972	rs313943112	PPM1B	Head percentage QTL (15598)
3	24809972	rs313943112	PPM1B	Body weight (1 day) QTL (7167)
3	24809972	rs313943112	PPM1B	Body weight (35 days) QTL (7171)
3	24809972	rs313943112	PPM1B	Body weight (41 days) QTL (7174)
3	24809972	rs313943112	PPM1B	Pectoralis major weight QTL (1954)
3	24809972	rs313943112	PPM1B	Cholesterol level QTL (1955)
3	24809972	rs313943112	PPM1B	Abdominal fat weight QTL (1958)
3	24809972	rs313943112	PPM1B	Body weight QTL (1979)
3	24809972	rs313943112	PPM1B	Body weight QTL (1980)
3	24809972	rs313943112	PPM1B	Body weight (35 days) QTL (7180)
3	24809972	rs313943112	PPM1B	Body weight (35 days) QTL (55904)
3	24809972	rs313943112	PPM1B	Growth (0-35 days) QTL (55929)
3	24809972	rs313943112	PPM1B	Body weight QTL (1957)
3	24811608	rs16240336	PPM1B	Abdominal fat percentage QTL (9417)
3	24811608	rs16240336	PPM1B	Abdominal fat weight QTL (9418)
3	24811608	rs16240336	PPM1B	Breast muscle percentage QTL (9416)
3	24811608	rs16240336	PPM1B	Age at first egg QTL (14445)
3	24811608	rs16240336	PPM1B	Age at first egg QTL (14451)
3	24811608	rs16240336	PPM1B	Bone mineral density QTL (6765)
3	24811608	rs16240336	PPM1B	Bone mineral density QTL (6766)
3	24811608	rs16240336	PPM1B	Abdominal fat weight QTL (1947)

3	24811608	rs16240336	PPM1B	Body weight (84 days) QTL (24864)
3	24811608	rs16240336	PPM1B	Body weight (336 days) QTL (24881)
3	24811608	rs16240336	PPM1B	Body weight (504 days) QTL (24889)
3	24811608	rs16240336	PPM1B	Body weight (42 days) QTL (24852)
3	24811608	rs16240336	PPM1B	Average daily gain QTL (24896)
3	24811608	rs16240336	PPM1B	Age at sexual maturity QTL (16808)
3	24811608	rs16240336	PPM1B	Bone mineral density QTL (9376)
3	24811608	rs16240336	PPM1B	Body weight QTL (1951)
3	24811608	rs16240336	PPM1B	Tibia bone mineral density QTL (1950)
3	24811608	rs16240336	PPM1B	Head percentage QTL (15598)
3	24811608	rs16240336	PPM1B	Body weight (1 day) QTL (7167)
3	24811608	rs16240336	PPM1B	Body weight (35 days) QTL (7171)
3	24811608	rs16240336	PPM1B	Body weight (41 days) QTL (7174)
3	24811608	rs16240336	PPM1B	Pectoralis major weight QTL (1954)
3	24811608	rs16240336	PPM1B	Cholesterol level QTL (1955)
3	24811608	rs16240336	PPM1B	Abdominal fat weight QTL (1958)
3	24811608	rs16240336	PPM1B	Body weight QTL (1979)
3	24811608	rs16240336	PPM1B	Body weight QTL (1980)
3	24811608	rs16240336	PPM1B	Body weight (35 days) QTL (7180)
3	24811608	rs16240336	PPM1B	Body weight (35 days) QTL (55904)
3	24811608	rs16240336	PPM1B	Growth (0-35 days) QTL (55929)
3	24811608	rs16240336	PPM1B	Body weight QTL (1957)
3	24811640	rs317341558	PPM1B	Abdominal fat percentage QTL (9417)
3	24811640	rs317341558	PPM1B	Abdominal fat weight QTL (9418)
3	24811640	rs317341558	PPM1B	Breast muscle percentage QTL (9416)
3	24811640	rs317341558	PPM1B	Age at first egg QTL (14445)
3	24811640	rs317341558	PPM1B	Age at first egg QTL (14451)
3	24811640	rs317341558	PPM1B	Bone mineral density QTL (6765)
3	24811640	rs317341558	PPM1B	Bone mineral density QTL (6766)
3	24811640	rs317341558	PPM1B	Abdominal fat weight QTL (1947)
3	24811640	rs317341558	PPM1B	Body weight (84 days) QTL (24864)
3	24811640	rs317341558	PPM1B	Body weight (336 days) QTL (24881)
3	24811640	rs317341558	PPM1B	Body weight (504 days) QTL (24889)
3	24811640	rs317341558	PPM1B	Body weight (42 days) QTL (24852)
3	24811640	rs317341558	PPM1B	Average daily gain QTL (24896)
3	24811640	rs317341558	PPM1B	Age at sexual maturity QTL (16808)
3	24811640	rs317341558	PPM1B	Bone mineral density QTL (9376)
3	24811640	rs317341558	PPM1B	Body weight QTL (1951)
3	24811640	rs317341558	PPM1B	Tibia bone mineral density QTL (1950)
3	24811640	rs317341558	PPM1B	Head percentage QTL (15598)
3	24811640	rs317341558	PPM1B	Body weight (1 day) QTL (7167)

3	24811640	rs317341558	PPM1B	Body weight (35 days) QTL (7171)
3	24811640	rs317341558	PPM1B	Body weight (41 days) QTL (7174)
3	24811640	rs317341558	PPM1B	Pectoralis major weight QTL (1954)
3	24811640	rs317341558	PPM1B	Cholesterol level QTL (1955)
3	24811640	rs317341558	PPM1B	Abdominal fat weight QTL (1958)
3	24811640	rs317341558	PPM1B	Body weight QTL (1979)
3	24811640	rs317341558	PPM1B	Body weight QTL (1980)
3	24811640	rs317341558	PPM1B	Body weight (35 days) QTL (7180)
3	24811640	rs317341558	PPM1B	Body weight (35 days) QTL (55904)
3	24811640	rs317341558	PPM1B	Growth (0-35 days) QTL (55929)
3	24811640	rs317341558	PPM1B	Body weight QTL (1957)
3	24811659	rs737292718	PPM1B	Abdominal fat percentage QTL (9417)
3	24811659	rs737292718	PPM1B	Abdominal fat weight QTL (9418)
3	24811659	rs737292718	PPM1B	Breast muscle percentage QTL (9416)
3	24811659	rs737292718	PPM1B	Age at first egg QTL (14445)
3	24811659	rs737292718	PPM1B	Age at first egg QTL (14451)
3	24811659	rs737292718	PPM1B	Bone mineral density QTL (6765)
3	24811659	rs737292718	PPM1B	Bone mineral density QTL (6766)
3	24811659	rs737292718	PPM1B	Abdominal fat weight QTL (1947)
3	24811659	rs737292718	PPM1B	Body weight (84 days) QTL (24864)
3	24811659	rs737292718	PPM1B	Body weight (336 days) QTL (24881)
3	24811659	rs737292718	PPM1B	Body weight (504 days) QTL (24889)
3	24811659	rs737292718	PPM1B	Body weight (42 days) QTL (24852)
3	24811659	rs737292718	PPM1B	Average daily gain QTL (24896)
3	24811659	rs737292718	PPM1B	Age at sexual maturity QTL (16808)
3	24811659	rs737292718	PPM1B	Bone mineral density QTL (9376)
3	24811659	rs737292718	PPM1B	Body weight QTL (1951)
3	24811659	rs737292718	PPM1B	Tibia bone mineral density QTL (1950)
3	24811659	rs737292718	PPM1B	Head percentage QTL (15598)
3	24811659	rs737292718	PPM1B	Body weight (1 day) QTL (7167)
3	24811659	rs737292718	PPM1B	Body weight (35 days) QTL (7171)
3	24811659	rs737292718	PPM1B	Body weight (41 days) QTL (7174)
3	24811659	rs737292718	PPM1B	Pectoralis major weight QTL (1954)
3	24811659	rs737292718	PPM1B	Cholesterol level QTL (1955)
3	24811659	rs737292718	PPM1B	Abdominal fat weight QTL (1958)
3	24811659	rs737292718	PPM1B	Body weight QTL (1979)
3	24811659	rs737292718	PPM1B	Body weight QTL (1980)
3	24811659	rs737292718	PPM1B	Body weight (35 days) QTL (7180)
3	24811659	rs737292718	PPM1B	Body weight (35 days) QTL (55904)
3	24811659	rs737292718	PPM1B	Growth (0-35 days) QTL (55929)
3	24811659	rs737292718	PPM1B	Body weight QTL (1957)

3	24811662	rs740679964	PPM1B	Abdominal fat percentage QTL (9417)
3	24811662	rs740679964	PPM1B	Abdominal fat weight QTL (9418)
3	24811662	rs740679964	PPM1B	Breast muscle percentage QTL (9416)
3	24811662	rs740679964	PPM1B	Age at first egg QTL (14445)
3	24811662	rs740679964	PPM1B	Age at first egg QTL (14451)
3	24811662	rs740679964	PPM1B	Bone mineral density QTL (6765)
3	24811662	rs740679964	PPM1B	Bone mineral density QTL (6766)
3	24811662	rs740679964	PPM1B	Abdominal fat weight QTL (1947)
3	24811662	rs740679964	PPM1B	Body weight (84 days) QTL (24864)
3	24811662	rs740679964	PPM1B	Body weight (336 days) QTL (24881)
3	24811662	rs740679964	PPM1B	Body weight (504 days) QTL (24889)
3	24811662	rs740679964	PPM1B	Body weight (42 days) QTL (24852)
3	24811662	rs740679964	PPM1B	Average daily gain QTL (24896)
3	24811662	rs740679964	PPM1B	Age at sexual maturity QTL (16808)
3	24811662	rs740679964	PPM1B	Bone mineral density QTL (9376)
3	24811662	rs740679964	PPM1B	Body weight QTL (1951)
3	24811662	rs740679964	PPM1B	Tibia bone mineral density QTL (1950)
3	24811662	rs740679964	PPM1B	Head percentage QTL (15598)
3	24811662	rs740679964	PPM1B	Body weight (1 day) QTL (7167)
3	24811662	rs740679964	PPM1B	Body weight (35 days) QTL (7171)
3	24811662	rs740679964	PPM1B	Body weight (41 days) QTL (7174)
3	24811662	rs740679964	PPM1B	Pectoralis major weight QTL (1954)
3	24811662	rs740679964	PPM1B	Cholesterol level QTL (1955)
3	24811662	rs740679964	PPM1B	Abdominal fat weight QTL (1958)
3	24811662	rs740679964	PPM1B	Body weight QTL (1979)
3	24811662	rs740679964	PPM1B	Body weight QTL (1980)
3	24811662	rs740679964	PPM1B	Body weight (35 days) QTL (7180)
3	24811662	rs740679964	PPM1B	Body weight (35 days) QTL (55904)
3	24811662	rs740679964	PPM1B	Growth (0-35 days) QTL (55929)
3	24811662	rs740679964	PPM1B	Body weight QTL (1957)
3	24811667	rs732689598	PPM1B	Abdominal fat percentage QTL (9417)
3	24811667	rs732689598	PPM1B	Abdominal fat weight QTL (9418)
3	24811667	rs732689598	PPM1B	Breast muscle percentage QTL (9416)
3	24811667	rs732689598	PPM1B	Age at first egg QTL (14445)
3	24811667	rs732689598	PPM1B	Age at first egg QTL (14451)
3	24811667	rs732689598	PPM1B	Bone mineral density QTL (6765)
3	24811667	rs732689598	PPM1B	Bone mineral density QTL (6766)
3	24811667	rs732689598	PPM1B	Abdominal fat weight QTL (1947)
3	24811667	rs732689598	PPM1B	Body weight (84 days) QTL (24864)
3	24811667	rs732689598	PPM1B	Body weight (336 days) QTL (24881)
3	24811667	rs732689598	PPM1B	Body weight (504 days) QTL (24889)

3	24811667	rs732689598	PPM1B	Body weight (42 days) QTL (24852)
3	24811667	rs732689598	PPM1B	Average daily gain QTL (24896)
3	24811667	rs732689598	PPM1B	Age at sexual maturity QTL (16808)
3	24811667	rs732689598	PPM1B	Bone mineral density QTL (9376)
3	24811667	rs732689598	PPM1B	Body weight QTL (1951)
3	24811667	rs732689598	PPM1B	Tibia bone mineral density QTL (1950)
3	24811667	rs732689598	PPM1B	Head percentage QTL (15598)
3	24811667	rs732689598	PPM1B	Body weight (1 day) QTL (7167)
3	24811667	rs732689598	PPM1B	Body weight (35 days) QTL (7171)
3	24811667	rs732689598	PPM1B	Body weight (41 days) QTL (7174)
3	24811667	rs732689598	PPM1B	Pectoralis major weight QTL (1954)
3	24811667	rs732689598	PPM1B	Cholesterol level QTL (1955)
3	24811667	rs732689598	PPM1B	Abdominal fat weight QTL (1958)
3	24811667	rs732689598	PPM1B	Body weight QTL (1979)
3	24811667	rs732689598	PPM1B	Body weight QTL (1980)
3	24811667	rs732689598	PPM1B	Body weight (35 days) QTL (7180)
3	24811667	rs732689598	PPM1B	Body weight (35 days) QTL (55904)
3	24811667	rs732689598	PPM1B	Growth (0-35 days) QTL (55929)
3	24811667	rs732689598	PPM1B	Body weight QTL (1957)
3	24812259	_Variant_638	PPM1B	Abdominal fat percentage QTL (9417)
3	24812259	_Variant_638	PPM1B	Abdominal fat weight QTL (9418)
3	24812259	_Variant_638	PPM1B	Breast muscle percentage QTL (9416)
3	24812259	_Variant_638	PPM1B	Age at first egg QTL (14445)
3	24812259	_Variant_638	PPM1B	Age at first egg QTL (14451)
3	24812259	_Variant_638	PPM1B	Bone mineral density QTL (6765)
3	24812259	_Variant_638	PPM1B	Bone mineral density QTL (6766)
3	24812259	_Variant_638	PPM1B	Abdominal fat weight QTL (1947)
3	24812259	_Variant_638	PPM1B	Body weight (84 days) QTL (24864)
3	24812259	_Variant_638	PPM1B	Body weight (336 days) QTL (24881)
3	24812259	_Variant_638	PPM1B	Body weight (504 days) QTL (24889)
3	24812259	_Variant_638	PPM1B	Body weight (42 days) QTL (24852)
3	24812259	_Variant_638	PPM1B	Average daily gain QTL (24896)
3	24812259	_Variant_638	PPM1B	Age at sexual maturity QTL (16808)
3	24812259	_Variant_638	PPM1B	Bone mineral density QTL (9376)
3	24812259	_Variant_638	PPM1B	Body weight QTL (1951)
3	24812259	_Variant_638	PPM1B	Tibia bone mineral density QTL (1950)
3	24812259	_Variant_638	PPM1B	Head percentage QTL (15598)
3	24812259	_Variant_638	PPM1B	Body weight (1 day) QTL (7167)
3	24812259	_Variant_638	PPM1B	Body weight (35 days) QTL (7171)
3	24812259	_Variant_638	PPM1B	Body weight (41 days) QTL (7174)
3	24812259	_Variant_638	PPM1B	Pectoralis major weight QTL (1954)

3	24812259	_Variant_638	PPM1B	Cholesterol level QTL (1955)
3	24812259	_Variant_638	PPM1B	Abdominal fat weight QTL (1958)
3	24812259	_Variant_638	PPM1B	Body weight QTL (1979)
3	24812259	_Variant_638	PPM1B	Body weight QTL (1980)
3	24812259	_Variant_638	PPM1B	Body weight (35 days) QTL (7180)
3	24812259	_Variant_638	PPM1B	Body weight (35 days) QTL (55904)
3	24812259	_Variant_638	PPM1B	Growth (0-35 days) QTL (55929)
3	24812259	_Variant_638	PPM1B	Body weight QTL (1957)
3	24816341	rs741067461	PPM1B	Abdominal fat percentage QTL (9417)
3	24816341	rs741067461	PPM1B	Abdominal fat weight QTL (9418)
3	24816341	rs741067461	PPM1B	Breast muscle percentage QTL (9416)
3	24816341	rs741067461	PPM1B	Age at first egg QTL (14445)
3	24816341	rs741067461	PPM1B	Age at first egg QTL (14451)
3	24816341	rs741067461	PPM1B	Bone mineral density QTL (6765)
3	24816341	rs741067461	PPM1B	Bone mineral density QTL (6766)
3	24816341	rs741067461	PPM1B	Abdominal fat weight QTL (1947)
3	24816341	rs741067461	PPM1B	Body weight (84 days) QTL (24864)
3	24816341	rs741067461	PPM1B	Body weight (336 days) QTL (24881)
3	24816341	rs741067461	PPM1B	Body weight (504 days) QTL (24889)
3	24816341	rs741067461	PPM1B	Body weight (42 days) QTL (24852)
3	24816341	rs741067461	PPM1B	Average daily gain QTL (24896)
3	24816341	rs741067461	PPM1B	Age at sexual maturity QTL (16808)
3	24816341	rs741067461	PPM1B	Bone mineral density QTL (9376)
3	24816341	rs741067461	PPM1B	Body weight QTL (1951)
3	24816341	rs741067461	PPM1B	Tibia bone mineral density QTL (1950)
3	24816341	rs741067461	PPM1B	Head percentage QTL (15598)
3	24816341	rs741067461	PPM1B	Body weight (1 day) QTL (7167)
3	24816341	rs741067461	PPM1B	Body weight (35 days) QTL (7171)
3	24816341	rs741067461	PPM1B	Body weight (41 days) QTL (7174)
3	24816341	rs741067461	PPM1B	Pectoralis major weight QTL (1954)
3	24816341	rs741067461	PPM1B	Cholesterol level QTL (1955)
3	24816341	rs741067461	PPM1B	Abdominal fat weight QTL (1958)
3	24816341	rs741067461	PPM1B	Body weight QTL (1979)
3	24816341	rs741067461	PPM1B	Body weight QTL (1980)
3	24816341	rs741067461	PPM1B	Body weight (35 days) QTL (7180)
3	24816341	rs741067461	PPM1B	Body weight (35 days) QTL (55904)
3	24816341	rs741067461	PPM1B	Growth (0-35 days) QTL (55929)
3	24816341	rs741067461	PPM1B	Body weight QTL (1957)
3	24819471	rs15301049	PPM1B	Abdominal fat percentage QTL (9417)
3	24819471	rs15301049	PPM1B	Abdominal fat weight QTL (9418)
3	24819471	rs15301049	PPM1B	Breast muscle percentage QTL (9416)

3	24819471	rs15301049	PPM1B	Age at first egg QTL (14445)
3	24819471	rs15301049	PPM1B	Age at first egg QTL (14451)
3	24819471	rs15301049	PPM1B	Bone mineral density QTL (6765)
3	24819471	rs15301049	PPM1B	Bone mineral density QTL (6766)
3	24819471	rs15301049	PPM1B	Abdominal fat weight QTL (1947)
3	24819471	rs15301049	PPM1B	Body weight (84 days) QTL (24864)
3	24819471	rs15301049	PPM1B	Body weight (336 days) QTL (24881)
3	24819471	rs15301049	PPM1B	Body weight (504 days) QTL (24889)
3	24819471	rs15301049	PPM1B	Body weight (42 days) QTL (24852)
3	24819471	rs15301049	PPM1B	Average daily gain QTL (24896)
3	24819471	rs15301049	PPM1B	Age at sexual maturity QTL (16808)
3	24819471	rs15301049	PPM1B	Bone mineral density QTL (9376)
3	24819471	rs15301049	PPM1B	Body weight QTL (1951)
3	24819471	rs15301049	PPM1B	Tibia bone mineral density QTL (1950)
3	24819471	rs15301049	PPM1B	Head percentage QTL (15598)
3	24819471	rs15301049	PPM1B	Body weight (1 day) QTL (7167)
3	24819471	rs15301049	PPM1B	Body weight (35 days) QTL (7171)
3	24819471	rs15301049	PPM1B	Body weight (41 days) QTL (7174)
3	24819471	rs15301049	PPM1B	Pectoralis major weight QTL (1954)
3	24819471	rs15301049	PPM1B	Cholesterol level QTL (1955)
3	24819471	rs15301049	PPM1B	Abdominal fat weight QTL (1958)
3	24819471	rs15301049	PPM1B	Body weight QTL (1979)
3	24819471	rs15301049	PPM1B	Body weight QTL (1980)
3	24819471	rs15301049	PPM1B	Body weight (35 days) QTL (7180)
3	24819471	rs15301049	PPM1B	Body weight (35 days) QTL (55904)
3	24819471	rs15301049	PPM1B	Growth (0-35 days) QTL (55929)
3	24819471	rs15301049	PPM1B	Body weight QTL (1957)
3	24822333	rs794597744	PPM1B	Abdominal fat percentage QTL (9417)
3	24822333	rs794597744	PPM1B	Abdominal fat weight QTL (9418)
3	24822333	rs794597744	PPM1B	Breast muscle percentage QTL (9416)
3	24822333	rs794597744	PPM1B	Age at first egg QTL (14445)
3	24822333	rs794597744	PPM1B	Age at first egg QTL (14451)
3	24822333	rs794597744	PPM1B	Bone mineral density QTL (6765)
3	24822333	rs794597744	PPM1B	Bone mineral density QTL (6766)
3	24822333	rs794597744	PPM1B	Abdominal fat weight QTL (1947)
3	24822333	rs794597744	PPM1B	Body weight (84 days) QTL (24864)
3	24822333	rs794597744	PPM1B	Body weight (336 days) QTL (24881)
3	24822333	rs794597744	PPM1B	Body weight (504 days) QTL (24889)
3	24822333	rs794597744	PPM1B	Body weight (42 days) QTL (24852)
3	24822333	rs794597744	PPM1B	Average daily gain QTL (24896)
3	24822333	rs794597744	PPM1B	Age at sexual maturity QTL (16808)

3	24822333	rs794597744	PPM1B	Bone mineral density QTL (9376)
3	24822333	rs794597744	PPM1B	Body weight QTL (1951)
3	24822333	rs794597744	PPM1B	Tibia bone mineral density QTL (1950)
3	24822333	rs794597744	PPM1B	Head percentage QTL (15598)
3	24822333	rs794597744	PPM1B	Body weight (1 day) QTL (7167)
3	24822333	rs794597744	PPM1B	Body weight (35 days) QTL (7171)
3	24822333	rs794597744	PPM1B	Body weight (41 days) QTL (7174)
3	24822333	rs794597744	PPM1B	Pectoralis major weight QTL (1954)
3	24822333	rs794597744	PPM1B	Cholesterol level QTL (1955)
3	24822333	rs794597744	PPM1B	Abdominal fat weight QTL (1958)
3	24822333	rs794597744	PPM1B	Body weight QTL (1979)
3	24822333	rs794597744	PPM1B	Body weight QTL (1980)
3	24822333	rs794597744	PPM1B	Body weight (35 days) QTL (7180)
3	24822333	rs794597744	PPM1B	Body weight (35 days) QTL (55904)
3	24822333	rs794597744	PPM1B	Growth (0-35 days) QTL (55929)
3	24822333	rs794597744	PPM1B	Body weight QTL (1957)
4	9330391	rs15489961	SH3BGRL	Body weight (56 days) QTL (9468)
4	9330391	rs15489961	SH3BGRL	Body weight (42 days) QTL (24854)
4	9330391	rs15489961	SH3BGRL	Body weight (84 days) QTL (24865)
4	9330391	rs15489961	SH3BGRL	Average daily gain QTL (24898)
4	9330391	rs15489961	SH3BGRL	Age at first egg QTL (14453)
4	9330391	rs15489961	SH3BGRL	Body weight QTL (1989)
4	9330391	rs15489961	SH3BGRL	Body weight QTL (1990)
4	9330391	rs15489961	SH3BGRL	Body weight QTL (1991)
4	9330391	rs15489961	SH3BGRL	Shank length QTL (11794)
4	9330391	rs15489961	SH3BGRL	Visceral fat weight QTL (17321)
5	14095079	_Variant_893	ENSGALG00000006740	Dressing percentage QTL (15578)
5	14095079	_Variant_893	ENSGALG00000006740	Breast percentage QTL (15581)
5	14095079	_Variant_893	ENSGALG00000006740	Spleen weight QTL (9959)
5	14095079	_Variant_893	ENSGALG00000006740	Yolk weight QTL (24972)
5	14095079	_Variant_893	ENSGALG00000006740	Albumen height QTL (36755)
5	14095079	_Variant_893	ENSGALG00000006740	Drumstick weight QTL (2064)
5	14095079	_Variant_893	ENSGALG00000006740	Conformation score QTL (2066)
5	14095079	_Variant_893	ENSGALG00000006740	Weight of the front half of the carcass QTL (2068)
5	14095079	_Variant_893	ENSGALG00000006740	Body weight (112 days) QTL (6616)
5	14095079	_Variant_893	ENSGALG00000006740	Body weight (200 days) QTL (6617)
5	14095079	_Variant_893	ENSGALG00000006740	Growth (46-112 days) QTL (6618)
5	14095079	_Variant_893	ENSGALG00000006740	Body weight QTL (2071)
5	14095079	_Variant_893	ENSGALG00000006740	Shank diameter QTL (9429)
5	14095079	_Variant_893	ENSGALG00000006740	Antibody response to SRBC antigen QTL (2075)

5	14095079	_Variant_893	ENSGALG00000006740	Shank length QTL (9428)
5	14095079	_Variant_893	ENSGALG00000006740	Egg number QTL (17070)
5	14095079	_Variant_893	ENSGALG00000006740	Head width QTL (16720)
5	14095079	_Variant_893	ENSGALG00000006740	Thigh muscle weight QTL (9397)
5	14095079	_Variant_893	ENSGALG00000006740	Shank weight QTL (9398)
5	14095079	_Variant_893	ENSGALG00000006740	Carcass weight QTL (17112)
5	14095079	_Variant_893	ENSGALG00000006740	Growth (70-105 days) QTL (55939)
7	13826038	rs733814084	ITGA4	Abdominal fat weight QTL (2140)
7	13826038	rs733814084	ITGA4	Abdominal fat weight QTL (12629)
7	13826038	rs733814084	ITGA4	Skin fat weight QTL (12639)
7	13826038	rs733814084	ITGA4	Fat distribution QTL (12644)
7	13826038	rs733814084	ITGA4	Drumstick and thigh muscle weight QTL (13397)
7	13826038	rs733814084	ITGA4	Drumstick and thigh muscle weight QTL (13407)
7	13826038	rs733814084	ITGA4	Body weight (112 days) QTL (6626)
7	13826038	rs733814084	ITGA4	Body weight (200 days) QTL (6627)
7	13826038	rs733814084	ITGA4	Body weight QTL (2136)
7	13826038	rs733814084	ITGA4	Average daily gain QTL (24915)
7	13826038	rs733814084	ITGA4	Wing weight QTL (2137)
7	13826038	rs733814084	ITGA4	Thigh muscle weight QTL (2139)
7	13826038	rs733814084	ITGA4	Thigh weight QTL (2141)
7	13826038	rs733814084	ITGA4	Drumstick weight QTL (2142)
7	13826038	rs733814084	ITGA4	Carcass weight QTL (2143)
7	13826038	rs733814084	ITGA4	Drumstick muscle weight QTL (2144)
7	13826038	rs733814084	ITGA4	Breast muscle weight QTL (2145)
7	13826038	rs733814084	ITGA4	Body weight QTL (2149)
7	13826038	rs733814084	ITGA4	Body weight QTL (2151)
7	13826038	rs733814084	ITGA4	Body weight QTL (2153)
7	13826038	rs733814084	ITGA4	Body weight QTL (2147)
7	13826038	rs733814084	ITGA4	Body weight QTL (2148)
7	13826038	rs733814084	ITGA4	Body weight QTL (2150)
7	13826038	rs733814084	ITGA4	Body weight QTL (2146)
7	13826038	rs733814084	ITGA4	Body weight QTL (2158)
7	13826038	rs733814084	ITGA4	Body weight QTL (2160)
7	13826038	rs733814084	ITGA4	Intestine length QTL (7166)
7	13826038	rs733814084	ITGA4	Body weight (35 days) QTL (17308)
7	13826038	rs733814084	ITGA4	Body weight (14 days) QTL (17305)
7	13826038	rs733814084	ITGA4	Body weight (77 days) QTL (17314)
7	13826068	rs732496374	ITGA4	Abdominal fat weight QTL (2140)
7	13826068	rs732496374	ITGA4	Abdominal fat weight QTL (12629)
7	13826068	rs732496374	ITGA4	Skin fat weight QTL (12639)

7	13826068	rs732496374	ITGA4	Fat distribution QTL (12644)
7	13826068	rs732496374	ITGA4	Drumstick and thigh muscle weight QTL (13397)
7	13826068	rs732496374	ITGA4	Drumstick and thigh muscle weight QTL (13407)
7	13826068	rs732496374	ITGA4	Body weight (112 days) QTL (6626)
7	13826068	rs732496374	ITGA4	Body weight (200 days) QTL (6627)
7	13826068	rs732496374	ITGA4	Body weight QTL (2136)
7	13826068	rs732496374	ITGA4	Average daily gain QTL (24915)
7	13826068	rs732496374	ITGA4	Wing weight QTL (2137)
7	13826068	rs732496374	ITGA4	Thigh muscle weight QTL (2139)
7	13826068	rs732496374	ITGA4	Thigh weight QTL (2141)
7	13826068	rs732496374	ITGA4	Drumstick weight QTL (2142)
7	13826068	rs732496374	ITGA4	Carcass weight QTL (2143)
7	13826068	rs732496374	ITGA4	Drumstick muscle weight QTL (2144)
7	13826068	rs732496374	ITGA4	Breast muscle weight QTL (2145)
7	13826068	rs732496374	ITGA4	Body weight QTL (2149)
7	13826068	rs732496374	ITGA4	Body weight QTL (2151)
7	13826068	rs732496374	ITGA4	Body weight QTL (2153)
7	13826068	rs732496374	ITGA4	Body weight QTL (2147)
7	13826068	rs732496374	ITGA4	Body weight QTL (2148)
7	13826068	rs732496374	ITGA4	Body weight QTL (2150)
7	13826068	rs732496374	ITGA4	Body weight QTL (2146)
7	13826068	rs732496374	ITGA4	Body weight QTL (2158)
7	13826068	rs732496374	ITGA4	Body weight QTL (2160)
7	13826068	rs732496374	ITGA4	Intestine length QTL (7166)
7	13826068	rs732496374	ITGA4	Body weight (35 days) QTL (17308)
7	13826068	rs732496374	ITGA4	Body weight (14 days) QTL (17305)
7	13826068	rs732496374	ITGA4	Body weight (77 days) QTL (17314)
7	13826306	rs738105407	ITGA4	Abdominal fat weight QTL (2140)
7	13826306	rs738105407	ITGA4	Abdominal fat weight QTL (12629)
7	13826306	rs738105407	ITGA4	Skin fat weight QTL (12639)
7	13826306	rs738105407	ITGA4	Fat distribution QTL (12644)
7	13826306	rs738105407	ITGA4	Drumstick and thigh muscle weight QTL (13397)
7	13826306	rs738105407	ITGA4	Drumstick and thigh muscle weight QTL (13407)
7	13826306	rs738105407	ITGA4	Body weight (112 days) QTL (6626)
7	13826306	rs738105407	ITGA4	Body weight (200 days) QTL (6627)
7	13826306	rs738105407	ITGA4	Body weight QTL (2136)
7	13826306	rs738105407	ITGA4	Average daily gain QTL (24915)
7	13826306	rs738105407	ITGA4	Wing weight QTL (2137)
7	13826306	rs738105407	ITGA4	Thigh muscle weight QTL (2139)

7	13826306	rs738105407	ITGA4	Thigh weight QTL (2141)
7	13826306	rs738105407	ITGA4	Drumstick weight QTL (2142)
7	13826306	rs738105407	ITGA4	Carcass weight QTL (2143)
7	13826306	rs738105407	ITGA4	Drumstick muscle weight QTL (2144)
7	13826306	rs738105407	ITGA4	Breast muscle weight QTL (2145)
7	13826306	rs738105407	ITGA4	Body weight QTL (2149)
7	13826306	rs738105407	ITGA4	Body weight QTL (2151)
7	13826306	rs738105407	ITGA4	Body weight QTL (2153)
7	13826306	rs738105407	ITGA4	Body weight QTL (2147)
7	13826306	rs738105407	ITGA4	Body weight QTL (2148)
7	13826306	rs738105407	ITGA4	Body weight QTL (2150)
7	13826306	rs738105407	ITGA4	Body weight QTL (2146)
7	13826306	rs738105407	ITGA4	Body weight QTL (2158)
7	13826306	rs738105407	ITGA4	Body weight QTL (2160)
7	13826306	rs738105407	ITGA4	Intestine length QTL (7166)
7	13826306	rs738105407	ITGA4	Body weight (35 days) QTL (17308)
7	13826306	rs738105407	ITGA4	Body weight (14 days) QTL (17305)
7	13826306	rs738105407	ITGA4	Body weight (77 days) QTL (17314)
8	20095084	rs315798025	GPBP1L1	Growth (42-56 days) QTL (12507)
8	20095084	rs315798025	GPBP1L1	Lactate level QTL (9236)
8	20095084	rs315798025	GPBP1L1	Yolk color QTL (36794)
8	20095084	rs315798025	GPBP1L1	Albumen percentage QTL (36764)
8	20095084	rs315798025	GPBP1L1	Albumen weight QTL (36752)
8	20095084	rs315798025	GPBP1L1	Tibia bone mineral density QTL (24360)
8	20095084	rs315798025	GPBP1L1	Body weight (504 days) QTL (24891)
8	20095084	rs315798025	GPBP1L1	Growth (70-105 days) QTL (55940)
8	20095084	rs315798025	GPBP1L1	Body weight (140 days) QTL (55922)
8	20095084	rs315798025	GPBP1L1	Drumstick and thigh weight QTL (17102)
8	20095084	rs315798025	GPBP1L1	Yolk index QTL (36786)
8	20095084	rs315798025	GPBP1L1	Albumen percentage QTL (36763)
8	20095084	rs315798025	GPBP1L1	Breast muscle weight QTL (2180)
8	20095084	rs315798025	GPBP1L1	Chest width QTL (9306)
8	20095084	rs315798025	GPBP1L1	Crooked digits QTL (9319)
8	20095084	rs315798025	GPBP1L1	Tibia weight QTL (9328)
8	20095084	rs315798025	GPBP1L1	Tibia width QTL (9336)
8	20095084	rs315798025	GPBP1L1	Tibia marrow diameter QTL (9343)
8	20095084	rs315798025	GPBP1L1	Tibia plateau angle QTL (9350)
8	20095084	rs315798025	GPBP1L1	Tibia strength QTL (9353)
8	20095084	rs315798025	GPBP1L1	Body weight (63 days) QTL (9767)
8	20095084	rs315798025	GPBP1L1	Growth (21-42 days) QTL (9768)
8	20095084	rs315798025	GPBP1L1	Breast muscle weight QTL (13389)

8	20095084	rs315798025	GPBP1L1	Drumstick muscle weight QTL (13392)
8	20095084	rs315798025	GPBP1L1	Body weight QTL (2190)
8	20095084	rs315798025	GPBP1L1	Wing weight QTL (2191)
8	20095084	rs315798025	GPBP1L1	Body weight QTL (2199)
8	20095084	rs315798025	GPBP1L1	Body weight QTL (2201)
8	20095084	rs315798025	GPBP1L1	Yolk color QTL (36788)
8	20095084	rs315798025	GPBP1L1	Albumen weight QTL (36749)
8	20095084	rs315798025	GPBP1L1	Marek's disease-related traits QTL (4081)
8	20095084	rs315798025	GPBP1L1	Yolk height QTL (36778)
8	20095084	rs315798025	GPBP1L1	Albumen weight QTL (36750)
8	20095084	rs315798025	GPBP1L1	Breast muscle weight QTL (9401)
8	20095084	rs315798025	GPBP1L1	Yolk index QTL (36783)
8	20095084	rs315798025	GPBP1L1	Albumen percentage QTL (36767)
8	20095084	rs315798025	GPBP1L1	Albumen percentage QTL (36765)
8	20095084	rs315798025	GPBP1L1	Yolk height QTL (36779)
8	20095084	rs315798025	GPBP1L1	Yolk index QTL (36782)
8	20095084	rs315798025	GPBP1L1	Egg shell shape QTL (2188)
8	20095084	rs315798025	GPBP1L1	Body weight (21 days) QTL (24844)
8	20095084	rs315798025	GPBP1L1	Body weight (42 days) QTL (24857)
8	20095084	rs315798025	GPBP1L1	Body weight (84 days) QTL (24868)
8	20095084	rs315798025	GPBP1L1	Body weight (168 days) QTL (24877)
8	20095084	rs315798025	GPBP1L1	Femur torsional strength QTL (6769)
8	20095084	rs315798025	GPBP1L1	Femur torsional strength QTL (6770)
8	20095084	rs315798025	GPBP1L1	Body weight (day of first egg) QTL (14458)
8	20095084	rs315798025	GPBP1L1	Body weight (day of first egg) QTL (14471)
8	20095084	rs315798025	GPBP1L1	Egg aftertaste QTL (6668)
10	11850414	rs314604288	ABHD17C	Total mortality QTL (2230)
10	11850414	rs314604288	ABHD17C	Body weight (140 days) QTL (55923)
10	11850414	rs314604288	ABHD17C	Body weight (35 days) QTL (55907)
10	11850414	rs314604288	ABHD17C	Growth (0-35 days) QTL (55931)
10	11850414	rs314604288	ABHD17C	Body weight (70 days) QTL (55911)
10	11850414	rs314604288	ABHD17C	Transport loss QTL (2233)
10	11850414	rs314604288	ABHD17C	Carcass weight QTL (17113)
10	11850414	rs314604288	ABHD17C	Body weight (105 days) QTL (55917)
10	11850414	rs314604288	ABHD17C	Antibody response to SRBC antigen QTL (2232)
10	11850414	rs314604288	ABHD17C	Egg shell strength QTL (14375)
10	11850414	rs314604288	ABHD17C	Egg shell stiffness QTL (14376)
10	11850703	rs315381519	ABHD17C	Total mortality QTL (2230)
10	11850703	rs315381519	ABHD17C	Body weight (140 days) QTL (55923)
10	11850703	rs315381519	ABHD17C	Body weight (35 days) QTL (55907)
10	11850703	rs315381519	ABHD17C	Growth (0-35 days) QTL (55931)

10	11850703	rs315381519	ABHD17C	Body weight (70 days) QTL (55911)
10	11850703	rs315381519	ABHD17C	Transport loss QTL (2233)
10	11850703	rs315381519	ABHD17C	Carcass weight QTL (17113)
10	11850703	rs315381519	ABHD17C	Body weight (105 days) QTL (55917)
10	11850703	rs315381519	ABHD17C	Antibody response to SRBC antigen QTL (2232)
10	11850703	rs315381519	ABHD17C	Egg shell strength QTL (14375)
10	11850703	rs315381519	ABHD17C	Egg shell stiffness QTL (14376)
10	11855189	rs317349624	ABHD17C	Total mortality QTL (2230)
10	11855189	rs317349624	ABHD17C	Body weight (140 days) QTL (55923)
10	11855189	rs317349624	ABHD17C	Body weight (35 days) QTL (55907)
10	11855189	rs317349624	ABHD17C	Growth (0-35 days) QTL (55931)
10	11855189	rs317349624	ABHD17C	Body weight (70 days) QTL (55911)
10	11855189	rs317349624	ABHD17C	Transport loss QTL (2233)
10	11855189	rs317349624	ABHD17C	Carcass weight QTL (17113)
10	11855189	rs317349624	ABHD17C	Body weight (105 days) QTL (55917)
10	11855189	rs317349624	ABHD17C	Antibody response to SRBC antigen QTL (2232)
10	11855189	rs317349624	ABHD17C	Egg shell strength QTL (14375)
10	11855189	rs317349624	ABHD17C	Egg shell stiffness QTL (14376)

Supplementary Table 26: Enriched QTL traits from the significant eQTLs of Liver of low and high FCR broilers

chr	pos	snp_id	Gene	QTL_Id
3	57057905	rs740509564	EPB41L2	Body weight QTL (3336)
3	57057905	rs740509564	EPB41L2	Egg shell color QTL (3337)
3	57057905	rs740509564	EPB41L2	Abdominal fat percentage QTL (9417)
3	57057905	rs740509564	EPB41L2	Body weight (63 days) QTL (17280)
3	57057905	rs740509564	EPB41L2	Body weight (70 days) QTL (17281)
3	57057905	rs740509564	EPB41L2	Body weight (56 days) QTL (17279)
3	57057905	rs740509564	EPB41L2	Glucose level QTL (9419)
3	57057905	rs740509564	EPB41L2	Drumstick and thigh percentage QTL (15556)
3	57057905	rs740509564	EPB41L2	Wing percentage QTL (15559)
3	57057905	rs740509564	EPB41L2	Drumstick and thigh percentage QTL (15584)
3	57057905	rs740509564	EPB41L2	Wing percentage QTL (15588)
3	57057905	rs740509564	EPB41L2	Tibia stress QTL (1959)
3	57057905	rs740509564	EPB41L2	Tibia length QTL (1963)
3	57057905	rs740509564	EPB41L2	Tibia area QTL (1964)
3	57057905	rs740509564	EPB41L2	Tibia breaking force QTL (1967)
3	57057905	rs740509564	EPB41L2	Fear-stand latency QTL (1966)
3	57057905	rs740509564	EPB41L2	Residual feed intake QTL (64556)
3	57057905	rs740509564	EPB41L2	Thigh muscle weight QTL (9392)
3	57057905	rs740509564	EPB41L2	Proventriculus weight QTL (9393)
3	57057905	rs740509564	EPB41L2	Age at first egg QTL (14450)
3	57057905	rs740509564	EPB41L2	Body weight (day of first egg) QTL (14469)
3	57057905	rs740509564	EPB41L2	Body weight QTL (1979)
3	57057905	rs740509564	EPB41L2	Body weight QTL (1980)
3	57057905	rs740509564	EPB41L2	Body weight (35 days) QTL (7180)
3	57057905	rs740509564	EPB41L2	Body weight (35 days) QTL (55904)
3	57057905	rs740509564	EPB41L2	Growth (0-35 days) QTL (55929)
3	57057905	rs740509564	EPB41L2	Body weight QTL (1957)
3	57057905	rs740509564	EPB41L2	Body weight (49 days) QTL (30854)
3	57057905	rs740509564	EPB41L2	Body weight QTL (1961)
3	57057905	rs740509564	EPB41L2	Body weight QTL (1962)
3	57057905	rs740509564	EPB41L2	Body weight (8 days) QTL (6610)
3	57057905	rs740509564	EPB41L2	Body weight (112 days) QTL (6611)
3	57057905	rs740509564	EPB41L2	Body weight (200 days) QTL (6612)
3	57057905	rs740509564	EPB41L2	Growth (1-8 days) QTL (6613)
3	57057905	rs740509564	EPB41L2	Lung weight QTL (1960)
3	57057905	rs740509564	EPB41L2	Body weight (63 days) QTL (9420)
3	57057905	rs740509564	EPB41L2	Skin fat weight QTL (1965)
3	57057905	rs740509564	EPB41L2	Skin fat weight QTL (12635)

3	57057905	rs740509564	EPB41L2	Leg twisting QTL (9313)
3	57057905	rs740509564	EPB41L2	Antibody response to KLH antigen QTL (1968)
3	57057905	rs312273451	EPB41L2	Antibody response to SRBC antigen QTL (1938)
5	36457370	rs312273451	AQR	Time to achieve maximum antibody response to SRBC QTL (2081)
5	36457370	rs312273451	AQR	Shank diameter QTL (9429)
5	36457370	rs312273451	AQR	Antibody response to SRBC antigen QTL (2075)
5	36457370	rs312273451	AQR	Shank length QTL (9428)
5	36457370	rs312273451	AQR	Egg number QTL (17070)
5	36457370	rs312273451	AQR	Carcass weight QTL (17112)
5	36457370	rs312273451	AQR	Growth (70-105 days) QTL (55939)
5	36457370	rs312273451	AQR	Breast muscle weight QTL (9430)
5	36457370	rs312273451	AQR	Egg weight QTL (17071)
5	36457370	rs312273451	AQR	Glucose level QTL (9434)
5	36457370	rs312273451	AQR	Body weight (40 days) QTL (9383)
5	36457370	rs312273451	AQR	Conformation score QTL (9384)
5	36457370	rs312273451	AQR	Abdominal fat weight QTL (9432)
5	36457370	rs312273451	AQR	Body weight QTL (2084)
5	36457370	rs312273451	AQR	Abdominal fat percentage QTL (9433)
5	36457370	rs312273451	AQR	Egg weight QTL (17072)
5	36457370	rs312273451	AQR	Body weight (140 days) QTL (17079)
5	36457370	rs312273451	AQR	Body depth QTL (16715)
5	36457370	rs312273451	AQR	Marek's disease-related traits QTL (4597)
5	36457370	rs312273451	AQR	Body weight (40 days) QTL (6661)
5	36457370	rs312273451	AQR	Growth (42-63 days) QTL (9763)
5	36457370	rs312273451	AQR	Antibody titer to LTA antigen QTL (16626)
5	36457370	rs312273451	AQR	Gizzard weight QTL (2091)
5	36457370	rs312273451	AQR	Troponin T concentration QTL (2092)
5	36457370	rs312273451	AQR	Wing weight QTL (2094)
5	36457370	rs312273451	AQR	Breast muscle weight QTL (6707)

Supplementary Table 27: Summary of 16S rDNA Amplicon and Metagenome WGS sequencing data for Low and High FCR broilers.

Sample Name	FCR_Group	Number of 16S rDNA sequences	Intestinal Lumen Segment
828CP2	Low	74271	Cecum
1069CP2	High	84179	Cecum
1001CP2	Low	70488	Cecum
995CP2	High	59233	Cecum
999CP2	Low	55790	Cecum
1003CP2	High	33861	Cecum
7475CP2	Low	75918	Cecum
7473CP2	High	42511	Cecum
7549CP2	Low	45935	Cecum
7550CP2	High	44036	Cecum
7844CP2	Low	47568	Cecum
7843CP2	High	33358	Cecum
5154CP2	Low	32650	Cecum
5153CP2	High	2030	Cecum
1069IP2	High	125869	Ileum
1001IP2	Low	51619	Ileum
995IP2	High	89022	Ileum
1003IP2	High	89430	Ileum
7475IP2	Low	146588	Ileum
7473IP2	High	90183	Ileum
7549IP2	Low	86712	Ileum
7550IP2	High	134324	Ileum
7844IP2	Low	106892	Ileum
7843IP2	High	62119	Ileum
5154IP2	Low	87295	Ileum
5153IP2	High	74397	Ileum
7475JP2	Low	95825	Jejunum
7473JP2	High	57743	Jejunum
7549JP2	Low	99181	Jejunum
7550JP2	High	95379	Jejunum
7844JP2	Low	42045	Jejunum
7843JP2	High	64047	Jejunum
5154JP2	Low	111644	Jejunum
5153JP2	High	115289	Jejunum

	7475_J	7475_I	7475_C	7473_J	7473_I	7473_C	5154_J	5154_I	5154_C	5153_J
Total Reads	2,166,622	808,739	1,030,079	2,551,858	920,150	977,463	2,265	1,187,171	1,415,384	3,092
Total Bases (MB)	530,259,740	219,564,909	290,727,566	717,761,437	259,058,872	262,725,500	559,871	330,883,556	396,296,728	761,937
QC passed reads	1,868,343	723,948	924,227	2,317,244	815,758	924,749	1,101	988,802	1,300,025	1,580
Reads assigned to 16S rDNA gene	11,961	8,921	5,241	20,427	15,341	4,441	4	10,201	6,598	3
Reads assigned to predicted proteins with known functions	561,132	616,707	605,661	2,098,468	719,621	578,996	530	920,263	977,252	618
Reads assigned to predicted proteins with unknown functions	952,260	87,011	280,081	89,025	80,796	318,148	378	57,046	292,394	701
Unassigned Reads	342,990	11,309	33,244	109,324	0	23,164	189	1,292	23,781	258

	5153_I	5153_C	7550_J	7550_I	7550_C	7549_J	7549_I	7549_C	828_J	828_I
Total Reads	2,089,034	1,547,105	4,450,453	1,844,715	1,097,544	4,055,402	1,052,271	1,194,847	ns	2,445,985
Total Bases (MB)	574,951,257	419,755,537	1,201,784,496	512,738,144	304,607,746	1,100,913,600	275,261,375	324,327,232	ns	617,467,706
QC passed reads	1,517,501	1,447,786	2,450,532	1,469,039	1,014,411	507,820	993,874	1,087,632	ns	2,054,488
Reads assigned to 16S rDNA gene	22,443	7,905	42,579	25,059	4,756	37,713	1,955	9,847	ns	14,415
Reads assigned to predicted proteins with known functions	1,334,024	943,732	2,195,181	1,358,360	733,426	3,146,463	909,566	825,282	ns	1,282,722
Reads assigned to predicted proteins with unknown functions	108,870	460,574	141,133	85,620	251,745	159,631	49,148	225,988	ns	585,120
Unassigned Reads	52,164	35,575	71,639	0	24,484	203,775	33,205	26,515	ns	172,231

	828_C		1069_J	1069_I	1069_C	1001_J	1001_I	1001_C	995_J	995_I	995_C
Total Reads	755,616		ns	ns	4,051,921	ns	ns	4,785,112	ns	ns	1,466,313
Total Bases (MB)	201,558,700		ns	ns	1,089,160,558	ns	ns	1,298,071,157	ns	ns	400,943,396
QC passed reads	632,508		ns	ns	3,265,347	ns	ns	4,331,833	ns	ns	1,252,743
Reads assigned to 16S rDNA gene	583		ns	ns	5,770	ns	ns	11,386	ns	ns	6,358
Reads assigned to predicted proteins with known functions	473,780		ns	ns	2,464,417	ns	ns	3,282,214	ns	ns	842,838
Reads assigned to predicted proteins with unknown functions	125,563		ns	ns	597,836	ns	ns	690,681	ns	ns	327,326
Unassigned Reads	32,582		ns	ns	197,324	ns	ns	347,552	ns	ns	76,221

	1003_J	1003_I	1003_C	999_J	999_I	999_C
Total Reads	ns	ns	2,008,387	ns	ns	1,781,575
Total Bases (MB)	ns	ns	539,622,613	ns	ns	482,682,900
QC passed reads	ns	ns	1,107,710	ns	ns	1,310,857
Reads assigned to 16S rDNA gene	ns	ns	935	ns	ns	11,676
Reads assigned to predicted proteins with known functions	ns	ns	774,249	ns	ns	834,023
Reads assigned to predicted proteins with unknown functions	ns	ns	235,166	ns	ns	372,741
Unassigned Reads	ns	ns	97,360	ns	ns	92,417

*ns- no sequencing available

Supplementary Table 28: Summary of alpha diversity indices of Low and High FCR broilers in each gut location

Sample Name	FCR Group	Number of 16S rDNA sequences	OTUs observed	Intestinal Lumen Segment	Observed species	chao1	shannon	simpson	PD whole tree
828CP2	Low	74271	1547	Cecum	1109	1938.188679	6.150532933	0.949750649	56.52874
1069CP2	High	84179	1753	Cecum	1143	1967.127273	6.683978196	0.972653254	61.71039
1001CP2	Low	70488	1741	Cecum	1266	2540.900662	7.182267606	0.98057711	62.13224
995CP2	High	59233	1981	Cecum	1533	2624.85	6.999544839	0.969743178	68.37848
999CP2	Low	55790	1587	Cecum	1260	2265.386503	7.232521258	0.979670022	66.43431
1003CP2	High	33861	1325	Cecum	1303	2309.449275	6.574841267	0.962929916	58.73034
7475CP2	Low	75918	1796	Cecum	1265	2143.160194	5.987944943	0.941923779	71.68984
7473CP2	High	42511	1398	Cecum	1247	1939.354839	6.937756163	0.974967162	62.85467
7549CP2	Low	45935	926	Cecum	804	1318.96124	5.968140425	0.964930673	45.81486
7550CP2	High	44036	947	Cecum	851	1223.554745	5.575108326	0.924005591	47.0593
7844CP2	Low	47568	944	Cecum	825	1246.53125	5.251442829	0.921432165	51.92022
7843CP2	High	33358	748	Cecum	744	937.0571429	5.424796662	0.922661383	43.29491
5154CP2	Low	32650	706	Cecum	705	921.3941606	5.142297941	0.918644715	44.62335
5153CP2	High	2030	753	Cecum	Missing	Missing	Missing	Missing	Missing
1069IP2	High	125869	2390	Ileum	1191	2729.534884	3.657442436	0.734774815	71.85003
1001IP2	Low	51619	1346	Ileum	1096	1840.924623	3.675090465	0.707061595	64.64801
995IP2	High	89022	835	Ileum	467	965.0540541	2.161487717	0.491904473	36.14271
1003IP2	High	89430	2433	Ileum	1531	2914.895161	5.23275347	0.868920439	75.25072
7475IP2	Low	146588	461	Ileum	198	420.6	3.282345612	0.833989976	12.61994
7473IP2	High	90183	412	Ileum	275	573	3.68924827	0.8708781	15.41917
7549IP2	Low	86712	569	Ileum	360	661.65625	3.519785163	0.834901336	21.83351
7550IP2	High	134324	768	Ileum	356	867.2162162	3.291094252	0.77438405	23.10623
7844IP2	Low	106892	480	Ileum	251	443.3846154	2.964102528	0.747875584	16.29818
7843IP2	High	62119	769	Ileum	593	916.619403	4.854111487	0.93739622	23.73904
5154IP2	Low	87295	289	Ileum	184	463.5882353	2.844374103	0.757146424	13.06194
5153IP2	High	74397	436	Ileum	301	553.7826087	3.234588674	0.744507181	16.28856
7475JP2	Low	95825	722	Jejunum	428	745.2891566	1.229510156	0.238025924	41.95528
7473JP2	High	57743	159	Jejunum	108	169.25	2.095945748	0.676974431	12.57793
7549JP2	Low	99181	338	Jejunum	192	479.3684211	2.839447122	0.775358879	15.1042
7550JP2	High	95379	280	Jejunum	167	353.1363636	2.171498597	0.618986017	14.9111
7844JP2	Low	42045	95	Jejunum	84	167.1538462	1.76588882	0.623456359	9.85643
7843JP2	High	64047	185	Jejunum	140	219.5652174	2.769637302	0.789804882	11.25231
5154JP2	Low	111644	505	Jejunum	247	760.3703704	2.183118937	0.666359124	26.63987
5153JP2	High	115289	430	Jejunum	188	426.5555556	0.925200297	0.218752924	17.31269

Non-Paramatic for each pair using Wilcoxon method

Group	Z-value	p-Value
Jejunum-Ileum	-2.58	0.0097
Jejunum-Cecum	-3.72	0.0002
Ileum-Cecum	-2.03	0.0422

Supplementary Table 29: Adonis and PERMANOVA results of microbial diversity between low and high FCR chickens based on Bray-Curtis distances with 999 permutations. MS, mean sum of squares; SS, sum of squares

	DF	SS	MS	Pseudo F	R^2	p-value
**Jejunum**						
FCR	1	0.05615	0.056151	0.55423	0.08456	0.415
Residuals	6	0.60788	0.101314		0.91544	
Total	7	0.66403			1.0000	
**Ileum**						
FCR	1	0.26329	0.26329	3.415	0.25456	**0.036***
Residuals	10	0.77100	0.07710		0.74544	
Total	11	1.03430			1.0000	
**Cecum**						
FCR	1	0.13552	0.13552	1.0647	0.08825	0.35
Residuals	11	1.40015	0.12729		0.91175	
Total	12	1.53566			1.0000	

Supplementary Table 30: Adjusted p-value for correlation between bacterial abundance and differentially expressed host genes in jejunum of low and high FCR.

	GABARAPL2	ENSGALG000 00027410	KLHL17	PMM2	PQLC2	USO1	RPS15A	ANO5	FABP5	SLC25A22	Lactobacillus	Klebsiella	Cronobacter	Escherichia	Clostridium	Pantoea	Shigella	Enterobacter	Citrobacter	Enterococcus
GABARAPL2		<.0001	<.0001	1	<.0001	<.0001	1	1	<.0001	1	1	0.7456	1	1	0.298	0.136	0.497	0.672	0.825	0.26
ENSGALG000 00027410	<.0001		<.0001	1	<.0001	<.0001	1	1	<.0001	<.0001	1	0.5259	1	1	0.242	0.549	0.136	0.136	0.222	0.345
KLHL17	<.0001	<.0001		1	<.0001	<.0001	<.0001	1	<.0001	<.0001	1	1	1	1	0.546	0.824	0.904	0.965	0.747	0.652
PMM2	1	1	1		1	<.0001	1	1	<.0001	<.0001	0.721	1	0.4873	1	1	0.6532	0.773	0.523	0.1946	0.55
PQLC2	<.0001	<.0001	<.0001	1		<.0001	1	1	<.0001	1	0.323	1	1	1	0.5164	0.87	0.18	0.415	0.347	0.54
USO1	<.0001	<.0001	<.0001	<.0001	<.0001		<.0001	<.0001	<.0001	<.0001	0.0014	1	1	1	0.1584	0.0109	0.0209	0.0111	0.0207	<.0001
RPS15A	1	1	1	1	1	<.0001		1	<.0001	<.0001	1	0.2171	1	1	0.1079	0.193	0.1584	0.1928	0.1239	<.0001
ANO5	1	1	1	1	1	1	1		1	<.0001	0.1946	1	1	1	0.6406	0.84	<.0001	<.0001	0.627	0.1684
FABP5	<.0001	<.0001	<.0001	<.0001	<.0001	<.0001	<.0001	1		<.0001	1	1	1	1	0.2345	0.179	0.108	0.1	0.227	0.397
SLC25A22	1	<.0001	<.0001	<.0001	1	<.0001	<.0001	<.0001	<.0001		0.104	1	1	1	0.2357	0.189	0.7	0.454	0.239	0.25
Lactobacillus	1	1	1	0.721	0.323	0.5014	1	0.1946	1	0.104		<.0001	1	0.651	0.2574	0.8252	1	1	0.193	<.0001
Klebsiella	0.7456	0.5259	1	1	1	1	0.2171	1	1	1	<.0001		1	0.4	1	1	1	1	1	0.56
Cronobacter	1	1	1	0.4873	1	1	1	1	1	1	1	1		0.0044	1	1	1	1	1	1
Escherichia	1	1	1	1	1	1	1	1	1	1	0.651	0.4	0.0044		1	1	1	1	1	1
Clostridium	0.298	0.242	0.546	1	0.5164	0.1584	0.1079	0.6406	0.2345	0.2357	0.2574	1	1	1		1	1	1	1	0.2621
Pantoea	0.136	0.549	0.824	0.6532	0.87	0.109	0.193	0.84	0.179	0.189	0.8252	1	1	1	1		0.9058	0.6707	<.0001	0.1166
Shigella	0.497	0.136	0.904	0.773	0.18	0.209	0.1584	<.0001	0.108	0.7	1	1	1	1	1	0.9058		<.0001	0.2015	1
Enterobacter	0.672	0.136	0.965	0.523	0.415	0.111	0.1928	<.0001	0.1	0.454	1	1	1	1	1	0.6707	<.0001		0.2357	1
Citrobacter	0.825	0.222	0.747	0.1946	0.347	0.207	0.1239	0.627	0.227	0.239	0.193	1	1	1	1	<.0001	0.2015	0.2357		0.2129
Enterococcus	0.26	0.345	0.652	0.55	0.54	<.0001	<.0001	0.1684	0.397	0.25	<.0001	0.56	1	1	0.2621	0.1166	1	1	0.2129	

Supplementary Table 31: Adjusted p-value for correlation between bacterial abundance and differentially expressed host genes in ileum of low and high FCR.

	CENPO	HSP90AA1	G3BP1	LOC424109	Lactobacillus	Enterococcus	Clostridium	Weissella	Rothia	Bacillus	Veillonella	Gallibacterium	Faecalibacterium	Sarcina
CENPO		1	0.0173	1	1	1	1	1	1	0.6438	1	1	1	1
HSP90AA1	1		1	1	1	0.8251	0.0002	0.4491	0.3358	0.6155	0.676	0.1039	1	<.0001
G3BP1	0.0173	1		1	1	1	1	1	1	1	0.5329	0.4211	0.2035	1
LOC424109	1	1	1		1	0.676	1	1	1	1	0.995	1	0.9624	1
Lactobacillus	1	1	1	1		1	0.026	1	1	1	1	1	1	0.8795
Enterococcus	1	0.8251	1	0.676	1		1	1	1	1	0.0513	0.0005	0.1027	1
Clostridium	1	0.0002	1	1	0.026	1		0.8251	0.68	0.4569	1	1	1	0.003
Weissella	1	0.4491	1	1	1	1	0.8251		<.0001	1	1	1	1	0.8036
Rothia	1	0.3358	1	1	1	1	0.68	<.0001		1	1	1	1	0.6429
Bacillus	0.6438	0.6155	1	1	1	1	0.4569	1	1		1	1	1	0.2132
Veillonella	1	0.676	0.5329	0.995	1	0.0513	1	1	1	1		0.04	<.0001	1
Gallibacterium	1	0.1039	0.4211	1	1	0.0005	1	1	1	1	0.04		0.0593	0.676
Faecalibacterium	1	1	0.2035	0.9624	1	0.1027	1	1	1	1	<.0001	0.0593		1
Sarcina	1	<.0001	1	1	0.8795	1	0.003	0.8036	0.6429	0.2132	1	0.676	1	

Supplementary Table 32: Adjusted p-value for correlation between bacterial abundance and differentially expressed host genes in ceca of low and high FCR.

	COL18A1	EML6	PLCD1	ITGA4	DNAJA3	INVS	GPBP1L1	EIF2S3L	SH3BGRL	NADK	Alistipes	Bacteroides	Faecali-bacterium	Selenomonas	Clostridium	Rumino-coccus	Barnesiella	Lacto-bacillus	Parabacteroides	Alkaliphilus
COL18A1		<.0001	<.0001	<.0001	<.0001	0.5176	0.3941	0.326	0.1166	0.2135	0.152	1	1	1	1	0.5176	1	1	1	1
EML6	<.0001		<.0001	<.0001	<.0001	0.5303	0.42	0.3369	0.1121	0.2122	0.145	1	1	1	1	0.5297	1	1	1	1
PLCD1	<.0001	<.0001		<.0001	<.0001	0.5176	0.4126	0.3104	0.1114	0.2135	0.0117	1	1	1	1	0.4573	1	1	1	1
ITGA4	<.0001	<.0001	<.0001		<.0001	0.1503	0.1401	0.711	0.256	0.725	0.0054	1	1	1	1	0.284	1	1	1	1
DNAJA3	<.0001	<.0001	<.0001	<.0001		0.3522	0.2656	0.2012	0.68	0.1401	0.133	1	1	1	1	0.4863	1	1	1	1
INVS	0.5176	0.5303	0.5176	0.1503	0.3522		<.0001	<.0001	<.0001	<.0001	0.4351	1	1	1	1	1	1	1	1	1
GPBP1L1	0.3941	0.42	0.4126	0.1401	0.2656	<.0001		<.0001	<.0001	<.0001	1	1	1	1	1	1	1	1	1	1
EIF2S3L	0.326	0.3369	0.3104	0.711	0.2012	<.0001	<.0001		<.0001	0.903	0.1252	0.608	1	1	1	0.4558	1	1	1	1
SH3BGRL	0.1166	0.1121	0.1114	0.256	0.68	<.0001	<.0001	<.0001		<.0001	1	1	1	1	1	1	1	1	1	1
NADK	0.2135	0.2122	0.2135	0.725	0.1401	<.0001	<.0001	0.903	<.0001		1	1	1	1	1	1	1	1	1	1
Alistipes	0.152	0.145	0.0117	0.0054	0.133	0.4351	1	0.1252	1	1		0.79	1	0.3791	0.569	<.0001	1	1	0.5641	0.0207
Bacteroides	1	1	1	1	1	1	1	0.608	1	1	0.79		1	1	0.0001	<.0001	1	0.2721	0.26	0.0005
Faecalibacterium	1	1	1	1	1	1	1	1	1	1	1	1		1	1	1	1	1	1	1
Selenomonas	1	1	1	1	1	1	1	1	1	1	0.3791	1	1		0.5169	0.1472	1	1	1	0.603
Clostridium	1	1	1	1	1	1	1	1	1	1	0.569	0.0001	1	0.5169		0.0065	1	0.0878	0.145	<.0001
Ruminococcus	0.5176	0.5297	0.4573	0.284	0.4863	1	1	0.4558	1	1	<.0001	<.0001	1	0.1472	0.0065		1	1	0.4863	0.0004
Barnesiella	1	1	1	1	1	1	1	1	1	1	1	1	1	1	1	1		1	1	1
Lactobacillus	1	1	1	1	1	1	1	1	1	1	1	0.2721	1	1	0.0878	1	1		1	1
Parabacteroides	1	1	1	1	1	1	1	1	1	1	0.5641	0.26	1	1	0.145	0.4863	1	1		0.404
Alkaliphilus	1	1	1	1	1	1	1	1	1	1	0.0207	0.0005	1	0.603	<.0001	0.0004	1	1	0.404	

Supplementary Table 33: Adjusted p-value for correlation between functional features of the enteric microbiota and differentially expressed host genes in jejunum of low and high FCR.

	GABARAPL2	ENSGALG00000027410	KLHL17	PMM2	PQLC2	USO1	RPS15A	ANO5	FABP5	SLC25A22	ABC transporters	Aminoacyl-tRNA biosynthesis	Two-component system	Pyruvate metabolism	Purine metabolism	Phospho-transferase system (PTS)	DNA replication	Ribosome	Pentose phosphate pathway	Bacterial secretion system
GABARAPL2		<.0001	<.0001	1	<.0001	<.0001	1	1	<.0001	1	1	0.2291	0.061	<.0001	0.0002	1	0.894	1	0.378	1
ENSGALG00000027410	<.0001		<.0001	1	<.0001	<.0001	1	1	<.0001	<.0001	1	0.3179	0.647	0.269	0.872	1	0.183	1	0.764	0.8253
KLHL17	<.0001	<.0001		1	<.0001	<.0001	<.0001	1	<.0001	<.0001	1	1	0.325	0.148	0.3179	1	0.155	1	0.173	0.3179
PMM2	1	1	1		1	<.0001	1	1	<.0001	<.0001	1	1	0.8121	1	1	1	0.135	1	0.1374	0.1218
PQLC2	<.0001	<.0001	<.0001	1		<.0001	1	1	<.0001	1	0.7062	0.4105	0.1184	0.123	0.0003	1	0.1336	1	0.212	1
USO1	<.0001	<.0001	<.0001	<.0001	<.0001		<.0001	<.0001	<.0001	<.0001	1	0.3068	0.695	0.165	0.3179	1	0.198	1	0.167	0.5572
RPS15A	1	1	1	1	1	<.0001		1	<.0001	<.0001	1	0.206	0.154	0.2889	0.3736	1	0.106	1	0.157	1
ANO5	1	1	1	1	1	1	1		1	<.0001	1	0.1107	0.137	1	1	1	0.126	1	0.107	0.443
FABP5	<.0001	<.0001	<.0001	<.0001	<.0001	<.0001	<.0001	1		<.0001	1	0.2239	0.1729	0.1514	0.1824	1	0.155	1	0.198	1
SLC25A22	1	<.0001	<.0001	<.0001	1	<.0001	<.0001	<.0001	<.0001		1	0.2532	0.14	0.6051	1	1	0.234	1	0.113	0.1803
ABC transporters	1	1	1	1	0.7062	1	1	1	1	1		1	1	0.2686	0.3179	1	1	<.0001	1	1
Aminoacyl-tRNA biosynthesis	0.2291	0.3179	1	1	0.4105	0.3068	0.206	0.1107	0.2239	0.2532	1		<.0001	0.4347	0.4347	1	0.14	1	0.1803	1
Two-component system	0.061	0.647	0.325	0.8121	0.1184	0.695	0.154	0.137	0.1729	0.14	1	<.0001		0.2686	0.3167	1	<.0001	1	0.0049	1
Pyruvate metabolism	<.0001	0.269	0.148	1	0.123	0.165	0.2889	1	0.1514	0.6051	0.2686	0.4347	0.2686		<.0001	0.1286	1	1	0.107	1
Purine metabolism	0.0002	0.872	0.3179	1	0.0003	0.3179	0.3736	1	0.1824	1	0.3179	0.4347	0.3167	<.0001		0.0046	1	1	0.1503	1
Phosphotransferase system (PTS)	1	1	1	1	1	1	1	1	1	1	1	1	1	0.1286	0.0046		1	1	1	1
DNA replication	0.894	0.183	0.155	0.135	0.1336	0.198	0.106	0.126	0.155	0.234	1	0.14	<.0001	1	1	1		0.5572	0.106	0.1478
Ribosome	1	1	1	1	1	1	1	1	1	1	<.0001	1	1	1	1	1	0.5572		1	1
Pentose phosphate pathway	0.378	0.764	0.173	0.1374	0.212	0.167	0.157	0.107	0.198	0.113	1	0.1803	0.0049	0.107	0.1503	1	0.106	1		0.0141
Bacterial secretion system	1	0.8253	0.3179	0.1218	1	0.5572	1	0.443	1	0.1803	1	1	1	1	1	1	0.1478	1	0.0141	

Supplementary Table 34: Adjusted p-value for correlation between functional features of the enteric microbiota and differentially expressed host genes in ileum of low and high FCR.

	CENPO	HSP90AA1	G3BP1	LOC424109	Metabolism of cofactors and vitamins	Transport and catabolism	Xenobiotics biodegradation and metabolism	Translation	Cell growth and death	Nucleotide metabolism	Glycan biosynthesis and metabolism	Replication and repair	Cancers	Signal transduction
CENPO		1	0.0173	1	0.104	0.104	0.151	0.104	0.109	0.278	0.109	0.188	0.1207	0.1034
HSP90AA1	1		1	1	1	1	1	1	1	1	1	1	1	1
G3BP1	0.0173	1		1	<.0001	<.0001	<.0001	<.0001	<.0001	0.107	<.0001	<.0001	<.0001	<.0001
LOC424109	1	1	1		0.246	0.338	0.432	0.519	0.223	0.106	0.168	0.175	1	0.102
Metabolism of cofactors and vitamins	0.104	1	<.0001	0.246		<.0001	<.0001	<.0001	<.0001	<.0001	<.0001	<.0001	<.0001	<.0001
Transport and catabolism	0.104	1	<.0001	0.338	<.0001		<.0001	<.0001	<.0001	<.0001	<.0001	<.0001	<.0001	<.0001
Xenobiotics biodegradation and metabolism	0.151	1	<.0001	0.432	<.0001	<.0001		<.0001	<.0001	<.0001	<.0001	<.0001	0.102	<.0001
Translation	0.104	1	<.0001	0.519	<.0001	<.0001	<.0001		<.0001	<.0001	<.0001	<.0001	<.0001	<.0001
Cell growth and death	0.109	1	<.0001	0.223	<.0001	<.0001	<.0001	<.0001		<.0001	<.0001	<.0001	<.0001	<.0001
Nucleotide metabolism	0.278	1	0.107	0.106	<.0001	<.0001	<.0001	<.0001	<.0001		0.142	<.0001	0.109	<.0001
Glycan biosynthesis and metabolism	0.109	1	<.0001	0.168	<.0001	<.0001	<.0001	<.0001	<.0001	0.142		<.0001	0.129	<.0001
Replication and repair	0.188	1	<.0001	0.175	<.0001	<.0001	<.0001	<.0001	<.0001	<.0001	<.0001		<.0001	<.0001
Cancers	0.1207	1	<.0001	1	<.0001	<.0001	0.102	<.0001	<.0001	0.109	0.129	<.0001		0.103
Signal transduction	0.1034	1	<.0001	0.102	<.0001	<.0001	<.0001	<.0001	<.0001	<.0001	<.0001	<.0001	0.103	

Supplementary Table 35: Adjusted p-value for correlation between functional features of the enteric microbiota and differentially expressed host genes in ceca of low and high FCR.

	COL18A1	EML6	PLCD1	ITGA4	DNAJA3	INVS	GPBP1L1	EIF2S3L	SH3BGRL	NADK	Metabolism of terpenoids and polyketides	Biosynthesis of other secondary metabolites	Metabolism of other amino acids
COL18A1		<.0001	<.0001	<.0001	<.0001	0.5176	0.3941	0.326	0.1166	0.2135	<.0001	0.1299	0.1358
EML6	<.0001		<.0001	<.0001	<.0001	0.5303	0.42	0.3369	0.1121	0.2122	<.0001	0.1299	0.1358
PLCD1	<.0001	<.0001		<.0001	<.0001	0.5176	0.4126	0.3104	0.1114	0.2135	<.0001	0.1299	0.1358
ITGA4	<.0001	<.0001	<.0001		<.0001	0.1503	0.1401	0.711	0.256	0.725	<.0001	0.1182	0.1354
DNAJA3	<.0001	<.0001	<.0001	<.0001		0.3522	0.2656	0.2012	0.68	0.1401	<.0001	0.1299	0.1358
INVS	0.5176	0.5303	0.5176	0.1503	0.3522		<.0001	<.0001	<.0001	<.0001	<.0001	0.1817	0.0361
GPBP1L1	0.3941	0.42	0.4126	0.1401	0.2656	<.0001		<.0001	<.0001	<.0001	0.0098	0.457	0.0434
EIF2S3L	0.326	0.3369	0.3104	0.711	0.2012	<.0001	<.0001		<.0001	0.903	<.0001	0.1358	0.0683
SH3BGRL	0.1166	0.1121	0.1114	0.256	0.68	<.0001	<.0001	<.0001		<.0001	0.0037	0.4544	0.007
NADK	0.2135	0.2122	0.2135	0.725	0.1401	<.0001	<.0001	0.903	<.0001		0.102	0.457	0.451
Metabolism of terpenoids and polyketides	<.0001	<.0001	<.0001	<.0001	<.0001	<.0001	0.108	<.0001	0.0037	0.102		0.0451	0.1299
Biosynthesis of other secondary metabolites	0.1299	0.1299	0.1299	0.1182	0.1299	0.1817	0.457	0.1358	0.4544	0.457	0.0451		0.4544
Metabolism of other amino acids	0.1358	0.1358	0.1358	0.1354	0.1358	0.0361	0.0434	0.0683	0.007	0.451	0.1299	0.4544	

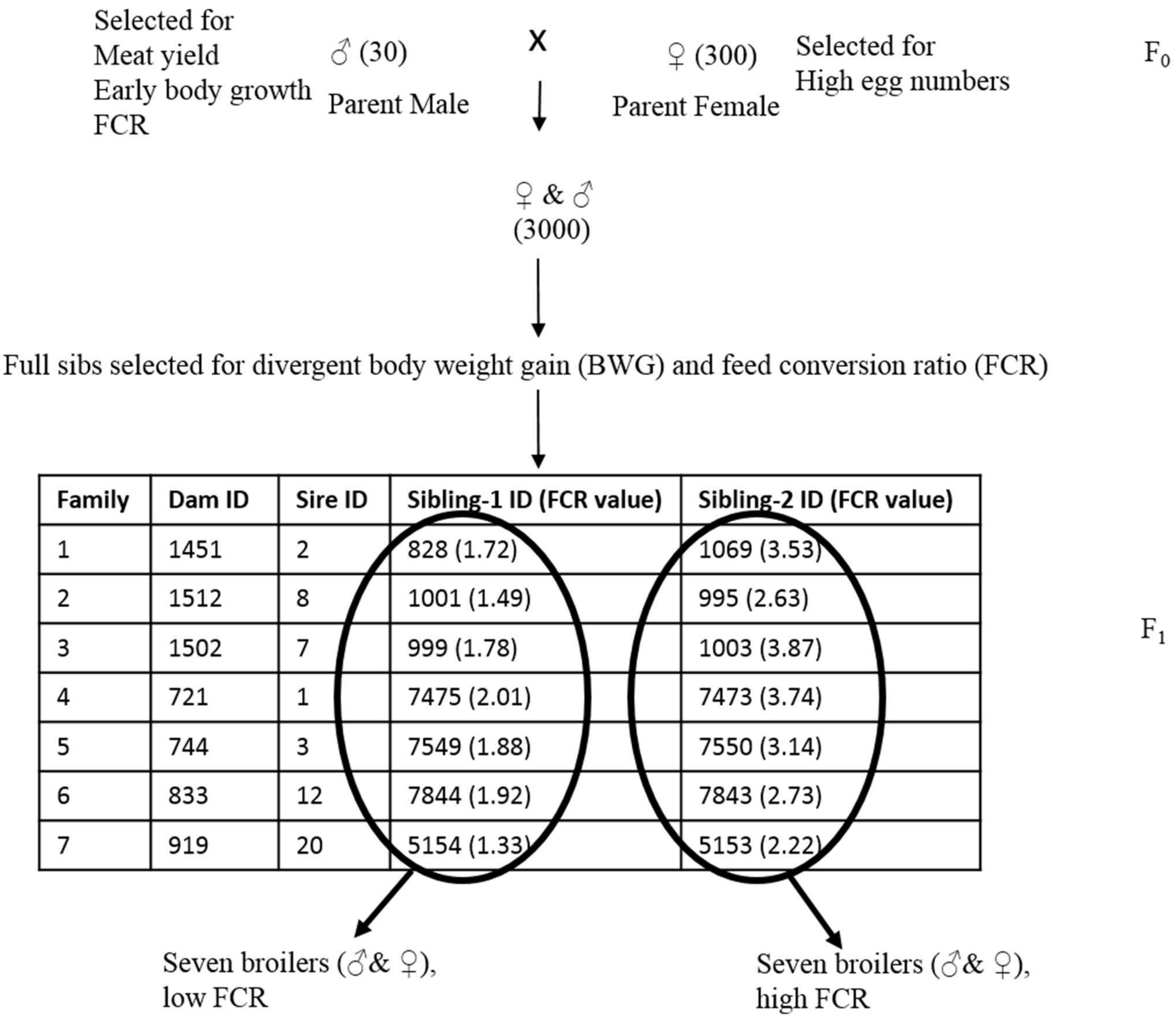

Family	Dam ID	Sire ID	Sibling-1 ID (FCR value)	Sibling-2 ID (FCR value)
1	1451	2	828 (1.72)	1069 (3.53)
2	1512	8	1001 (1.49)	995 (2.63)
3	1502	7	999 (1.78)	1003 (3.87)
4	721	1	7475 (2.01)	7473 (3.74)
5	744	3	7549 (1.88)	7550 (3.14)
6	833	12	7844 (1.92)	7843 (2.73)
7	919	20	5154 (1.33)	5153 (2.22)

Supplementary Figure 1: Description of the study design used to identify sibling broiler chickens characterized by divergent feed conversion ratio (FCR). Figure adapted from Shah et al., 2016.

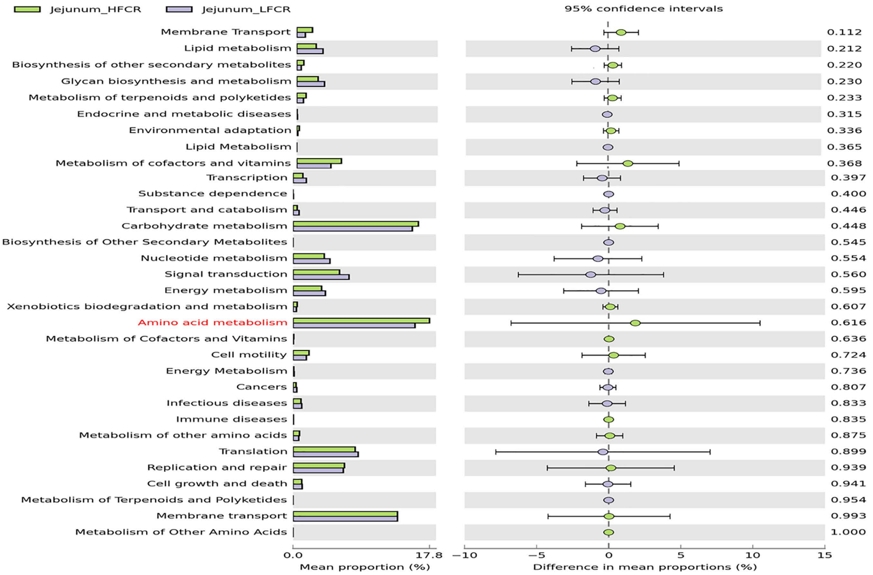

Supplementary Figure 2: Mean proportion and their differences in functional metagenomics profiles of the jejunum microbiome of low and high FCR broilers.

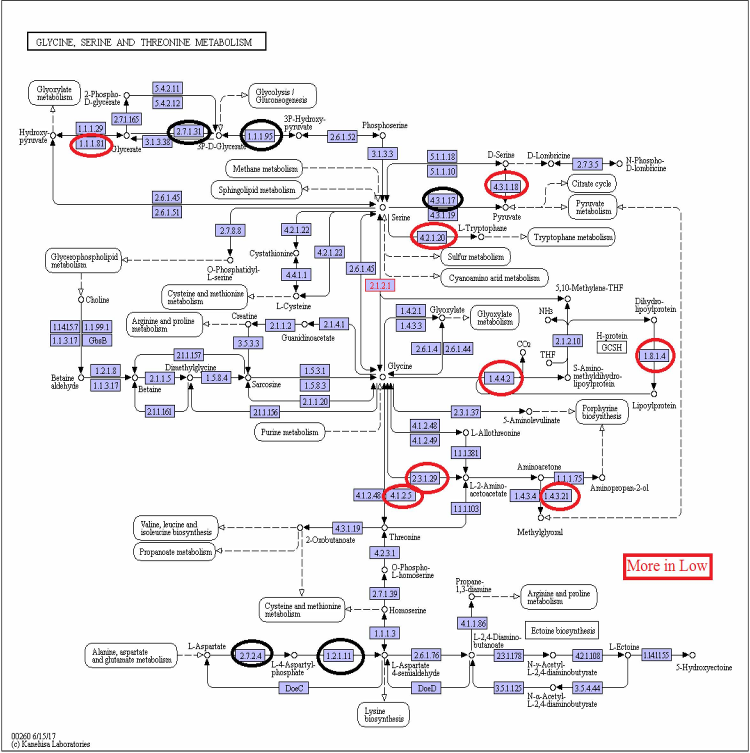

Supplementary Figure 3: Genes involved in Glycine, Serine and Threonine metabolism from functional metagenomics profiles of the ileum microbiome of low and high FCR broilers.
